# Human subtelomeric duplicon structure and organization

**DOI:** 10.1186/gb-2007-8-7-r151

**Published:** 2007-07-30

**Authors:** Anthony Ambrosini, Sheila Paul, Sufen Hu, Harold Riethman

**Affiliations:** 1 The Wistar Institute, Spruce St, Philadelphia, PA 19104, USA; 2 Department of Molecular Biology, Princeton University, Princeton, NJ 08544, USA

## Abstract

**Background:**

Human subtelomeric segmental duplications ('subtelomeric repeats') comprise about 25% of the most distal 500 kb and 80% of the most distal 100 kb in human DNA. A systematic analysis of the duplication substructure of human subtelomeric regions was done in order to develop a detailed understanding of subtelomeric sequence organization and a nucleotide sequence-level characterization of subtelomeric duplicon families.

**Results:**

The extent of nucleotide sequence divergence within subtelomeric duplicon families varies considerably, as does the organization of duplicon blocks at subtelomere alleles. Subtelomeric internal (TTAGGG)n-like tracts occur at duplicon boundaries, suggesting their involvement in the generation of the complex sequence organization. Most duplicons have copies at both subtelomere and non-subtelomere locations, but a class of duplicon blocks is identified that are subtelomere-specific. In addition, a group of six subterminal duplicon families are identified that, together with six single-copy telomere-adjacent segments, include all of the (TTAGGG)n-adjacent sequence identified so far in the human genome.

**Conclusion:**

Identification of a class of duplicon blocks that is subtelomere-specific will facilitate high-resolution analysis of subtelomere repeat copy number variation as well as studies involving somatic subtelomere rearrangements. The significant levels of nucleotide sequence divergence within many duplicon families as well as the differential organization of duplicon blocks on subtelomere alleles may provide opportunities for allele-specific subtelomere marker development; this is especially true for subterminal regions, where divergence and organizational differences are the greatest. These subterminal sequence families comprise the immediate cis-elements for (TTAGGG)n tracts, and are prime candidates for subtelomeric sequences regulating telomere-specific (TTAGGG)n tract length in humans.

## Abstract 2

The sequence divergence within subtelomeric duplicon families varies considerably, as does the organization of duplicon blocks at subtelomere alleles; a class of duplicon blocks was identified that are subtelomere-specific.

## Background

Segmental duplications, defined operationally as duplicated stretches of genomic DNA at least 1 kb in length with >90% nucleotide sequence identity, comprise roughly 5% of euchromatin in the human genome [1]. They are preferential sites of genomic instability, associated with recurrent pathology-associated chromosome breakpoints [2], large-scale copy number polymorphisms [3,4], and evolutionary chromosome breakpoint regions [5]. While they are distributed throughout the human genome, they tend to cluster near centromeres and telomeres [1].

Human subtelomeric segmental duplications ('subtelomeric repeats') comprise about 25% of the most distal 500 kb and 80% of the most distal 100 kb in human DNA [1,6]. From extensive early work on these complex regions it was recognized that telomere-adjacent sequence stretches contained low copy subtelomeric repeat segments of varying sizes and degrees of divergence [7,8]. The first completed sequences of human subtelomere regions revealed at least two general classes of duplicons, sometimes separated by internal (TTAGGG)n-like islands; large and highly similar centromerically positioned subtelomere duplications and more abundant, dissimilar distal duplicons [9]. While it is now well-established that subtelomeric repeat (Srpt) regions are composed of mosaic patchworks of duplicons [10,11], genome-wide analyses of these regions are revealing new details. The patchworks of subtelomeric duplicons appear to arise from translocations involving the tips of chromosomes, followed by transmission of unbalanced chromosomal complements to offspring [12]. The overall size, sequence content, and organization of subtelomeric segmental duplications relative to the terminal (TTAGGG)n repeat tracts and to subtelomeric single-copy DNA are different for each subtelomere [6], and the large-scale polymorphisms (50 kb to 500 kb) found near many human telomeres seem to be due primarily to variant combinations of subtelomeric segmental duplications [10,11,13]. Thus, the architecture of each human subtelomere region is determined largely by its specific subtelomeric segmental duplication content and organization, which vary from telomere to telomere and are often allele-specific.

Terminal (TTAGGG)n tracts lie immediately distal to subtelomeric segmental duplication regions and form the ends of chromosomes. The lengths of (TTAGGG)n tracts have been shown to vary from telomere to telomere within individual cells [14-16] and between alleles at the same telomere [17-19]. Individual-specific patterns of relative telomere-specific (TTAGGG)n tract lengths have a significant heritable component closely associated with the telomeres themselves [19,20], and these patterns appear to be defined in the zygote and maintained throughout life [16]. Since the immediate effects of (TTAGGG)n tract loss on cell viability and chromosome stability may be attributable to the shortest telomere(s) in a cell, rather than to average telomere length [18,21], individual-specific patterns of allele-specific (TTAGGG)n tract lengths may be crucial for the biological functions of telomeres and the effects of telomere attrition and dysfunction associated with aging, cancer, stress and coronary artery disease [22-24].

The overall picture of duplicated subtelomeric DNA that has emerged is one of a very plastic and rapidly evolving genome compartment. Some of the DNA segments within this subtelomeric compartment can exchange sequences with each other inter-chromosomally [12]; these genomic fragments behave essentially as a multi-allelic subtelomeric gene family, with paralogs on separate subtelomeres sometimes sharing higher sequence similarity than alleles on homologous chromosomes. Thus, in order to track individual subtelomere alleles in these regions, it will be essential to define markers that can distinguish the allele not just from its homolog, but from each of its paralogs. This is a fundamental challenge in developing subtelomeric markers, and one that requires a detailed understanding of both subtelomeric sequence organization and the nucleotide sequence-level characterization of duplicon families. We therefore set out to characterize these features systematically based upon the available human DNA sequence.

## Results

### Subtelomeric duplicon definition

Subtelomeric regions of human chromosomes are known to be composed, in part, of mosaic patchworks of duplicons [10-12,25]. In order to analyze their sequence organization in a systematic manner, we developed a set of rules to identify modules of DNA defined by sequence similarity between segments of subtelomeric DNA from single telomeres and the assembled human genome. A hybrid reference genome composed of 500 kb subtelomere assemblies [6] incorporated into human genome build 35 at the appropriate subtelomere coordinates (Additional data file 1) was used for this purpose. The hybrid build used in the current analysis essentially replaces some of the build 35 subtelomeres with more complete and rigorously validated subtelomere assemblies [6], but is otherwise identical to the build 35 public reference sequence.

The sequence of the most distal 500 kb of each human subtelomere region from this reference hybrid build was used to query the complete hybrid reference genome sequence as described in Materials and methods and in Additional data file 2. Adjacent and properly oriented BLAST matches with ≥90% nucleotide sequence identity and ≥1 kb in size were assembled into chains; the query sequence and each aligned region identified in this manner were termed 'duplicons' defined by that query, and this set of homologous sequences is a single 'module'. Each module was thus defined by a set of pairwise alignments with the query subtelomere sequence, and a percent nucleotide sequence identity for the non-masked parts of each chained pairwise alignment was derived from the BLAST alignments. In cases where more than one duplicon was defined by matches to a segment of subtelomere query sequence, the average percent identity of all pairwise alignments in the module was also calculated (the %ID_avg_). Interestingly, in most cases the best nucleotide sequence identity between the query subtelomere sequence and the duplicons was very similar to the average pairwise nucleotide sequence identity, indicating that either subtelomeric duplications within a group of this class occurred in a relatively narrow evolutionary time window, or gene conversion of duplicated sequences within the group has occurred at a relatively constant rate. The full set of modules, including the coordinates of their genomic alignments, is presented in Additional data file 3.

**Figure 1:**
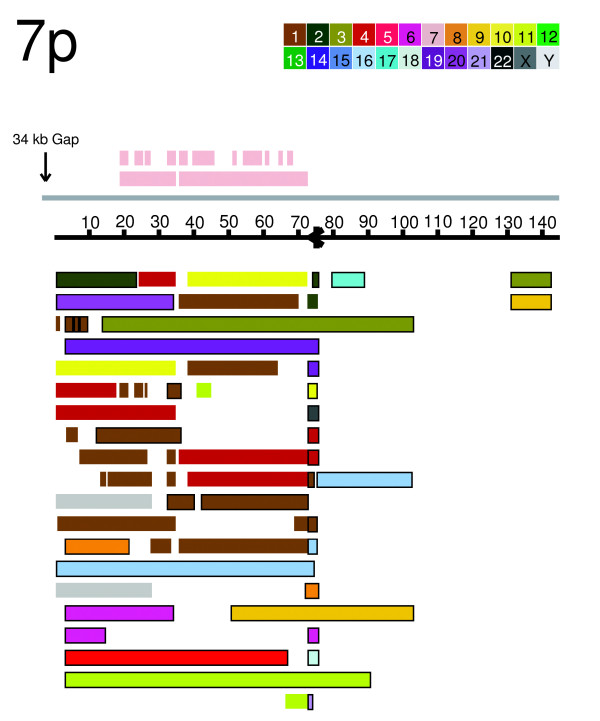
Duplicon substructure of the 7p subtelomere region. The most distal 140 kb of the chromosome 7p reference sequence is shown oriented with the telomeric end on the left (34 kb of unsequenced 7p DNA lie beyond the sequenced region shown, and the remaining 350 kb of the 7p subtelomere region centromeric to that shown does not contain duplicated DNA). The distance from the end of the sequence to the start of the terminal repeat array is indicated by the vertical arrow at the telomeric end of the sequence. The position and 5'-3' G-strand orientation of (TTAGGG)n elements are shown as black arrows. Duplicated genomic segments are identified by chromosome (color) and whether they are subtelomeric (bounded rectangles), non-telomeric (unbounded rectangles), or intra-chromosomal (located above the subtelomere coordinates).

Figure 1 illustrates this analysis graphically for the 7p subtelomere region. Each rectangle in Figure 1 represents a separate duplicon; for example, the chromosome 7 intrachromosomal duplicons (pink, above the coordinate line) include two large blocks and many smaller ones, with each duplicon corresponding to distinct, internal chromosome 7 coordinates. The large (90 kb) duplicon at the bottom of the figure matches a subtelomeric segment of chromosome 11 (bounded light green rectangle) whereas chromosome 1 is the site of 25 distinct 7ptel duplicons of various sizes, 9 of which are subtelomeric (bounded brown rectangles) and 16 non-subtelomeric (unbounded brown rectangles). The remaining duplicons defined by pairwise alignment with the 7ptel query sequence are designated in a similar fashion.

**Figure 2:**
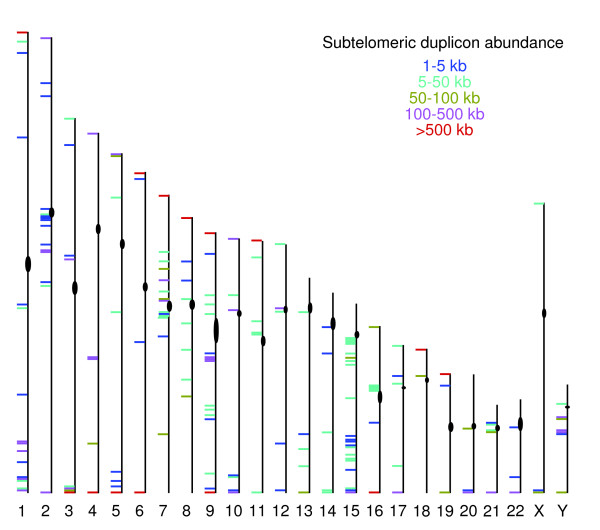
Genomic distribution of subtelomeric duplicons. The total number of duplicon bases for each 1 Mb interval in the human genome is indicated by the following color designations: red, greater than 500 kb; purple, 100-500 kb; aqua, 50-100 kb; green, 5-50 kb; and blue, 1-5 kb. The positions of the centromeric gaps in build 35 are indicated as black cylinders.

This systematic analysis resulted in the definition of 1,151 subtelomeric modules whose coordinates define duplicon families; 461 modules define duplicon families located exclusively in subtelomere regions, whereas the remainder have copies in both subtelomeric and non-subtelomeric DNA. The duplication module numbers are broken down by subtelomere in Additional data file 4. The abundance and genomic distribution for the subtelomere modules and each of their duplicons are summarized in Figure 2. In addition to the expected subtelomeric enrichment of duplicons, they are also localized at many pericentromeric loci and at a relatively small number of internal chromosome sites. Internal loci particularly enriched for subtelomeric duplicons include 2q13-q14 (at the site where ancestral primate telomeres fused to form modern human chromosome 2), 1q42.11-1q42.12, 1q42.13, 1q43-q44, 3p12.3, 3q29, 4q26, 7p13, 9q12-q13, and Yq11.23. These sites have been documented previously in genome-wide analyses of segmental duplications [26] and represent sites that were apparently susceptible to either donation or acceptance of these duplicated chromosome segments in recent evolutionary time.

### Subtelomeric duplicon characterization

The defined subtelomere modules and their duplicons were characterized according to size and nucleotide sequence similarity. Duplicons that occupy subtelomeric sequences were generally both larger and more abundant than those occurring elsewhere in the genome (Additional data file 5), consistent with the notion that subtelomeric location in humans is permissive for and/or somehow promotes large duplication events. Although smaller and fewer, non-subtelomeric copies of duplicons tended to cluster at the relatively few pericentric and interstitial loci described above (Figure 2).

**Figure 3:**
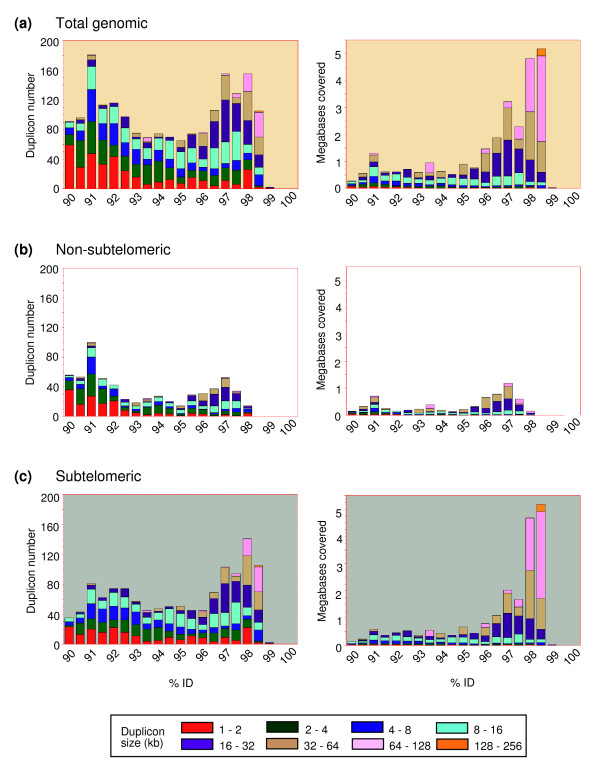
Duplicon number, size, and total bases covered as a function of percent nucleotide sequence identity. Duplicon number (left panels) and the total bases in duplicons (right panels) are shown on the Y-axis, and percent nucleotide sequence identity for the non-RepeatMasked bases is shown on the X-axis. The size ranges (kb) of duplicons in each category are indicated by the colors shown in the key at the bottom of the figure.

Figure 3 shows the results of an analysis of duplicon number as a function of percent nucleotide identity. There is a bimodal distribution of duplicon number versus percent nucleotide sequence identities, with peaks at 98% and 91% (Figure 3, left panels). The 98% peak was highly enriched in subtelomeric duplicons. The combined large size and high sequence similarity of a subset of subtelomeric duplicons is highlighted in the right panels of Figure 3, which plots the total bases covered by the duplicons as a function of the nucleotide sequence identity. The bimodal distribution of duplicon peaks might suggest two evolutionary waves of duplications, with the more recent one accounting for most of the large subtelomeric duplicons; this sort of punctuated duplication pattern is reminiscent of that observed by Eichler and co-workers [27] for segmentally duplicated DNA in a pericentromeric chromosome region. Alternatively, the 98% peak may be due to maintenance of sequence similarity by ongoing interchromosomal gene conversion between the large subtelomeric duplicons.

### Subtelomeric duplicon organization and divergence

**Figure 4:**
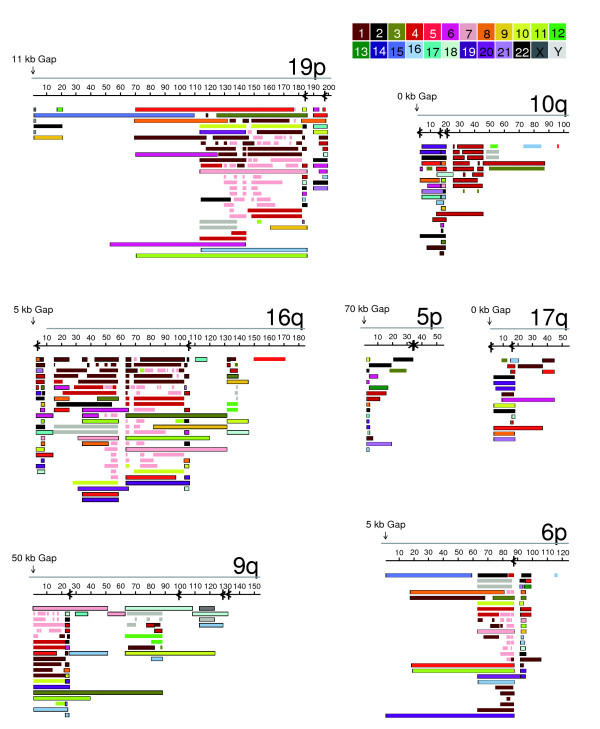
Duplicon organization of selected telomeres. The sequences are oriented with the telomeres at the left, with the distance from the end of the sequence to the start of the terminal repeat array indicated by the vertical arrow at the telomeric end of the sequence. The position and 5'-3' G-strand orientation of (TTAGGG)n elements are shown as black arrows. Note the co-localization of nearly all of the internal (TTAGGG)n islands with duplicon boundaries. The duplicon substructure for each of the 43 non-satellited telomeres is shown in Additional data files 6-47.

Visual inspection of the duplicon organization for the subtelomeres revealed several key features (Figure 4, Additional data files 6-47). The internal (TTAGGG)n sequences are usually oriented towards the telomere and almost always co-localize to duplicon boundaries. The orientations of the duplicons in the segmentally duplicated regions are similarly maintained, consistent with a recent model for their generation that features subtelomeric translocation of chromosome tips followed by transmission of unbalanced subtelomeric chromosome complements [12]. In an unusual case where the orientations are opposite to the telomere (Figure 4, 5p telomere), the (TTAGGG)n occurs head-to-head with one in the normal orientation, perhaps indicating the relic of a head-to-head telomere fusion event transmitted in the germline. Subtelomeric internal (TTAGGG)n-like sequences at duplicon boundaries suggest the possibility of internal binding/interaction sites for some (TTAGGG)n-binding protein components found primarily at terminal (TTAGGG)n tracts; published data showing TRF2 and TIN2 localization at internal (TTAGGG)n tracts resulting from a fused human chromosome pair support this idea [28]. The subtelomeric internal (TTAGGG)n-like islands range in size up to 823 base-pairs (bp), with most in the 150-200 bp range; they vary considerably in similarity to canonical (TTAGGG)n repeats [6] as well as in the relative abundance of (TTAGGG)n-related motifs. Several of the (TTAGGG)n-related motifs found in these islands were detected previously in proximal regions of telomeres (for example, TGAGGG, TCAGGG, TTGGGG [29,30] (H Riethman, unpublished)). A more detailed analysis of these interesting sequence islands and their comparison with a more comprehensive set of telomere-proximal sequences than is currently available might shed light on their origins and the relative timing of their internalization.

For any given segment of a subtelomere, the level of nucleotide sequence similarity with duplicated DNA depends entirely on the specific duplicon content and organization and does not necessarily correlate with its distance from the telomere terminus (Additional data files 6-47, bottom panels). Large duplicons with relatively high sequence similarity amongst family members cover a large proportion of the duplicated sequence space, but occupy only a subset of subtelomere regions and exist at variable distances from the terminal (TTAGGG)n tract. Since many of the currently incomplete assemblies terminate within these large duplicons, the actual sequence organization is still unknown for these chromosome ends (1p, 3q, 6p, 7p, 8p, 9q, 11p, 19p). For assemblies completed or very nearly completed that contain the large duplicons, there is a consistent pattern of higher divergence in (TTAGGG)n-adjacent subterminal sequence than in adjacent large duplicon regions (4q, 5q, 6q, 10q, 15q, 16q, and 17q, bottom panels). For subtelomeres that lack the large duplicons, there is typically a much lower degree of sequence similarity throughout these subtelomeric duplication regions (often 90-96% nucleotide sequence identity; 1q, 2p, 4p, 5p, 10p, 13q, 14q, 18p, 19q, 21q, 22q). The 3p, 14q, and 20p subtelomeres have unsequenced gaps adjacent to their terminal (TTAGGG)n tracts; hybridization experiments showed that 3p and 14q have small Srpt regions, whereas that for 20p is more extensive and contains large duplicons (H Riethman, data not shown).

The duplicon sequence similarity characteristics of a small group of telomeres falls outside of the general patterns mentioned above. The 16p reference allele subtelomere and the Xq/Yq subtelomere have small, highly similar subterminal duplicons and more divergent adjacent subtelomeric ones, whereas the 2q, 12p, 17p, and 20q subtelomeres have moderately sized duplicons with <96% to 98.5% similarity throughout the duplicated regions. The 9p subtelomere has subterminal duplicons with high sequence similarity (98.5-99%) and several large blocks of sequence that correspond to the 2qfus internal site and several internal loci on chromosome 9 (Additional data file 22) [31].

The telomere assemblies analyzed here represent only a single reference sequence, and there is extensive evidence for large copy number polymorphism at many of these chromosome ends [32-35]. Known major variant alleles differ quite dramatically in sequence organization from the shown reference alleles. For example, the 16p allele shown is one of at least three large variants of this subtelomere [32]; finished sequence data from part of a second allele show the presence of additional duplicated DNA sequences, including several large duplicons bearing very high sequence similarity (97-98.5%) with those characterized in this study (data not shown). Similarly, the 11p reference allele assembly shown here is part of a long segmental variant of this subtelomere; the short version (whose existence has been validated by cloning and mapping (H Riethman, data not shown)) ends at an internal (TTAGGG)n sequence present within the long allele (coordinate 115 kb), and has a structure similar to the 17p subtelomere (compare Additional data files 26 and 37). As additional variant subtelomeres are cloned and characterized, it is likely that further combinations of duplicons will be discovered on alleles that may, in many instances, be more similar to their paralogs than their homologs.

### Subtelomere-only sequence blocks

Systematic analysis of each subtelomere revealed a limited set of subtelomeric segments whose sequence aligned exclusively with other subtelomeric DNA sequences (detailed in Additional data file 48). The 11 largest stretches of these subtelomere-only duplicon blocks, each greater than 10 kb in length, are summarized in Table 1. The size and subtelomere origin of the largest homology block for each of these duplicon families is indicated, along with the number of copies and the range of pairwise nucleotide sequence identities of the subtelomere alignments to the query DNA segments. It should be noted that some of the duplicons are smaller than the largest query block, either because they are missing some of the sequences or because they are from the edge of an incomplete subtelomeric sequence assembly. The subtel-only blocks include portions of the largest duplicated regions with highest sequence similarity among copies (blocks 1, 2, 3, 6, 6', and 12) in addition to several blocks with somewhat lower sequence similarity among copies. Because they are restricted exclusively to subtelomeres and are of sufficient size and sequence similarity to be detected by FISH-based approaches, this class of duplicon blocks is an attractive starting point for developing subtelomere paint probes for tracking somatic changes to subtelomeres *in situ*. Their delineation here will permit the development of sequence-based copy number quantification assays to assist in the analysis of subtelomere allele dosage changes in both germline DNA and the somatic evolution of genomes in cancer.

**Table 1:** Large subtelomere-specific duplicons

Subtel block	Telomere	Size (kb)	Duplicated blocks	Percent identity	Named transcripts
1	1p	25	4	97.15-98.42	Sim to protein phosphatase 1 inhibitor subunit 2
2	15q	88	7	97.84-98.33	OR4F3, OR4F4, OR4F5, OR4F29, OR4F21, OR4F16, OR4F17, C6orf88
3	1p	35	1	97	
5	2p	17	5	90.49-92.00	Sim to RPL23AP7
6	3q	38	5	97.15-97.89	Sim to RPL23AP7
6'	11p	11	1	97.98	RYD5
7	4q	28	1	*	DUX4
8	4q	14	6	91.33-94.60	TUBB4q
10	2q	49	1	96.47	FBXO25
11	9q	36	6	91.26 - 95.68	IL9R
12	12p	15	1	97.89	IQSEC3

* Block 7 corresponds to the D4Z4 tandem repeat on the 4q and 10q subtelomeres, for which no percent identity is calculated because of the very large number and diverse % identities of the BLAST alignments among tandem D4Z4 repeats.

### Subterminal sequence blocks

Adjacent to some of the terminal (TTAGGG)n sequences and to many internal (TTAGGG)n sequences are stacks of small duplicons (for example, 7p in Figure 1, 19p, 10q, 16q, 9q, 6p in Figure 4, and telomeres 2p, 3q, 4p, 4q, 5q, 6q, 8p, 11p, 17q, 18p, 19q, 21q, 22q in Additional data files 6-47). This subterminal duplicon class has sequence similarity to DNA positioned adjacent to the terminal (TTAGGG)n tract of at least one chromosome end. To more formally define these sequences, we examined the duplicon structure of each of the finished and near-finished (within 5 kb of the terminal (TTAGGG)n) subtelomere assemblies [6,36] and identified subterminal sequence segments that are flanked by terminal (TTAGGG)n and by a position <25 kb from the terminal (TTAGGG)n that corresponds to a boundary of multiple duplicons. These sequences were termed subterminal modules and were used as query sequence to define subterminal duplicons that contained sequence aligned to them using the criteria outlined in Additional data file 2. Six subterminal duplicon families were defined in this manner (Additional data file 49). Together with six one-copy DNA (TTAGGG)n-adjacent regions (7q, 8q, 11q, 12q, 18q, and Xp/Yp), these duplicon families represent the global set of sequences occupying the DNA space immediately cis to terminal (TTAGGG)n tracts. As such, they are among the sequences most likely to directly impact terminal (TTAGGG)n tract regulation [19].

Table 2 shows the telomere and the defining subterminal segment sizes for these six duplicon families, as well as the copy number for each family. The copies are categorized according to those that occur in other subterminal regions (<25 kb from any known terminal (TTAGGG)n tract; subterm), those that occur in subtelomeric repeat regions but are not subterminal (subtel), and those that occur in non-subtelomeric regions (non_subtel). Subterminal duplicons that occur at internal subtelomeric sites are often adjacent to internal (TTAGGG)n tracts and are evident graphically as stacks of duplicated DNA segments (for example, 7p in Figure 1, and 19p, 10q, 16q, 9q, and 6p in Figure 4). However, some duplicons in such stacks are bounded by an internal (TTAGGG)n and some are not. The same situation can be visualized at several subtelomeric sites defined by stacks of subterminal duplicons but that lack internal (TTAGGG)n (for example, telomere 5p in Figure 4, and telomeres 1p, 1q, 5p, 6q, 16q from Additional data files 6-47). The simplest explanation for these observations is that these duplicon edges correspond to the positions of terminal translocations where (TTAGGG)n sequences on the recipient telomeres were lost or where the (TTAGGG)n motif was originally present but has decayed beyond recognition.

**Table 2:** Subterminal duplicons

Subterm block	Telomere	Size (kb)	Duplicated blocks	Location	%ID	Named transcripts
A	2p	7	6	Subterm	91.74-92.46	Sim to RPL23AP7, FAM41C
A	2p	7	12	Subtel	91.24 - 92.65	Sim to RPL23AP7, FAM41C
A	2p	7	1	Non_subtel	91.8	Sim to RPL23AP7, FAM41C
						
B	4p	17	10	Subterm	90.67-98.39	Sim to RPL23AP7, FAM41C
B	4p	17	16	Subtel	90.57-93.66	Sim to RPL23AP7, FAM41C
B	4p	17	1	Non_subtel	91.9	Sim to RPL23AP7, FAM41C
						
C	9p	10	6	Subterm	98.29-99.00	Sim to MGC13005, sim to DDX11, CXYorf1-related
C	9p	10	1	Non_subtel	98.27	Sim to MGC13005, sim to DDX11, CXYorf1-related
						
D	10q	22	10	Subterm	90.7-96.65	Sim to RPL23AP7, FAM41C
D	10q	22	15	Subtel	91.68-96.09	Sim to RPL23AP7, FAM41C
D	10q	22	2	Non_subtel	93.69-95.80	Sim to RPL23AP7, FAM41C
						
E	17p	21	5	Subterm	95.97-97.16	
						
F	18p	15	1	Subterm	99.00	
F	18p	15	1	Subtel	93.58	
F	18p	15	8	Non_subtel	91.19-94.27	

A limited set of non-subtelomeric copies of subterminal duplicons also exist (Table 2, Additional data file 49). Their genomic locations suggest sites of ancestral telomere-associated chromosome rearrangements, including a well-documented telomere fusion at 2q13-q14 [37] and ancestral inversion of a chromosome arm followed by duplication of pericentromeric sequences (see legend to Additional data file 49).

The relationship between subterminal duplicon copies within a family and between several related subterminal families (also detailed in the legend to Additional data file 49) is complex and broadly consistent with an earlier model of subtelomere structure (based upon the first completely sequenced subtelomeres) featuring a subterminal 'compartment' with more active recombinational features than the larger and less abundant centromerically positioned subtelomere duplications [9]. In particular, many of the subterminal intra-family and cross-family homology regions are relatively short, their positions within the subterminal blocks vary, and they are located at different distances from the terminal (TTAGGG)n tract. In addition, there are several alternative organizations of high-copy repetitive elements (masked and not examined in detail in this study) within these subterminal blocks. Further refinement of the classification of these subterminal families appears feasible and will benefit from more extensive sampling of (TTAGGG)n-adjacent sequences from additional alleles.

## Discussion

Tracking subtelomere alleles using conventional DNA markers is currently very difficult. All but six of the most distal 30 kb euchromatic subtelomere segments are composed exclusively of segmental duplications, and for a significant number of subtelomeres the duplication regions can be far more extensive (hundreds of kilobases) as well as highly variable in size and duplication content among alleles. Most of this subtelomeric DNA lies outside of the 'Hapmappable' genome; using single nucleotide polymorphisms to follow haplotypes in these regions is virtually impossible using current high-throughput technologies because of subtelomeric duplication content. Our high-resolution analysis of subtelomeric duplication sequence content and organization demonstrates significant differences in the levels of sequence similarity between distinct subtelomere duplicon families as well as large variations in the types and sequence organization of duplicons present at particular subtelomeres. These differences may offer opportunities for distinguishing individual subtelomere alleles in the context of genomic DNA samples, ultimately permitting large-scale studies associating subtelomere haplotypes or haplotype combinations with particular phenotypes.

Our analysis of subtelomeric duplicon substructure and nucleotide sequence similarity provides a different and more detailed perspective on subtelomere sequence organization than the subtelomere paralogy analysis included as part of the Linardopoulou *et al*. [12] study. The starting point for our analysis was a comprehensive set of manually curated and physically mapped subtelomere sequence assemblies [6], and we incorporated all segmental duplications of the subtelomeric sequences (both non-subtelomeric and subtelomeric) into our duplicon definition and analysis strategy; this led to the systematic and comprehensive definition and sequence characterization of duplicons anchored to each subtelomere (Additional data files 6-47). The paralogy map derived from the Linardopoulou *et al*. [12] analysis does not incorporate non-subtelomeric homology blocks or the newer subtelomeric sequence included in our assemblies. Because of these differences, the paralogy blocks they define overlap with, but do not correspond to, any of the subtel-only blocks or subterminal blocks defined in this study (Additional data file 50). In addition, we determined raw percent nucleotide sequence similarity numbers directly from the pairwise blastn alignments of RepeatMasked sequence, rather than calculating this parameter from alignments of non-RepeatMasked DNA post-processed to exclude gaps and small insertions/deletions from alignment percent identity scoring [12]. This accounts for the generally higher divergence between our duplicon sequence alignments compared to those of Linardopoulou *et al*. [12], and helps to focus attention on sequence differences most likely to be useful for allelic and paralog discrimination.

Duplicons and sets of adjacent duplicon blocks that comprise segmentally duplicated subtelomeric DNA were classified according to several practically useful and perhaps biologically significant groups. Duplicon blocks that occur only in subtelomeric regions (Table 1) can be used to develop sequence-based approaches to the analysis of subtelomere variation and subtelomeric somatic evolution of individual genomes, without interfering background signals from non-subtelomeric sites. Subterminal duplicon blocks of sequence (Table 2) were defined that, together with six one-copy subterminal regions, comprise all of the cis-elements adjacent to terminal (TTAGGG)n tracts. These sequences are believed to be involved in telomere-specific and allele-specific (TTAGGG)n tract regulation [19], and are amongst the first non-(TTAGGG)n sequences expected to be affected by telomere dysfunction, aberrant telomere replication, and telomere instability. Their delineation and analysis of their variation are crucial for understanding the role of human subtelomeres in telomere length regulation and telomere biology.

Subtelomeric duplicons are known to harbor protein-encoding genes and predicted protein-encoding genes as well as pseudogenes and many transcripts of unknown function [6,12,35] (H Riethman, unpublished). Known genes embedded in the subtelomere-specific duplicons and in the subterminal duplicons are listed in Tables 1 and 2, respectively; a comprehensive listing of RefSeq matches with these duplicons is given in Additional data files 51 and 52. For several subtelomeric transcript families (IL9R, DUX4, FBXO25) functional evidence for protein expression from at least one transcript locus is available [38-40]. However, for most transcript families the evidence for encoded protein function relies upon the existence of one or more actively transcribed loci with open reading frames predicted to encode evolutionarily conserved proteins [41-44]. While these data strongly suggest that one or more members of each of these gene families encode functional protein, in most cases pseudogene copies of the respective gene family co-exist amongst the duplicons and a great deal of work lies ahead in terms of deciphering the functions of individual members of subtelomeric gene families as well as their evolution. In this light, it is important to note that only a single reference sequence has been sampled in this analysis, and given the abundant large-scale variation in these regions, there are certain to be many additional members of most of these gene families yet to be discovered in the human population.

One of the most intriguing transcript families embedded in the subtelomere repeat region is one predicted to encode odorant receptors [35,41], in subtelomere-specific duplicon block 2 (Table 1). The highly variable dosage and polymorphic distribution of these genes in humans reflect a recent and evolutionarily rapid expansion of this gene family. Subtelomeric duplicon regions of yeast, Plasmodium, and trypanosomes are each associated with rapid duplication and generation of functional diversity in their embedded genes (discussed in [10]), and it is intriguing to speculate that similar mechanisms are active in human evolution. A very interesting transcript family of unknown function (CXYorf1-related) is embedded in subterminal duplicon block C (Table 2); many of these transcripts are predicted to encode variants of an evolutionarily conserved open reading frame with one copy in the mouse genome [44]. This transcript family varies widely in both dosage and telomere distribution in individual genomes, and usually terminates less than 5 kb from the start of the terminal (TTAGGG)n tract; thus, individual telomeric transcription sites for this family might be differentially susceptible to position effects depending on local telomeric chromatin/heterochromatin status and on chromosome-specific telomere lengths.

From our analysis, it is clear that most subterminal duplicon sequences are more divergent than the large duplicons that exist more centromerically, both in nucleotide sequence similarity and in sequence organization. This divergence might be exploited to develop subterminal allele-specific PCR assays to track some of these sequences genetically in the context of total genomic DNA. For both the highly similar and the more divergent duplicon families, coupling quantitative PCR assays designed to amplify sequences across these regions with new bead-based single molecule characterization and sequencing methods [45,46] might provide an extremely powerful means for determining both the copy number and a global set of short-range subtelomere haplotypes within an individual genome. Thus, subtelomere variation might be linked with phenotypes at this level. Extending these global short-range sequence haplotypes into longer-range subtelomere allele haplotypes will be more challenging, and may require the isolation, detailed characterization, and perhaps complete sequencing of many additional variant subtelomere alleles.

## Conclusion

This comprehensive analysis of the segmental duplication substructure in human subtelomere regions yielded a number of insights with important biological implications. The localization of interstitial subtelomeric (TTAGGG)n-like sequences at duplicon boundaries suggests their involvement in the generation of the complex sequence organization. Their existence at subtelomeres suggests the possibility of internal binding/interaction sites for some (TTAGGG)n-binding protein components found primarily at terminal (TTAGGG)n tracts. Identification of a class of duplicon blocks that are subtelomere-specific will facilitate high-resolution analysis of subtelomere repeat copy number variation as well as studies involving somatic subtelomere rearrangements. Finally, the significant levels of nucleotide sequence divergence within many duplicon families as well as the differential organization of duplicon blocks on subtelomere alleles may provide opportunities for allele-specific subtelomere marker development; this is especially true for subterminal regions, where divergence and organizational differences are the greatest. These subterminal sequence families comprise the immediate cis-elements for (TTAGGG)n tracts, and are prime candidates for subtelomeric sequences regulating telomere-specific (TTAGGG)n tract length in humans. Their delineation and analysis of their variation will be crucial for understanding the role of human subtelomeres in telomere length regulation and telomere biology.

## Materials and methods

### 'Hybrid' genome build

Both build 35 subtelomeres and the Riethman *et al*. [6] subtelomere sequences are based upon the same mapping data [6,36], but the manually curated subtelomere assemblies [6] are more complete, containing some subtelomere sequences missing and/or misincorporated in the public builds. A single hybrid reference genome was therefore created and used in the current analysis, so that duplicons could be identified and consistently defined in the context of the highest quality sequence available. The centromeric single-copy regions of our assemblies matched build 35 perfectly, so the 500 kb subtelomeric assemblies [6] (see also Riethman Lab Website [47]) were substituted for build 35 sequence at the appropriate sequence coordinates (given in Additional data file 1; for each of the non-acrocentric chromosome ends the appropriate p-arm sequence was attached at the p-arm coordinate. The reverse complement of the q-arm sequences were attached at the indicated q-arm coordinates).

### Rules for modules of BLAST hits

Duplicon modules were defined by processing the results of BLAST [48] searches of in-house curated subtelomere sequence with repeats masked by RepeatMasker [49] and Tandem Repeats Finder [50] against the hybrid build 35 genome build described above. Blast hits (≥90% identity and ≥100 bp length) were segregated according to chromosomal location and orientation. Any blast hits that were colinear, within 25 kb of each other in both loci, and uninterrupted by other hits from the same group were combined to form these duplicons. Our methods were tolerant of large insertions and deletions (for example, of retrotransposons) but not rearrangements. Groups of combined blast hits ≥1 kb were defined as duplicons, and those smaller were discarded. The percent identity of each pairwise alignment was derived directly from the blastn output; no post-processing of alignments to remove small insertions and deletions as described by Linardopoulou *et al*. [12] was done.

### Subtel-only block definition and characterization

The master module list (Additional data file 3) was scanned for regions in which the query sequences shared homology with other subtelomeres but not any non-subtelomeric regions. A representative was taken from the longest stretch of query associated with each of these regions. This subsequence was passed through the module definition pipeline described above (Additional data file 2) to give sets of duplicons whose boundaries correspond precisely with the delineated subsequence.

### Subterminal block definition and characterization

We examined the duplicon structure (Figures 1 and 4, Additional data files 6-47) of each of the finished and near-finished subtelomere assemblies (finished to within 5 kb of the terminal (TTAGGG)n) [6] and identified subterminal sequence segments that are flanked at one end by a terminal (TTAGGG)n and at the other by a position within 25 kb of the terminal (TTAGGG)n that corresponds to the boundary of multiple duplicons. These sequence blocks were used as query sequence to define subterminal duplicons that contained sequence aligned to the query subterminal block using the criteria outlined in Additional data file 2. The six subterminal families represent a minimally redundant set of such subterminal blocks.

## Additional data files

The following additional data are available with the online version of this paper. Additional data file 1 provides coordinates of build 35 to which the 500 kb subtelomeric [6] assemblies were added prior to the subtelomeric duplicon analysis. Additional data file 2 is a definition of subtelomeric duplicons. Additional data file 3 is a table giving duplicon definition and characterization. Additional data file 4 is a summary of modules defined by similarity to human subtelomeric DNA. Additional data file 5 gives the number and size range of duplicons found in non-subtelomeric genome regions and in subtelomeric genome regions. Additional data files 6, 7, 8, 9, 10, 11, 12, 13, 14, 15, 16, 17, 18, 19, 20, 21, 22, 23, 24, 25, 26, 27, 28, 29, 30, 31, 32, 33, 34, 35, 36, 37, 38, 39, 40, 41, 42, 43, 44, 45, 46, 47 show the duplicons defined in the terminal 500 kb of all non-satellited telomeres (1p-Yq); each has a top panel and a bottom panel, with the top panel showing duplicon origin and organization and the bottom panel showing the % nucleotide sequence similarity for each of these duplicons. Additional data file 48 is a table listing duplicon blocks that are specific for subtelomeric regions of the human genome. Additional data file 49 is a table listing duplicon blocks that are adjacent to terminal (TTAGGG)n repeats. Additional data file 50 is a Comparison of subtel-only and subterminal duplicon blocks defined in this work with the subtelomeric homology blocks reported in Linardopoulou *et al*. [12]. Additional data file 51 is a table listing subtel-only block transcript matches. Additional data file 52 is a table listing subterminal block transcript matches.

## Supplementary Material

Additional data file 1 The p-arm sequence as given was attached at the p-arm coordinate, and the reverse complement of the q-arm sequences were attached at the indicated q-arm coordinates Click here for file

Additional data file 2 Duplicon modules were defined by processing the results of BLAST searches of in-house curated subtelomere query sequences (see text and Materials and methods). Colinear and properly oriented pairs of BLAST matches to the query sequence were joined into a chain if not separated by greater than 25 kb and not uninterrupted by other hits from the same query sequence. Groups of chained blast hits spanning ≥1 kb of the subject sequence were defined as duplicons. These methods were tolerant of insertions and deletions <25 kb in size (for example, of retrotransposons) but not tolerant of rearrangements. Click here for file

Additional data file 3 Each module is defined by a set of pairwise alignments, and each reference sequence in these sets is represented as a single row in this table. The first column (module) contains an identifier for the particular copy of the module (duplicon) indicated in the next three columns. These columns (query sequence) list the subtelomeric location of the query sequence defining the module (see Materials and methods). The 'aligned sequences' column shows the locations of other duplicons in this module, matched by the query. The coordinates in this column refer either to our published subtelomeric assemblies (designated by chromosome and arm p or q) or the human genome build 35 (all other designations). The %ID_each _is percent nucleotide sequence identity across the chained pairwise alignment, excluding masked sequence. The %ID_avg _is the average percent identity of all pairwise alignments in the module. This was the number used for %ID in charts and analyses in this paper. The final column shows a 1 if the module contains intrachromosomal non-subtelomeric sequence matches, and 0 if it does not. Click here for file

Additional data file 4 This table shows the numbers of duplicon modules defined per subtelomere. The complete list of these modules is included in Additional data file 3. The 'subtelomeric' column shows the total number of modules for each subtelomere region (since each module is defined by a set of subtelomeric coordinates). The 'non-subtelomeric' column lists the subset of these modules with homology to duplicated regions that lie outside the subtelomeres. A comparison of these non-subtelomeric duplicons to the subtelomeric copies is included in Figure 3 and in Additional data file 5. The 'intra-chromosomal' column indicates the subset of modules with homology to a different region on the same chromosome. Click here for file

Additional data file 5 Subtelomeric regions correspond to the set of query sequences enumerated in Additional data file 1 and the average percent identity across the sequences to which each is aligned. The non-subtelomeric regions correspond to the aligned sequences that fall outside the subtelomere regions (the subset listed in Additional data file 2). Click here for file

Additional data file 6 The subtelomere sequences shown are the assemblies published previously [6] and are available at the Riethman Lab website [47]. The telomeric end of each sequence assembly is located at the left. The distance from the end of the sequence to the start of the terminal repeat array is indicated by the vertical arrow at the telomeric end of the sequence. The position and orientation of (TTAGGG)n tracts are shown as black arrows. Top panels: duplicated genomic segments are identified by chromosome (color) and whether they are subtelomeric (bounded rectangles), non-subtelomeric (unbounded rectangles), or intra-chromosomal (located above the subtelomere coordinates). Each rectangle represents a separate duplicon. Bottom panels: duplicated genomic segments are the same as in the top panels, but identified by nucleotide sequence similarity with the query subtelomere sequence (color scheme as indicated in the key). Click here for file

Additional data file 7 The subtelomere sequences shown are the assemblies published previously [6] and are available at the Riethman Lab website [47]. The telomeric end of each sequence assembly is located at the left. The distance from the end of the sequence to the start of the terminal repeat array is indicated by the vertical arrow at the telomeric end of the sequence. The position and orientation of (TTAGGG)n tracts are shown as black arrows. Top panels: duplicated genomic segments are identified by chromosome (color) and whether they are subtelomeric (bounded rectangles), non-subtelomeric (unbounded rectangles), or intra-chromosomal (located above the subtelomere coordinates). Each rectangle represents a separate duplicon. Bottom panels: duplicated genomic segments are the same as in the top panels, but identified by nucleotide sequence similarity with the query subtelomere sequence (color scheme as indicated in the key). Click here for file

Additional data file 8 The subtelomere sequences shown are the assemblies published previously [6] and are available at the Riethman Lab website [47]. The telomeric end of each sequence assembly is located at the left. The distance from the end of the sequence to the start of the terminal repeat array is indicated by the vertical arrow at the telomeric end of the sequence. The position and orientation of (TTAGGG)n tracts are shown as black arrows. Top panels: duplicated genomic segments are identified by chromosome (color) and whether they are subtelomeric (bounded rectangles), non-subtelomeric (unbounded rectangles), or intra-chromosomal (located above the subtelomere coordinates). Each rectangle represents a separate duplicon. Bottom panels: duplicated genomic segments are the same as in the top panels, but identified by nucleotide sequence similarity with the query subtelomere sequence (color scheme as indicated in the key). Click here for file

Additional data file 9 The subtelomere sequences shown are the assemblies published previously [6] and are available at the Riethman Lab website [47]. The telomeric end of each sequence assembly is located at the left. The distance from the end of the sequence to the start of the terminal repeat array is indicated by the vertical arrow at the telomeric end of the sequence. The position and orientation of (TTAGGG)n tracts are shown as black arrows. Top panels: duplicated genomic segments are identified by chromosome (color) and whether they are subtelomeric (bounded rectangles), non-subtelomeric (unbounded rectangles), or intra-chromosomal (located above the subtelomere coordinates). Each rectangle represents a separate duplicon. Bottom panels: duplicated genomic segments are the same as in the top panels, but identified by nucleotide sequence similarity with the query subtelomere sequence (color scheme as indicated in the key). Click here for file

Additional data file 10 The subtelomere sequences shown are the assemblies published previously [6] and are available at the Riethman Lab website [47]. The telomeric end of each sequence assembly is located at the left. The distance from the end of the sequence to the start of the terminal repeat array is indicated by the vertical arrow at the telomeric end of the sequence. The position and orientation of (TTAGGG)n tracts are shown as black arrows. Top panels: duplicated genomic segments are identified by chromosome (color) and whether they are subtelomeric (bounded rectangles), non-subtelomeric (unbounded rectangles), or intra-chromosomal (located above the subtelomere coordinates). Each rectangle represents a separate duplicon. Bottom panels: duplicated genomic segments are the same as in the top panels, but identified by nucleotide sequence similarity with the query subtelomere sequence (color scheme as indicated in the key). Click here for file

Additional data file 11 The subtelomere sequences shown are the assemblies published previously [6] and are available at the Riethman Lab website [47]. The telomeric end of each sequence assembly is located at the left. The distance from the end of the sequence to the start of the terminal repeat array is indicated by the vertical arrow at the telomeric end of the sequence. The position and orientation of (TTAGGG)n tracts are shown as black arrows. Top panels: duplicated genomic segments are identified by chromosome (color) and whether they are subtelomeric (bounded rectangles), non-subtelomeric (unbounded rectangles), or intra-chromosomal (located above the subtelomere coordinates). Each rectangle represents a separate duplicon. Bottom panels: duplicated genomic segments are the same as in the top panels, but identified by nucleotide sequence similarity with the query subtelomere sequence (color scheme as indicated in the key). Click here for file

Additional data file 12 The subtelomere sequences shown are the assemblies published previously [6] and are available at the Riethman Lab website [47]. The telomeric end of each sequence assembly is located at the left. The distance from the end of the sequence to the start of the terminal repeat array is indicated by the vertical arrow at the telomeric end of the sequence. The position and orientation of (TTAGGG)n tracts are shown as black arrows. Top panels: duplicated genomic segments are identified by chromosome (color) and whether they are subtelomeric (bounded rectangles), non-subtelomeric (unbounded rectangles), or intra-chromosomal (located above the subtelomere coordinates). Each rectangle represents a separate duplicon. Bottom panels: duplicated genomic segments are the same as in the top panels, but identified by nucleotide sequence similarity with the query subtelomere sequence (color scheme as indicated in the key). Click here for file

Additional data file 13 The subtelomere sequences shown are the assemblies published previously [6] and are available at the Riethman Lab website [47]. The telomeric end of each sequence assembly is located at the left. The distance from the end of the sequence to the start of the terminal repeat array is indicated by the vertical arrow at the telomeric end of the sequence. The position and orientation of (TTAGGG)n tracts are shown as black arrows. Top panels: duplicated genomic segments are identified by chromosome (color) and whether they are subtelomeric (bounded rectangles), non-subtelomeric (unbounded rectangles), or intra-chromosomal (located above the subtelomere coordinates). Each rectangle represents a separate duplicon. Bottom panels: duplicated genomic segments are the same as in the top panels, but identified by nucleotide sequence similarity with the query subtelomere sequence (color scheme as indicated in the key). Click here for file

Additional data file 14 The subtelomere sequences shown are the assemblies published previously [6] and are available at the Riethman Lab website [47]. The telomeric end of each sequence assembly is located at the left. The distance from the end of the sequence to the start of the terminal repeat array is indicated by the vertical arrow at the telomeric end of the sequence. The position and orientation of (TTAGGG)n tracts are shown as black arrows. Top panels: duplicated genomic segments are identified by chromosome (color) and whether they are subtelomeric (bounded rectangles), non-subtelomeric (unbounded rectangles), or intra-chromosomal (located above the subtelomere coordinates). Each rectangle represents a separate duplicon. Bottom panels: duplicated genomic segments are the same as in the top panels, but identified by nucleotide sequence similarity with the query subtelomere sequence (color scheme as indicated in the key). Click here for file

Additional data file 15 The subtelomere sequences shown are the assemblies published previously [6] and are available at the Riethman Lab website [47]. The telomeric end of each sequence assembly is located at the left. The distance from the end of the sequence to the start of the terminal repeat array is indicated by the vertical arrow at the telomeric end of the sequence. The position and orientation of (TTAGGG)n tracts are shown as black arrows. Top panels: duplicated genomic segments are identified by chromosome (color) and whether they are subtelomeric (bounded rectangles), non-subtelomeric (unbounded rectangles), or intra-chromosomal (located above the subtelomere coordinates). Each rectangle represents a separate duplicon. Bottom panels: duplicated genomic segments are the same as in the top panels, but identified by nucleotide sequence similarity with the query subtelomere sequence (color scheme as indicated in the key). Click here for file

Additional data file 16 The subtelomere sequences shown are the assemblies published previously [6] and are available at the Riethman Lab website [47]. The telomeric end of each sequence assembly is located at the left. The distance from the end of the sequence to the start of the terminal repeat array is indicated by the vertical arrow at the telomeric end of the sequence. The position and orientation of (TTAGGG)n tracts are shown as black arrows. Top panels: duplicated genomic segments are identified by chromosome (color) and whether they are subtelomeric (bounded rectangles), non-subtelomeric (unbounded rectangles), or intra-chromosomal (located above the subtelomere coordinates). Each rectangle represents a separate duplicon. Bottom panels: duplicated genomic segments are the same as in the top panels, but identified by nucleotide sequence similarity with the query subtelomere sequence (color scheme as indicated in the key). Click here for file

Additional data file 17 The subtelomere sequences shown are the assemblies published previously [6] and are available at the Riethman Lab website [47]. The telomeric end of each sequence assembly is located at the left. The distance from the end of the sequence to the start of the terminal repeat array is indicated by the vertical arrow at the telomeric end of the sequence. The position and orientation of (TTAGGG)n tracts are shown as black arrows. Top panels: duplicated genomic segments are identified by chromosome (color) and whether they are subtelomeric (bounded rectangles), non-subtelomeric (unbounded rectangles), or intra-chromosomal (located above the subtelomere coordinates). Each rectangle represents a separate duplicon. Bottom panels: duplicated genomic segments are the same as in the top panels, but identified by nucleotide sequence similarity with the query subtelomere sequence (color scheme as indicated in the key). Click here for file

Additional data file 18 The subtelomere sequences shown are the assemblies published previously [6] and are available at the Riethman Lab website [47]. The telomeric end of each sequence assembly is located at the left. The distance from the end of the sequence to the start of the terminal repeat array is indicated by the vertical arrow at the telomeric end of the sequence. The position and orientation of (TTAGGG)n tracts are shown as black arrows. Top panels: duplicated genomic segments are identified by chromosome (color) and whether they are subtelomeric (bounded rectangles), non-subtelomeric (unbounded rectangles), or intra-chromosomal (located above the subtelomere coordinates). Each rectangle represents a separate duplicon. Bottom panels: duplicated genomic segments are the same as in the top panels, but identified by nucleotide sequence similarity with the query subtelomere sequence (color scheme as indicated in the key). Click here for file

Additional data file 19 The subtelomere sequences shown are the assemblies published previously [6] and are available at the Riethman Lab website [47]. The telomeric end of each sequence assembly is located at the left. The distance from the end of the sequence to the start of the terminal repeat array is indicated by the vertical arrow at the telomeric end of the sequence. The position and orientation of (TTAGGG)n tracts are shown as black arrows. Top panels: duplicated genomic segments are identified by chromosome (color) and whether they are subtelomeric (bounded rectangles), non-subtelomeric (unbounded rectangles), or intra-chromosomal (located above the subtelomere coordinates). Each rectangle represents a separate duplicon. Bottom panels: duplicated genomic segments are the same as in the top panels, but identified by nucleotide sequence similarity with the query subtelomere sequence (color scheme as indicated in the key). Click here for file

Additional data file 20 The subtelomere sequences shown are the assemblies published previously [6] and are available at the Riethman Lab website [47]. The telomeric end of each sequence assembly is located at the left. The distance from the end of the sequence to the start of the terminal repeat array is indicated by the vertical arrow at the telomeric end of the sequence. The position and orientation of (TTAGGG)n tracts are shown as black arrows. Top panels: duplicated genomic segments are identified by chromosome (color) and whether they are subtelomeric (bounded rectangles), non-subtelomeric (unbounded rectangles), or intra-chromosomal (located above the subtelomere coordinates). Each rectangle represents a separate duplicon. Bottom panels: duplicated genomic segments are the same as in the top panels, but identified by nucleotide sequence similarity with the query subtelomere sequence (color scheme as indicated in the key). Click here for file

Additional data file 21 The subtelomere sequences shown are the assemblies published previously [6] and are available at the Riethman Lab website [47]. The telomeric end of each sequence assembly is located at the left. The distance from the end of the sequence to the start of the terminal repeat array is indicated by the vertical arrow at the telomeric end of the sequence. The position and orientation of (TTAGGG)n tracts are shown as black arrows. Top panels: duplicated genomic segments are identified by chromosome (color) and whether they are subtelomeric (bounded rectangles), non-subtelomeric (unbounded rectangles), or intra-chromosomal (located above the subtelomere coordinates). Each rectangle represents a separate duplicon. Bottom panels: duplicated genomic segments are the same as in the top panels, but identified by nucleotide sequence similarity with the query subtelomere sequence (color scheme as indicated in the key). Click here for file

Additional data file 22 The subtelomere sequences shown are the assemblies published previously [6] and are available at the Riethman Lab website [47]. The telomeric end of each sequence assembly is located at the left. The distance from the end of the sequence to the start of the terminal repeat array is indicated by the vertical arrow at the telomeric end of the sequence. The position and orientation of (TTAGGG)n tracts are shown as black arrows. Top panels: duplicated genomic segments are identified by chromosome (color) and whether they are subtelomeric (bounded rectangles), non-subtelomeric (unbounded rectangles), or intra-chromosomal (located above the subtelomere coordinates). Each rectangle represents a separate duplicon. Bottom panels: duplicated genomic segments are the same as in the top panels, but identified by nucleotide sequence similarity with the query subtelomere sequence (color scheme as indicated in the key). Click here for file

Additional data file 23 The subtelomere sequences shown are the assemblies published previously [6] and are available at the Riethman Lab website [47]. The telomeric end of each sequence assembly is located at the left. The distance from the end of the sequence to the start of the terminal repeat array is indicated by the vertical arrow at the telomeric end of the sequence. The position and orientation of (TTAGGG)n tracts are shown as black arrows. Top panels: duplicated genomic segments are identified by chromosome (color) and whether they are subtelomeric (bounded rectangles), non-subtelomeric (unbounded rectangles), or intra-chromosomal (located above the subtelomere coordinates). Each rectangle represents a separate duplicon. Bottom panels: duplicated genomic segments are the same as in the top panels, but identified by nucleotide sequence similarity with the query subtelomere sequence (color scheme as indicated in the key). Click here for file

Additional data file 24 The subtelomere sequences shown are the assemblies published previously [6] and are available at the Riethman Lab website [47]. The telomeric end of each sequence assembly is located at the left. The distance from the end of the sequence to the start of the terminal repeat array is indicated by the vertical arrow at the telomeric end of the sequence. The position and orientation of (TTAGGG)n tracts are shown as black arrows. Top panels: duplicated genomic segments are identified by chromosome (color) and whether they are subtelomeric (bounded rectangles), non-subtelomeric (unbounded rectangles), or intra-chromosomal (located above the subtelomere coordinates). Each rectangle represents a separate duplicon. Bottom panels: duplicated genomic segments are the same as in the top panels, but identified by nucleotide sequence similarity with the query subtelomere sequence (color scheme as indicated in the key). Click here for file

Additional data file 25 The subtelomere sequences shown are the assemblies published previously [6] and are available at the Riethman Lab website [47]. The telomeric end of each sequence assembly is located at the left. The distance from the end of the sequence to the start of the terminal repeat array is indicated by the vertical arrow at the telomeric end of the sequence. The position and orientation of (TTAGGG)n tracts are shown as black arrows. Top panels: duplicated genomic segments are identified by chromosome (color) and whether they are subtelomeric (bounded rectangles), non-subtelomeric (unbounded rectangles), or intra-chromosomal (located above the subtelomere coordinates). Each rectangle represents a separate duplicon. Bottom panels: duplicated genomic segments are the same as in the top panels, but identified by nucleotide sequence similarity with the query subtelomere sequence (color scheme as indicated in the key). Click here for file

Additional data file 26 The subtelomere sequences shown are the assemblies published previously [6] and are available at the Riethman Lab website [47]. The telomeric end of each sequence assembly is located at the left. The distance from the end of the sequence to the start of the terminal repeat array is indicated by the vertical arrow at the telomeric end of the sequence. The position and orientation of (TTAGGG)n tracts are shown as black arrows. Top panels: duplicated genomic segments are identified by chromosome (color) and whether they are subtelomeric (bounded rectangles), non-subtelomeric (unbounded rectangles), or intra-chromosomal (located above the subtelomere coordinates). Each rectangle represents a separate duplicon. Bottom panels: duplicated genomic segments are the same as in the top panels, but identified by nucleotide sequence similarity with the query subtelomere sequence (color scheme as indicated in the key). Click here for file

Additional data file 27 The subtelomere sequences shown are the assemblies published previously [6] and are available at the Riethman Lab website [47]. The telomeric end of each sequence assembly is located at the left. The distance from the end of the sequence to the start of the terminal repeat array is indicated by the vertical arrow at the telomeric end of the sequence. The position and orientation of (TTAGGG)n tracts are shown as black arrows. Top panels: duplicated genomic segments are identified by chromosome (color) and whether they are subtelomeric (bounded rectangles), non-subtelomeric (unbounded rectangles), or intra-chromosomal (located above the subtelomere coordinates). Each rectangle represents a separate duplicon. Bottom panels: duplicated genomic segments are the same as in the top panels, but identified by nucleotide sequence similarity with the query subtelomere sequence (color scheme as indicated in the key). Click here for file

Additional data file 28 The subtelomere sequences shown are the assemblies published previously [6] and are available at the Riethman Lab website [47]. The telomeric end of each sequence assembly is located at the left. The distance from the end of the sequence to the start of the terminal repeat array is indicated by the vertical arrow at the telomeric end of the sequence. The position and orientation of (TTAGGG)n tracts are shown as black arrows. Top panels: duplicated genomic segments are identified by chromosome (color) and whether they are subtelomeric (bounded rectangles), non-subtelomeric (unbounded rectangles), or intra-chromosomal (located above the subtelomere coordinates). Each rectangle represents a separate duplicon. Bottom panels: duplicated genomic segments are the same as in the top panels, but identified by nucleotide sequence similarity with the query subtelomere sequence (color scheme as indicated in the key). Click here for file

Additional data file 29 The subtelomere sequences shown are the assemblies published previously [6] and are available at the Riethman Lab website [47]. The telomeric end of each sequence assembly is located at the left. The distance from the end of the sequence to the start of the terminal repeat array is indicated by the vertical arrow at the telomeric end of the sequence. The position and orientation of (TTAGGG)n tracts are shown as black arrows. Top panels: duplicated genomic segments are identified by chromosome (color) and whether they are subtelomeric (bounded rectangles), non-subtelomeric (unbounded rectangles), or intra-chromosomal (located above the subtelomere coordinates). Each rectangle represents a separate duplicon. Bottom panels: duplicated genomic segments are the same as in the top panels, but identified by nucleotide sequence similarity with the query subtelomere sequence (color scheme as indicated in the key). Click here for file

Additional data file 30 The subtelomere sequences shown are the assemblies published previously [6] and are available at the Riethman Lab website [47]. The telomeric end of each sequence assembly is located at the left. The distance from the end of the sequence to the start of the terminal repeat array is indicated by the vertical arrow at the telomeric end of the sequence. The position and orientation of (TTAGGG)n tracts are shown as black arrows. Top panels: duplicated genomic segments are identified by chromosome (color) and whether they are subtelomeric (bounded rectangles), non-subtelomeric (unbounded rectangles), or intra-chromosomal (located above the subtelomere coordinates). Each rectangle represents a separate duplicon. Bottom panels: duplicated genomic segments are the same as in the top panels, but identified by nucleotide sequence similarity with the query subtelomere sequence (color scheme as indicated in the key). Click here for file

Additional data file 31 The subtelomere sequences shown are the assemblies published previously [6] and are available at the Riethman Lab website [47]. The telomeric end of each sequence assembly is located at the left. The distance from the end of the sequence to the start of the terminal repeat array is indicated by the vertical arrow at the telomeric end of the sequence. The position and orientation of (TTAGGG)n tracts are shown as black arrows. Top panels: duplicated genomic segments are identified by chromosome (color) and whether they are subtelomeric (bounded rectangles), non-subtelomeric (unbounded rectangles), or intra-chromosomal (located above the subtelomere coordinates). Each rectangle represents a separate duplicon. Bottom panels: duplicated genomic segments are the same as in the top panels, but identified by nucleotide sequence similarity with the query subtelomere sequence (color scheme as indicated in the key). Click here for file

Additional data file 32 The subtelomere sequences shown are the assemblies published previously [6] and are available at the Riethman Lab website [47]. The telomeric end of each sequence assembly is located at the left. The distance from the end of the sequence to the start of the terminal repeat array is indicated by the vertical arrow at the telomeric end of the sequence. The position and orientation of (TTAGGG)n tracts are shown as black arrows. Top panels: duplicated genomic segments are identified by chromosome (color) and whether they are subtelomeric (bounded rectangles), non-subtelomeric (unbounded rectangles), or intra-chromosomal (located above the subtelomere coordinates). Each rectangle represents a separate duplicon. Bottom panels: duplicated genomic segments are the same as in the top panels, but identified by nucleotide sequence similarity with the query subtelomere sequence (color scheme as indicated in the key). Click here for file

Additional data file 33 The subtelomere sequences shown are the assemblies published previously [6] and are available at the Riethman Lab website [47]. The telomeric end of each sequence assembly is located at the left. The distance from the end of the sequence to the start of the terminal repeat array is indicated by the vertical arrow at the telomeric end of the sequence. The position and orientation of (TTAGGG)n tracts are shown as black arrows. Top panels: duplicated genomic segments are identified by chromosome (color) and whether they are subtelomeric (bounded rectangles), non-subtelomeric (unbounded rectangles), or intra-chromosomal (located above the subtelomere coordinates). Each rectangle represents a separate duplicon. Bottom panels: duplicated genomic segments are the same as in the top panels, but identified by nucleotide sequence similarity with the query subtelomere sequence (color scheme as indicated in the key). Click here for file

Additional data file 34 The subtelomere sequences shown are the assemblies published previously [6] and are available at the Riethman Lab website [47]. The telomeric end of each sequence assembly is located at the left. The distance from the end of the sequence to the start of the terminal repeat array is indicated by the vertical arrow at the telomeric end of the sequence. The position and orientation of (TTAGGG)n tracts are shown as black arrows. Top panels: duplicated genomic segments are identified by chromosome (color) and whether they are subtelomeric (bounded rectangles), non-subtelomeric (unbounded rectangles), or intra-chromosomal (located above the subtelomere coordinates). Each rectangle represents a separate duplicon. Bottom panels: duplicated genomic segments are the same as in the top panels, but identified by nucleotide sequence similarity with the query subtelomere sequence (color scheme as indicated in the key). Click here for file

Additional data file 35 The subtelomere sequences shown are the assemblies published previously [6] and are available at the Riethman Lab website [47]. The telomeric end of each sequence assembly is located at the left. The distance from the end of the sequence to the start of the terminal repeat array is indicated by the vertical arrow at the telomeric end of the sequence. The position and orientation of (TTAGGG)n tracts are shown as black arrows. Top panels: duplicated genomic segments are identified by chromosome (color) and whether they are subtelomeric (bounded rectangles), non-subtelomeric (unbounded rectangles), or intra-chromosomal (located above the subtelomere coordinates). Each rectangle represents a separate duplicon. Bottom panels: duplicated genomic segments are the same as in the top panels, but identified by nucleotide sequence similarity with the query subtelomere sequence (color scheme as indicated in the key). Click here for file

Additional data file 36 The subtelomere sequences shown are the assemblies published previously [6] and are available at the Riethman Lab website [47]. The telomeric end of each sequence assembly is located at the left. The distance from the end of the sequence to the start of the terminal repeat array is indicated by the vertical arrow at the telomeric end of the sequence. The position and orientation of (TTAGGG)n tracts are shown as black arrows. Top panels: duplicated genomic segments are identified by chromosome (color) and whether they are subtelomeric (bounded rectangles), non-subtelomeric (unbounded rectangles), or intra-chromosomal (located above the subtelomere coordinates). Each rectangle represents a separate duplicon. Bottom panels: duplicated genomic segments are the same as in the top panels, but identified by nucleotide sequence similarity with the query subtelomere sequence (color scheme as indicated in the key). Click here for file

Additional data file 37 The subtelomere sequences shown are the assemblies published previously [6] and are available at the Riethman Lab website [47]. The telomeric end of each sequence assembly is located at the left. The distance from the end of the sequence to the start of the terminal repeat array is indicated by the vertical arrow at the telomeric end of the sequence. The position and orientation of (TTAGGG)n tracts are shown as black arrows. Top panels: duplicated genomic segments are identified by chromosome (color) and whether they are subtelomeric (bounded rectangles), non-subtelomeric (unbounded rectangles), or intra-chromosomal (located above the subtelomere coordinates). Each rectangle represents a separate duplicon. Bottom panels: duplicated genomic segments are the same as in the top panels, but identified by nucleotide sequence similarity with the query subtelomere sequence (color scheme as indicated in the key). Click here for file

Additional data file 38 The subtelomere sequences shown are the assemblies published previously [6] and are available at the Riethman Lab website [47]. The telomeric end of each sequence assembly is located at the left. The distance from the end of the sequence to the start of the terminal repeat array is indicated by the vertical arrow at the telomeric end of the sequence. The position and orientation of (TTAGGG)n tracts are shown as black arrows. Top panels: duplicated genomic segments are identified by chromosome (color) and whether they are subtelomeric (bounded rectangles), non-subtelomeric (unbounded rectangles), or intra-chromosomal (located above the subtelomere coordinates). Each rectangle represents a separate duplicon. Bottom panels: duplicated genomic segments are the same as in the top panels, but identified by nucleotide sequence similarity with the query subtelomere sequence (color scheme as indicated in the key). Click here for file

Additional data file 39 The subtelomere sequences shown are the assemblies published previously [6] and are available at the Riethman Lab website [47]. The telomeric end of each sequence assembly is located at the left. The distance from the end of the sequence to the start of the terminal repeat array is indicated by the vertical arrow at the telomeric end of the sequence. The position and orientation of (TTAGGG)n tracts are shown as black arrows. Top panels: duplicated genomic segments are identified by chromosome (color) and whether they are subtelomeric (bounded rectangles), non-subtelomeric (unbounded rectangles), or intra-chromosomal (located above the subtelomere coordinates). Each rectangle represents a separate duplicon. Bottom panels: duplicated genomic segments are the same as in the top panels, but identified by nucleotide sequence similarity with the query subtelomere sequence (color scheme as indicated in the key). Click here for file

Additional data file 40 The subtelomere sequences shown are the assemblies published previously [6] and are available at the Riethman Lab website [47]. The telomeric end of each sequence assembly is located at the left. The distance from the end of the sequence to the start of the terminal repeat array is indicated by the vertical arrow at the telomeric end of the sequence. The position and orientation of (TTAGGG)n tracts are shown as black arrows. Top panels: duplicated genomic segments are identified by chromosome (color) and whether they are subtelomeric (bounded rectangles), non-subtelomeric (unbounded rectangles), or intra-chromosomal (located above the subtelomere coordinates). Each rectangle represents a separate duplicon. Bottom panels: duplicated genomic segments are the same as in the top panels, but identified by nucleotide sequence similarity with the query subtelomere sequence (color scheme as indicated in the key). Click here for file

Additional data file 41 The subtelomere sequences shown are the assemblies published previously [6] and are available at the Riethman Lab website [47]. The telomeric end of each sequence assembly is located at the left. The distance from the end of the sequence to the start of the terminal repeat array is indicated by the vertical arrow at the telomeric end of the sequence. The position and orientation of (TTAGGG)n tracts are shown as black arrows. Top panels: duplicated genomic segments are identified by chromosome (color) and whether they are subtelomeric (bounded rectangles), non-subtelomeric (unbounded rectangles), or intra-chromosomal (located above the subtelomere coordinates). Each rectangle represents a separate duplicon. Bottom panels: duplicated genomic segments are the same as in the top panels, but identified by nucleotide sequence similarity with the query subtelomere sequence (color scheme as indicated in the key). Click here for file

Additional data file 42 The subtelomere sequences shown are the assemblies published previously [6] and are available at the Riethman Lab website [47]. The telomeric end of each sequence assembly is located at the left. The distance from the end of the sequence to the start of the terminal repeat array is indicated by the vertical arrow at the telomeric end of the sequence. The position and orientation of (TTAGGG)n tracts are shown as black arrows. Top panels: duplicated genomic segments are identified by chromosome (color) and whether they are subtelomeric (bounded rectangles), non-subtelomeric (unbounded rectangles), or intra-chromosomal (located above the subtelomere coordinates). Each rectangle represents a separate duplicon. Bottom panels: duplicated genomic segments are the same as in the top panels, but identified by nucleotide sequence similarity with the query subtelomere sequence (color scheme as indicated in the key). Click here for file

Additional data file 43 The subtelomere sequences shown are the assemblies published previously [6] and are available at the Riethman Lab website [47]. The telomeric end of each sequence assembly is located at the left. The distance from the end of the sequence to the start of the terminal repeat array is indicated by the vertical arrow at the telomeric end of the sequence. The position and orientation of (TTAGGG)n tracts are shown as black arrows. Top panels: duplicated genomic segments are identified by chromosome (color) and whether they are subtelomeric (bounded rectangles), non-subtelomeric (unbounded rectangles), or intra-chromosomal (located above the subtelomere coordinates). Each rectangle represents a separate duplicon. Bottom panels: duplicated genomic segments are the same as in the top panels, but identified by nucleotide sequence similarity with the query subtelomere sequence (color scheme as indicated in the key). Click here for file

Additional data file 44 The subtelomere sequences shown are the assemblies published previously [6] and are available at the Riethman Lab website [47]. The telomeric end of each sequence assembly is located at the left. The distance from the end of the sequence to the start of the terminal repeat array is indicated by the vertical arrow at the telomeric end of the sequence. The position and orientation of (TTAGGG)n tracts are shown as black arrows. Top panels: duplicated genomic segments are identified by chromosome (color) and whether they are subtelomeric (bounded rectangles), non-subtelomeric (unbounded rectangles), or intra-chromosomal (located above the subtelomere coordinates). Each rectangle represents a separate duplicon. Bottom panels: duplicated genomic segments are the same as in the top panels, but identified by nucleotide sequence similarity with the query subtelomere sequence (color scheme as indicated in the key). Click here for file

Additional data file 45 The subtelomere sequences shown are the assemblies published previously [6] and are available at the Riethman Lab website [47]. The telomeric end of each sequence assembly is located at the left. The distance from the end of the sequence to the start of the terminal repeat array is indicated by the vertical arrow at the telomeric end of the sequence. The position and orientation of (TTAGGG)n tracts are shown as black arrows. Top panels: duplicated genomic segments are identified by chromosome (color) and whether they are subtelomeric (bounded rectangles), non-subtelomeric (unbounded rectangles), or intra-chromosomal (located above the subtelomere coordinates). Each rectangle represents a separate duplicon. Bottom panels: duplicated genomic segments are the same as in the top panels, but identified by nucleotide sequence similarity with the query subtelomere sequence (color scheme as indicated in the key). Click here for file

Additional data file 46 The subtelomere sequences shown are the assemblies published previously [6] and are available at the Riethman Lab website [47]. The telomeric end of each sequence assembly is located at the left. The distance from the end of the sequence to the start of the terminal repeat array is indicated by the vertical arrow at the telomeric end of the sequence. The position and orientation of (TTAGGG)n tracts are shown as black arrows. Top panels: duplicated genomic segments are identified by chromosome (color) and whether they are subtelomeric (bounded rectangles), non-subtelomeric (unbounded rectangles), or intra-chromosomal (located above the subtelomere coordinates). Each rectangle represents a separate duplicon. Bottom panels: duplicated genomic segments are the same as in the top panels, but identified by nucleotide sequence similarity with the query subtelomere sequence (color scheme as indicated in the key). Click here for file

Additional data file 47 The subtelomere sequences shown are the assemblies published previously [6] and are available at the Riethman Lab website [47]. The telomeric end of each sequence assembly is located at the left. The distance from the end of the sequence to the start of the terminal repeat array is indicated by the vertical arrow at the telomeric end of the sequence. The position and orientation of (TTAGGG)n tracts are shown as black arrows. Top panels: duplicated genomic segments are identified by chromosome (color) and whether they are subtelomeric (bounded rectangles), non-subtelomeric (unbounded rectangles), or intra-chromosomal (located above the subtelomere coordinates). Each rectangle represents a separate duplicon. Bottom panels: duplicated genomic segments are the same as in the top panels, but identified by nucleotide sequence similarity with the query subtelomere sequence (color scheme as indicated in the key). Click here for file

Additional data file 48 This table shows blocks of modules that occur exclusively in subtelomere regions. The first column gives an identifier for each block. The next three columns (query sequence) give the subtelomeric location that defines the block (which will consist of one or more adjacent modules). For completeness, in some cases aligned sequences have been included in these blocks even though they fell below thresholds for module definition. The percent identity of the chained alignments between the sequences is indicated (excluding masked sequence). Named genes/gene families that have transcripts matching part or all of the respective duplicon blocks are listed in the last column. Block 7 is the D4Z4 tandem repeat on the 4q and 10q subtelomeres, for which no percent identity is calculated because of the very large number and diverse percent identities of the BLAST alignments among tandem D4Z4 repeats. Click here for file

Additional data file 49 This table shows blocks of modules that are adjacent to the ends of finished telomeres (see Materials and methods). The columns describe the same categories of information as indicated in Additional data file 48. A limited set of non-subtelomeric copies of subterminal duplicons exist (Additional data file 49). Their genomic locations suggest sites of ancestral telomere-associated chromosome rearrangements, including a well-documented telomere fusion at 2q13-q14 [37] that contains representatives of subterminal duplicon families A, B, C, and D (Additional data file 49). The non-subtelomeric site of a duplicon from family D at 3p12.3 is the tip of an extended duplication region; the DNA on the centromeric flank of this site contains 4q and 10q subtelomere homology, including beta satellite repeat structure resembling part of the D4Z4 repeat. Subterminal family F contains several non-subtelomeric sites of duplicons; those on chromosomes 22q, 14q, and 12p are very close to the respective centromeres (Additional data file 49), indicating potential ancestral inversion of a chromosome arm followed by duplication of pericentromeric sequences as a mechanism for the genesis of the non-subterminal copies of this subterminal sequence family. The sequence similarity between subterminal duplicon copies within a family is mainly in the 90-96% range for subterminal blocks A, B, and D (Table 2; see Additional data file 49 for the rare exceptions.). As with the subtel-only blocks, some of these duplicons correspond to only part of the subterminal block sequence. There is also some overlap in sequences occupied by subterminal duplicon blocks A, B, and D; this is reflected in their occupancy of parts of the same transcript families RPL23A7 and FAM41C (Table 2). The cross-family homologies between subterminal blocks A, B, and D are also in the 90-96% identity range but the positions of the duplicons within the blocks vary and are located at different distances from the (TTAGGG)n tract; also, there are several alternative organizations of high-copy repetitive elements (masked and not examined in detail in this study) within these subterminal blocks. Thus, there might be more frequent shuffling of subterminal sequences than sequences located more centromerically, at least within a subset of subtelomere alleles; this idea is broadly consistent with an earlier model of subtelomere structure featuring compartments with distinct functional properties [9]. Further refinement of the classification of these subterminal families appears feasible and will benefit from more extensive sampling of (TTAGGG)n-adjacent sequences from additional alleles. Subterminal Block F contains one duplicon on 10p with very high similarity to the 18p query sequence, suggesting a very recent duplication event; the remaining duplicons were all in the 91-94% identity range. Block C has the highest sequence similarity among all subterminal duplicon sequence families, and has a copy at the 2q fusion locus. Block E (96-97%) is unusual in that it corresponds to a portion of subtelomere-only duplicon family 6 (Table 1), and is the only subterminal duplicon sequence family with subtel-only properties. This particular sequenced allele of 17p might have formed by the truncation of a chromosome end within this large subtelomere-only duplicon, as there is mapping evidence for several longer alleles of the 17p telomere (H Riethman, unpublished). It is interesting to note that (TTAGGG)n tracts at 17p and, indeed, on this particular allele of 17p tend to be consistently among the shortest in the human genome [19,51]. Click here for file

Additional data file 50 Comparison of subtel-only and subterminal duplicon blocks defined in this work with the subtelomeric homology blocks reported in Linardopoulou *et al*. [12] Click here for file

Additional data file 51 Candidate transcripts were identified by blasting the representative subtelomere-only query sequences (Additional data file 48) against the NCBI RefSeq mrna database (downloaded 24 July 2006) [52]. Human mRNAs with 90% or greater homology were run through Spidey [53] against the set of subtelomere-only duplicon block representatives. This table has been filtered to those hits above 95% identity according to the Spidey predictions. The first and second columns indicate the subtelomere-only block and RefSeq accession that align to each other. The third is the description line from the RefSeq database. The fourth and fifth columns are the percent identity and percent coverage of the aligned mRNA as reported by Spidey. Click here for file

Additional data file 52 Candidate transcripts were identified by blasting the representative subterminal query sequences (Additional data file 49) against the NCBI RefSeq mrna database (downloaded 24 July 2006) [52]. Human mRNAs with 90% or greater homology were run through Spidey [53] against the set of subterminal duplicon block representatives. The first and second columns indicate the subterminal block and RefSeq accession that align to each other. The third is the description line from the RefSeq database. The fourth and fifth columns are the percent identity and percent coverage of the aligned mRNA as reported by Spidey. Click here for file
