# Supplementary material for: Human subtelomeric duplicon structure and organization
Source: Genome Biol. 2007 Jul 30;8(7):R151. doi: 10.1186/gb-2007-8-7-r151 (PMC2323237; doi:10.1186/gb-2007-8-7-r151)
Supplement: Additional data file 1 — The p-arm sequence as given was attached at the p-arm coordinate, and the reverse complement of the q-arm sequences were attached at the indicated q-arm coordinates [file gb-2007-8-7-r151-S1.pdf]

Additional Data File 1. Hybrid Genome Coordinates

chr	p - cut	q - cut
chr1	1062370	244977446
chr2	500175	242315013
chr3	508216	198951071
chr4	500000	190901334
chr5	570131	180341779
chr6	505000	170472700
chr7	513631	158128301
chr8	536613	145775593
chr9	499099	137929465
chr10	550000	134913691
chr11	551333	133950513
chr12	515901	131889851
chr13	0	113632083
chr14	0	105851506
chr15	0	99838916
chr16	500000	88320853
chr17	465509	78294648
chr18	499161	75621849
chr19	510078	63310821
chr20	507039	61935965
chr21	0	46445316
chr22	0	48997402
chrX	637491	154324169
chrY	637491	57158604
