# Supplementary figures and images for: Human subtelomeric duplicon structure and organization
Source: Genome Biol. 2007 Jul 30;8(7):R151. doi: 10.1186/gb-2007-8-7-r151 (PMC2323237; doi:10.1186/gb-2007-8-7-r151)

Additional Data File 2. Definition of subtelomeric duplicons.

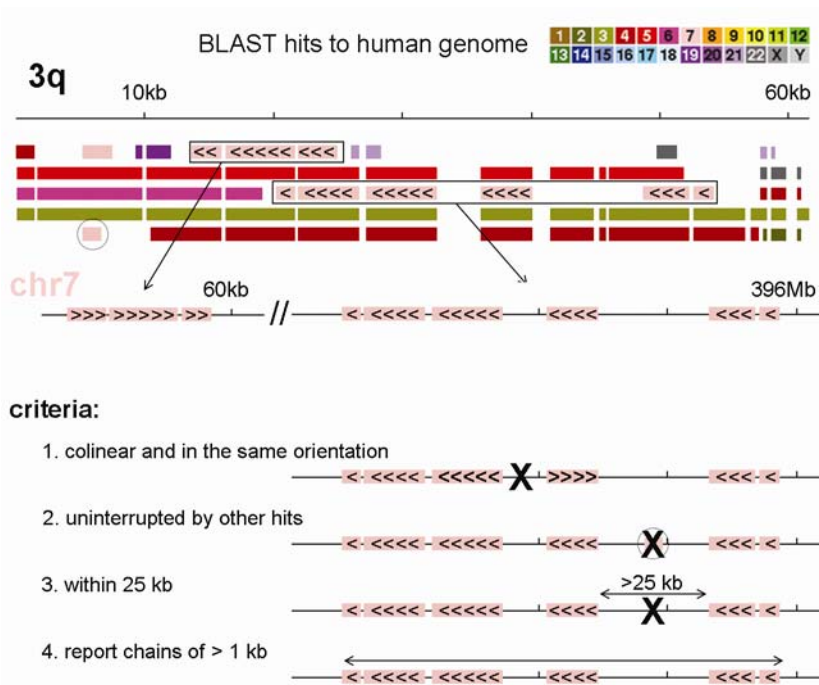

Supplement: Additional data file 2 — Duplicon modules were defined by processing the results of BLAST searches of in-house curated subtelomere query sequences (see text and Materials and methods). Colinear and properly oriented pairs of BLAST matches to the query sequence were joined into a chain if not separated by greater than 25 kb and not uninterrupted by other hits from the same query sequence. Groups of chained blast hits spanning ≥1 kb of the subject sequence were defined as duplicons. These methods were tolerant of insertions and deletions <25 kb in size (for example, of retrotransposons) but not tolerant of rearrangements. [file gb-2007-8-7-r151-S2.pdf]

Additional Data File 5. Number and Size Range of Duplicons.

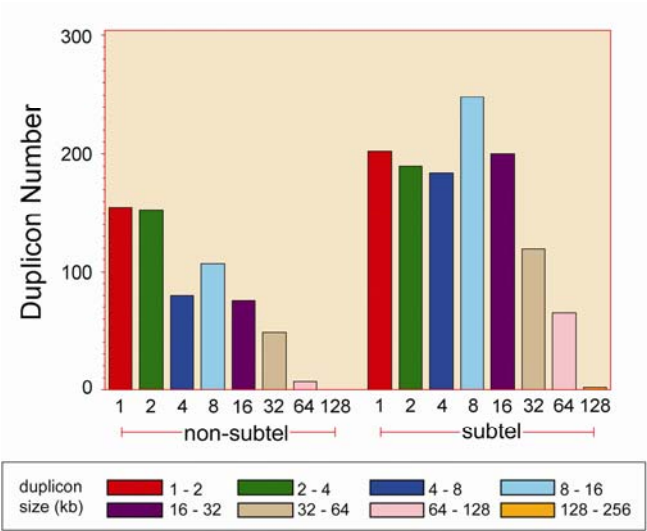

Supplement: Additional data file 5 — Subtelomeric regions correspond to the set of query sequences enumerated in Additional data file 1 and the average percent identity across the sequences to which each is aligned. The non-subtelomeric regions correspond to the aligned sequences that fall outside the subtelomere regions (the subset listed in Additional data file 2). [file gb-2007-8-7-r151-S5.pdf]

1p

15 kb Gap

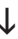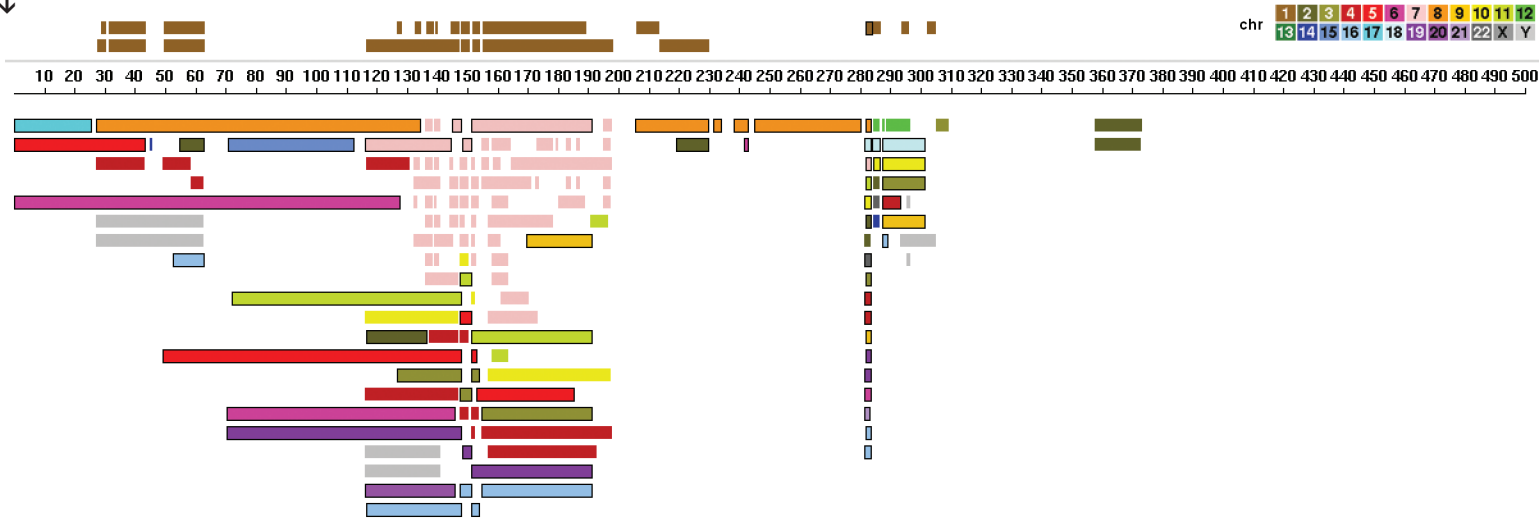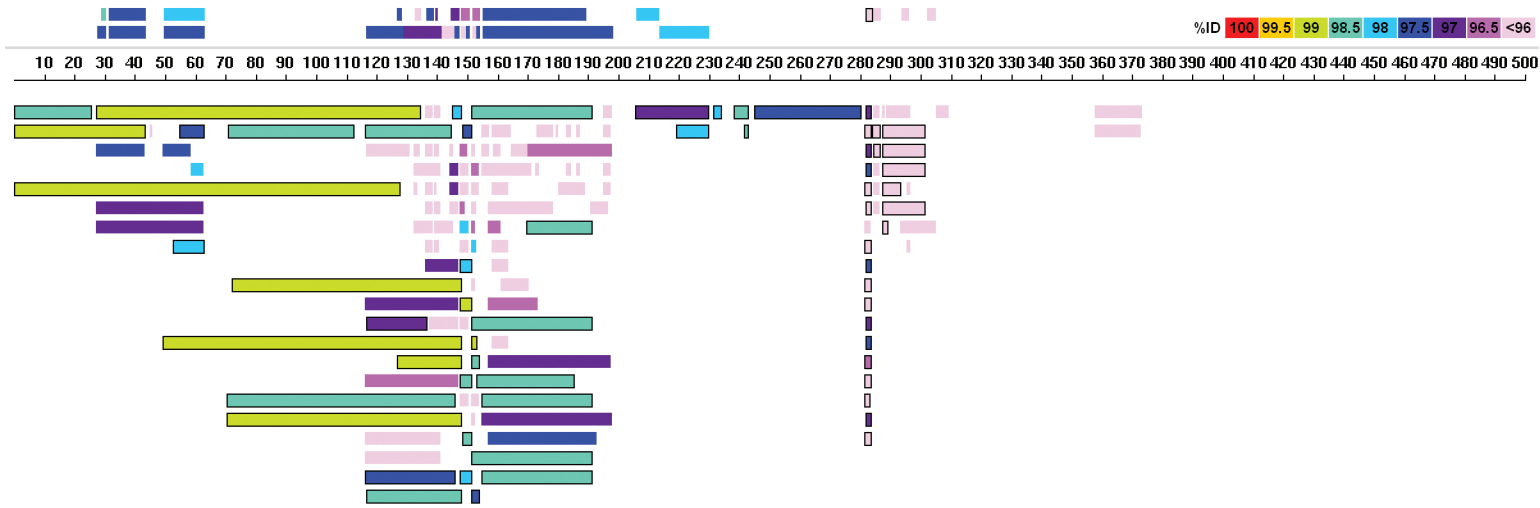

Supplement: Additional data file 6 — The subtelomere sequences shown are the assemblies published previously [6] and are available at the Riethman Lab website [47]. The telomeric end of each sequence assembly is located at the left. The distance from the end of the sequence to the start of the terminal repeat array is indicated by the vertical arrow at the telomeric end of the sequence. The position and orientation of (TTAGGG)n tracts are shown as black arrows. Top panels: duplicated genomic segments are identified by chromosome (color) and whether they are subtelomeric (bounded rectangles), non-subtelomeric (unbounded rectangles), or intra-chromosomal (located above the subtelomere coordinates). Each rectangle represents a separate duplicon. Bottom panels: duplicated genomic segments are the same as in the top panels, but identified by nucleotide sequence similarity with the query subtelomere sequence (color scheme as indicated in the key). [file gb-2007-8-7-r151-S6.pdf]

1q

10 kb Gap

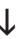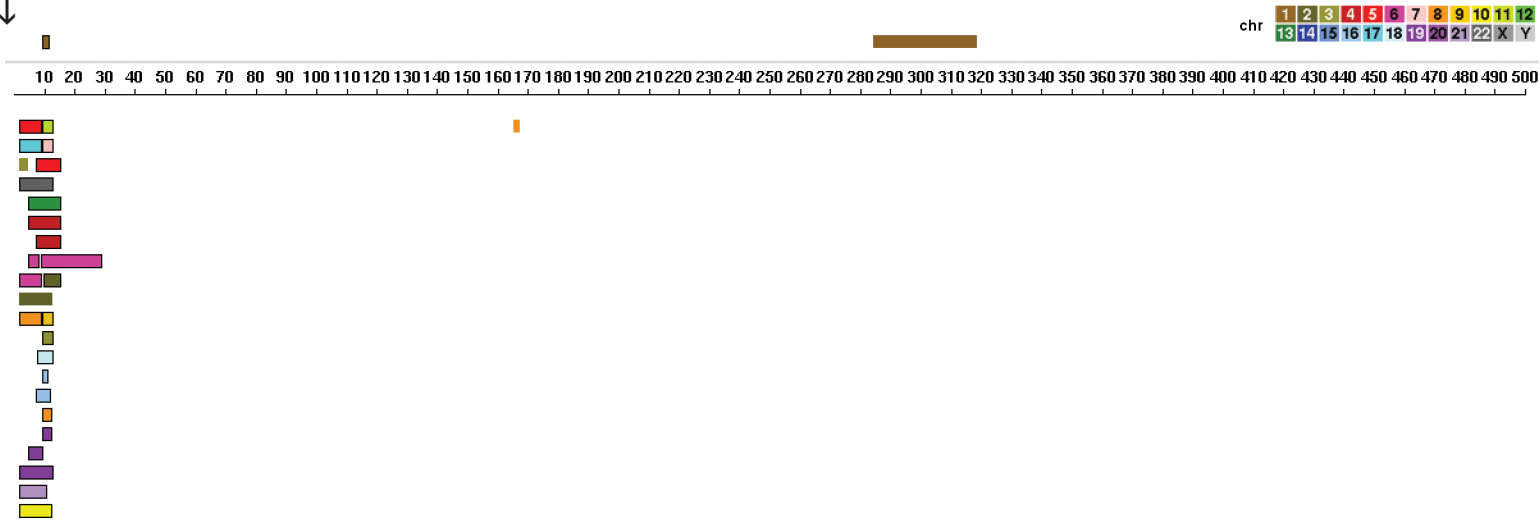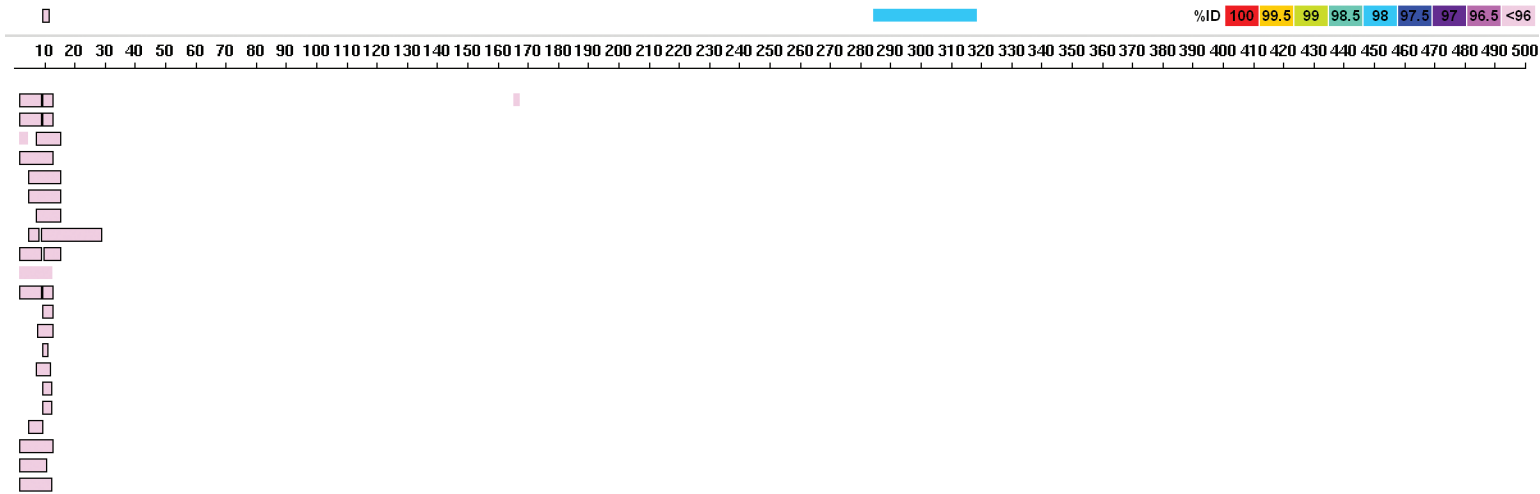

Supplement: Additional data file 7 — The subtelomere sequences shown are the assemblies published previously [6] and are available at the Riethman Lab website [47]. The telomeric end of each sequence assembly is located at the left. The distance from the end of the sequence to the start of the terminal repeat array is indicated by the vertical arrow at the telomeric end of the sequence. The position and orientation of (TTAGGG)n tracts are shown as black arrows. Top panels: duplicated genomic segments are identified by chromosome (color) and whether they are subtelomeric (bounded rectangles), non-subtelomeric (unbounded rectangles), or intra-chromosomal (located above the subtelomere coordinates). Each rectangle represents a separate duplicon. Bottom panels: duplicated genomic segments are the same as in the top panels, but identified by nucleotide sequence similarity with the query subtelomere sequence (color scheme as indicated in the key). [file gb-2007-8-7-r151-S7.pdf]

2p

0 kb Gap

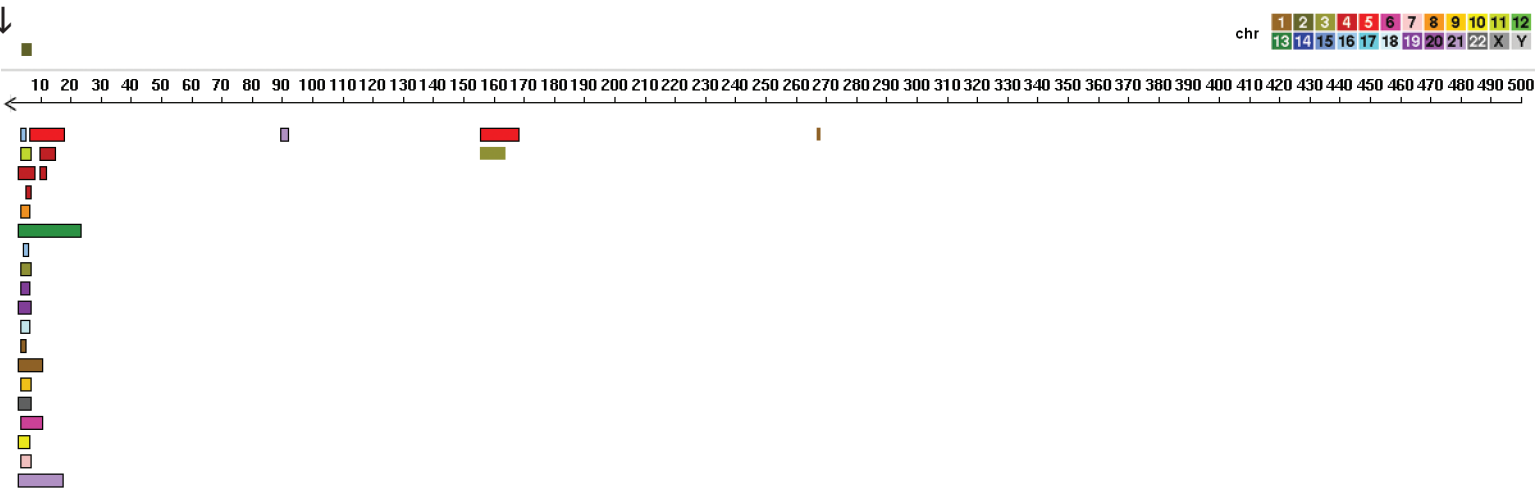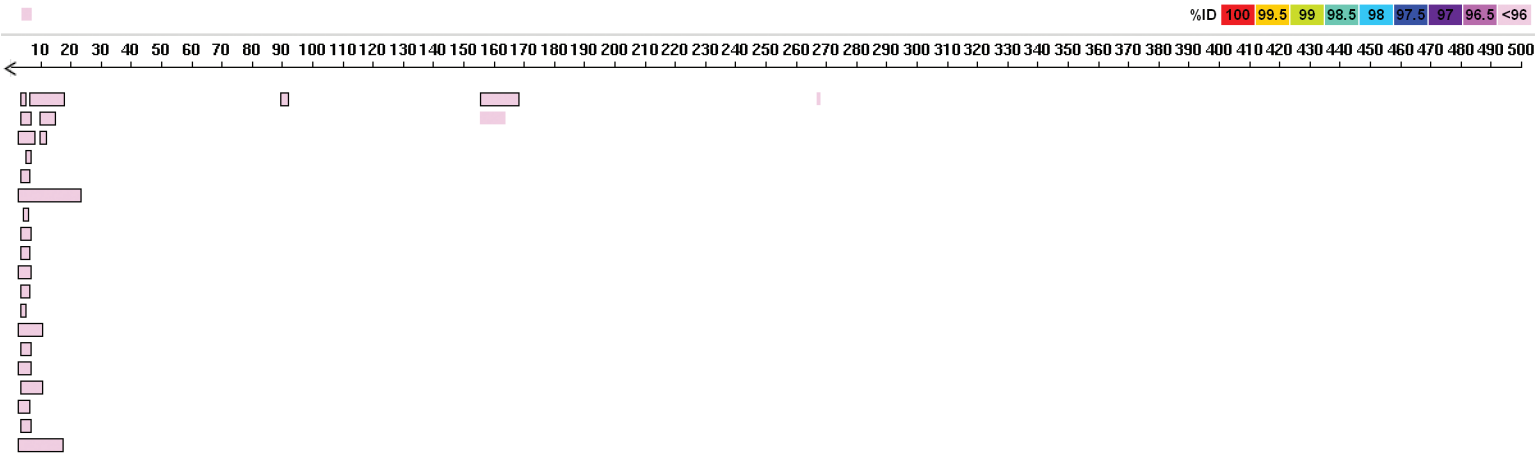

Supplement: Additional data file 8 — The subtelomere sequences shown are the assemblies published previously [6] and are available at the Riethman Lab website [47]. The telomeric end of each sequence assembly is located at the left. The distance from the end of the sequence to the start of the terminal repeat array is indicated by the vertical arrow at the telomeric end of the sequence. The position and orientation of (TTAGGG)n tracts are shown as black arrows. Top panels: duplicated genomic segments are identified by chromosome (color) and whether they are subtelomeric (bounded rectangles), non-subtelomeric (unbounded rectangles), or intra-chromosomal (located above the subtelomere coordinates). Each rectangle represents a separate duplicon. Bottom panels: duplicated genomic segments are the same as in the top panels, but identified by nucleotide sequence similarity with the query subtelomere sequence (color scheme as indicated in the key). [file gb-2007-8-7-r151-S8.pdf]

2q

16 kb Gap

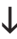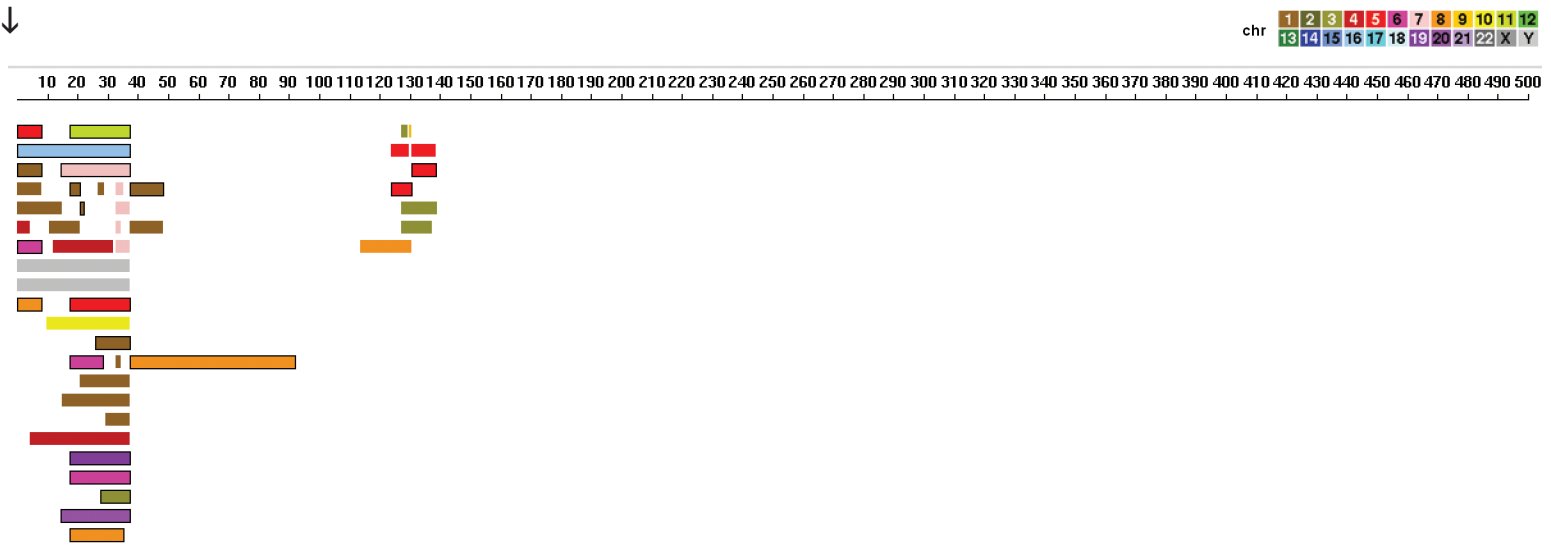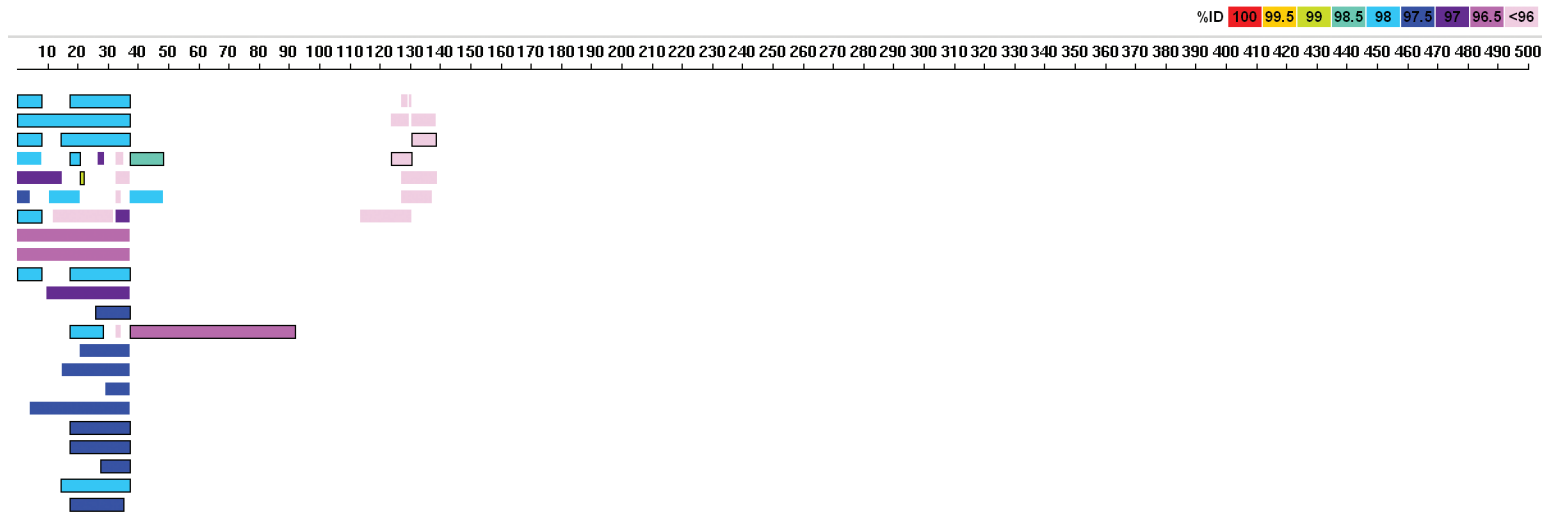

Supplement: Additional data file 9 — The subtelomere sequences shown are the assemblies published previously [6] and are available at the Riethman Lab website [47]. The telomeric end of each sequence assembly is located at the left. The distance from the end of the sequence to the start of the terminal repeat array is indicated by the vertical arrow at the telomeric end of the sequence. The position and orientation of (TTAGGG)n tracts are shown as black arrows. Top panels: duplicated genomic segments are identified by chromosome (color) and whether they are subtelomeric (bounded rectangles), non-subtelomeric (unbounded rectangles), or intra-chromosomal (located above the subtelomere coordinates). Each rectangle represents a separate duplicon. Bottom panels: duplicated genomic segments are the same as in the top panels, but identified by nucleotide sequence similarity with the query subtelomere sequence (color scheme as indicated in the key). [file gb-2007-8-7-r151-S9.pdf]

3p

10 kb Gap

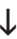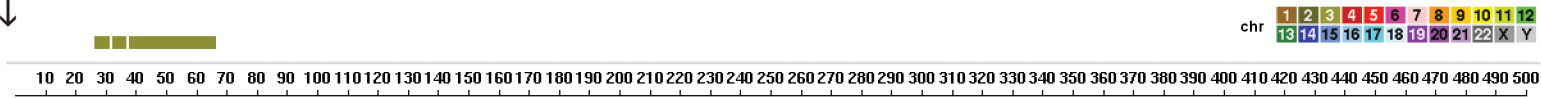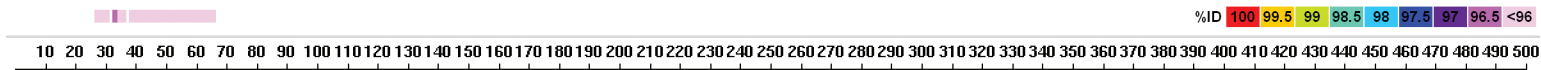

Supplement: Additional data file 10 — The subtelomere sequences shown are the assemblies published previously [6] and are available at the Riethman Lab website [47]. The telomeric end of each sequence assembly is located at the left. The distance from the end of the sequence to the start of the terminal repeat array is indicated by the vertical arrow at the telomeric end of the sequence. The position and orientation of (TTAGGG)n tracts are shown as black arrows. Top panels: duplicated genomic segments are identified by chromosome (color) and whether they are subtelomeric (bounded rectangles), non-subtelomeric (unbounded rectangles), or intra-chromosomal (located above the subtelomere coordinates). Each rectangle represents a separate duplicon. Bottom panels: duplicated genomic segments are the same as in the top panels, but identified by nucleotide sequence similarity with the query subtelomere sequence (color scheme as indicated in the key). [file gb-2007-8-7-r151-S10.pdf]

3q

50 kb Gap

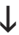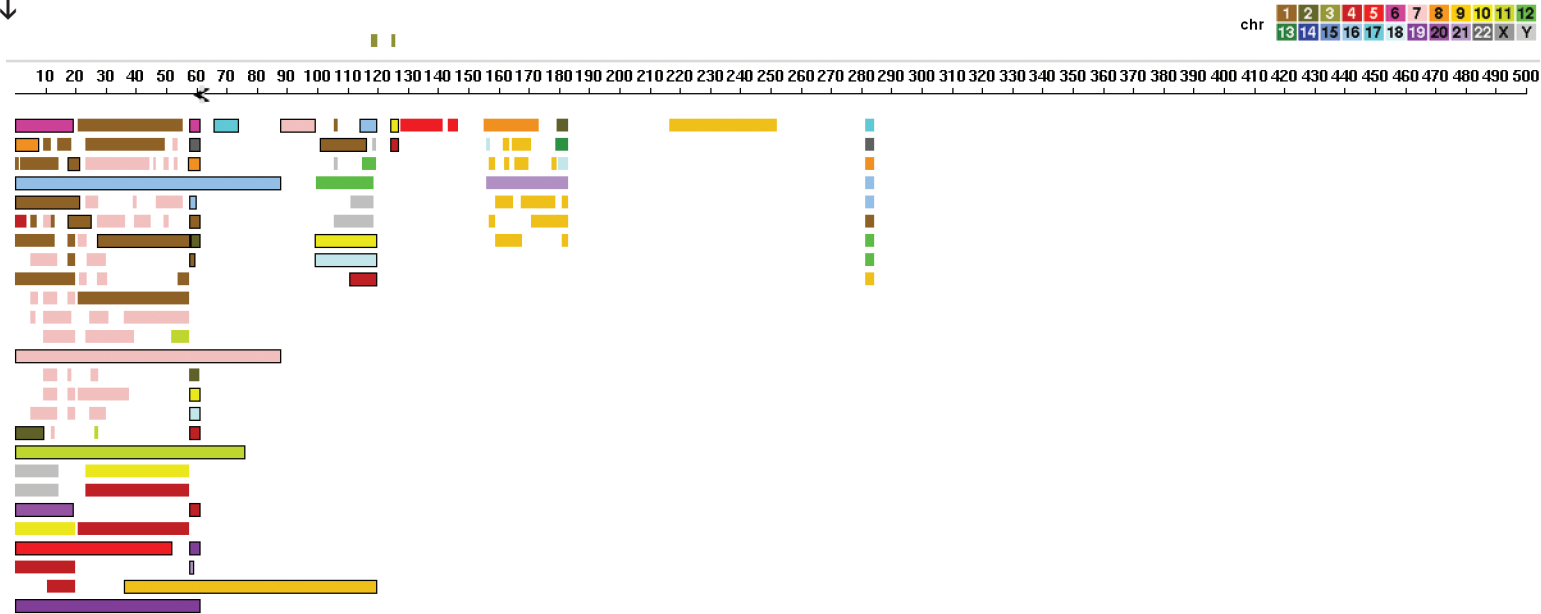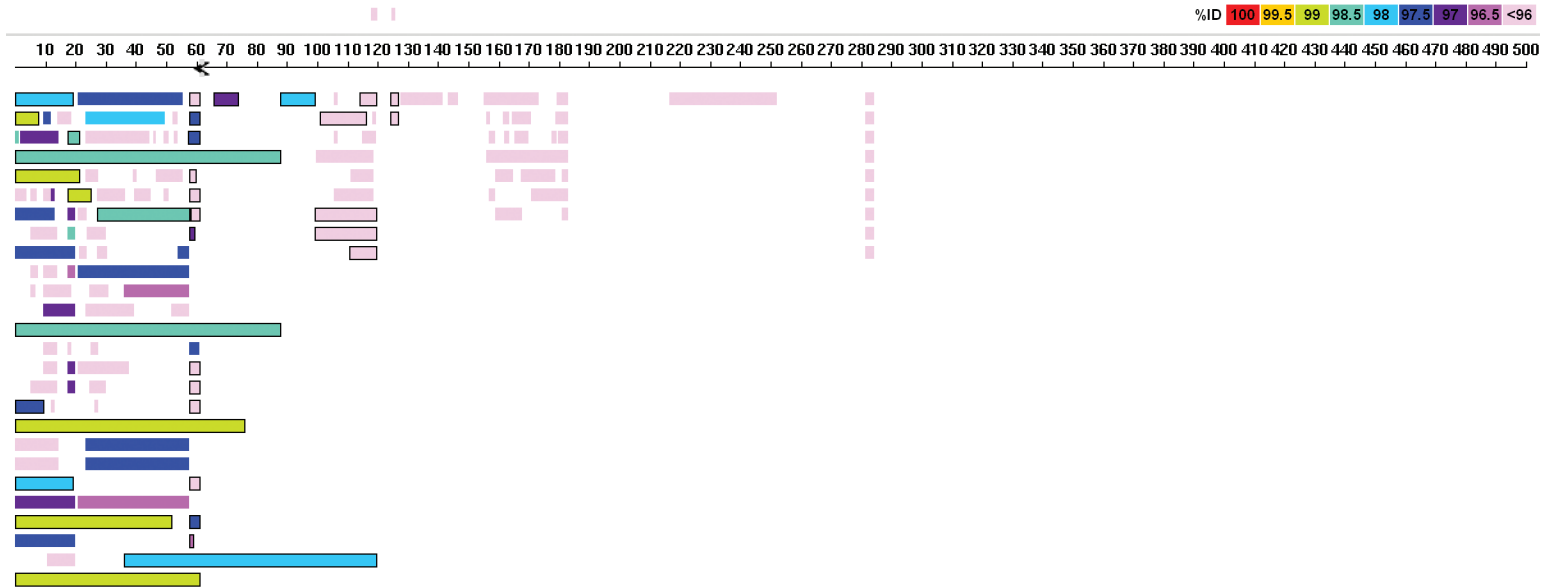

Supplement: Additional data file 11 — The subtelomere sequences shown are the assemblies published previously [6] and are available at the Riethman Lab website [47]. The telomeric end of each sequence assembly is located at the left. The distance from the end of the sequence to the start of the terminal repeat array is indicated by the vertical arrow at the telomeric end of the sequence. The position and orientation of (TTAGGG)n tracts are shown as black arrows. Top panels: duplicated genomic segments are identified by chromosome (color) and whether they are subtelomeric (bounded rectangles), non-subtelomeric (unbounded rectangles), or intra-chromosomal (located above the subtelomere coordinates). Each rectangle represents a separate duplicon. Bottom panels: duplicated genomic segments are the same as in the top panels, but identified by nucleotide sequence similarity with the query subtelomere sequence (color scheme as indicated in the key). [file gb-2007-8-7-r151-S11.pdf]

4p

0 kb Gap

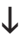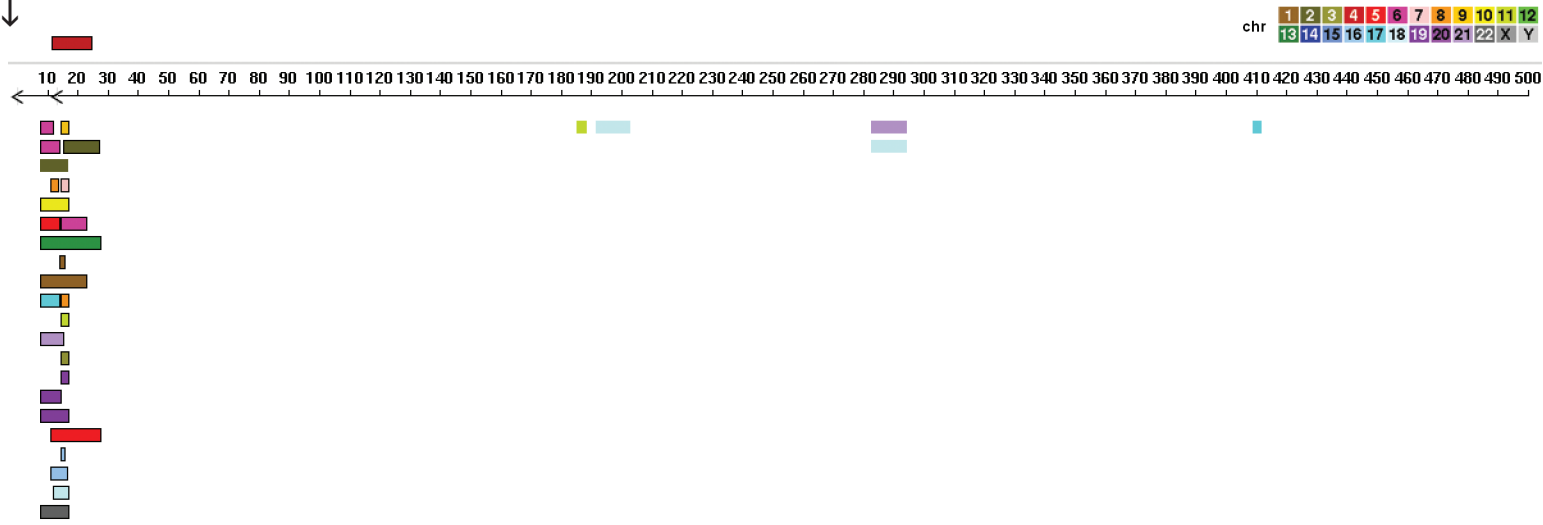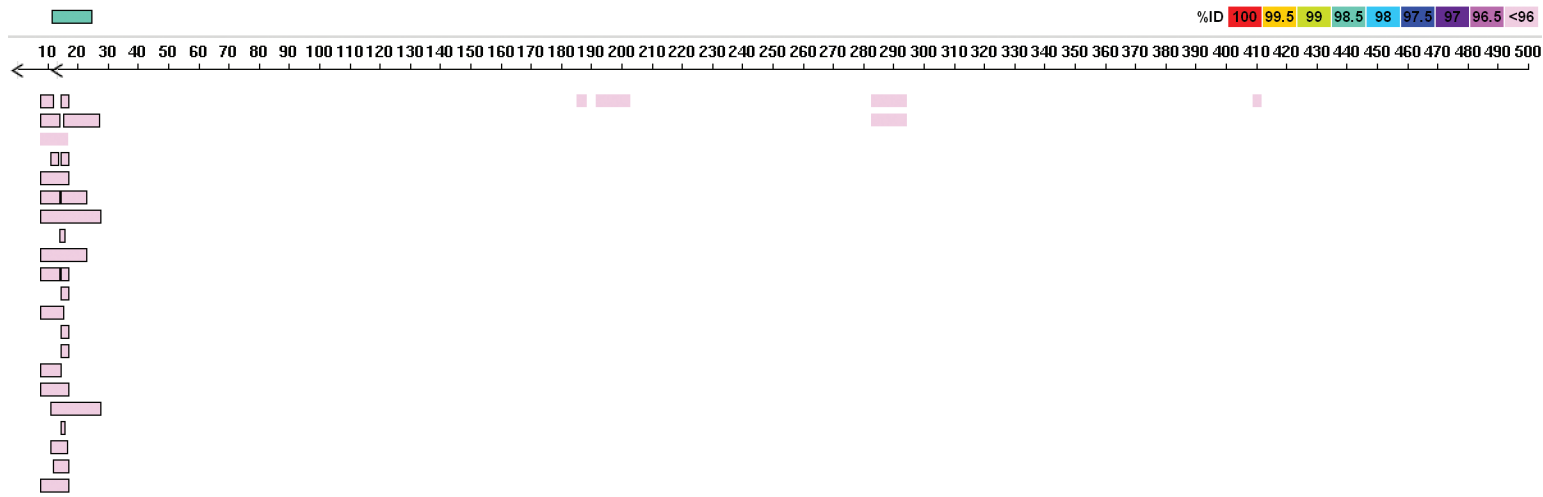

Supplement: Additional data file 12 — The subtelomere sequences shown are the assemblies published previously [6] and are available at the Riethman Lab website [47]. The telomeric end of each sequence assembly is located at the left. The distance from the end of the sequence to the start of the terminal repeat array is indicated by the vertical arrow at the telomeric end of the sequence. The position and orientation of (TTAGGG)n tracts are shown as black arrows. Top panels: duplicated genomic segments are identified by chromosome (color) and whether they are subtelomeric (bounded rectangles), non-subtelomeric (unbounded rectangles), or intra-chromosomal (located above the subtelomere coordinates). Each rectangle represents a separate duplicon. Bottom panels: duplicated genomic segments are the same as in the top panels, but identified by nucleotide sequence similarity with the query subtelomere sequence (color scheme as indicated in the key). [file gb-2007-8-7-r151-S12.pdf]

4q

10 kb Gap

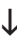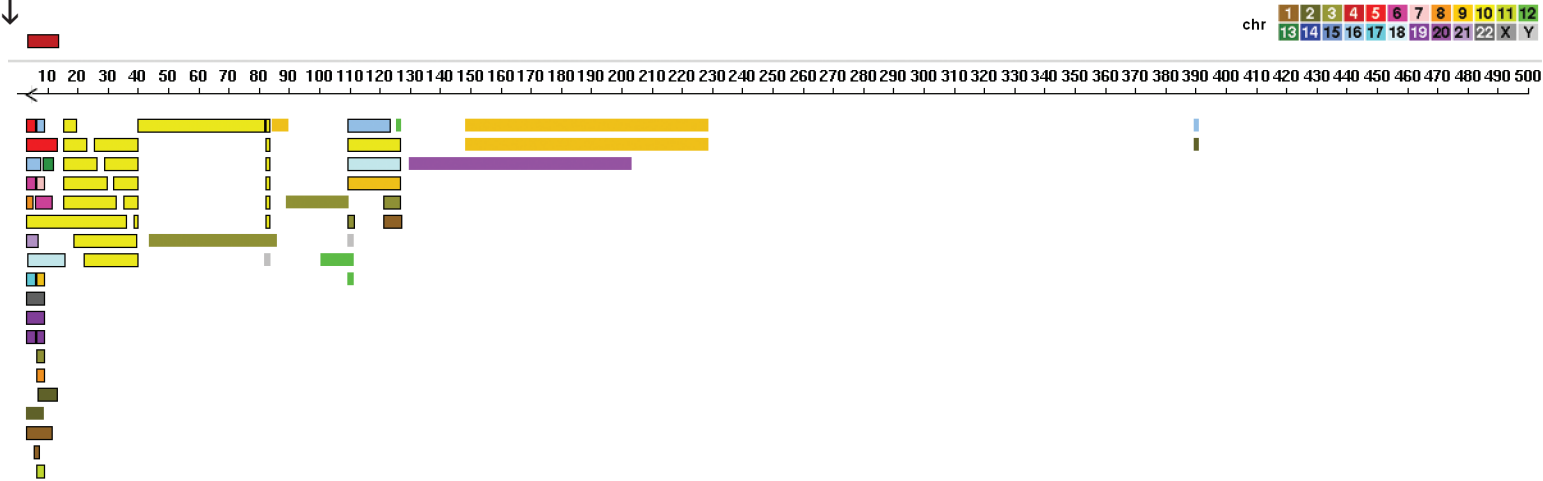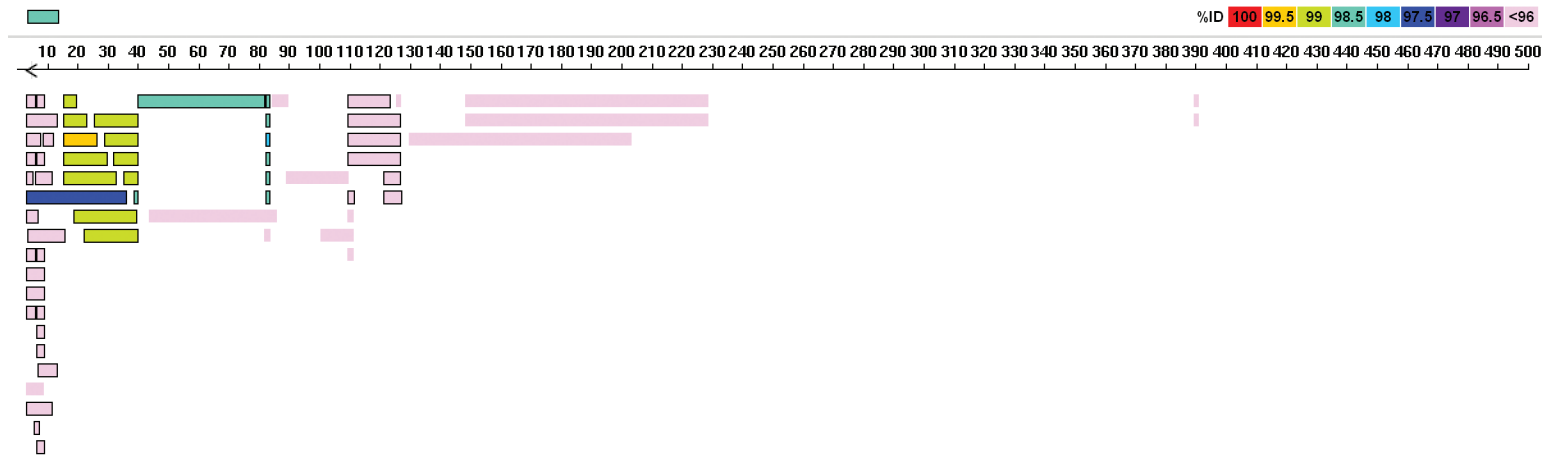

Supplement: Additional data file 13 — The subtelomere sequences shown are the assemblies published previously [6] and are available at the Riethman Lab website [47]. The telomeric end of each sequence assembly is located at the left. The distance from the end of the sequence to the start of the terminal repeat array is indicated by the vertical arrow at the telomeric end of the sequence. The position and orientation of (TTAGGG)n tracts are shown as black arrows. Top panels: duplicated genomic segments are identified by chromosome (color) and whether they are subtelomeric (bounded rectangles), non-subtelomeric (unbounded rectangles), or intra-chromosomal (located above the subtelomere coordinates). Each rectangle represents a separate duplicon. Bottom panels: duplicated genomic segments are the same as in the top panels, but identified by nucleotide sequence similarity with the query subtelomere sequence (color scheme as indicated in the key). [file gb-2007-8-7-r151-S13.pdf]

5p

70 kb Gap

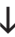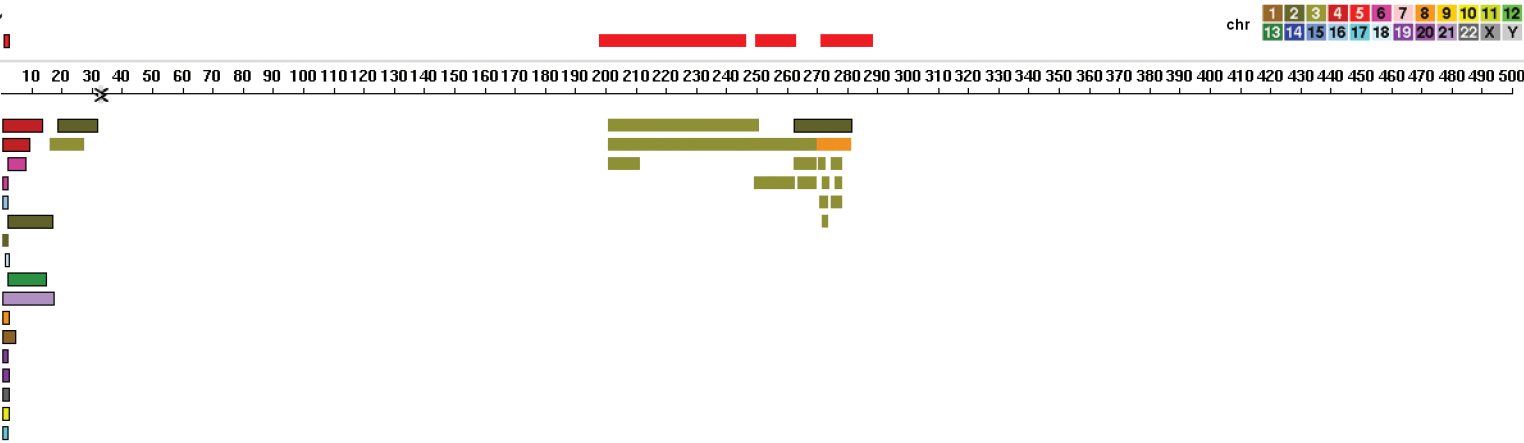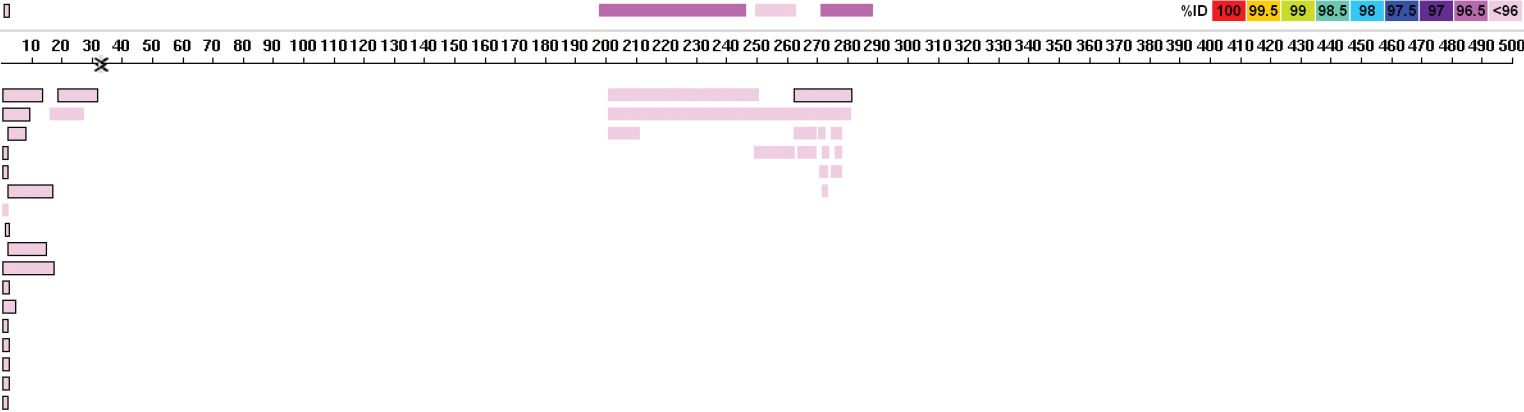

Supplement: Additional data file 14 — The subtelomere sequences shown are the assemblies published previously [6] and are available at the Riethman Lab website [47]. The telomeric end of each sequence assembly is located at the left. The distance from the end of the sequence to the start of the terminal repeat array is indicated by the vertical arrow at the telomeric end of the sequence. The position and orientation of (TTAGGG)n tracts are shown as black arrows. Top panels: duplicated genomic segments are identified by chromosome (color) and whether they are subtelomeric (bounded rectangles), non-subtelomeric (unbounded rectangles), or intra-chromosomal (located above the subtelomere coordinates). Each rectangle represents a separate duplicon. Bottom panels: duplicated genomic segments are the same as in the top panels, but identified by nucleotide sequence similarity with the query subtelomere sequence (color scheme as indicated in the key). [file gb-2007-8-7-r151-S14.pdf]

5q

20 kb Gap

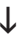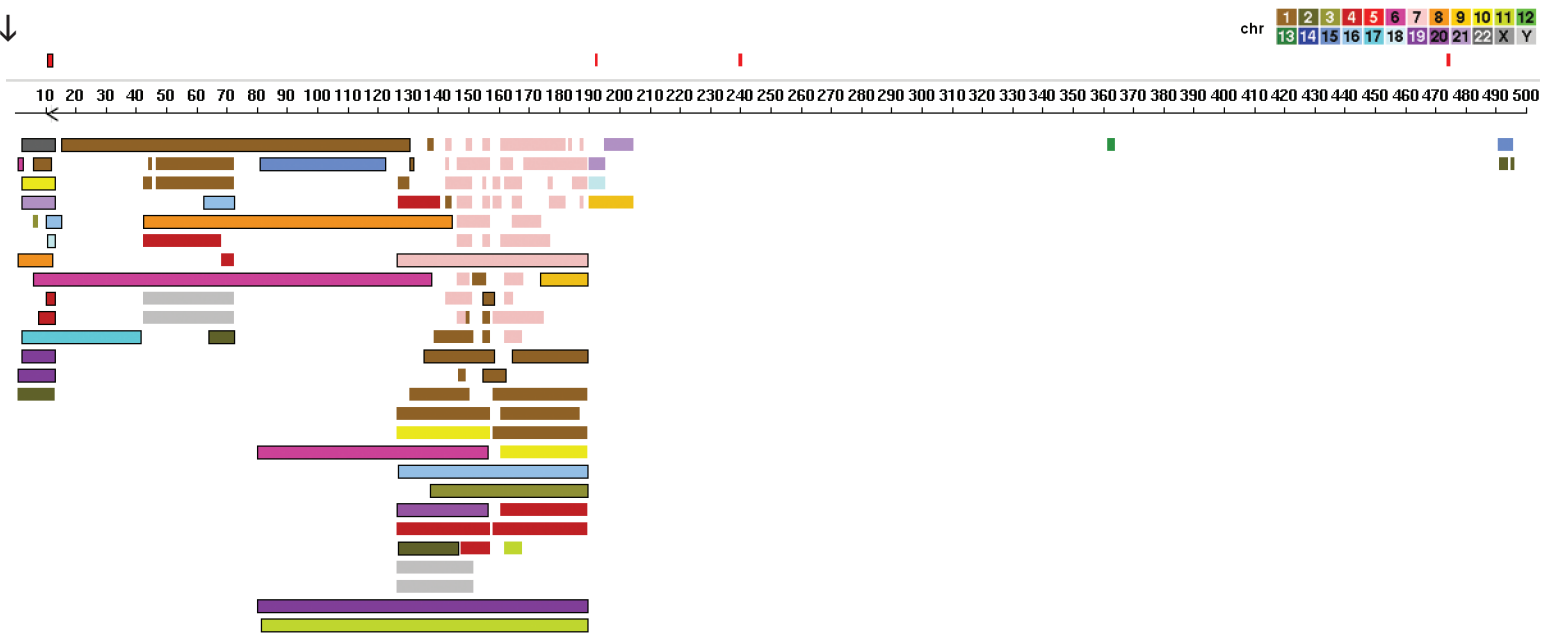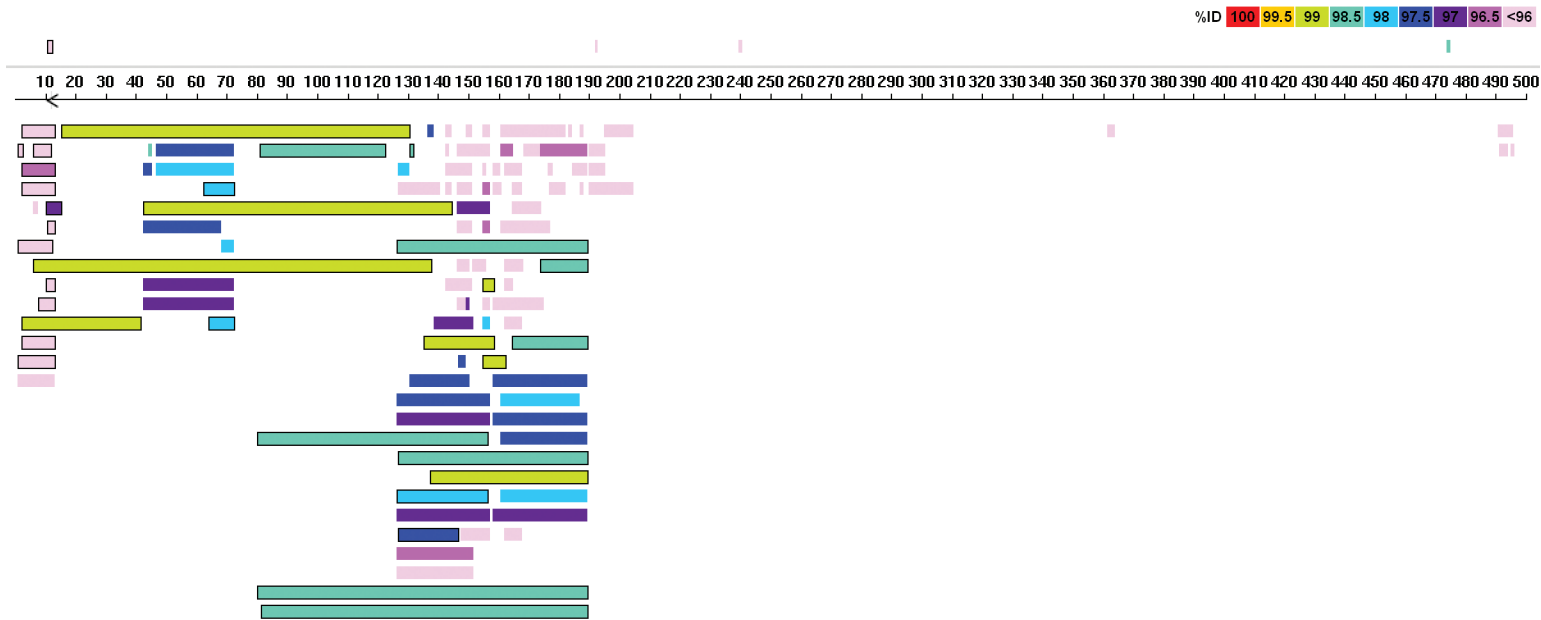

Supplement: Additional data file 15 — The subtelomere sequences shown are the assemblies published previously [6] and are available at the Riethman Lab website [47]. The telomeric end of each sequence assembly is located at the left. The distance from the end of the sequence to the start of the terminal repeat array is indicated by the vertical arrow at the telomeric end of the sequence. The position and orientation of (TTAGGG)n tracts are shown as black arrows. Top panels: duplicated genomic segments are identified by chromosome (color) and whether they are subtelomeric (bounded rectangles), non-subtelomeric (unbounded rectangles), or intra-chromosomal (located above the subtelomere coordinates). Each rectangle represents a separate duplicon. Bottom panels: duplicated genomic segments are the same as in the top panels, but identified by nucleotide sequence similarity with the query subtelomere sequence (color scheme as indicated in the key). [file gb-2007-8-7-r151-S15.pdf]

6p

5 kb Gap

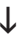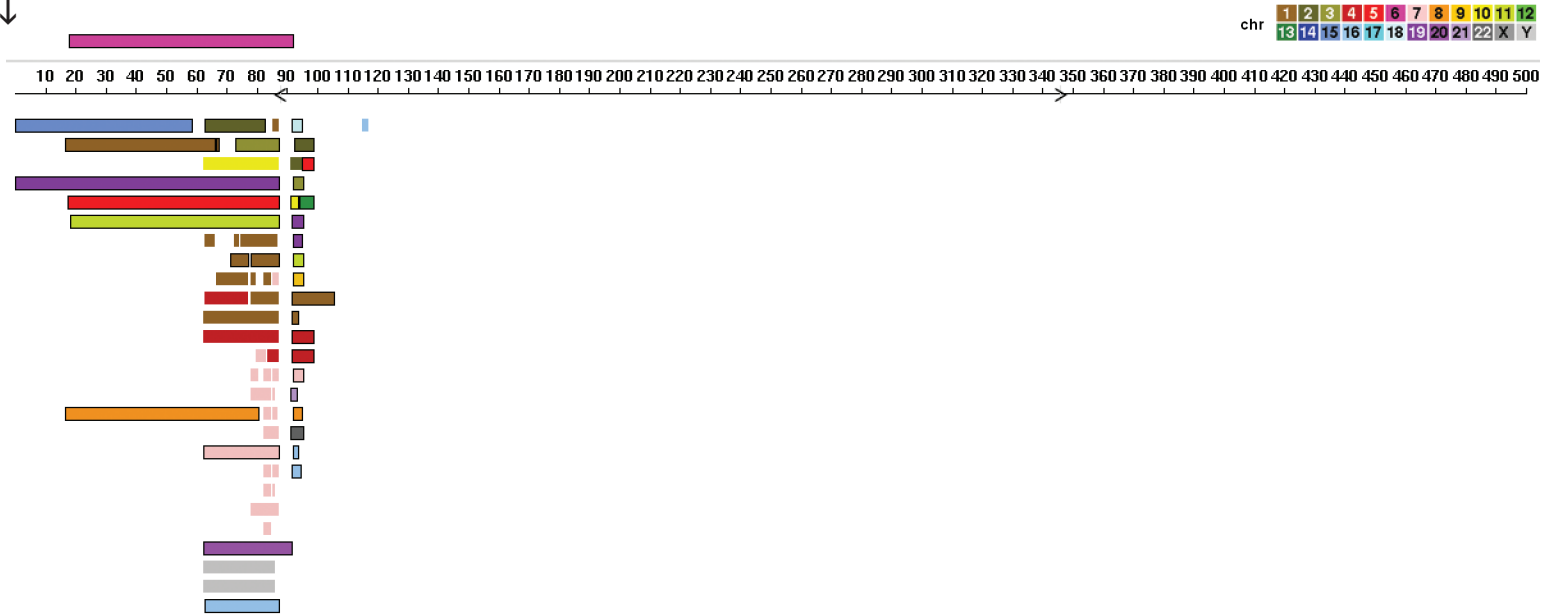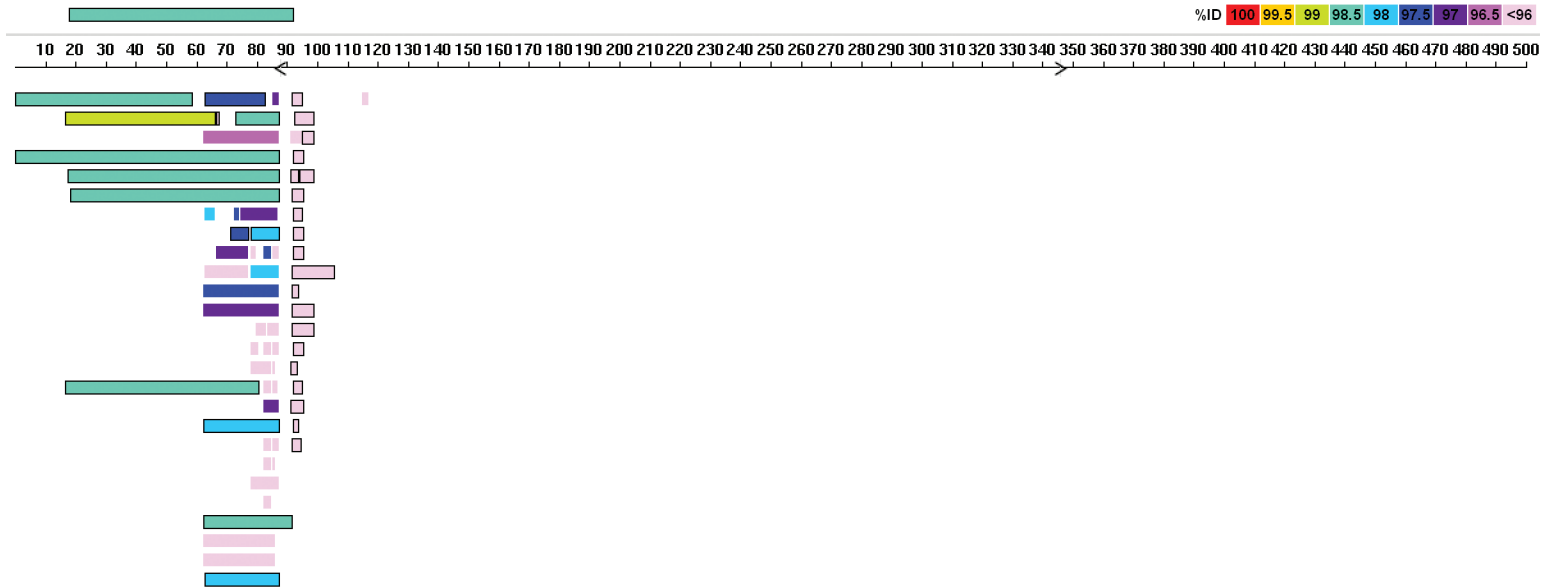

Supplement: Additional data file 16 — The subtelomere sequences shown are the assemblies published previously [6] and are available at the Riethman Lab website [47]. The telomeric end of each sequence assembly is located at the left. The distance from the end of the sequence to the start of the terminal repeat array is indicated by the vertical arrow at the telomeric end of the sequence. The position and orientation of (TTAGGG)n tracts are shown as black arrows. Top panels: duplicated genomic segments are identified by chromosome (color) and whether they are subtelomeric (bounded rectangles), non-subtelomeric (unbounded rectangles), or intra-chromosomal (located above the subtelomere coordinates). Each rectangle represents a separate duplicon. Bottom panels: duplicated genomic segments are the same as in the top panels, but identified by nucleotide sequence similarity with the query subtelomere sequence (color scheme as indicated in the key). [file gb-2007-8-7-r151-S16.pdf]

6q

3 kb Gap

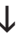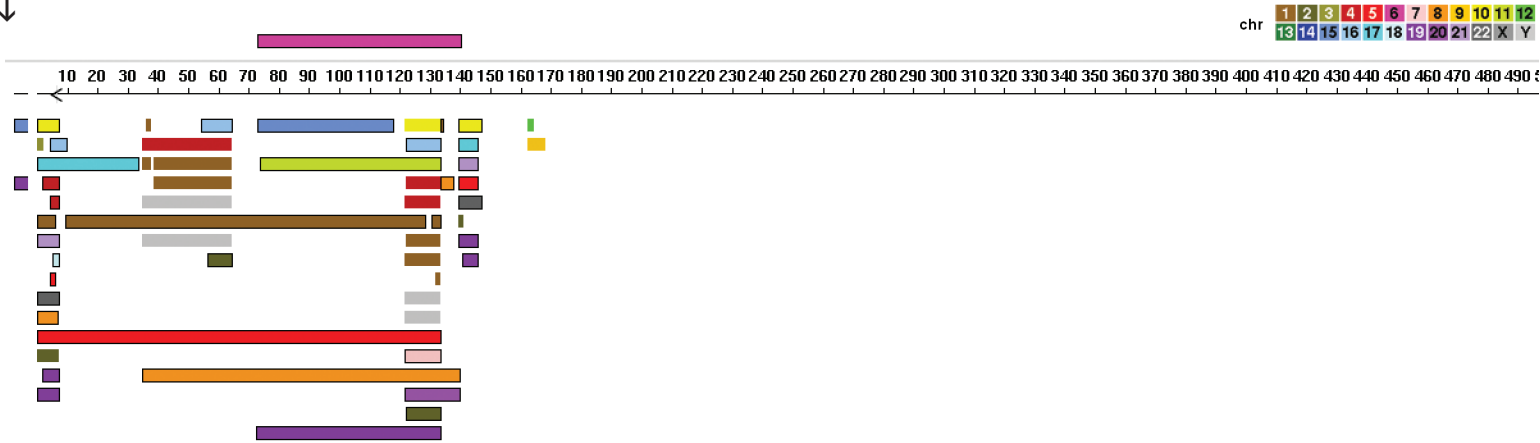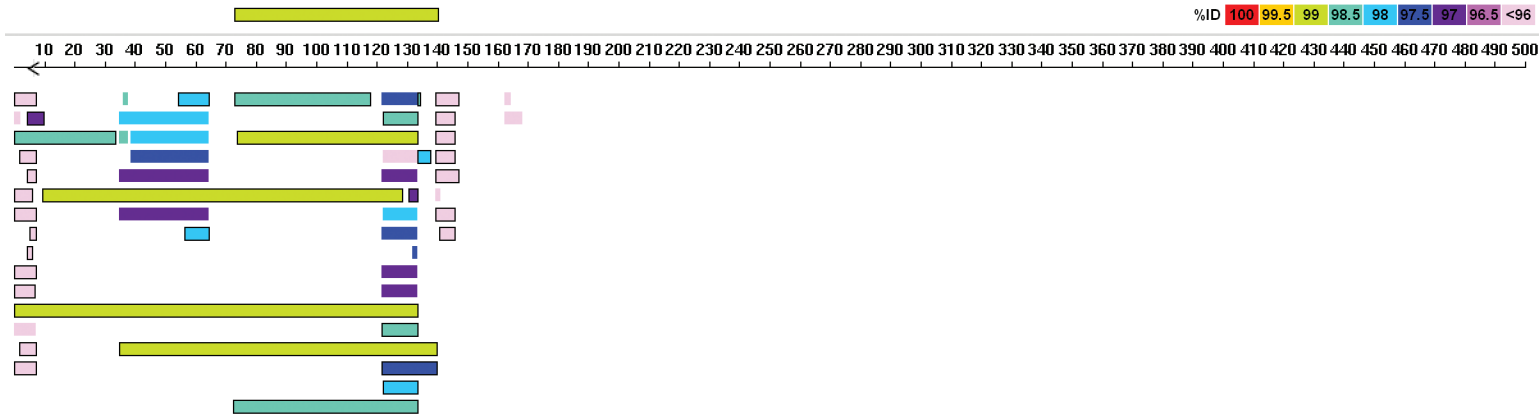

Supplement: Additional data file 17 — The subtelomere sequences shown are the assemblies published previously [6] and are available at the Riethman Lab website [47]. The telomeric end of each sequence assembly is located at the left. The distance from the end of the sequence to the start of the terminal repeat array is indicated by the vertical arrow at the telomeric end of the sequence. The position and orientation of (TTAGGG)n tracts are shown as black arrows. Top panels: duplicated genomic segments are identified by chromosome (color) and whether they are subtelomeric (bounded rectangles), non-subtelomeric (unbounded rectangles), or intra-chromosomal (located above the subtelomere coordinates). Each rectangle represents a separate duplicon. Bottom panels: duplicated genomic segments are the same as in the top panels, but identified by nucleotide sequence similarity with the query subtelomere sequence (color scheme as indicated in the key). [file gb-2007-8-7-r151-S17.pdf]

7p

34 kb Gap

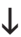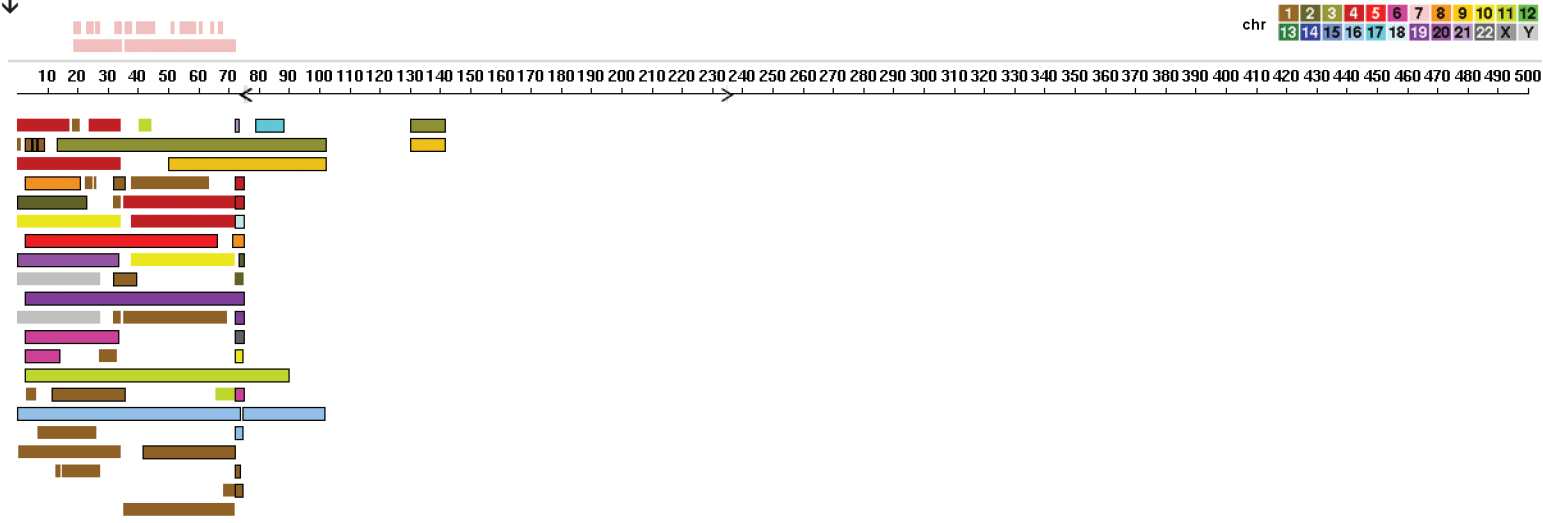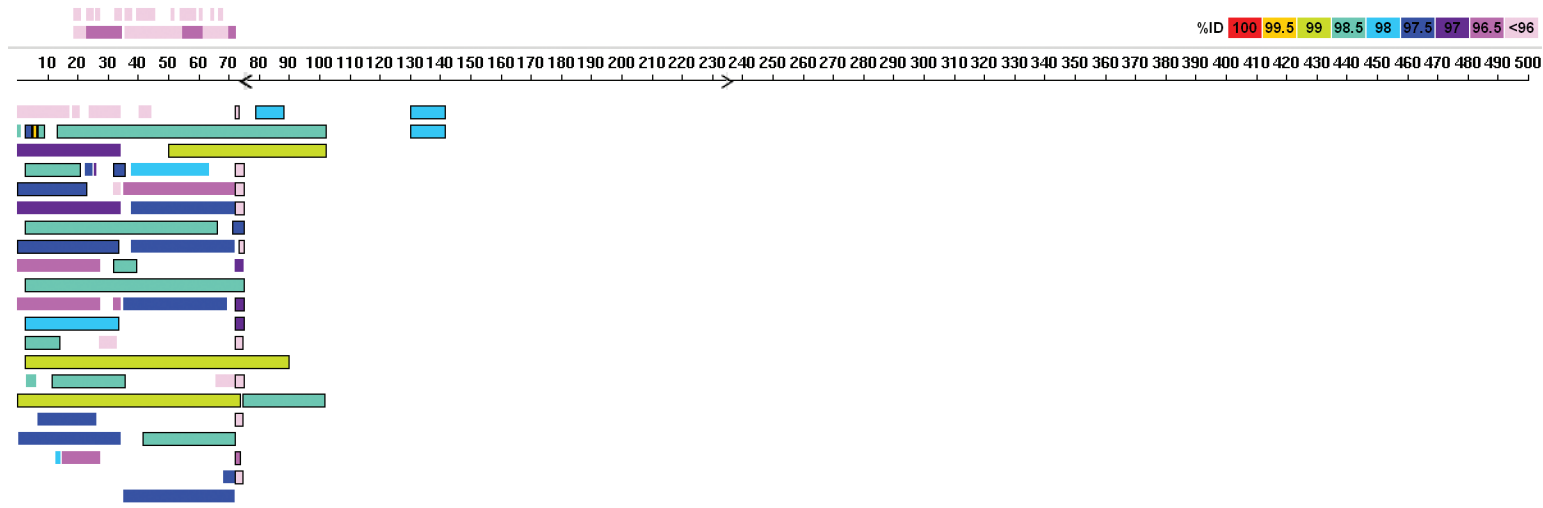

Supplement: Additional data file 18 — The subtelomere sequences shown are the assemblies published previously [6] and are available at the Riethman Lab website [47]. The telomeric end of each sequence assembly is located at the left. The distance from the end of the sequence to the start of the terminal repeat array is indicated by the vertical arrow at the telomeric end of the sequence. The position and orientation of (TTAGGG)n tracts are shown as black arrows. Top panels: duplicated genomic segments are identified by chromosome (color) and whether they are subtelomeric (bounded rectangles), non-subtelomeric (unbounded rectangles), or intra-chromosomal (located above the subtelomere coordinates). Each rectangle represents a separate duplicon. Bottom panels: duplicated genomic segments are the same as in the top panels, but identified by nucleotide sequence similarity with the query subtelomere sequence (color scheme as indicated in the key). [file gb-2007-8-7-r151-S18.pdf]

7q

0 kb Gap

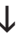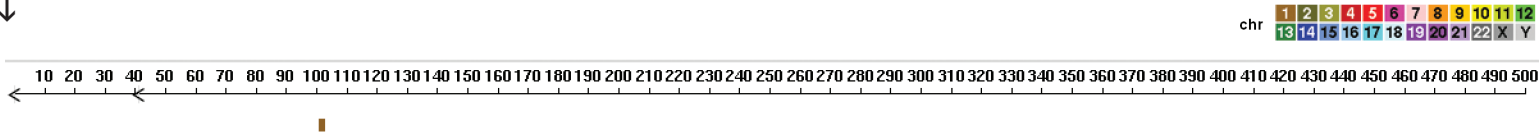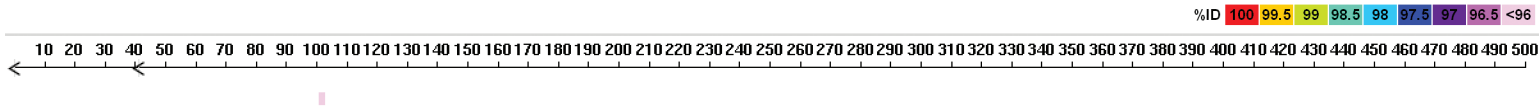

Supplement: Additional data file 19 — The subtelomere sequences shown are the assemblies published previously [6] and are available at the Riethman Lab website [47]. The telomeric end of each sequence assembly is located at the left. The distance from the end of the sequence to the start of the terminal repeat array is indicated by the vertical arrow at the telomeric end of the sequence. The position and orientation of (TTAGGG)n tracts are shown as black arrows. Top panels: duplicated genomic segments are identified by chromosome (color) and whether they are subtelomeric (bounded rectangles), non-subtelomeric (unbounded rectangles), or intra-chromosomal (located above the subtelomere coordinates). Each rectangle represents a separate duplicon. Bottom panels: duplicated genomic segments are the same as in the top panels, but identified by nucleotide sequence similarity with the query subtelomere sequence (color scheme as indicated in the key). [file gb-2007-8-7-r151-S19.pdf]

8p

89 kb Gap

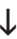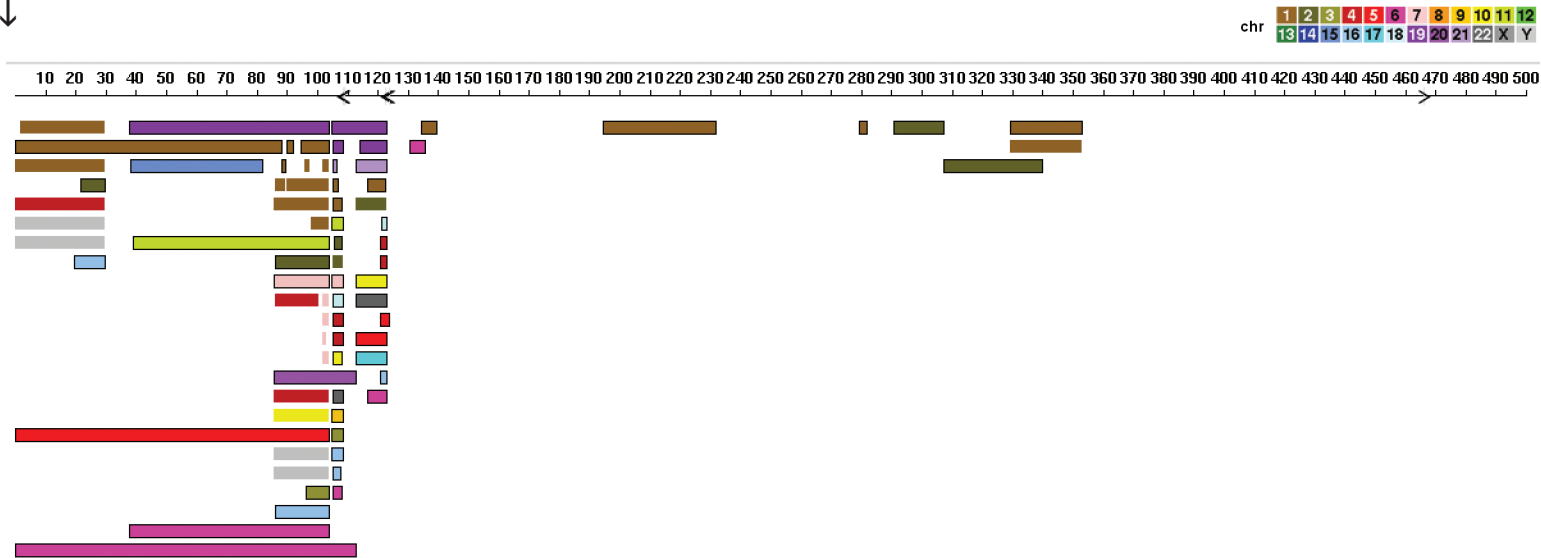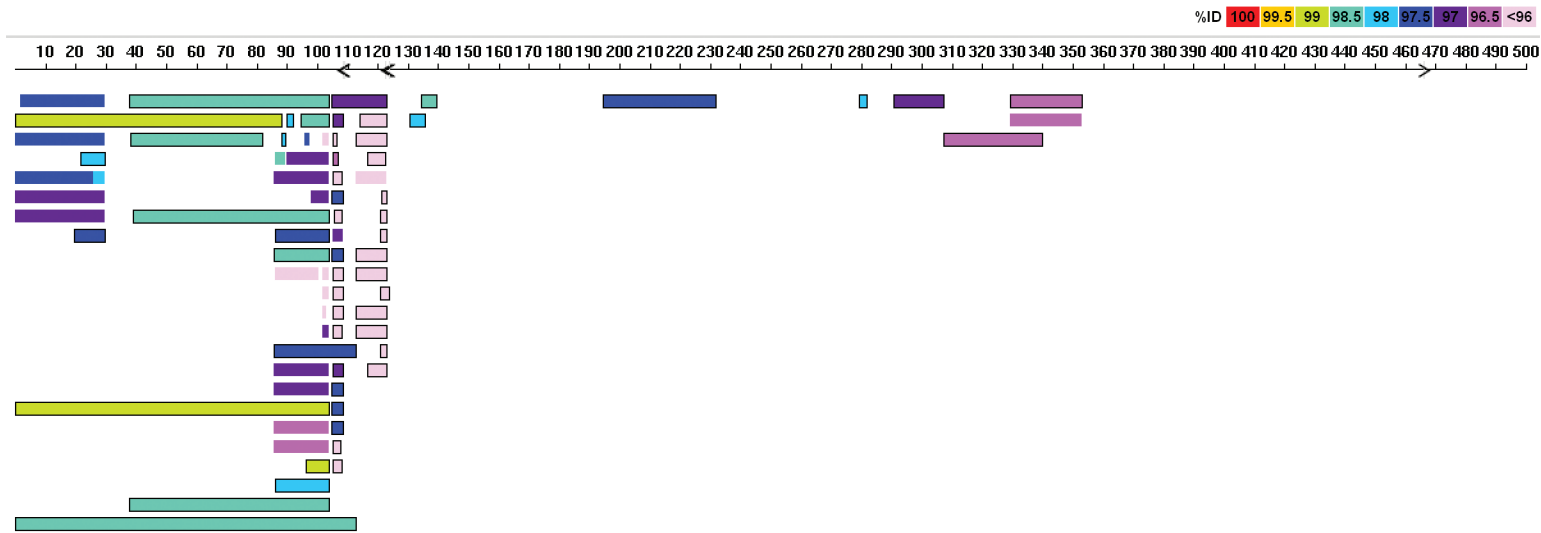

Supplement: Additional data file 20 — The subtelomere sequences shown are the assemblies published previously [6] and are available at the Riethman Lab website [47]. The telomeric end of each sequence assembly is located at the left. The distance from the end of the sequence to the start of the terminal repeat array is indicated by the vertical arrow at the telomeric end of the sequence. The position and orientation of (TTAGGG)n tracts are shown as black arrows. Top panels: duplicated genomic segments are identified by chromosome (color) and whether they are subtelomeric (bounded rectangles), non-subtelomeric (unbounded rectangles), or intra-chromosomal (located above the subtelomere coordinates). Each rectangle represents a separate duplicon. Bottom panels: duplicated genomic segments are the same as in the top panels, but identified by nucleotide sequence similarity with the query subtelomere sequence (color scheme as indicated in the key). [file gb-2007-8-7-r151-S20.pdf]

8q

0 kb Gap  
↓

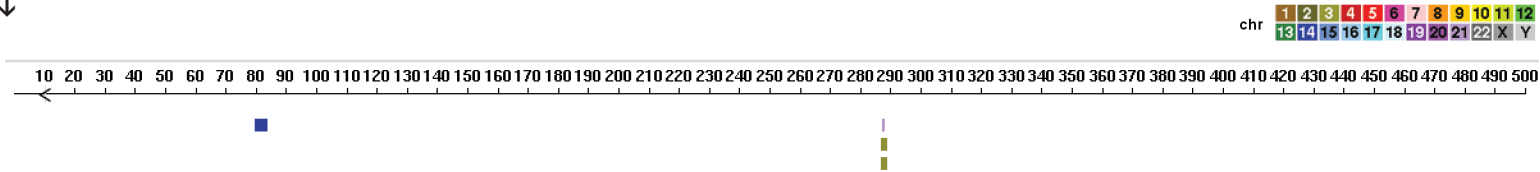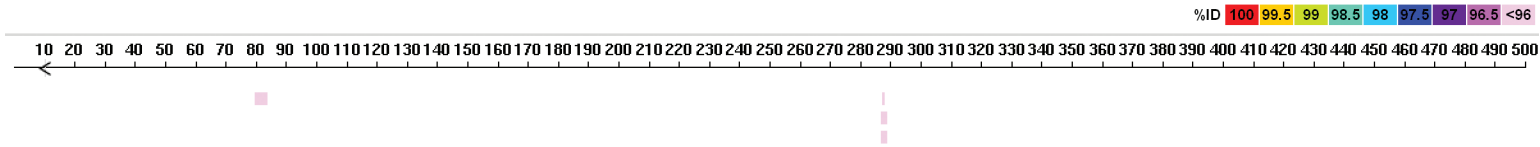

Supplement: Additional data file 21 — The subtelomere sequences shown are the assemblies published previously [6] and are available at the Riethman Lab website [47]. The telomeric end of each sequence assembly is located at the left. The distance from the end of the sequence to the start of the terminal repeat array is indicated by the vertical arrow at the telomeric end of the sequence. The position and orientation of (TTAGGG)n tracts are shown as black arrows. Top panels: duplicated genomic segments are identified by chromosome (color) and whether they are subtelomeric (bounded rectangles), non-subtelomeric (unbounded rectangles), or intra-chromosomal (located above the subtelomere coordinates). Each rectangle represents a separate duplicon. Bottom panels: duplicated genomic segments are the same as in the top panels, but identified by nucleotide sequence similarity with the query subtelomere sequence (color scheme as indicated in the key). [file gb-2007-8-7-r151-S21.pdf]

9p

0 kb Gap  
↓

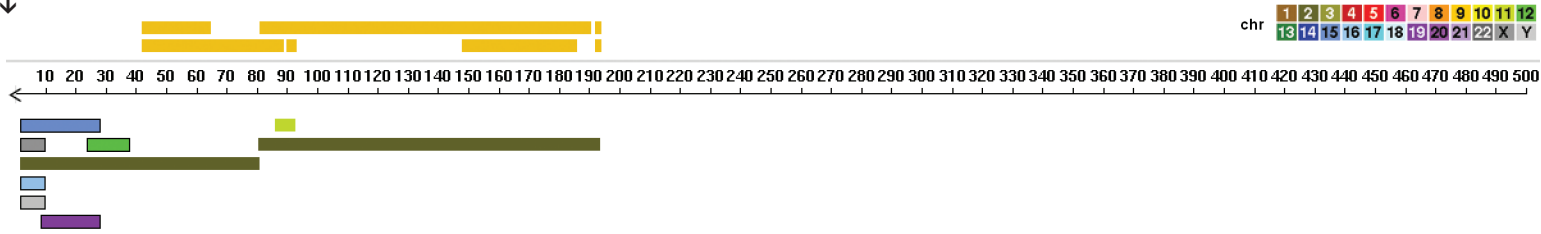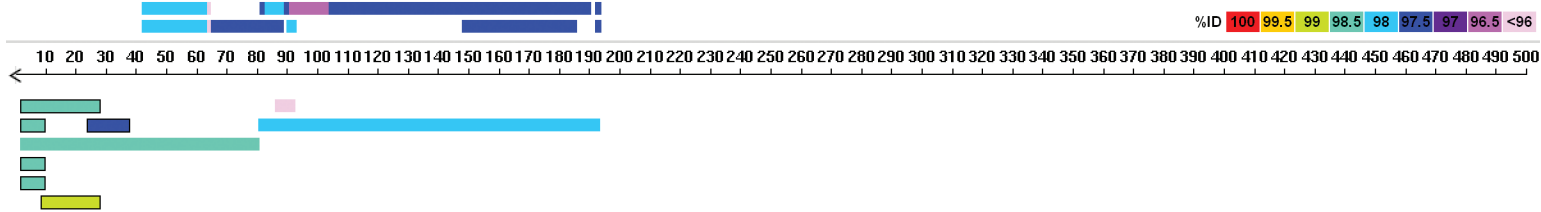

Supplement: Additional data file 22 — The subtelomere sequences shown are the assemblies published previously [6] and are available at the Riethman Lab website [47]. The telomeric end of each sequence assembly is located at the left. The distance from the end of the sequence to the start of the terminal repeat array is indicated by the vertical arrow at the telomeric end of the sequence. The position and orientation of (TTAGGG)n tracts are shown as black arrows. Top panels: duplicated genomic segments are identified by chromosome (color) and whether they are subtelomeric (bounded rectangles), non-subtelomeric (unbounded rectangles), or intra-chromosomal (located above the subtelomere coordinates). Each rectangle represents a separate duplicon. Bottom panels: duplicated genomic segments are the same as in the top panels, but identified by nucleotide sequence similarity with the query subtelomere sequence (color scheme as indicated in the key). [file gb-2007-8-7-r151-S22.pdf]

9q

50 kb Gap

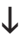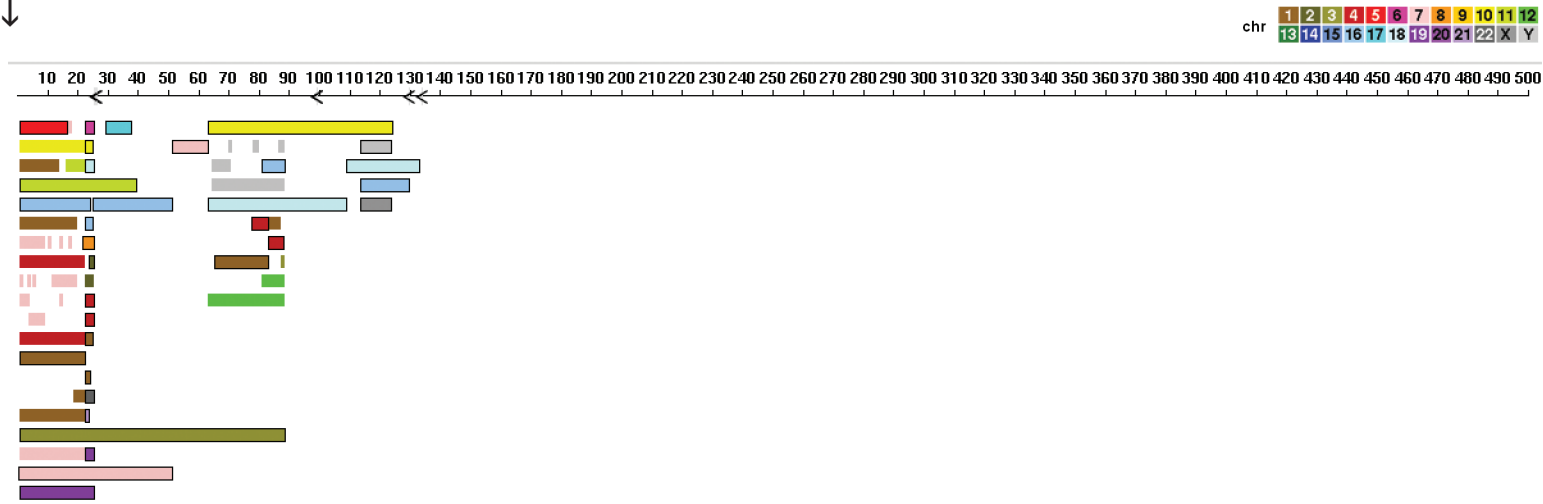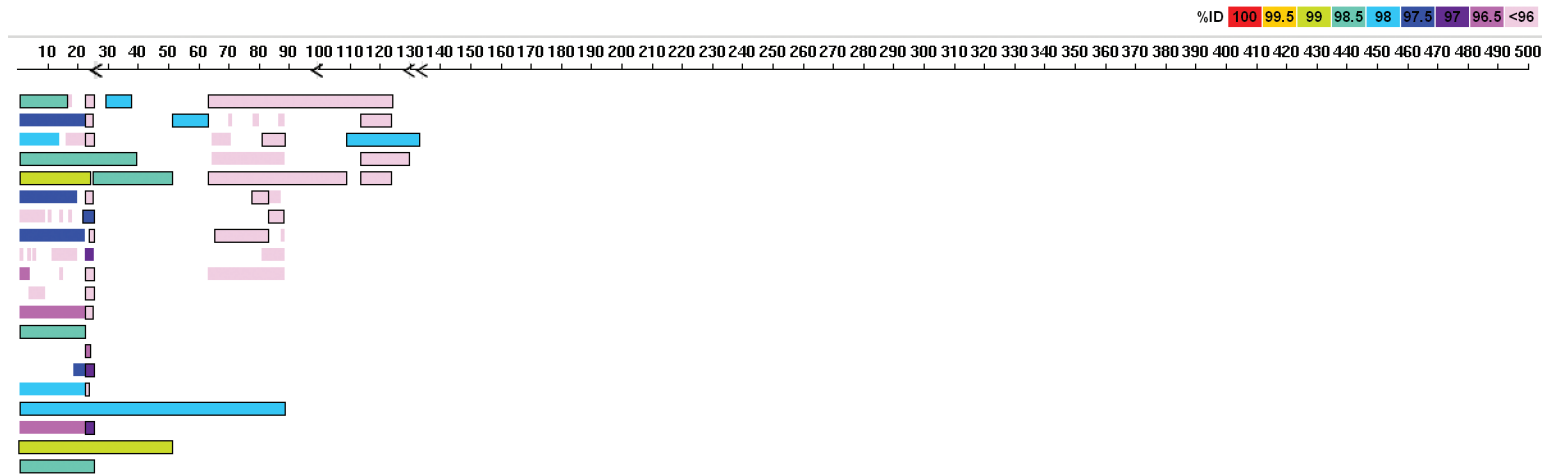

Supplement: Additional data file 23 — The subtelomere sequences shown are the assemblies published previously [6] and are available at the Riethman Lab website [47]. The telomeric end of each sequence assembly is located at the left. The distance from the end of the sequence to the start of the terminal repeat array is indicated by the vertical arrow at the telomeric end of the sequence. The position and orientation of (TTAGGG)n tracts are shown as black arrows. Top panels: duplicated genomic segments are identified by chromosome (color) and whether they are subtelomeric (bounded rectangles), non-subtelomeric (unbounded rectangles), or intra-chromosomal (located above the subtelomere coordinates). Each rectangle represents a separate duplicon. Bottom panels: duplicated genomic segments are the same as in the top panels, but identified by nucleotide sequence similarity with the query subtelomere sequence (color scheme as indicated in the key). [file gb-2007-8-7-r151-S23.pdf]

10p

14 kb Gap

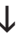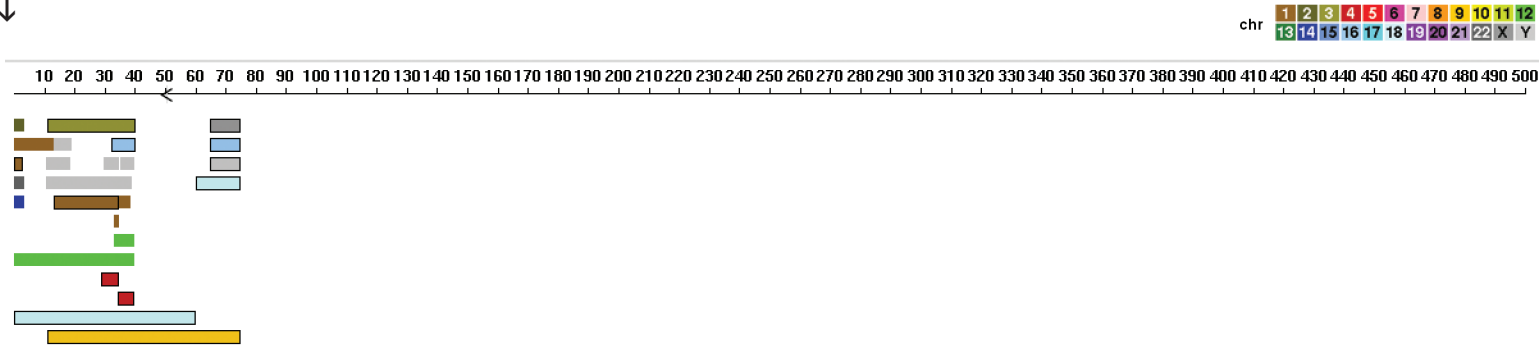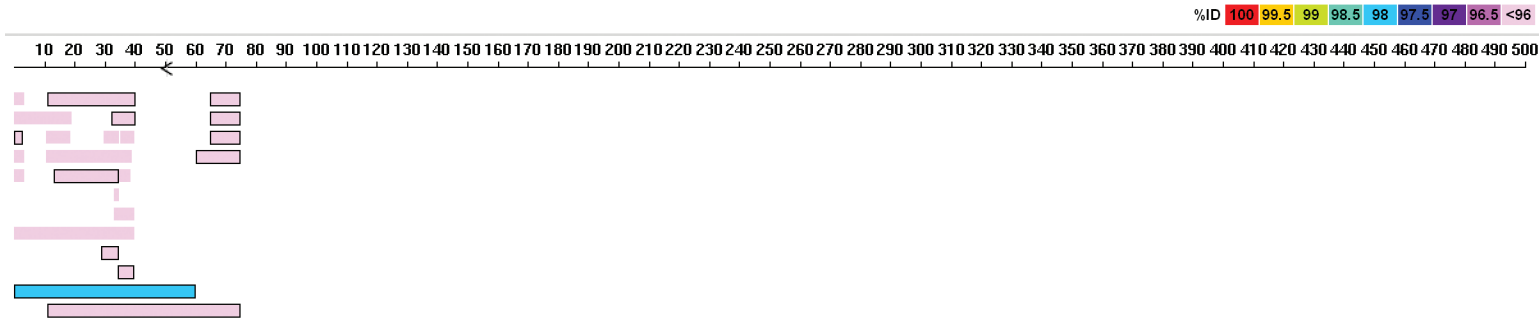

Supplement: Additional data file 24 — The subtelomere sequences shown are the assemblies published previously [6] and are available at the Riethman Lab website [47]. The telomeric end of each sequence assembly is located at the left. The distance from the end of the sequence to the start of the terminal repeat array is indicated by the vertical arrow at the telomeric end of the sequence. The position and orientation of (TTAGGG)n tracts are shown as black arrows. Top panels: duplicated genomic segments are identified by chromosome (color) and whether they are subtelomeric (bounded rectangles), non-subtelomeric (unbounded rectangles), or intra-chromosomal (located above the subtelomere coordinates). Each rectangle represents a separate duplicon. Bottom panels: duplicated genomic segments are the same as in the top panels, but identified by nucleotide sequence similarity with the query subtelomere sequence (color scheme as indicated in the key). [file gb-2007-8-7-r151-S24.pdf]

10q

0 kb Gap

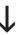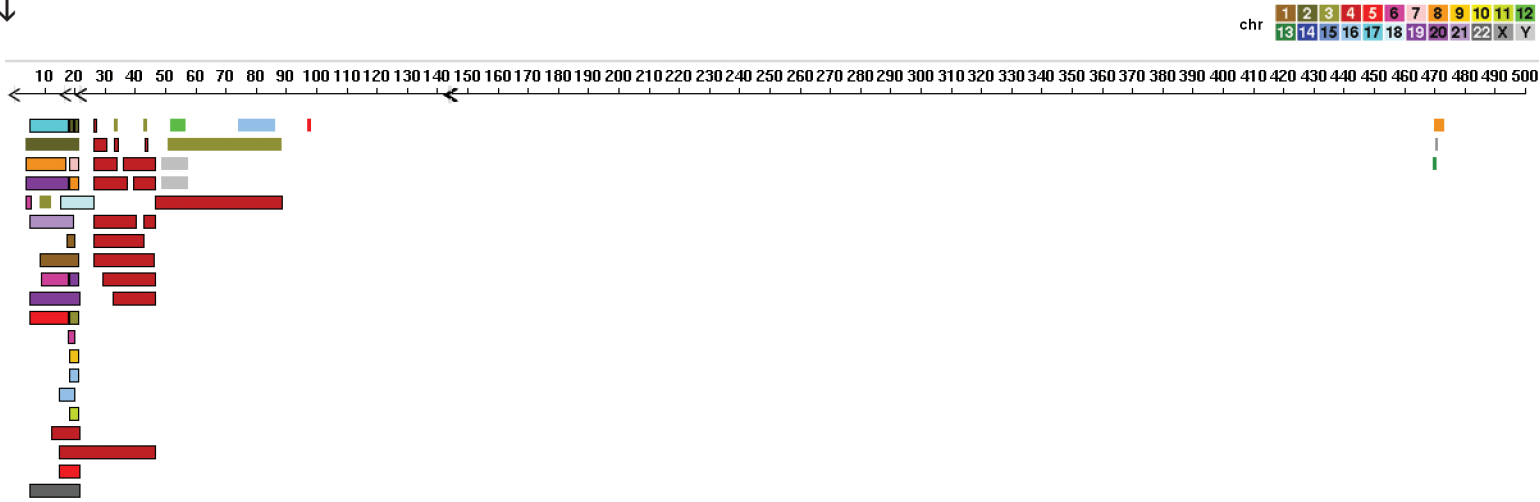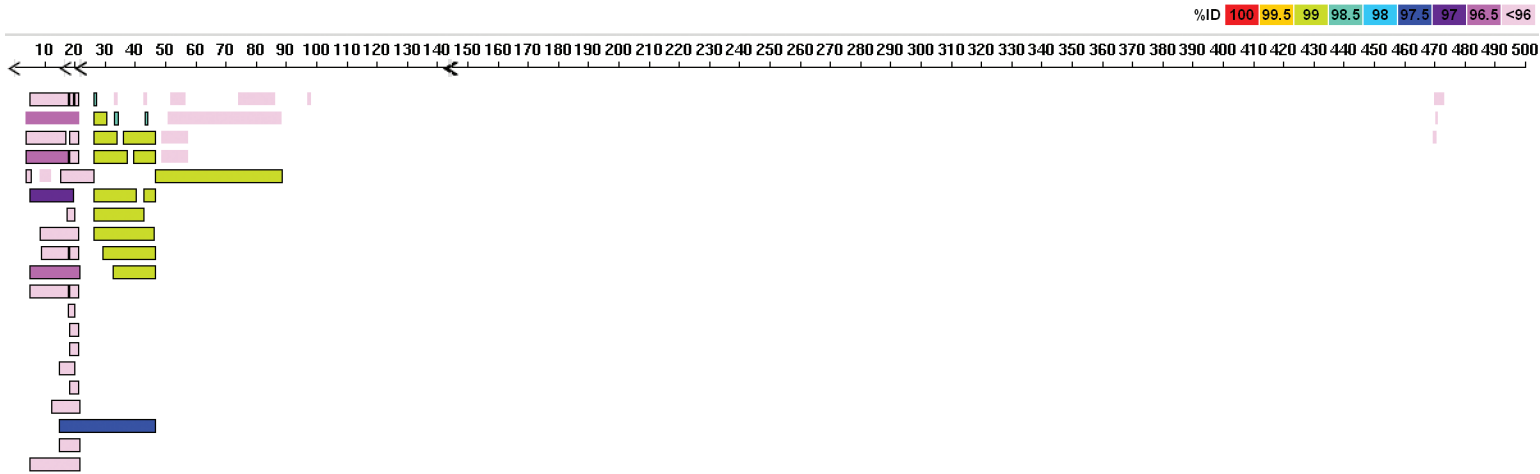

Supplement: Additional data file 25 — The subtelomere sequences shown are the assemblies published previously [6] and are available at the Riethman Lab website [47]. The telomeric end of each sequence assembly is located at the left. The distance from the end of the sequence to the start of the terminal repeat array is indicated by the vertical arrow at the telomeric end of the sequence. The position and orientation of (TTAGGG)n tracts are shown as black arrows. Top panels: duplicated genomic segments are identified by chromosome (color) and whether they are subtelomeric (bounded rectangles), non-subtelomeric (unbounded rectangles), or intra-chromosomal (located above the subtelomere coordinates). Each rectangle represents a separate duplicon. Bottom panels: duplicated genomic segments are the same as in the top panels, but identified by nucleotide sequence similarity with the query subtelomere sequence (color scheme as indicated in the key). [file gb-2007-8-7-r151-S25.pdf]

11p

150 kb Gap

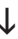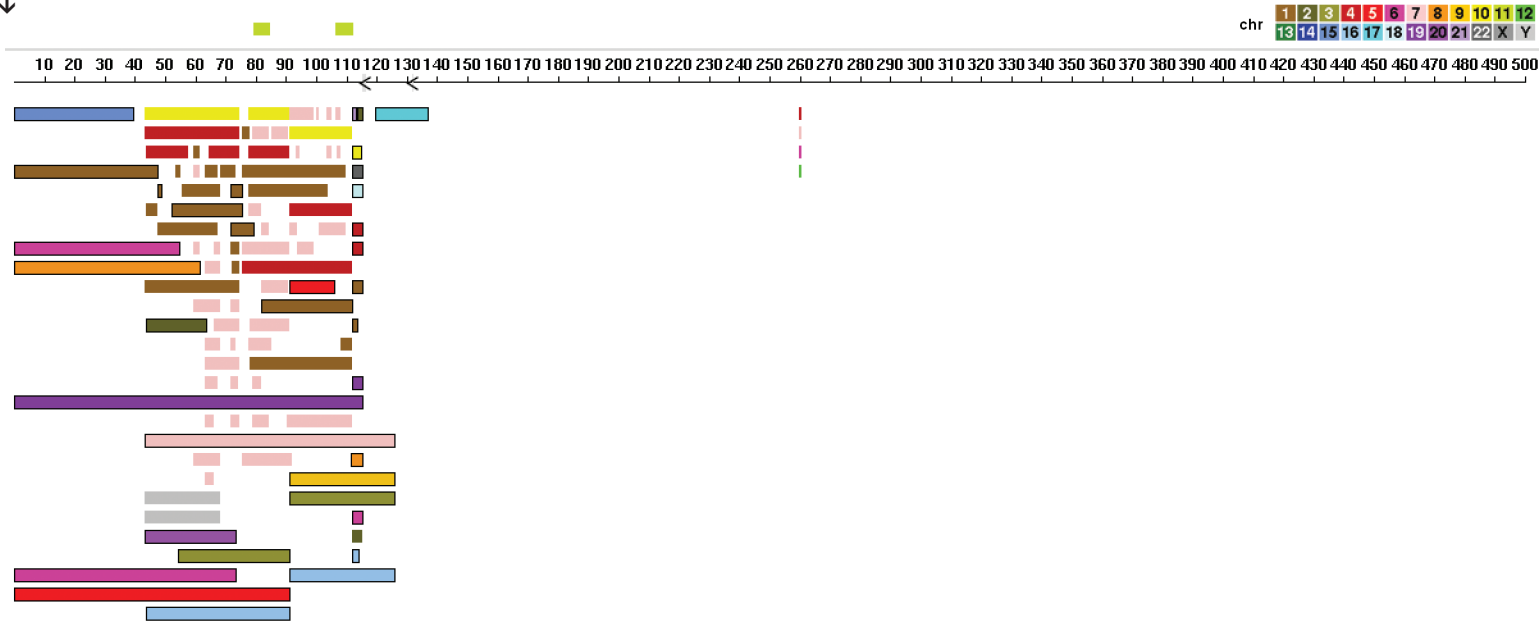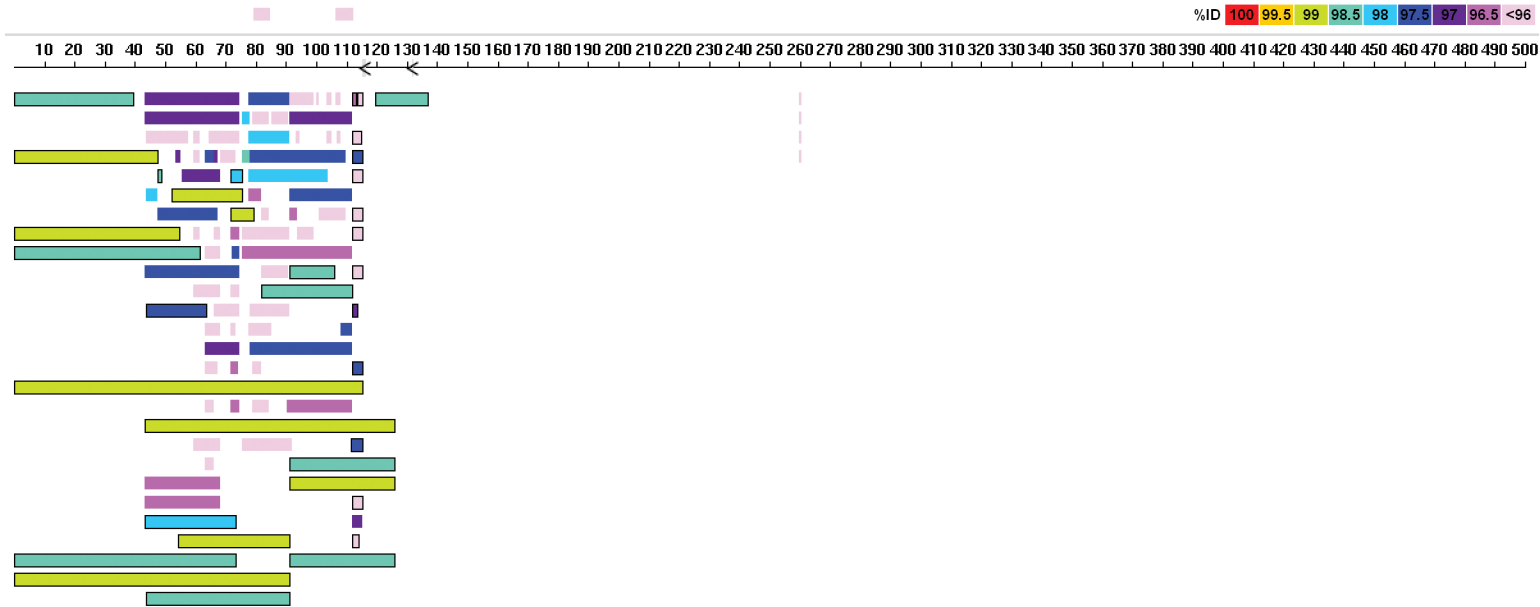

Supplement: Additional data file 26 — The subtelomere sequences shown are the assemblies published previously [6] and are available at the Riethman Lab website [47]. The telomeric end of each sequence assembly is located at the left. The distance from the end of the sequence to the start of the terminal repeat array is indicated by the vertical arrow at the telomeric end of the sequence. The position and orientation of (TTAGGG)n tracts are shown as black arrows. Top panels: duplicated genomic segments are identified by chromosome (color) and whether they are subtelomeric (bounded rectangles), non-subtelomeric (unbounded rectangles), or intra-chromosomal (located above the subtelomere coordinates). Each rectangle represents a separate duplicon. Bottom panels: duplicated genomic segments are the same as in the top panels, but identified by nucleotide sequence similarity with the query subtelomere sequence (color scheme as indicated in the key). [file gb-2007-8-7-r151-S26.pdf]

# 11q

0 kb Gap

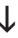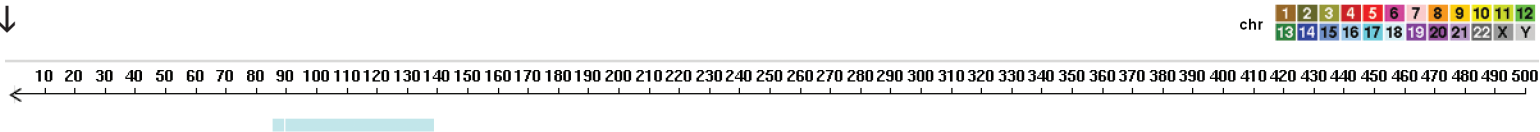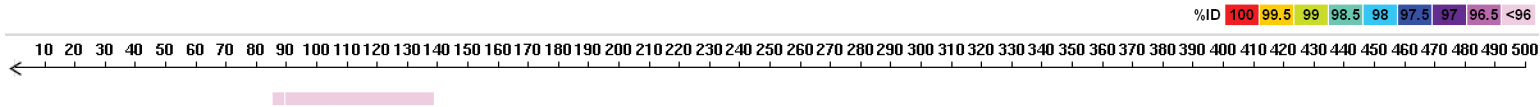

Supplement: Additional data file 27 — The subtelomere sequences shown are the assemblies published previously [6] and are available at the Riethman Lab website [47]. The telomeric end of each sequence assembly is located at the left. The distance from the end of the sequence to the start of the terminal repeat array is indicated by the vertical arrow at the telomeric end of the sequence. The position and orientation of (TTAGGG)n tracts are shown as black arrows. Top panels: duplicated genomic segments are identified by chromosome (color) and whether they are subtelomeric (bounded rectangles), non-subtelomeric (unbounded rectangles), or intra-chromosomal (located above the subtelomere coordinates). Each rectangle represents a separate duplicon. Bottom panels: duplicated genomic segments are the same as in the top panels, but identified by nucleotide sequence similarity with the query subtelomere sequence (color scheme as indicated in the key). [file gb-2007-8-7-r151-S27.pdf]

12p

16 kb Gap

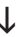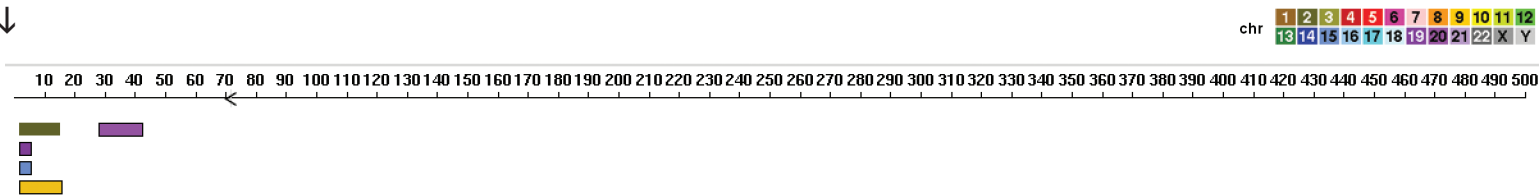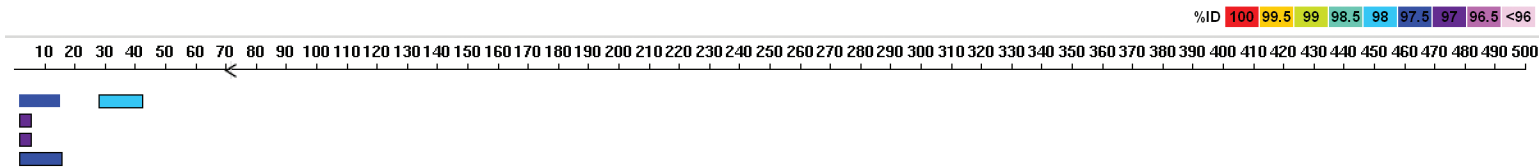

Supplement: Additional data file 28 — The subtelomere sequences shown are the assemblies published previously [6] and are available at the Riethman Lab website [47]. The telomeric end of each sequence assembly is located at the left. The distance from the end of the sequence to the start of the terminal repeat array is indicated by the vertical arrow at the telomeric end of the sequence. The position and orientation of (TTAGGG)n tracts are shown as black arrows. Top panels: duplicated genomic segments are identified by chromosome (color) and whether they are subtelomeric (bounded rectangles), non-subtelomeric (unbounded rectangles), or intra-chromosomal (located above the subtelomere coordinates). Each rectangle represents a separate duplicon. Bottom panels: duplicated genomic segments are the same as in the top panels, but identified by nucleotide sequence similarity with the query subtelomere sequence (color scheme as indicated in the key). [file gb-2007-8-7-r151-S28.pdf]

# 12q

60 kb Gap

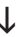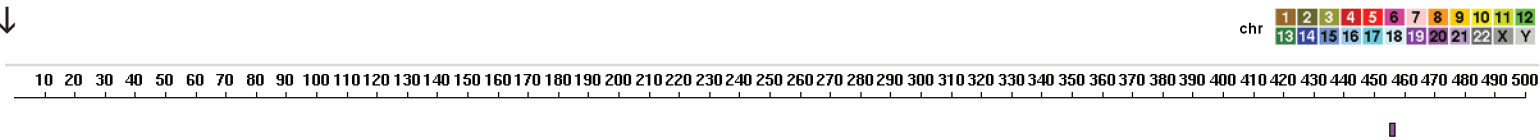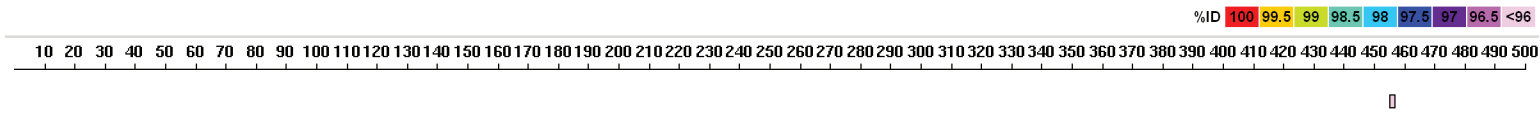

Supplement: Additional data file 29 — The subtelomere sequences shown are the assemblies published previously [6] and are available at the Riethman Lab website [47]. The telomeric end of each sequence assembly is located at the left. The distance from the end of the sequence to the start of the terminal repeat array is indicated by the vertical arrow at the telomeric end of the sequence. The position and orientation of (TTAGGG)n tracts are shown as black arrows. Top panels: duplicated genomic segments are identified by chromosome (color) and whether they are subtelomeric (bounded rectangles), non-subtelomeric (unbounded rectangles), or intra-chromosomal (located above the subtelomere coordinates). Each rectangle represents a separate duplicon. Bottom panels: duplicated genomic segments are the same as in the top panels, but identified by nucleotide sequence similarity with the query subtelomere sequence (color scheme as indicated in the key). [file gb-2007-8-7-r151-S29.pdf]

# 13q

15 kb Gap

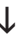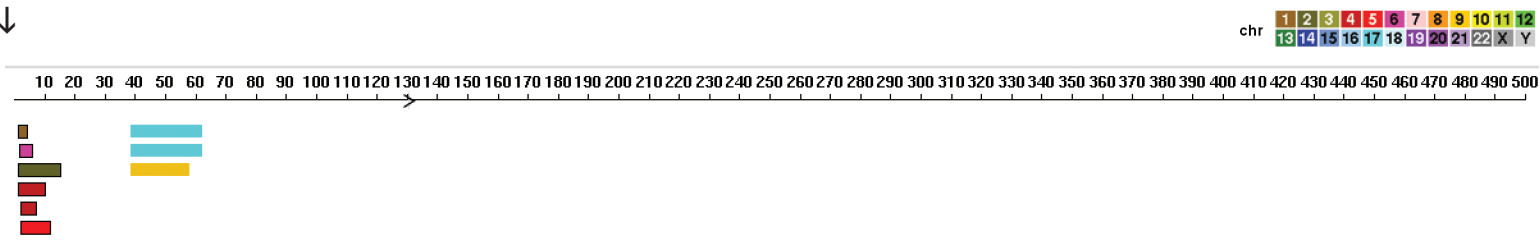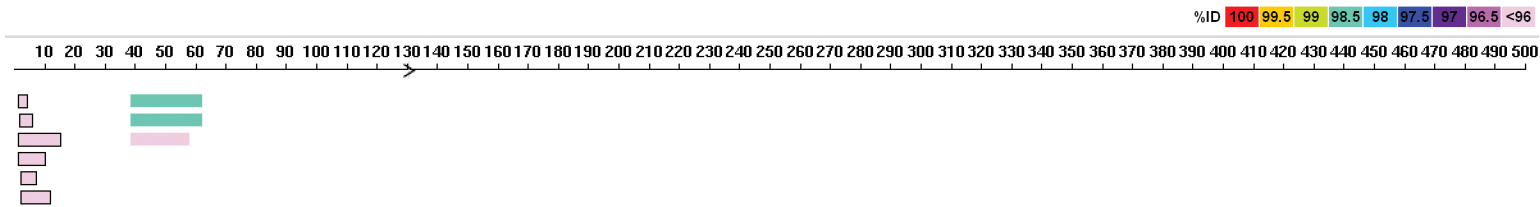

Supplement: Additional data file 30 — The subtelomere sequences shown are the assemblies published previously [6] and are available at the Riethman Lab website [47]. The telomeric end of each sequence assembly is located at the left. The distance from the end of the sequence to the start of the terminal repeat array is indicated by the vertical arrow at the telomeric end of the sequence. The position and orientation of (TTAGGG)n tracts are shown as black arrows. Top panels: duplicated genomic segments are identified by chromosome (color) and whether they are subtelomeric (bounded rectangles), non-subtelomeric (unbounded rectangles), or intra-chromosomal (located above the subtelomere coordinates). Each rectangle represents a separate duplicon. Bottom panels: duplicated genomic segments are the same as in the top panels, but identified by nucleotide sequence similarity with the query subtelomere sequence (color scheme as indicated in the key). [file gb-2007-8-7-r151-S30.pdf]

# 14q

7 kb Gap  
↓

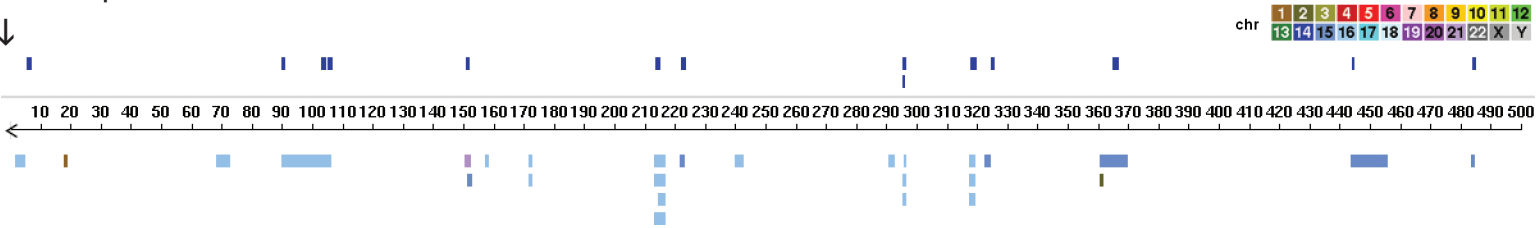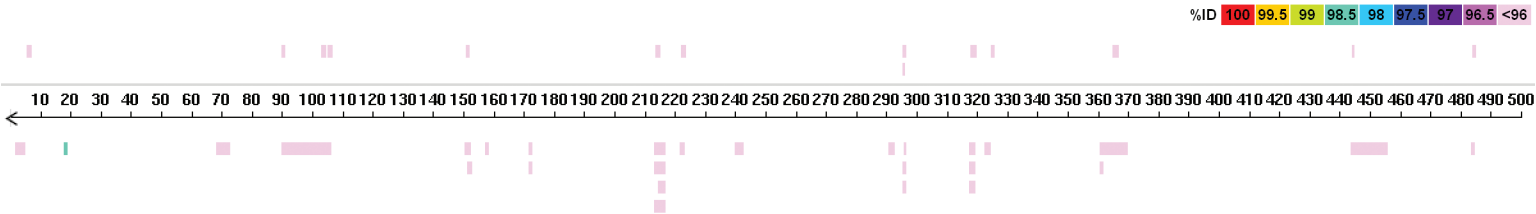

Supplement: Additional data file 31 — The subtelomere sequences shown are the assemblies published previously [6] and are available at the Riethman Lab website [47]. The telomeric end of each sequence assembly is located at the left. The distance from the end of the sequence to the start of the terminal repeat array is indicated by the vertical arrow at the telomeric end of the sequence. The position and orientation of (TTAGGG)n tracts are shown as black arrows. Top panels: duplicated genomic segments are identified by chromosome (color) and whether they are subtelomeric (bounded rectangles), non-subtelomeric (unbounded rectangles), or intra-chromosomal (located above the subtelomere coordinates). Each rectangle represents a separate duplicon. Bottom panels: duplicated genomic segments are the same as in the top panels, but identified by nucleotide sequence similarity with the query subtelomere sequence (color scheme as indicated in the key). [file gb-2007-8-7-r151-S31.pdf]

# 15q

0 kb Gap

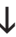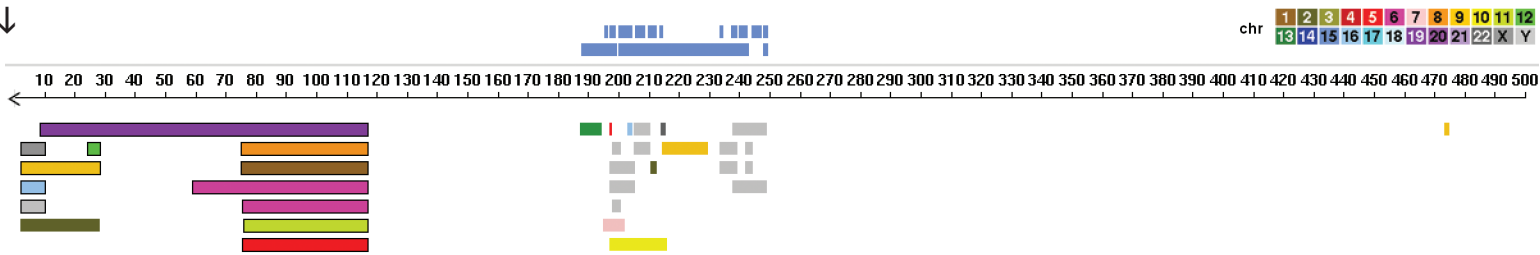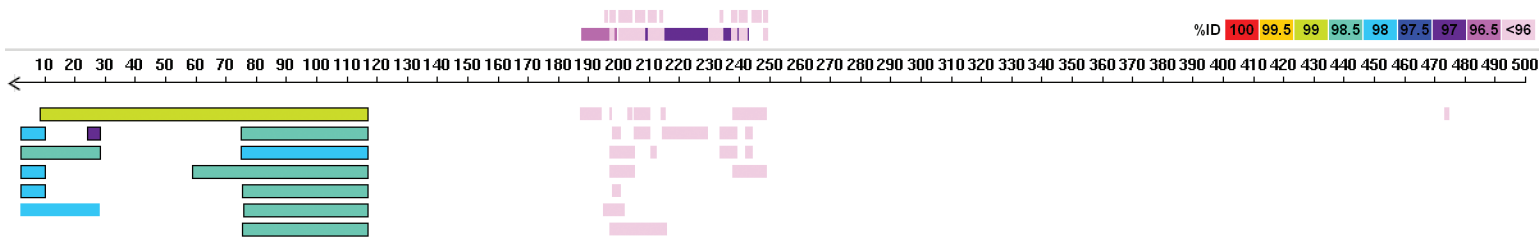

Supplement: Additional data file 32 — The subtelomere sequences shown are the assemblies published previously [6] and are available at the Riethman Lab website [47]. The telomeric end of each sequence assembly is located at the left. The distance from the end of the sequence to the start of the terminal repeat array is indicated by the vertical arrow at the telomeric end of the sequence. The position and orientation of (TTAGGG)n tracts are shown as black arrows. Top panels: duplicated genomic segments are identified by chromosome (color) and whether they are subtelomeric (bounded rectangles), non-subtelomeric (unbounded rectangles), or intra-chromosomal (located above the subtelomere coordinates). Each rectangle represents a separate duplicon. Bottom panels: duplicated genomic segments are the same as in the top panels, but identified by nucleotide sequence similarity with the query subtelomere sequence (color scheme as indicated in the key). [file gb-2007-8-7-r151-S32.pdf]

16p

0 kb Gap

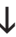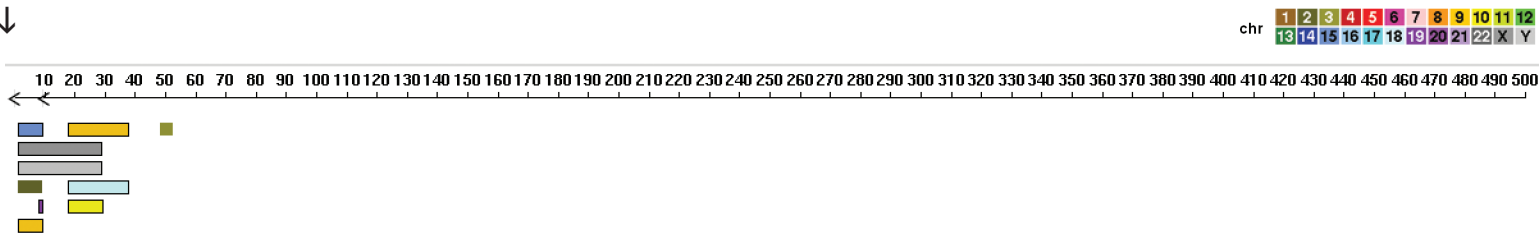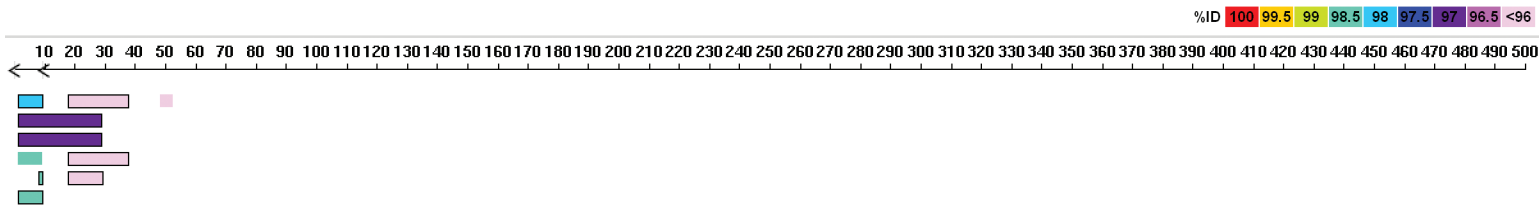

Supplement: Additional data file 33 — The subtelomere sequences shown are the assemblies published previously [6] and are available at the Riethman Lab website [47]. The telomeric end of each sequence assembly is located at the left. The distance from the end of the sequence to the start of the terminal repeat array is indicated by the vertical arrow at the telomeric end of the sequence. The position and orientation of (TTAGGG)n tracts are shown as black arrows. Top panels: duplicated genomic segments are identified by chromosome (color) and whether they are subtelomeric (bounded rectangles), non-subtelomeric (unbounded rectangles), or intra-chromosomal (located above the subtelomere coordinates). Each rectangle represents a separate duplicon. Bottom panels: duplicated genomic segments are the same as in the top panels, but identified by nucleotide sequence similarity with the query subtelomere sequence (color scheme as indicated in the key). [file gb-2007-8-7-r151-S33.pdf]

16q

5 kb Gap

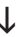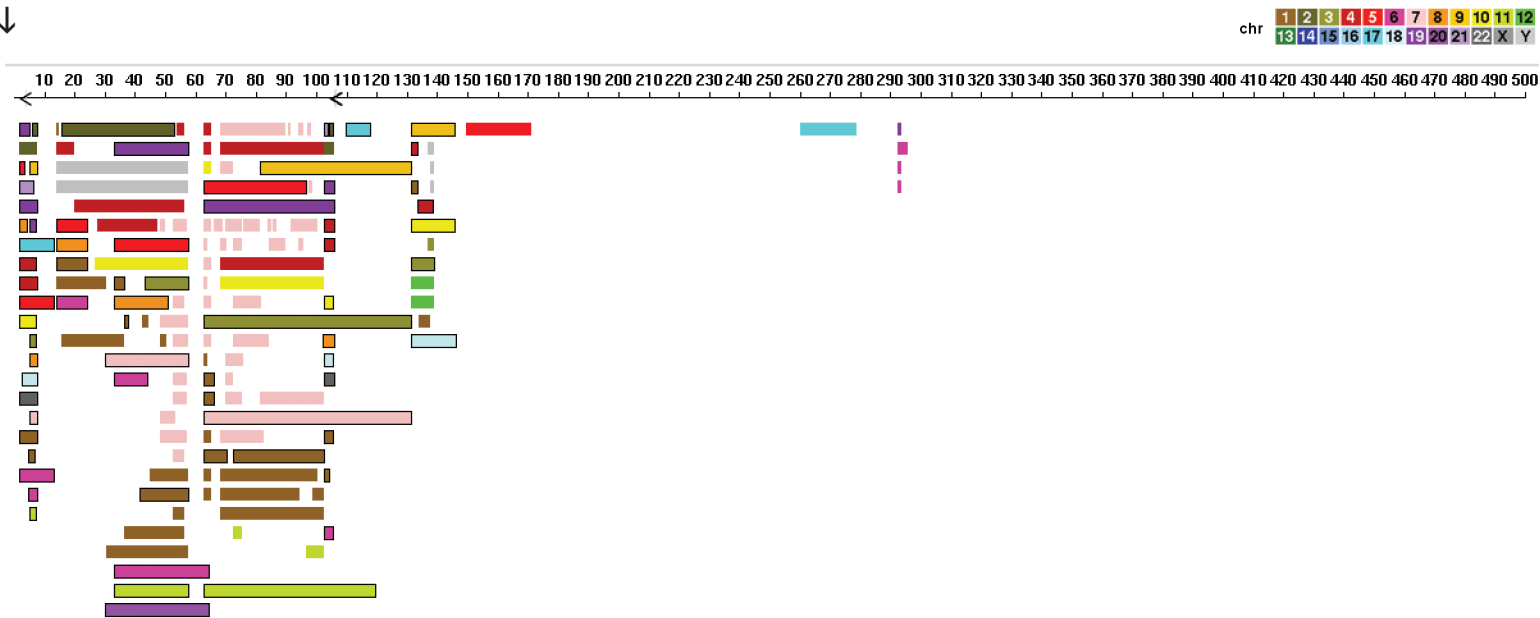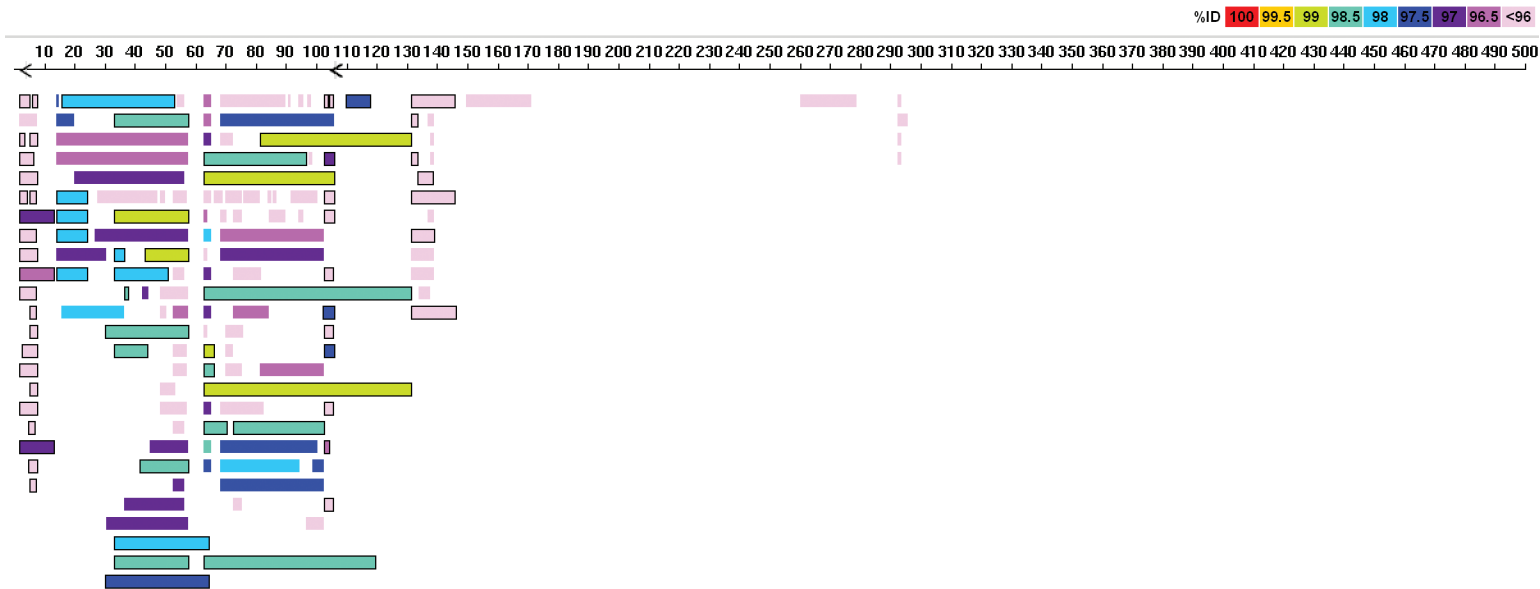

Supplement: Additional data file 34 — The subtelomere sequences shown are the assemblies published previously [6] and are available at the Riethman Lab website [47]. The telomeric end of each sequence assembly is located at the left. The distance from the end of the sequence to the start of the terminal repeat array is indicated by the vertical arrow at the telomeric end of the sequence. The position and orientation of (TTAGGG)n tracts are shown as black arrows. Top panels: duplicated genomic segments are identified by chromosome (color) and whether they are subtelomeric (bounded rectangles), non-subtelomeric (unbounded rectangles), or intra-chromosomal (located above the subtelomere coordinates). Each rectangle represents a separate duplicon. Bottom panels: duplicated genomic segments are the same as in the top panels, but identified by nucleotide sequence similarity with the query subtelomere sequence (color scheme as indicated in the key). [file gb-2007-8-7-r151-S34.pdf]

17p

2 kb Gap

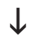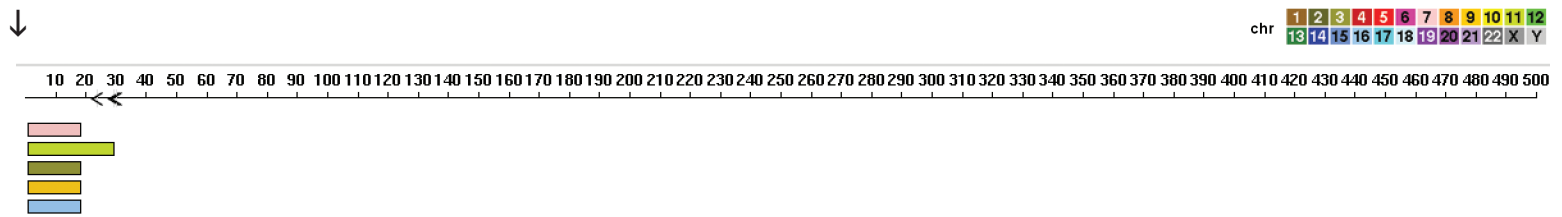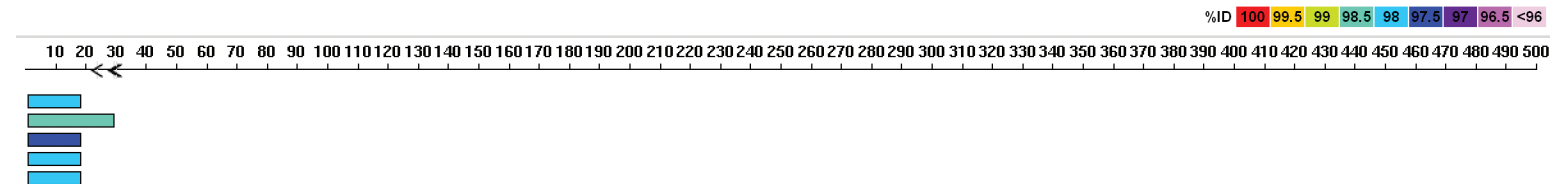

Supplement: Additional data file 35 — The subtelomere sequences shown are the assemblies published previously [6] and are available at the Riethman Lab website [47]. The telomeric end of each sequence assembly is located at the left. The distance from the end of the sequence to the start of the terminal repeat array is indicated by the vertical arrow at the telomeric end of the sequence. The position and orientation of (TTAGGG)n tracts are shown as black arrows. Top panels: duplicated genomic segments are identified by chromosome (color) and whether they are subtelomeric (bounded rectangles), non-subtelomeric (unbounded rectangles), or intra-chromosomal (located above the subtelomere coordinates). Each rectangle represents a separate duplicon. Bottom panels: duplicated genomic segments are the same as in the top panels, but identified by nucleotide sequence similarity with the query subtelomere sequence (color scheme as indicated in the key). [file gb-2007-8-7-r151-S35.pdf]

17q

0 kb Gap

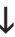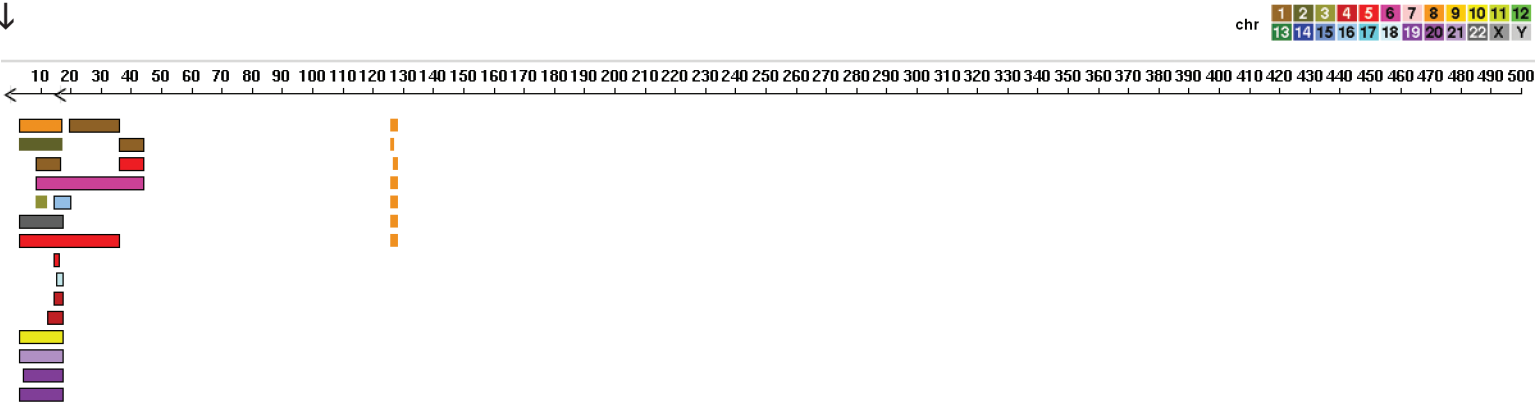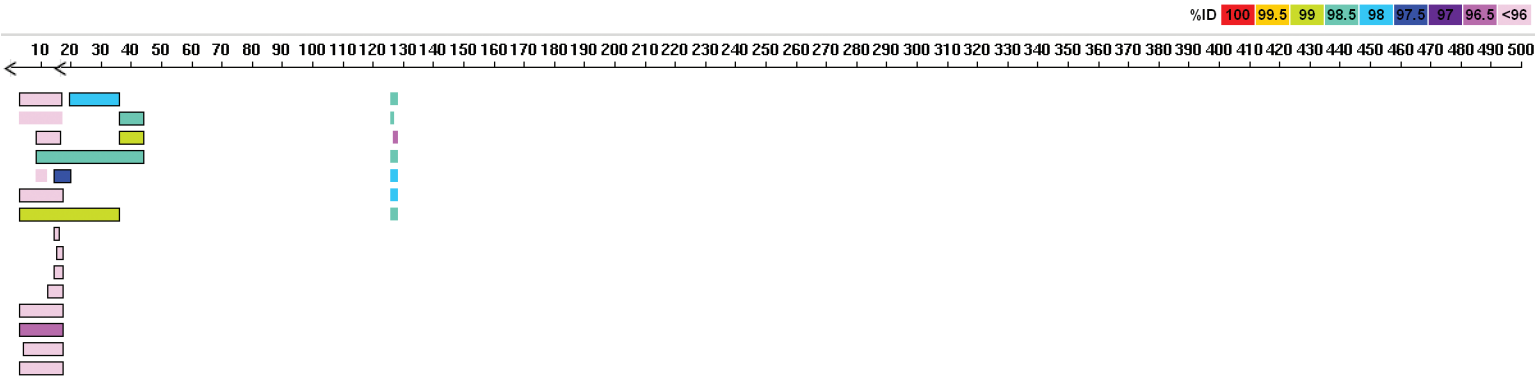

Supplement: Additional data file 36 — The subtelomere sequences shown are the assemblies published previously [6] and are available at the Riethman Lab website [47]. The telomeric end of each sequence assembly is located at the left. The distance from the end of the sequence to the start of the terminal repeat array is indicated by the vertical arrow at the telomeric end of the sequence. The position and orientation of (TTAGGG)n tracts are shown as black arrows. Top panels: duplicated genomic segments are identified by chromosome (color) and whether they are subtelomeric (bounded rectangles), non-subtelomeric (unbounded rectangles), or intra-chromosomal (located above the subtelomere coordinates). Each rectangle represents a separate duplicon. Bottom panels: duplicated genomic segments are the same as in the top panels, but identified by nucleotide sequence similarity with the query subtelomere sequence (color scheme as indicated in the key). [file gb-2007-8-7-r151-S36.pdf]

18p

0 kb Gap

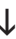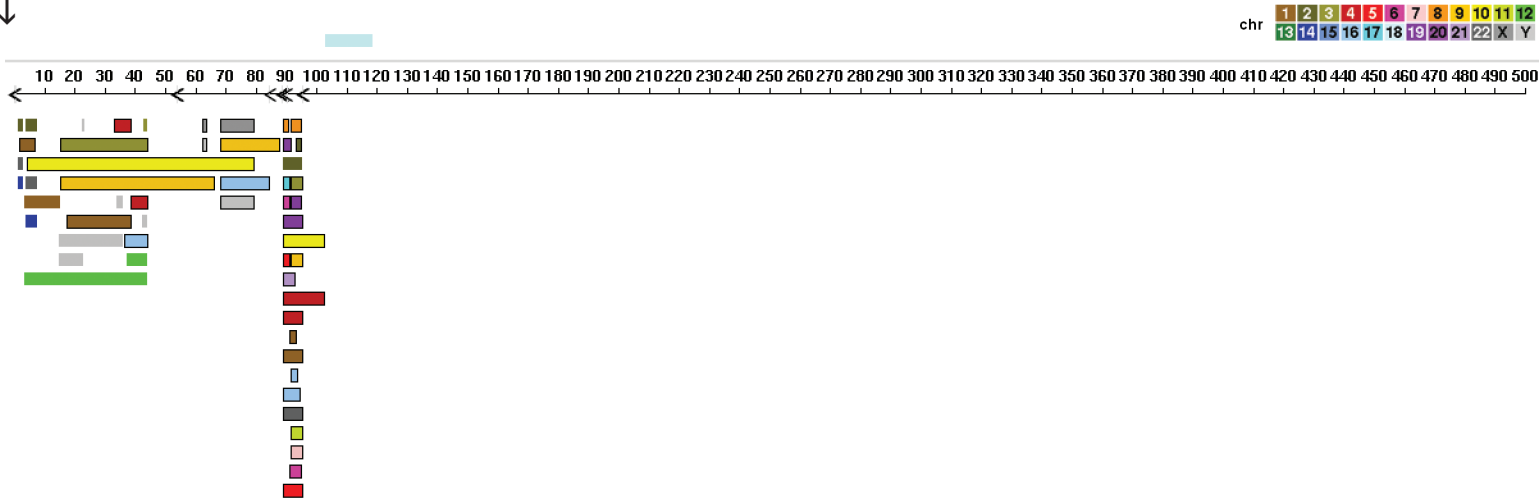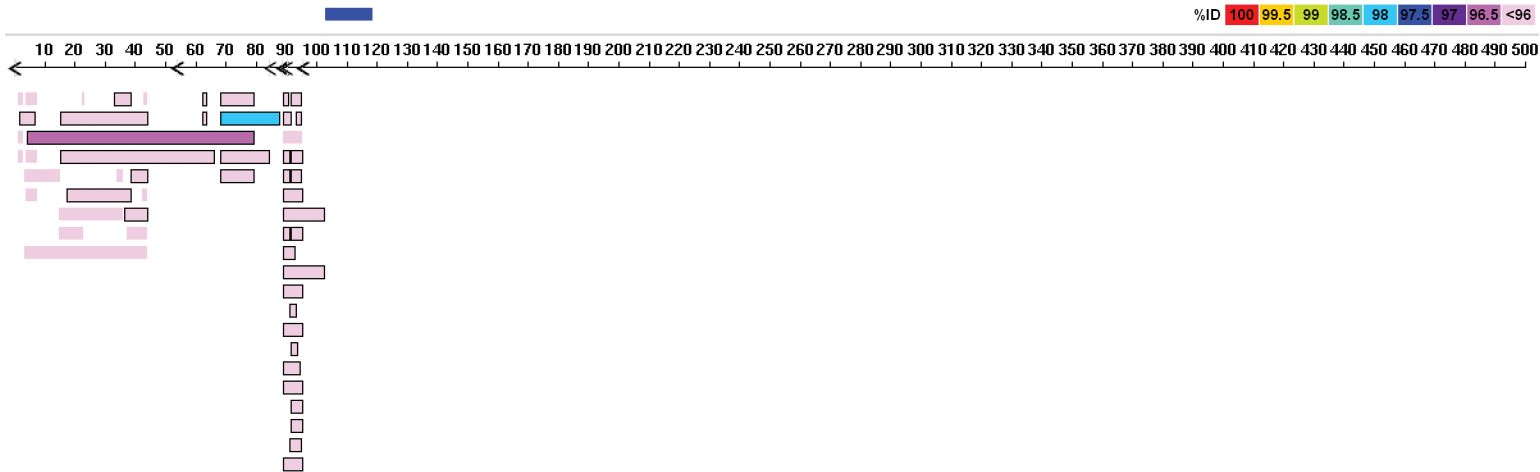

Supplement: Additional data file 37 — The subtelomere sequences shown are the assemblies published previously [6] and are available at the Riethman Lab website [47]. The telomeric end of each sequence assembly is located at the left. The distance from the end of the sequence to the start of the terminal repeat array is indicated by the vertical arrow at the telomeric end of the sequence. The position and orientation of (TTAGGG)n tracts are shown as black arrows. Top panels: duplicated genomic segments are identified by chromosome (color) and whether they are subtelomeric (bounded rectangles), non-subtelomeric (unbounded rectangles), or intra-chromosomal (located above the subtelomere coordinates). Each rectangle represents a separate duplicon. Bottom panels: duplicated genomic segments are the same as in the top panels, but identified by nucleotide sequence similarity with the query subtelomere sequence (color scheme as indicated in the key). [file gb-2007-8-7-r151-S37.pdf]

# 18q

0 kb Gap

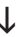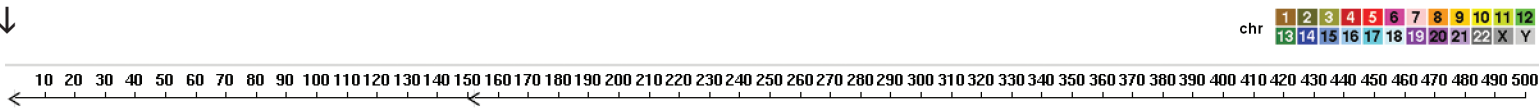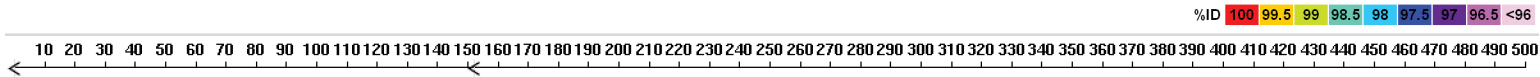

Supplement: Additional data file 38 — The subtelomere sequences shown are the assemblies published previously [6] and are available at the Riethman Lab website [47]. The telomeric end of each sequence assembly is located at the left. The distance from the end of the sequence to the start of the terminal repeat array is indicated by the vertical arrow at the telomeric end of the sequence. The position and orientation of (TTAGGG)n tracts are shown as black arrows. Top panels: duplicated genomic segments are identified by chromosome (color) and whether they are subtelomeric (bounded rectangles), non-subtelomeric (unbounded rectangles), or intra-chromosomal (located above the subtelomere coordinates). Each rectangle represents a separate duplicon. Bottom panels: duplicated genomic segments are the same as in the top panels, but identified by nucleotide sequence similarity with the query subtelomere sequence (color scheme as indicated in the key). [file gb-2007-8-7-r151-S38.pdf]

19p

11 kb Gap

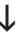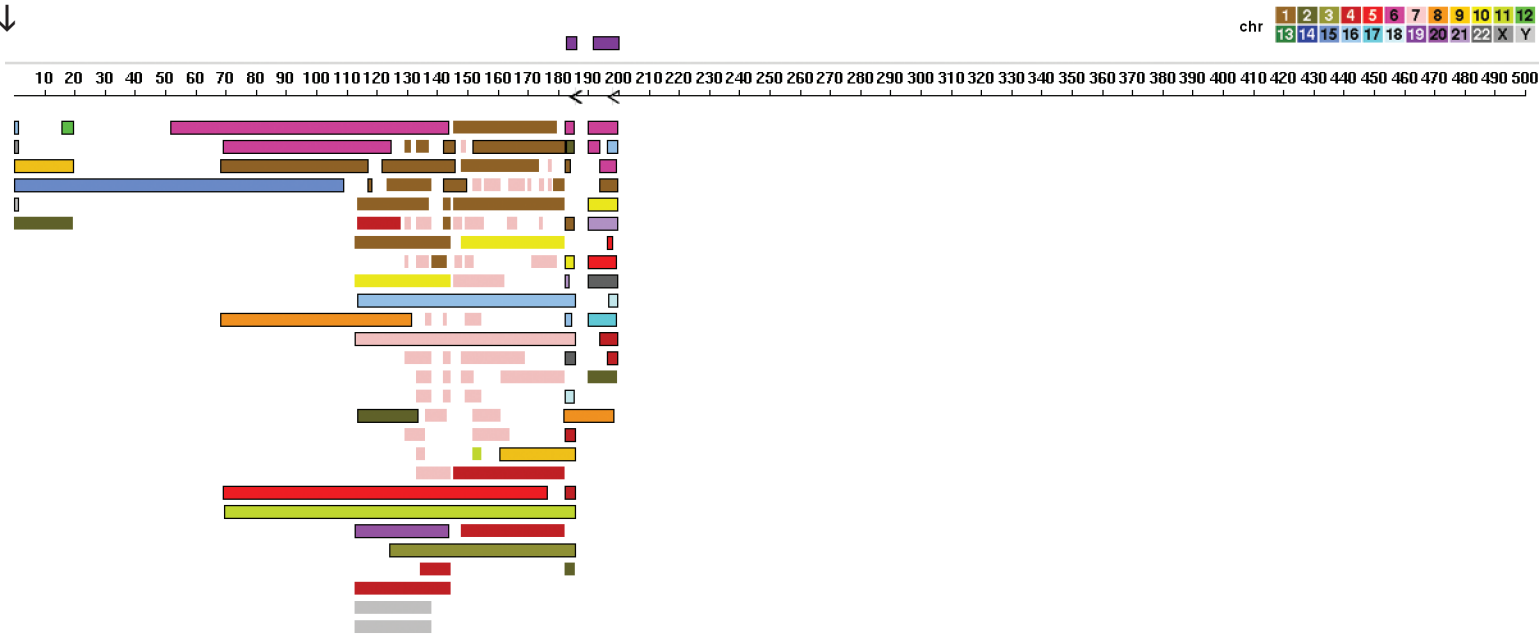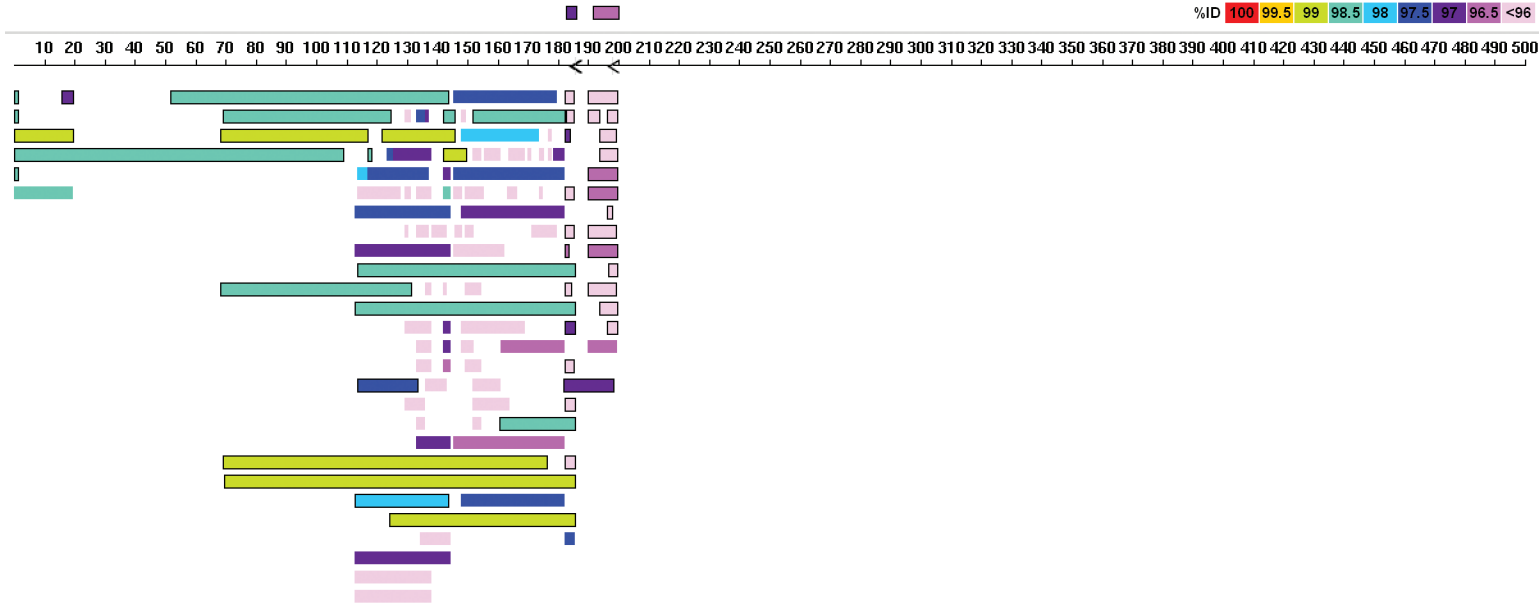

Supplement: Additional data file 39 — The subtelomere sequences shown are the assemblies published previously [6] and are available at the Riethman Lab website [47]. The telomeric end of each sequence assembly is located at the left. The distance from the end of the sequence to the start of the terminal repeat array is indicated by the vertical arrow at the telomeric end of the sequence. The position and orientation of (TTAGGG)n tracts are shown as black arrows. Top panels: duplicated genomic segments are identified by chromosome (color) and whether they are subtelomeric (bounded rectangles), non-subtelomeric (unbounded rectangles), or intra-chromosomal (located above the subtelomere coordinates). Each rectangle represents a separate duplicon. Bottom panels: duplicated genomic segments are the same as in the top panels, but identified by nucleotide sequence similarity with the query subtelomere sequence (color scheme as indicated in the key). [file gb-2007-8-7-r151-S39.pdf]

19q

5 kb Gap

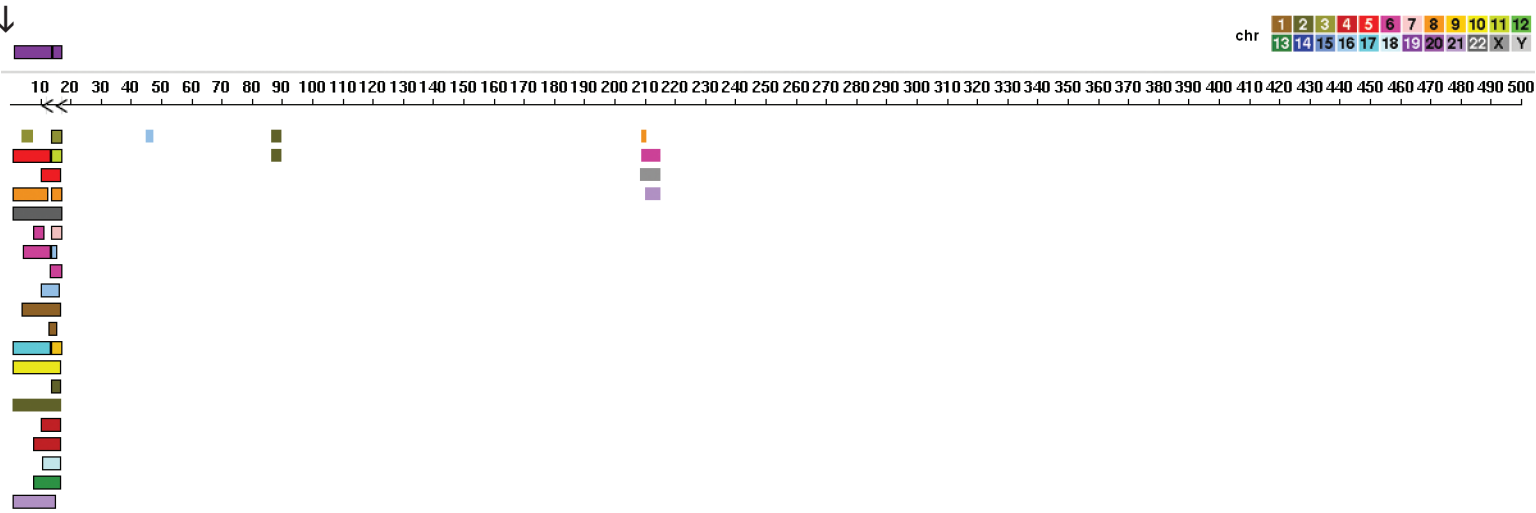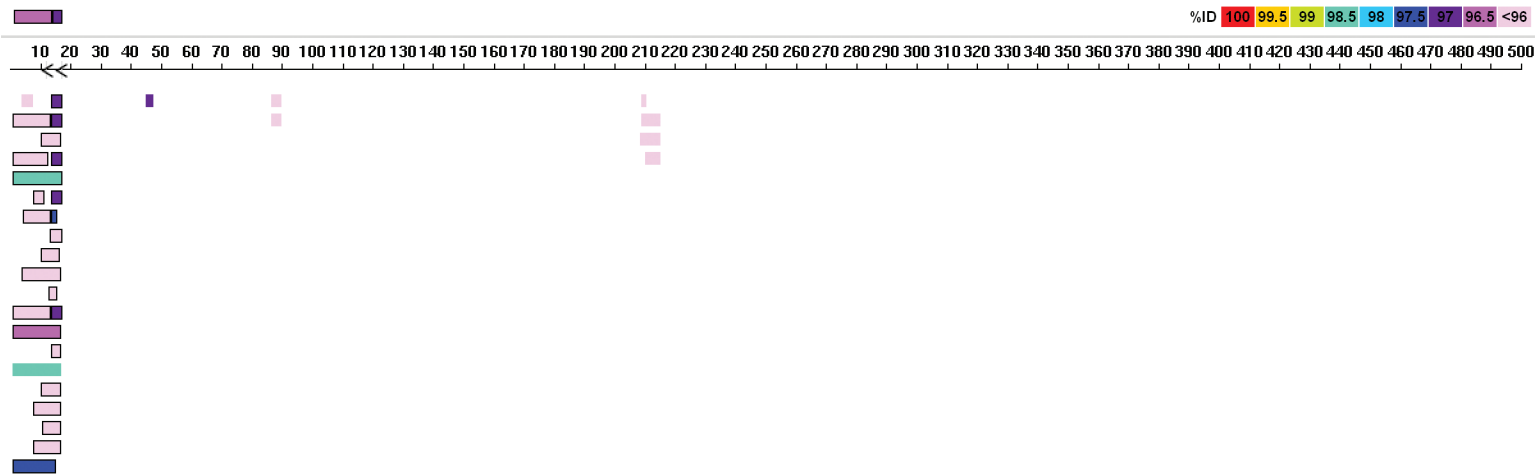

Supplement: Additional data file 40 — The subtelomere sequences shown are the assemblies published previously [6] and are available at the Riethman Lab website [47]. The telomeric end of each sequence assembly is located at the left. The distance from the end of the sequence to the start of the terminal repeat array is indicated by the vertical arrow at the telomeric end of the sequence. The position and orientation of (TTAGGG)n tracts are shown as black arrows. Top panels: duplicated genomic segments are identified by chromosome (color) and whether they are subtelomeric (bounded rectangles), non-subtelomeric (unbounded rectangles), or intra-chromosomal (located above the subtelomere coordinates). Each rectangle represents a separate duplicon. Bottom panels: duplicated genomic segments are the same as in the top panels, but identified by nucleotide sequence similarity with the query subtelomere sequence (color scheme as indicated in the key). [file gb-2007-8-7-r151-S40.pdf]

20p

105 kb Gap

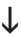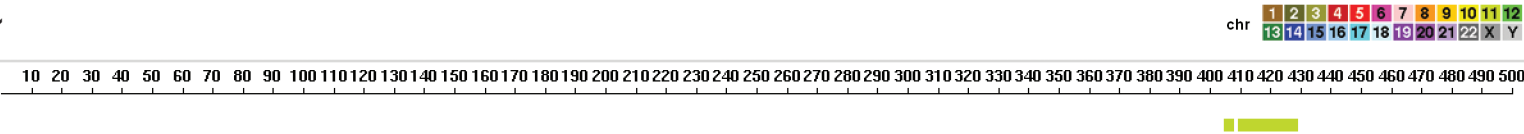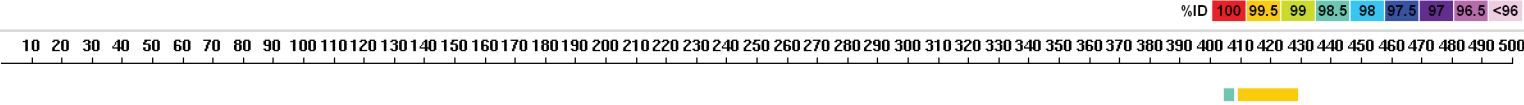

Supplement: Additional data file 41 — The subtelomere sequences shown are the assemblies published previously [6] and are available at the Riethman Lab website [47]. The telomeric end of each sequence assembly is located at the left. The distance from the end of the sequence to the start of the terminal repeat array is indicated by the vertical arrow at the telomeric end of the sequence. The position and orientation of (TTAGGG)n tracts are shown as black arrows. Top panels: duplicated genomic segments are identified by chromosome (color) and whether they are subtelomeric (bounded rectangles), non-subtelomeric (unbounded rectangles), or intra-chromosomal (located above the subtelomere coordinates). Each rectangle represents a separate duplicon. Bottom panels: duplicated genomic segments are the same as in the top panels, but identified by nucleotide sequence similarity with the query subtelomere sequence (color scheme as indicated in the key). [file gb-2007-8-7-r151-S41.pdf]

20q

50 kb Gap

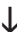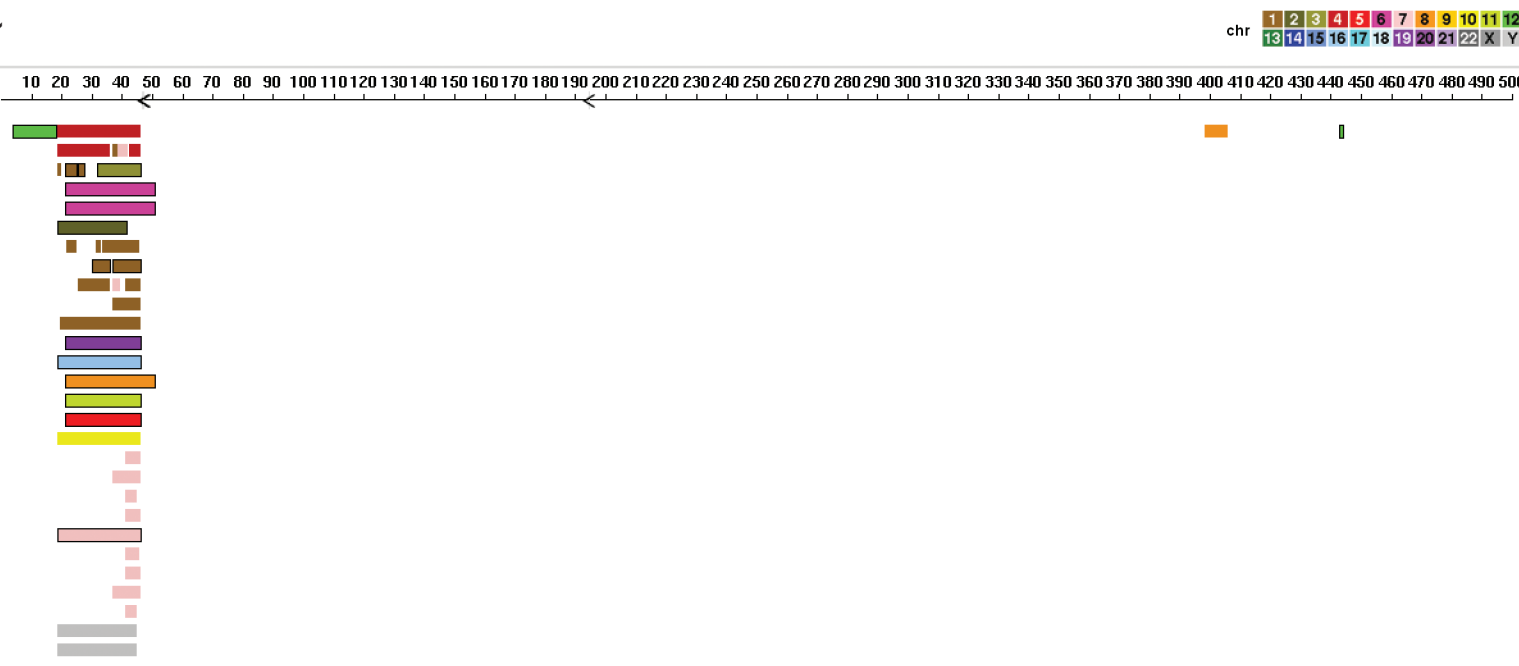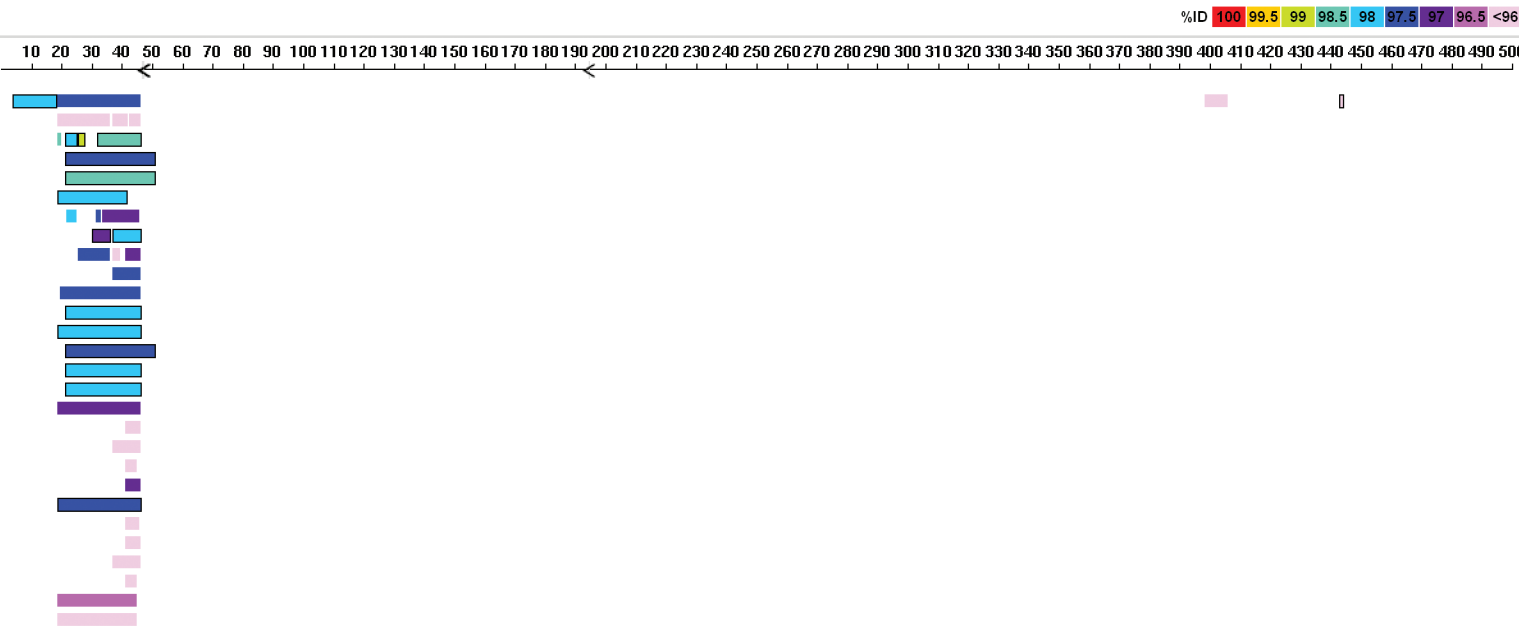

Supplement: Additional data file 42 — The subtelomere sequences shown are the assemblies published previously [6] and are available at the Riethman Lab website [47]. The telomeric end of each sequence assembly is located at the left. The distance from the end of the sequence to the start of the terminal repeat array is indicated by the vertical arrow at the telomeric end of the sequence. The position and orientation of (TTAGGG)n tracts are shown as black arrows. Top panels: duplicated genomic segments are identified by chromosome (color) and whether they are subtelomeric (bounded rectangles), non-subtelomeric (unbounded rectangles), or intra-chromosomal (located above the subtelomere coordinates). Each rectangle represents a separate duplicon. Bottom panels: duplicated genomic segments are the same as in the top panels, but identified by nucleotide sequence similarity with the query subtelomere sequence (color scheme as indicated in the key). [file gb-2007-8-7-r151-S42.pdf]

**21q**

0 kb Gap

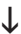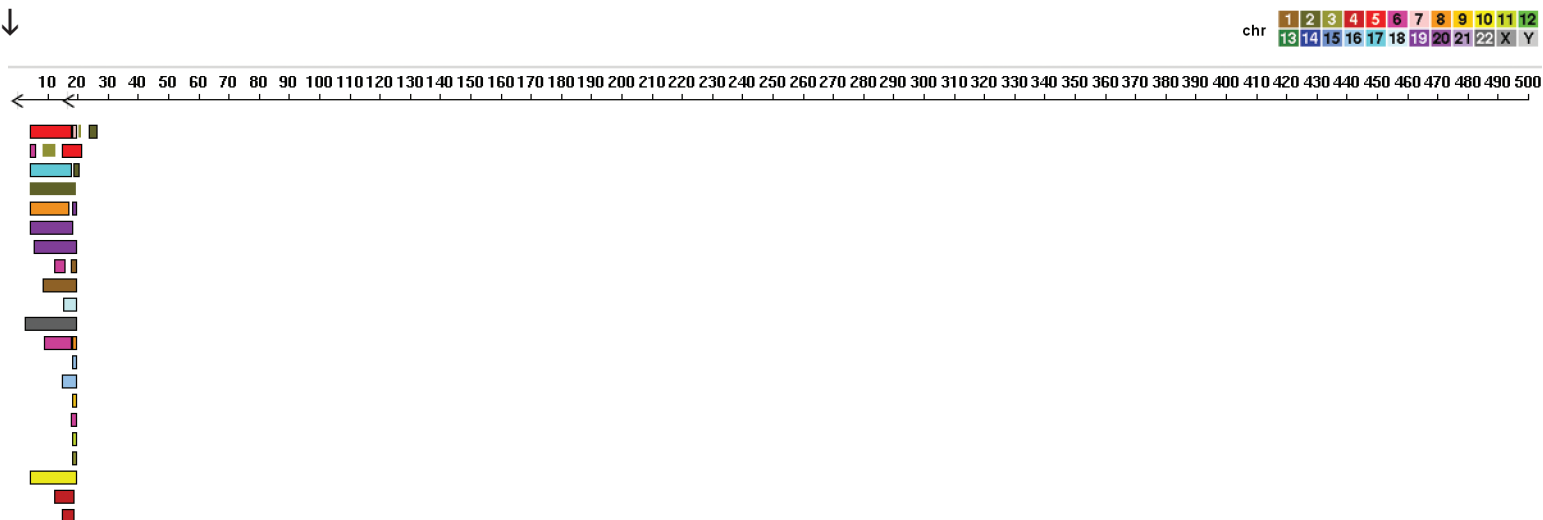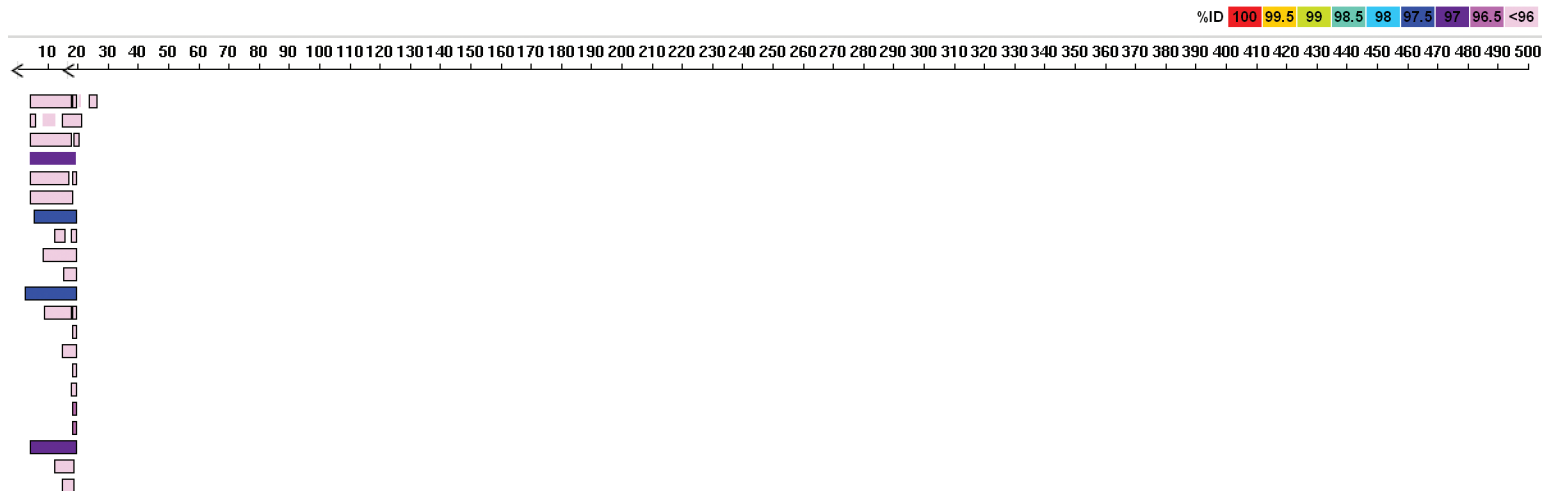

Supplement: Additional data file 43 — The subtelomere sequences shown are the assemblies published previously [6] and are available at the Riethman Lab website [47]. The telomeric end of each sequence assembly is located at the left. The distance from the end of the sequence to the start of the terminal repeat array is indicated by the vertical arrow at the telomeric end of the sequence. The position and orientation of (TTAGGG)n tracts are shown as black arrows. Top panels: duplicated genomic segments are identified by chromosome (color) and whether they are subtelomeric (bounded rectangles), non-subtelomeric (unbounded rectangles), or intra-chromosomal (located above the subtelomere coordinates). Each rectangle represents a separate duplicon. Bottom panels: duplicated genomic segments are the same as in the top panels, but identified by nucleotide sequence similarity with the query subtelomere sequence (color scheme as indicated in the key). [file gb-2007-8-7-r151-S43.pdf]

# 22q

20 kb Gap

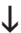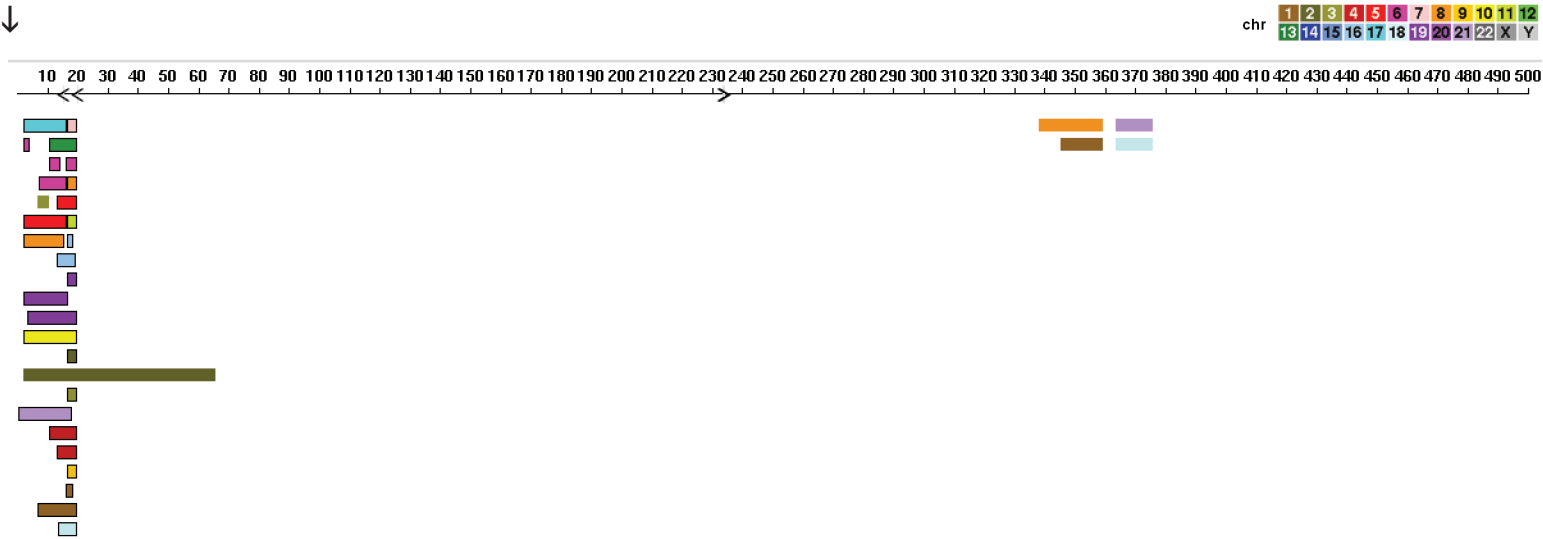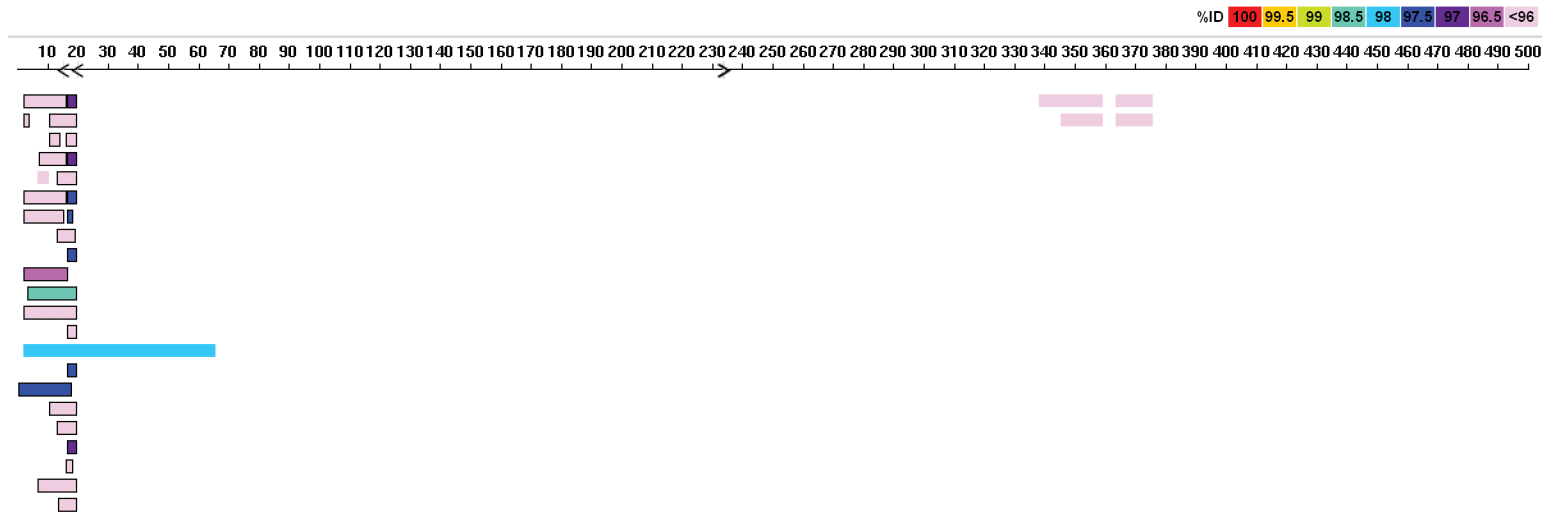

Supplement: Additional data file 44 — The subtelomere sequences shown are the assemblies published previously [6] and are available at the Riethman Lab website [47]. The telomeric end of each sequence assembly is located at the left. The distance from the end of the sequence to the start of the terminal repeat array is indicated by the vertical arrow at the telomeric end of the sequence. The position and orientation of (TTAGGG)n tracts are shown as black arrows. Top panels: duplicated genomic segments are identified by chromosome (color) and whether they are subtelomeric (bounded rectangles), non-subtelomeric (unbounded rectangles), or intra-chromosomal (located above the subtelomere coordinates). Each rectangle represents a separate duplicon. Bottom panels: duplicated genomic segments are the same as in the top panels, but identified by nucleotide sequence similarity with the query subtelomere sequence (color scheme as indicated in the key). [file gb-2007-8-7-r151-S44.pdf]

# Xp/Yp

0 kb Gap

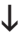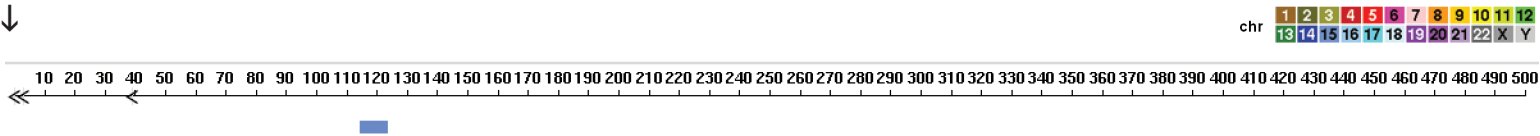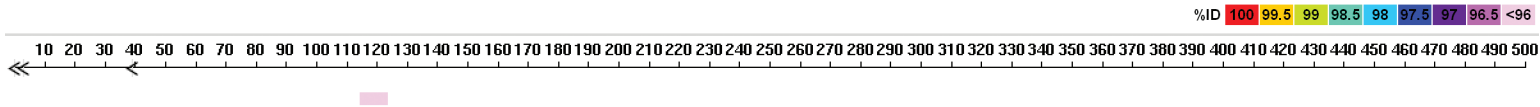

Supplement: Additional data file 45 — The subtelomere sequences shown are the assemblies published previously [6] and are available at the Riethman Lab website [47]. The telomeric end of each sequence assembly is located at the left. The distance from the end of the sequence to the start of the terminal repeat array is indicated by the vertical arrow at the telomeric end of the sequence. The position and orientation of (TTAGGG)n tracts are shown as black arrows. Top panels: duplicated genomic segments are identified by chromosome (color) and whether they are subtelomeric (bounded rectangles), non-subtelomeric (unbounded rectangles), or intra-chromosomal (located above the subtelomere coordinates). Each rectangle represents a separate duplicon. Bottom panels: duplicated genomic segments are the same as in the top panels, but identified by nucleotide sequence similarity with the query subtelomere sequence (color scheme as indicated in the key). [file gb-2007-8-7-r151-S45.pdf]

**Xq**

0 kb Gap

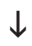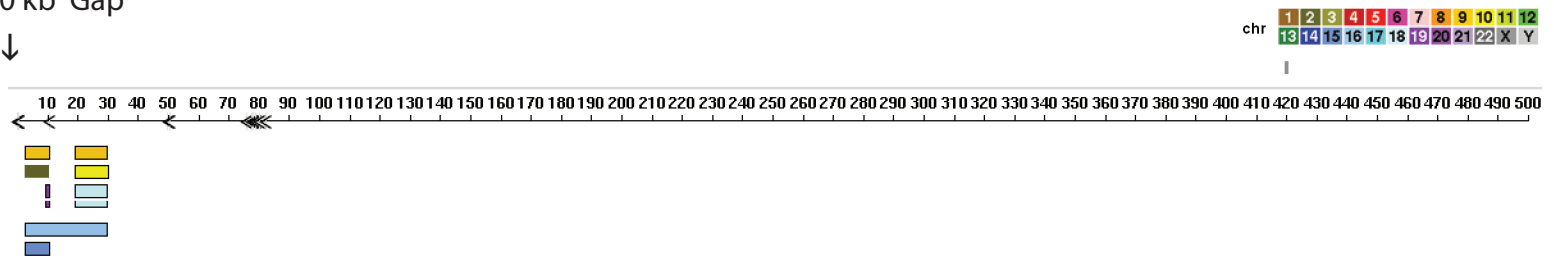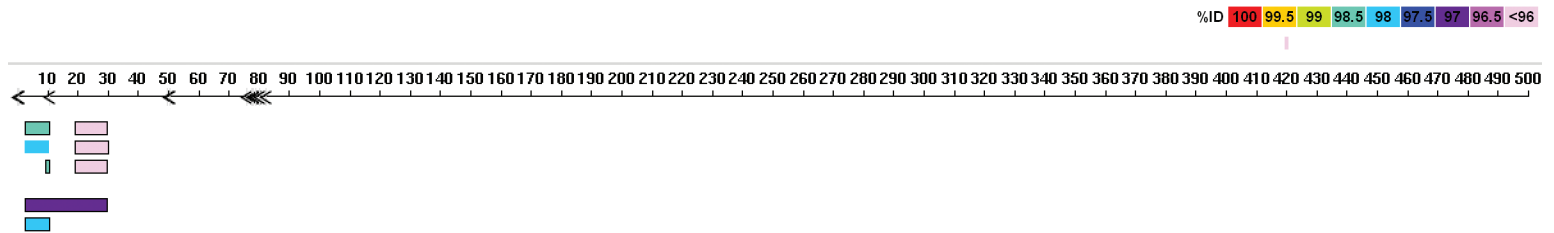

Supplement: Additional data file 46 — The subtelomere sequences shown are the assemblies published previously [6] and are available at the Riethman Lab website [47]. The telomeric end of each sequence assembly is located at the left. The distance from the end of the sequence to the start of the terminal repeat array is indicated by the vertical arrow at the telomeric end of the sequence. The position and orientation of (TTAGGG)n tracts are shown as black arrows. Top panels: duplicated genomic segments are identified by chromosome (color) and whether they are subtelomeric (bounded rectangles), non-subtelomeric (unbounded rectangles), or intra-chromosomal (located above the subtelomere coordinates). Each rectangle represents a separate duplicon. Bottom panels: duplicated genomic segments are the same as in the top panels, but identified by nucleotide sequence similarity with the query subtelomere sequence (color scheme as indicated in the key). [file gb-2007-8-7-r151-S46.pdf]

Yq

0 kb Gap

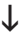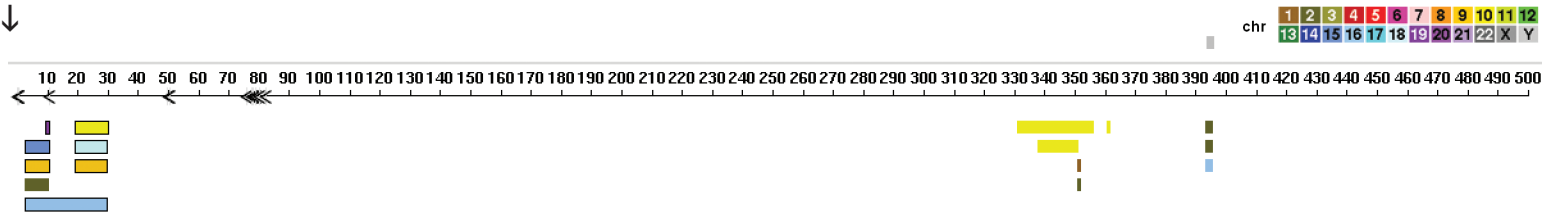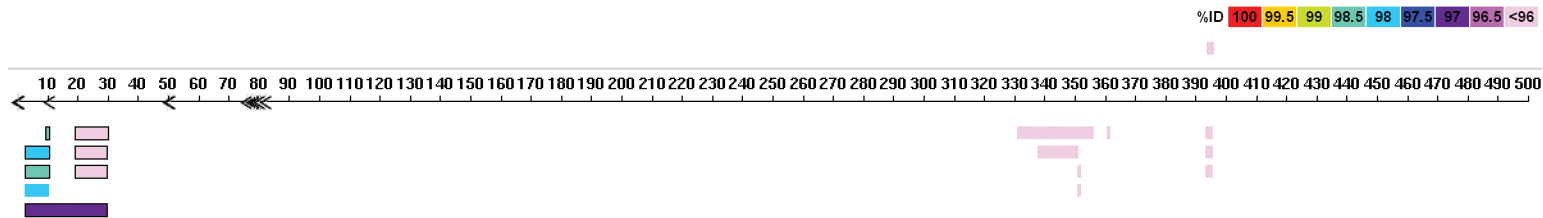

Supplement: Additional data file 47 — The subtelomere sequences shown are the assemblies published previously [6] and are available at the Riethman Lab website [47]. The telomeric end of each sequence assembly is located at the left. The distance from the end of the sequence to the start of the terminal repeat array is indicated by the vertical arrow at the telomeric end of the sequence. The position and orientation of (TTAGGG)n tracts are shown as black arrows. Top panels: duplicated genomic segments are identified by chromosome (color) and whether they are subtelomeric (bounded rectangles), non-subtelomeric (unbounded rectangles), or intra-chromosomal (located above the subtelomere coordinates). Each rectangle represents a separate duplicon. Bottom panels: duplicated genomic segments are the same as in the top panels, but identified by nucleotide sequence similarity with the query subtelomere sequence (color scheme as indicated in the key). [file gb-2007-8-7-r151-S47.pdf]
