# Supplementary material for: Human subtelomeric duplicon structure and organization
Source: Genome Biol. 2007 Jul 30;8(7):R151. doi: 10.1186/gb-2007-8-7-r151 (PMC2323237; doi:10.1186/gb-2007-8-7-r151)
Supplement: Additional data file 3 — Each module is defined by a set of pairwise alignments, and each reference sequence in these sets is represented as a single row in this table. The first column (module) contains an identifier for the particular copy of the module (duplicon) indicated in the next three columns. These columns (query sequence) list the subtelomeric location of the query sequence defining the module (see Materials and methods). The 'aligned sequences' column shows the locations of other duplicons in this module, matched by the query. The coordinates in this column refer either to our published subtelomeric assemblies (designated by chromosome and arm p or q) or the human genome build 35 (all other designations). The %IDeach is percent nucleotide sequence identity across the chained pairwise alignment, excluding masked sequence. The %IDavg is the average percent identity of all pairwise alignments in the module. This was the number used for %ID in charts and analyses in this paper. The final column shows a 1 if the module contains intrachromosomal non-subtelomeric sequence matches, and 0 if it does not. [file gb-2007-8-7-r151-S3.pdf]

module	query sequence			length	aligned sequences	$\%ID_{each}$	$\%ID_{avg}$	intra
1	1p	1	25292	25292	17q: 43948 - 19570	98.01	98.01	0
2	1p	1	43068	43068	5q: 58545 - 15084	98.93	98.93	0
3	1p	1	127509	127509	6q: 133267 - 9260	98.65	98.65	0
4	1p	26953	29950	2998	chr1: 239479432 - 239482412	97.27	97.27	1
5	1p	26953	43068	16116	chr4: 119705181 - 119722680	97.44	97.44	0
6	1p	26953	62722	35770	chrY: 25807359 - 25838662<br>chrY: 24800028 - 24831337	96.83<br>96.83	96.83	0
7	1p	26996	134371	107376	8p: 1 - 103892	98.69	98.69	0
8	1p	28586	29950	1365	chr1: 220383273 - 220384637	98.14	98.14	1
9	1p	30974	43068	12095	chr1: 220385665 - 220399178<br>chr1: 239483436 - 239496859	97.21<br>97.36	97.28	1
10	1p	44765	45785	1021	chr14: 32023058 - 32024075	93	93	0
11	1p	48942	58649	9708	chr4: 119695468 - 119705181	97.33	97.33	0
12	1p	48942	62722	13781	chr1: 220399178 - 220412991<br>chr1: 239496859 - 239510646	97.61<br>97.38	97.5	1
13	1p	48942	147924	98983	5q: 158314 - 58545	98.84	98.84	0
14	1p	52507	62722	10216	16q: 23961 - 13817	97.58	97.58	0
15	1p	54485	62722	8238	2q: 8258 - 107	97.46	97.46	0
16	1p	58653	62722	4070	chr4: 119944731 - 119948842	97.51	97.51	0
17	1p	70481	145957	75477	6p: 16681 - 87328	98.17	98.17	0
18	1p	70481	147924	77444	19p: 68278 - 145776	98.72	98.72	0
19	1p	70845	112213	41369	15q: 116787 - 75007	98	98	0
20	1p	71832	147924	76093	11p: 1 - 75496	98.67	98.67	0
21	1p	115941	141190	25250	chrY: 25847059 - 25878360<br>chrY: 24760333 - 24791629	95.87<br>95.89	95.88	0
22	1p	115941	144518	28578	7p: 2360 - 32053	98.39	98.39	0
23	1p	115941	146624	30684	20q: 46227 - 21029<br>chr10: 38769794 - 38800932<br>chr4: 119904851 - 119936314<br>chr1: 239518849 - 239549639	97.41<br>96.56<br>96.47<br>97.06	96.88	1
24	1p	116486	130948	14463	chr4: 165542477 - 165556842	95.61	95.61	0
25	1p	116486	136356	19871	2q: 37420 - 17207	96.97	96.97	0
26	1p	116486	139987	23502	chr1: 220421937 - 220445916	97.25	97.25	1
27	1p	116486	147924	31439	16q: 66298 - 32910	98.35	98.35	0
28	1p	126107	128002	1896	chr1: 224457879 - 224459772	97.23	97.23	1
29	1p	126808	147924	21117	3q: 21199 - 1	98.99	98.99	0
30	1p	128449	141190	12742	chr1: 224467506 - 224480262	96.89	96.89	1
31	1p	131997	140893	8897	chr7: 55985248 - 55994150	94.68	94.68	0
32	1p	132000	134020	2021	chr7: 61805021 - 61806480<br>chr1: 193837523 - 193839718<br>chr7: 55420846 - 55423188	92.69<br>92.72<br>92.81	92.74	1
33	1p	132020	138740	6721	chr7: 45414266 - 45420987	94.61	94.61	0
34	1p	135982	146847	10866	chr7: 127667204 - 127677946	96.65	96.65	0
35	1p	136001	138740	2740	chr7: 51009103 - 51011866<br>chr7: 55363599 - 55366358<br>chr7: 56432157 - 56434907<br>chr7: 39393644 - 39396387<br>chr7: 62457999 - 62460749<br>chr1: 219029846 - 219032485	93.00<br>94.00<br>94.00<br>93.00<br>94.00<br>97.00	94.17	1
36	1p	137311	146847	9537	chr4: 120680507 - 120690600	95.39	95.39	0
37	1p	138830	140589	1760	chr7: 56434997 - 56436149<br>chr1: 219032574 - 219033728<br>chr7: 55366448 - 55368463<br>chr7: 62455575 - 62457909<br>chr7: 51011956 - 51014002<br>chr7: 39391514 - 39393554	94.56<br>96.97<br>94.72<br>94.37<br>94.85<br>95.38	95.14	1
38	1p	138830	145546	6717	chr7: 45403637 - 45414176	95.05	95.05	0
39	1p	140895	145546	4652	chr1: 224460233 - 224466670	94.41	94.41	1
40	1p	144251	145546	1296	chr7: 51020104 - 51021398	95.75	95.75	0

**41**	1p	144252	146847	2596	chr7: 55375429 - 55378337<br>chr7: 56005902 - 56008498<br>chr7: 39386597 - 39389187<br>chr1: 219039323 - 219041921<br>chr1: 220447184 - 220449733	96.62<br>96.56<br>95.95<br>96.63<br>98.06	96.76	1
**42**	1p	144957	147924	2968	7p: 32493 - 35468	97.72	97.72	0
**43**	1p	147657	148954	1298	chr7: 45403637 - 45404932<br>chr1: 224460233 - 224461546	96.38<br>95.07	95.72	1
**44**	1p	147728	150225	2498	chr7: 55375429 - 55378283<br>chr7: 56005902 - 56008474<br>chr7: 39386621 - 39389187<br>chr7: 127667228 - 127669796<br>chr10: 38769818 - 38772390<br>chr4: 120688005 - 120690576<br>chr4: 119904875 - 119907443<br>chr1: 219039323 - 219041897<br>chr1: 239547046 - 239549615<br>chr1: 220447854 - 220449709	96.48<br>95.95<br>95.26<br>97.58<br>95.83<br>95.67<br>95.36<br>96.03<br>97.33<br>97.81	96.33	1
**45**	1p	147895	151331	3437	11p: 71805 - 75496<br>5q: 158314 - 154639<br>3q: 21199 - 17517<br>16q: 66298 - 62611<br>7p: 32499 - 35468<br>19p: 142809 - 145776	97.75<br>98.91<br>98.03<br>97.95<br>97.41<br>98.03	98.01	0
**46**	1p	151063	152515	1453	chr7: 45403637 - 45404932<br>chr10: 38771094 - 38772390<br>chr4: 119906144 - 119907443<br>chr1: 224460233 - 224461546<br>chr7: 39387491 - 39389187<br>chr7: 127668095 - 127669796<br>5q: 156343 - 154639<br>chr7: 51020104 - 51021398	96.00<br>95.59<br>95.57<br>94.70<br>95.44<br>97.99<br>98.58<br>94.95	96.1	1
**47**	1p	151063	191186	40124	7p: 31783 - 71832<br>11p: 71805 - 111964<br>19p: 142094 - 182218	98.30<br>98.43<br>98.37	98.37	0
**48**	1p	151065	153642	2578	chr7: 55375429 - 55378283<br>16q: 65198 - 62611<br>chr7: 56005902 - 56008476<br>3q: 20099 - 17517<br>chr4: 120688005 - 120690578<br>chr1: 219039323 - 219041899<br>chr1: 239547046 - 239549617<br>chr1: 220447184 - 220449711	96.28<br>97.48<br>95.93<br>98.01<br>95.84<br>96.12<br>97.13<br>97.77	96.82	1
**49**	1p	153070	185365	32296	5q: 189359 - 156637	98.4	98.4	0
**50**	1p	154479	171037	16559	chr7: 56005902 - 56027313	92.97	92.97	0
**51**	1p	154479	188802	34324	chr1: 219039323 - 219081989	97.3	97.3	1
**52**	1p	154479	191186	36708	3q: 57717 - 20931<br>16q: 102624 - 66030	98.44<br>98.38	98.41	0
**53**	1p	154479	192623	38145	chr1: 239547046 - 239591053	97.3	97.3	1
**54**	1p	154479	198083	43605	chr4: 120688005 - 120742610	96.55	96.55	0
**55**	1p	154526	157234	2709	chr7: 55375429 - 55383906<br>chr7: 56015725 - 56026447	93.85<br>90.67	92.26	0
**56**	1p	156853	197632	40780	chr10: 38723054 - 38764372	96.98	96.98	0
**57**	1p	156858	173095	16238	chr7: 127645453 - 127661758	96.14	96.14	0
**58**	1p	156858	178230	21373	chr7: 39360178 - 39381110	93	93	0
**59**	1p	156858	182943	26086	chr1: 220455168 - 220481396	97.88	97.88	1
**60**	1p	156858	192623	35766	chr4: 119869229 - 119899413	97.49	97.49	0
**61**	1p	156882	161096	4215	chr7: 45392682 - 45396890	96.06	96.06	0
**62**	1p	158227	163866	5640	chr11: 49997861 - 50003428<br>chr7: 56475714 - 56481294<br>chr7: 62412186 - 62417768<br>chr7: 64516409 - 64521893<br>chr7: 51029490 - 51036010	90.00<br>90.88<br>90.88<br>91.16<br>92.62	91.11	0
**63**	1p	158357	161096	2740	chr7: 55385504 - 55388239	94.41	94.41	0
**64**	1p	160844	170174	9331	chr7: 65587537 - 65593232	91.45	91.45	0

65	1p	164330	169875	5546	chr7: 64511343 - 64515807	91.43	91.43	0
66	1p	169656	191186	21531	9q: 22260 - 834	98.29	98.29	0
67	1p	169940	198083	28144	chr7: 45352592 - 45389874	96.19	96.19	0
68	1p	172348	173830	1483	chr7: 64507178 - 64508843	92.35	92.35	0
69	1p	172832	178286	5455	chr7: 56029594 - 56035175	93.01	93.01	0
70	1p	179165	180280	1116	chr7: 56036051 - 56037182	92.82	92.82	0
71	1p	180241	188802	8562	chr7: 127636388 - 127644996	95.34	95.34	0
72	1p	182809	184294	1486	chr7: 75222310 - 75223815<br>chr7: 65540948 - 65542445	92.76<br>94.07	93.41	0
73	1p	185854	187122	1269	chr7: 64469009 - 64470133<br>chr7: 63774859 - 63776045	90.32<br>90.41	90.36	0
74	1p	187408	198083	10676	chr1: 220481705 - 220501430	97.38	97.38	1
75	1p	190744	196652	5909	chr11: 50204185 - 50210330	92.36	92.36	0
76	1p	194962	197534	2573	chr7: 62184405 - 62186779<br>chr7: 56736665 - 56739034<br>chr7: 61936467 - 61938837<br>chr7: 35460461 - 35474160	92.15<br>92.15<br>92.11<br>91.99	92.1	0
77	1p	205659	213212	7554	chr1: 220510992 - 220518541	97.91	97.91	1
78	1p	205679	229635	23957	8p: 329082 - 353117	96.52	96.52	0
79	1p	213342	229635	16294	chr1: 220518672 - 220534939	97.99	97.99	1
80	1p	218996	229635	10640	2q: 48198 - 37437	97.96	97.96	0
81	1p	231380	233742	2363	8p: 279211 - 281570	97.7	97.7	0
82	1p	238096	242735	4640	8p: 134360 - 138988	98.07	98.07	0
83	1p	241682	242735	1054	6q: 134337 - 133279	98.36	98.36	0
84	1p	245037	280290	35254	8p: 194471 - 231807	97.08	97.08	0
85	1p	281460	283440	1981	21q: 19448 - 17648<br>18p: 91016 - 93277<br>10q: 19834 - 17578<br>22q: 18168 - 15901<br>4p: 14074 - 15591<br>4q: 7234 - 5717<br>19q: 15185 - 12919<br>16q: 6877 - 4620<br>chr2: 114093391 - 114095655<br>6p: 91533 - 93673<br>1q: 11022 - 8975<br>7p: 71845 - 73566<br>11p: 111977 - 113702<br>3q: 59455 - 57730<br>9q: 23994 - 22273<br>19p: 182231 - 183954<br>16q: 104358 - 102637<br>8p: 105117 - 106839<br>2p: 3251 - 5049	95.00<br>95.18<br>94.52<br>95.67<br>93.36<br>93.26<br>96.00<br>95.78<br>95.93<br>92.75<br>93.70<br>96.51<br>97.19<br>97.19<br>96.51<br>97.10<br>96.51<br>96.78<br>90.72	95.25	1
86	1p	284104	286515	2412	18p: 1669 - 6845<br>chr1: 224030580 - 224037287<br>10p: 125 - 2430<br>chr2: 132394854 - 132397167<br>chr22: 14844304 - 14846620<br>chr14: 18425318 - 18427632<br>chr12: 34147807 - 34150020	93.41<br>91.12<br>93.81<br>93.77<br>93.77<br>93.86<br>91.88	93.09	1
87	1p	287192	293392	6201	4q: 127186 - 121253	93.17	93.17	0
88	1p	287195	288715	1521	16q: 133323 - 131398<br>chr12: 36874324 - 36875442	93.18<br>90.00	91.59	0
89	1p	287201	301425	14225	18p: 17567 - 38559<br>10p: 13173 - 34166<br>9q: 82885 - 65244<br>3q: 115912 - 100958	93.46<br>93.46<br>93.78<br>93.84	93.64	0
90	1p	288354	296670	8317	chr12: 34182787 - 34200623	90.42	90.42	0
91	1p	293260	295734	2475	chr1: 238554207 - 238558412	91.14	91.14	1
92	1p	293274	304945	11672	chrY: 10561453 - 10577074	92.96	92.96	0
93	1p	295513	296696	1184	chrY: 18946969 - 18958077<br>chrY: 18047953 - 18059059	91.00<br>91.00	91	0
94	1p	301880	304623	2744	chr1: 5649685 - 5661440	93.19	93.19	1
95	1p	305118	309301	4184	chr3: 75933584 - 75937820	95.04	95.04	0

96	1p	357462	373002	15541	chr2: 130701507 - 130704726	94.88	94.88	0
97	1p	357462	373273	15812	chr2: 132032476 - 132035943	93.73	93.73	0
98	1q	1764	12469	10706	10q: 21274 - 8629<br>22q: 19698 - 6932<br>chr2: 114084410 - 114097183<br>19q: 16715 - 3954	93.99<br>94.40<br>94.47<br>94.69	94.39	0
99	1q	1764	10576	8813	21q: 19448 - 8673	95.16	95.16	0
100	1q	1764	4813	3050	chr3: 75756447 - 75760281	94.34	94.34	0
101	1q	1786	8975	7190	5q: 11967 - 5746<br>17q: 16452 - 8455<br>8p: 116333 - 122565<br>6q: 6141 - 1	94.87<br>94.81<br>93.30<br>95.01	94.5	0
102	1q	4691	9184	4494	19p: 193602 - 199397	94.71	94.71	0
103	1q	4691	7881	3191	6q: 147506 - 145675	90.83	90.83	0
104	1q	4692	15422	10731	4p: 7723 - 22772<br>13q: 5935 - 1401	92.86<br>91.64	92.25	0
105	1q	7033	15422	8390	5p: 617 - 8075<br>4q: 11634 - 2781	93.67<br>92.09	92.88	0
106	1q	7394	12244	4851	16q: 7774 - 1705<br>18p: 88797 - 95146	93.09<br>92.65	92.87	0
107	1q	8975	28891	19917	6p: 91611 - 105620	94.06	94.06	0
108	1q	9143	11119	1977	1p: 281407 - 283465<br>16q: 104535 - 102637	93.50<br>93.47	93.48	1
109	1q	9311	12434	3124	19p: 182231 - 185064<br>11p: 111977 - 115229<br>7p: 71845 - 75097<br>9q: 25525 - 22273<br>3q: 60982 - 57730<br>8p: 105117 - 107957	93.68<br>93.08<br>93.29<br>93.19<br>93.25<br>93.49	93.33	0
110	1q	9569	15422	5854	2p: 3280 - 10453	91.63	91.63	0
111	1q	165119	167249	2131	chr8: 95806410 - 95869973	91.24	91.24	0
112	1q	283789	318216	34428	chr1: 244914453 - 244949099	97.7	97.7	1
113	2p	2614	23214	20601	13q: 15153 - 1374	91.5	91.5	0
114	2p	2615	7987	5373	4p: 7699 - 18608	91.93	91.93	0
115	2p	2635	17320	14686	21q: 20521 - 12379	93.36	93.36	0
116	2p	2945	10453	7509	1q: 15422 - 4691<br>6p: 92174 - 98867	91.72<br>91.39	91.56	0
117	2p	3123	6333	3211	10q: 21274 - 12303<br>19q: 16715 - 7670<br>22q: 19698 - 10660<br>16q: 104535 - 102867<br>19p: 182461 - 185064<br>8p: 105346 - 107957<br>11p: 112207 - 115229<br>3q: 60982 - 57960<br>9q: 25525 - 22503<br>7p: 72075 - 75097<br>18p: 91812 - 94731<br>chr2: 114094473 - 114097183	92.82<br>92.07<br>92.30<br>91.43<br>91.91<br>92.54<br>92.08<br>92.08<br>91.70<br>91.70<br>92.23<br>91.75	92.05	1
118	2p	3689	5486	1798	1p: 281971 - 283465<br>16q: 7770 - 5957	91.20<br>92.01	91.61	0
119	2p	4878	6584	1707	4q: 8797 - 7063	92.03	92.03	0
120	2p	6456	17877	11422	5p: 2293 - 16944	91.64	91.64	0
121	2p	9594	11886	2293	4q: 13056 - 10778	90.51	90.51	0
122	2p	9594	14710	5117	4p: 21916 - 27322	91.16	91.16	0
123	2p	89266	91971	2706	21q: 26137 - 23656	92.95	92.95	0
124	2p	155328	164148	8821	chr3: 197233860 - 197243557	93.02	93.02	0
125	2p	155328	168204	12877	5p: 18771 - 31689	92.68	92.68	0
126	2p	266803	268101	1299	chr1: 230277578 - 230278744	93.72	93.72	0
127	2q	79	37420	37342	16q: 52766 - 15778<br>chrY: 25830407 - 25873515<br>chrY: 24765178 - 24808289	97.75<br>96.29<br>96.31	96.78	0
128	2q	107	14732	14626	chr1: 239502401 - 239517107	96.81	96.81	0

129	2q	107	8258	8152	5q: 72329 - 64090<br>1p: 54485 - 62722<br>chr1: 220404716 - 220412991<br>6q: 64494 - 56258<br>8p: 21631 - 29862	97.81<br>97.60<br>97.80<br>97.81<br>97.74	97.75	0
130	2q	107	4174	4068	chr4: 119695468 - 119699622	97.41	97.41	0
131	2q	4178	37417	33240	chr4: 119915974 - 119948842	97.12	97.12	0
132	2q	9926	37420	27495	chr10: 38780554 - 38807668	96.91	96.91	0
133	2q	10787	20769	9983	chr1: 220415524 - 220425497	97.78	97.78	0
134	2q	11804	31737	19934	chr4: 165537042 - 165556804	95.95	95.95	0
135	2q	14584	37420	22837	7p: 91 - 22770<br>20q: 41420 - 18783<br>chr1: 239517108 - 239539374	97.58<br>97.61<br>97.38	97.52	0
136	2q	17207	37420	20214	11p: 43725 - 63564<br>5q: 146656 - 126807<br>19p: 113350 - 133490<br>6p: 62675 - 82522	97.62<br>97.53<br>97.46<br>97.23	97.46	0
137	2q	17207	35177	17971	8p: 86034 - 103892	97.23	97.23	0
138	2q	17207	28265	11059	6q: 133270 - 122211	97.75	97.75	0
139	2q	17207	20769	3563	1p: 116486 - 120046	97.72	97.72	0
140	2q	20913	37420	16508	chr1: 220425641 - 220442288	97.24	97.24	0
141	2q	20913	22092	1180	1p: 120190 - 121369	98.67	98.67	0
142	2q	25664	37420	11757	1p: 124946 - 136356	97.06	97.06	0
143	2q	26855	28809	1955	chr1: 224457827 - 224459772	96.88	96.88	0
144	2q	27564	37420	9857	3q: 9502 - 1	97.47	97.47	0
145	2q	29178	37420	8243	chr1: 224472349 - 224480262	97.08	97.08	0
146	2q	32817	34583	1767	chr7: 61805021 - 61806470<br>chr1: 193837523 - 193838976<br>chr7: 55420846 - 55423188	92.52<br>92.35<br>92.75	92.54	0
147	2q	32825	37420	4596	chr7: 55985248 - 55989644<br>chr7: 45416639 - 45420987	95.89<br>96.77	96.33	0
148	2q	37437	48245	10809	1p: 218996 - 229635<br>chr1: 220524327 - 220535034	98.27<br>97.74	98	0
149	2q	37438	91777	54340	8p: 290588 - 339694	96.28	96.28	0
150	2q	113376	130689	17314	chr8: 70400743 - 70415059	91.27	91.27	0
151	2q	123778	130109	6332	chr5: 1658875 - 1664417<br>5p: 274416 - 281490	91.30<br>92.02	91.66	0
152	2q	127244	130638	3395	chr3: 196868578 - 196872736	92.6	92.6	0
153	2q	127253	129369	2117	chr3: 197206782 - 197208724	92.28	92.28	0
154	2q	127259	137427	10169	chr3: 198844371 - 198861538	92.86	92.86	0
155	2q	129544	130680	1137	chr9: 136942169 - 136943924	92.33	92.33	0
156	2q	130470	138715	8246	5p: 262223 - 274318<br>chr5: 1665247 - 1678396	92.81<br>94.03	93.42	0
157	2q	130535	139193	8659	chr3: 197151348 - 197162783	92.22	92.22	0
158	3p	25836	27430	1595	chr3: 75358906 - 75362529	95.58	95.58	1
159	3p	27552	30982	3431	chr3: 75362651 - 75370581	93.46	93.46	1
160	3p	31906	33277	1372	chr3: 75371501 - 75372877	96.22	96.22	1
161	3p	33334	36566	3233	chr3: 75372933 - 75376158	95.02	95.02	1
162	3p	37417	66238	28822	chr3: 75377008 - 75411740	95.3	95.3	1
163	3q	1	87720	87720	16q: 131364 - 43247<br>7p: 13255 - 102116	98.41<br>98.45	98.43	0
164	3q	1	75708	75708	11p: 54057 - 128459	98.67	98.67	0
165	3q	1	60961	60961	19p: 123991 - 185461	98.73	98.73	0
166	3q	1	51879	51879	5q: 189359 - 137159	98.61	98.61	0
167	3q	1	21199	21199	1p: 126808 - 147924	98.99	98.99	0
168	3q	1	19763	19763	6p: 72998 - 87328<br>20q: 46227 - 31895<br>chr1: 239529853 - 239549639<br>chr10: 38769794 - 38790071<br>chr4: 119904851 - 119925461	97.91<br>97.84<br>97.19<br>96.82<br>97.02	97.36	0
169	3q	1	14339	14339	chrY: 24760333 - 24774734<br>chrY: 25863958 - 25878360	95.96<br>95.94	95.95	0
170	3q	1	13137	13137	chr1: 220432757 - 220445916	97.49	97.49	0
171	3q	1	9502	9502	2q: 37420 - 27564	97.1	97.1	0
172	3q	1	7525	7525	8p: 96353 - 103892	98.57	98.57	0

173	3q	1	1249	1249	chr1: 224457827 - 224459071	98	98	0
174	3q	4	3716	3713	chr4: 165552704 - 165556462	95.58	95.58	0
175	3q	1594	14339	12746	chr1: 224467506 - 224480262	96.85	96.85	0
176	3q	5148	14042	8895	chr7: 55985248 - 55994150	94.51	94.51	0
177	3q	5151	7171	2021	chr7: 61805021 - 61806472<br>chr1: 193837523 - 193839718<br>chr7: 55420846 - 55423188	91.84<br>92.63<br>92.44	92.3	0
178	3q	5171	11886	6716	chr7: 45414266 - 45420987	94.61	94.61	0
179	3q	9134	20121	10988	chr7: 127667204 - 127677946	96.68	96.68	0
180	3q	9153	11886	2734	chr7: 51009103 - 51011866<br>chr7: 39393644 - 39396387<br>chr7: 62457999 - 62460749<br>chr7: 55363599 - 55366358<br>chr7: 56432157 - 56434907<br>chr1: 219029846 - 219032485	93.00<br>93.00<br>94.00<br>94.00<br>94.00<br>97.00	94.17	0
181	3q	10457	20121	9665	chr4: 120680507 - 120690600	94.96	94.96	0
182	3q	11976	18815	6840	chr7: 45403637 - 45414176	95.23	95.23	0
183	3q	11976	13736	1761	chr1: 219032574 - 219033728<br>chr7: 56434997 - 56436149<br>chr7: 62455590 - 62457909<br>chr7: 55366448 - 55368463<br>chr7: 39391514 - 39393554<br>chr7: 51011956 - 51014002	96.86<br>94.15<br>94.45<br>94.36<br>94.91<br>94.13	94.81	0
184	3q	14044	18815	4772	chr1: 224460233 - 224466670	94.42	94.42	0
185	3q	17517	25043	7527	1p: 151063 - 158591	98.72	98.72	0
186	3q	17517	21199	3683	1p: 147657 - 151331	98.39	98.39	0
187	3q	17520	18815	1296	chr7: 51020104 - 51021398	95.53	95.53	0
188	3q	17521	20121	2601	chr1: 219039323 - 219041921<br>chr7: 39386597 - 39389187<br>chr7: 55375429 - 55378337<br>chr7: 56005902 - 56008498<br>chr1: 220447184 - 220449733	96.72<br>96.16<br>96.61<br>96.65<br>98.20	96.87	0
189	3q	20931	57717	36787	chr1: 239547046 - 239589627<br>chr4: 120688005 - 120730560	97.29<br>96.44	96.87	0
190	3q	20931	55324	34394	chr1: 219039323 - 219081989	97.25	97.25	0
191	3q	20931	37501	16571	chr7: 56005902 - 56027313	92.93	92.93	0
192	3q	20978	23681	2704	chr7: 55375429 - 55383896<br>chr7: 56015725 - 56026447	94.49<br>90.68	92.59	0
193	3q	23307	57717	34411	chr10: 38729667 - 38764372<br>chr4: 119870679 - 119899413	97.09<br>97.41	97.25	0
194	3q	23310	49429	26120	chr1: 220455168 - 220481396	97.65	97.65	0
195	3q	23310	44682	21373	chr7: 39360178 - 39381110	92.94	92.94	0
196	3q	23310	39561	16252	chr7: 127645453 - 127661758	95.71	95.71	0
197	3q	23334	27562	4229	chr7: 45392682 - 45396890	95.91	95.91	0
198	3q	23597	30181	6585	chr7: 62412186 - 62418873	91.35	91.35	0
199	3q	24680	30560	5881	chr7: 56475714 - 56481294<br>chr7: 51029490 - 51036010	91.43<br>93.18	92.31	0
200	3q	24809	27562	2754	chr7: 55385504 - 55388239	93.96	93.96	0
201	3q	26086	27562	1477	chr11: 49999260 - 50000805	91.6	91.6	0
202	3q	27310	57717	30408	1p: 160844 - 191186	98.37	98.37	0
203	3q	27310	36336	9027	chr7: 64511343 - 64519292	91.14	91.14	0
204	3q	27310	30650	3341	chr7: 65590242 - 65593232	90.87	90.87	0
205	3q	36117	119439	83323	9q: 88449 - 834	97.8	97.8	0
206	3q	36123	57717	21595	chr7: 45368447 - 45390152	96.2	96.2	0
207	3q	38814	40276	1463	chr7: 64507178 - 64508843	92.35	92.35	0
208	3q	39298	44738	5441	chr7: 56029594 - 56035175	92.72	92.72	0
209	3q	45629	46769	1141	chr7: 56036051 - 56037182	92.73	92.73	0
210	3q	46730	55324	8595	chr7: 127636388 - 127644996	95.24	95.24	0
211	3q	49295	50780	1486	chr7: 75222310 - 75223815<br>chr7: 65540950 - 65542445	92.68<br>94.03	93.36	0
212	3q	51708	57717	6010	chr11: 50209890 - 50211058	90	90	0
213	3q	52275	53636	1362	chr7: 63774859 - 63776250<br>chr7: 64469009 - 64470133	90.56<br>90.32	90.44	0
214	3q	53922	57717	3796	chr1: 220481705 - 220485566	97.32	97.32	0

**215**	3q	57706	60967	3262	8p: 104622 - 108356<br>10q: 21671 - 18113<br>6p: 91944 - 95168<br>22q: 19684 - 16443<br>1q: 12559 - 9311<br>18p: 91554 - 95132<br>19q: 16701 - 13461<br>chr2: 114093933 - 114097169<br>4q: 8783 - 6261<br>4p: 14618 - 17140<br>2p: 3251 - 6570	97.39<br>93.48<br>92.86<br>97.31<br>92.80<br>94.48<br>97.01<br>97.05<br>93.49<br>93.52<br>91.59	94.63	0
**216**	3q	57730	59360	1631	1p: 281741 - 283465<br>16q: 7077 - 5156<br>21q: 19448 - 18188	96.81<br>95.38<br>96.13	96.11	0
**217**	3q	65850	73851	8002	17p: 727 - 18220	96.81	96.81	0
**218**	3q	87850	99280	11431	7p: 130045 - 141501	97.89	97.89	0
**219**	3q	99386	119228	19843	10p: 10952 - 39687<br>18p: 15346 - 44090<br>chr12: 34168714 - 34210423	95.14<br>95.27<br>91.27	93.89	0
**220**	3q	100958	115912	14955	1p: 287230 - 301429	94.14	94.14	0
**221**	3q	105654	118792	13139	chrY: 18047953 - 18076360	91.18	91.18	0
**222**	3q	105657	106765	1109	chr1: 243986733 - 243996864<br>chrY: 10567257 - 10568441	90.65<br>93.00	91.83	0
**223**	3q	110861	119065	8205	4q: 126874 - 109517<br>chrY: 18929676 - 18942635	93.92<br>91.05	92.48	0
**224**	3q	114461	119437	4977	16q: 138855 - 131467<br>chr12: 36865089 - 36875442	94.72<br>90.77	92.75	0
**225**	3q	117716	119438	1723	chr3: 75834770 - 75837024<br>chrY: 10552604 - 10553871	91.97<br>90.65	91.31	1
**226**	3q	124163	126679	2517	10q: 85668 - 83221<br>4q: 78877 - 76430	91.20<br>90.87	91.03	0
**227**	3q	124173	125380	1208	chr3: 75796196 - 75797387	91.68	91.68	1
**228**	3q	127585	140222	12638	chr5: 84158536 - 84166018	92.06	92.06	0
**229**	3q	140414	141426	1013	chr5: 84204349 - 84205411	91.68	91.68	0
**230**	3q	143348	146483	3136	chr5: 84253750 - 84256617	92.22	92.22	0
**231**	3q	155105	173470	18366	chr8: 43220224 - 43226455	91.37	91.37	0
**232**	3q	155996	183239	27244	chr21: 14220320 - 14273129	91.67	91.67	0
**233**	3q	155996	157246	1251	chr18: 14170148 - 14171404	91.3	91.3	0
**234**	3q	156747	159024	2278	chr9: 33512095 - 33515657<br>chr9: 38601554 - 38605067	91.47<br>90.48	90.97	0
**235**	3q	158691	167637	8947	chr9: 92652033 - 92662431	91.47	91.47	0
**236**	3q	158691	164706	6016	chr9: 97030524 - 97042993	91.02	91.02	0
**237**	3q	161678	163399	1722	chr9: 38595799 - 38598726<br>chr9: 33518744 - 33521425	90.77<br>90.84	90.81	0
**238**	3q	164741	170471	5731	chr9: 38588238 - 38594721<br>chr9: 97025319 - 97030101	91.44<br>91.89	91.66	0
**239**	3q	167479	178921	11443	chr9: 33525081 - 33536584	92.62	92.62	0
**240**	3q	170746	183256	12511	chr9: 97009645 - 97024576	91.26	91.26	0
**241**	3q	177510	179229	1720	chr9: 38579888 - 38581288	90.7	90.7	0
**242**	3q	179254	183237	3984	chr13: 18299772 - 18305947<br>chr2: 94893857 - 94900008<br>chr18: 14220855 - 14223626	91.94<br>90.79<br>92.65	91.79	0
**243**	3q	180892	183256	2365	chr9: 38573399 - 38575161<br>chr9: 33541649 - 33543410	91.29<br>91.29	91.29	0
**244**	3q	216508	252173	35666	chr9: 94333705 - 94366610	91.41	91.41	0
**245**	3q	281413	284402	2990	chr8: 122460261 - 122460556<br>chr12: 32898960 - 32899254<br>chr17: 24682666 - 24682959<br>chr16: 12874563 - 12874862<br>chr1: 200536390 - 200536689<br>chr12: 48534873 - 48535173<br>chr9: 71430302 - 71430601<br>chr22: 42677817 - 42678104	92.49<br>90.00<br>91.51<br>93.52<br>91.51<br>91.04<br>91.52<br>93.22	91.85	0
**246**	3q	281435	284404	2970	chr16: 67060724 - 67061002	91.56	91.56	0
**247**	4p	7699	12004	4306	6q: 147519 - 145651	90.58	90.58	0

**248**	4p	7699	27342	19644	13q: 10484 - 1375	91.42	91.42	0
**249**	4p	7714	17154	9441	10q: 21689 - 12304<br>chr2: 114088128 - 114097183<br>19q: 16715 - 7670<br>22q: 19698 - 10660	90.67<br>91.96<br>92.12<br>91.96	91.68	0
**250**	4p	7715	14458	6744	19p: 193578 - 199396<br>6q: 7195 - 1847<br>5q: 13021 - 7682<br>17q: 17506 - 12166<br>21q: 18851 - 12379	91.27<br>91.98<br>91.49<br>91.49<br>91.13	91.47	0
**251**	4p	7723	22772	15050	1q: 15422 - 4691	92.54	92.54	0
**252**	4p	11125	24198	13074	4q: 13060 - 2763	98.46	98.46	1
**253**	4p	11143	13573	2431	8p: 120622 - 123046	91	91	0
**254**	4p	11143	27342	16200	5p: 622 - 13652	92.52	92.52	0
**255**	4p	11500	16821	5322	16q: 7774 - 1699<br>18p: 88797 - 95146	91.88<br>92.65	92.27	0
**256**	4p	14202	22772	8571	6p: 91533 - 98867	92.79	92.79	0
**257**	4p	14346	15689	1344	1p: 281205 - 283465<br>16q: 104535 - 102639	91.78<br>92.95	92.37	0
**258**	4p	14618	17154	2537	9q: 25525 - 22275<br>7p: 71847 - 75097<br>8p: 105118 - 108370<br>11p: 111979 - 115229<br>3q: 60982 - 57732<br>19p: 182233 - 185482	93.64<br>93.64<br>91.76<br>93.06<br>92.60<br>93.14	92.97	0
**259**	4p	15154	27322	12169	2p: 3558 - 14710	91	91	0
**260**	4p	185032	188601	3570	chr11: 9553495 - 9560690	91.54	91.54	0
**261**	4p	191448	203036	11589	chr18: 14113043 - 14124512	91	91	0
**262**	4p	282780	294432	11653	chr21: 14317809 - 14326816<br>chr18: 14117071 - 14124599	90.80<br>91.06	90.93	0
**263**	4p	408846	411665	2820	chr17: 64646851 - 64681546	93.19	93.19	0
**264**	4q	2771	13058	10288	4p: 11123 - 24198<br>5p: 622 - 9438	98.46<br>91.91	95.19	1
**265**	4q	2775	5760	2986	5q: 13021 - 10020<br>6q: 7195 - 4191<br>17q: 17506 - 14505<br>8p: 120622 - 123046<br>19p: 196024 - 199396	91.26<br>91.79<br>91.26<br>91.36<br>91.44	91.42	0
**266**	4q	2776	7177	4402	16q: 7077 - 1699<br>21q: 18851 - 14729	91.61<br>90.86	91.23	0
**267**	4q	2781	36107	33327	10q: 46814 - 14674	97.27	97.27	0
**268**	4q	2781	11634	8854	1q: 15422 - 7036	91.7	91.7	0
**269**	4q	2781	8797	6017	22q: 19698 - 13010<br>19q: 16715 - 10020<br>chr2: 114090478 - 114097183	91.36<br>92.12<br>91.86	91.78	0
**270**	4q	3505	15653	12149	18p: 88797 - 102436	93.31	93.31	0
**271**	4q	5717	7234	1518	1p: 281205 - 283465	91.69	91.69	0
**272**	4q	5845	11634	5790	6p: 91533 - 98867	92.2	92.2	0
**273**	4q	6261	8797	2537	16q: 105889 - 102639<br>7p: 71847 - 75097<br>9q: 25525 - 22275<br>19p: 182233 - 185482<br>3q: 60982 - 57732<br>8p: 105118 - 108370<br>11p: 111979 - 115229	93.69<br>93.69<br>93.69<br>93.25<br>92.46<br>91.79<br>93.10	93.1	0
**274**	4q	6797	13056	6260	2p: 3558 - 11886	90.96	90.96	0
**275**	4q	8663	11716	3054	13q: 6017 - 2102	92.49	92.49	0
**276**	4q	15442	32814	17373	10q: 46814 - 29390	98.99	98.99	0
**277**	4q	15442	29521	14080	10q: 46814 - 32700	98.96	98.96	0
**278**	4q	15442	26221	10780	10q: 46814 - 35999	99	99	0
**279**	4q	15442	22927	7486	10q: 46814 - 39308	98.93	98.93	0
**280**	4q	15442	19634	4193	10q: 46814 - 42618	98.8	98.8	0
**281**	4q	18742	39404	20663	10q: 46814 - 26091	98.95	98.95	0
**282**	4q	22035	39686	17652	10q: 43786 - 26091	98.93	98.93	0
**283**	4q	25335	39686	14352	10q: 40476 - 26091	98.9	98.9	0

284	4q	28629	39682	11054	10q: 37163 - 26091	98.89	98.89	0
285	4q	31922	39686	7765	10q: 33868 - 26091	98.81	98.81	0
286	4q	35215	39686	4472	10q: 30558 - 26091	98.61	98.61	0
287	4q	38512	39686	1175	10q: 27259 - 26091	98.29	98.29	0
288	4q	39686	81906	42221	10q: 88677 - 46814	98.37	98.37	0
289	4q	43533	85810	42278	chr3: 75761698 - 75804455	95.11	95.11	0
290	4q	82188	83378	1191	chrY: 10602384 - 10605587<br>10q: 44069 - 43013<br>10q: 40759 - 39703<br>10q: 37450 - 36394<br>10q: 34151 - 33095<br>10q: 30841 - 29785<br>10q: 27542 - 26486	90.00<br>98.29<br>98.29<br>97.86<br>98.29<br>98.29<br>98.29	97.04	0
291	4q	84408	89657	5250	chr9: 64132151 - 64135635	90.86	90.86	0
292	4q	88909	109738	20830	chr3: 75803938 - 75835090	92.65	92.65	0
293	4q	100581	111447	10867	chr12: 34209036 - 34230448	91.45	91.45	0
294	4q	109513	126874	17362	10p: 28824 - 39586<br>18p: 33215 - 43990<br>9q: 88348 - 77401	93.36<br>93.53<br>94.03	93.64	0
295	4q	109513	123167	13655	16q: 138755 - 131398	94.73	94.73	0
296	4q	109517	111410	1894	chr12: 36865186 - 36867121<br>3q: 119338 - 117506<br>chrY: 10552713 - 10554516	91.56<br>90.98<br>91.23	91.26	0
297	4q	121272	127030	5759	1p: 287192 - 293392<br>3q: 115912 - 110514	93.27<br>94.15	93.71	0
298	4q	125569	127186	1618	chr12: 34196193 - 34197802	91.18	91.18	0
299	4q	129786	203410	73625	chr20: 28193700 - 28254858	93.54	93.54	0
300	4q	148465	228836	80372	chr9: 66813927 - 66892012<br>chr9: 67052957 - 67131004	93.61<br>93.60	93.6	0
301	4q	389382	391241	1860	chr16: 33865962 - 33867849	91.03	91.03	0
302	4q	389382	391239	1858	chr2: 132840626 - 132842508	90.64	90.64	0
303	5p	617	4451	3835	1q: 13870 - 7031	94.43	94.43	0
304	5p	617	17258	16642	21q: 21380 - 14724	92.48	92.48	0
305	5p	622	9438	8817	4q: 13056 - 2781	92.51	92.51	0
306	5p	622	13652	13031	4p: 11143 - 27342	92.68	92.68	0
307	5p	683	2362	1680	19p: 196019 - 197721<br>10q: 21689 - 14669<br>chr2: 114090473 - 114097185<br>8p: 120617 - 123564<br>19q: 16717 - 10015<br>22q: 19700 - 13005<br>6q: 5868 - 4197<br>16q: 3379 - 1705<br>5q: 11694 - 10026<br>17q: 16179 - 14511<br>18p: 88797 - 95146	93.86<br>93.28<br>93.36<br>95.03<br>93.63<br>93.46<br>94.70<br>94.00<br>93.30<br>93.30<br>91.99	93.63	1
308	5p	2293	8075	5783	6p: 95055 - 98867	93.71	93.71	0
309	5p	2293	14897	12605	13q: 11739 - 2108	91.61	91.61	0
310	5p	2293	16995	14703	2p: 6456 - 17928	91.78	91.78	0
311	5p	16125	27646	11522	chr3: 197230504 - 197244078	93.45	93.45	0
312	5p	18771	31666	12896	2p: 155328 - 168181	92.29	92.29	0
313	5p	197391	246221	48831	chr5: 1620762 - 1652265	96.44	96.44	1
314	5p	200769	251030	50262	chr3: 196873665 - 196909300	93.42	93.42	0
315	5p	200769	269778	69010	chr3: 197154630 - 197205838	93.59	93.59	0
316	5p	200777	211326	10550	chr3: 198834782 - 198843420	93.61	93.61	0
317	5p	248964	262636	13673	chr5: 1677980 - 1687907<br>chr3: 198862190 - 198872062	95.57<br>93.90	94.73	1
318	5p	262223	269778	7556	chr3: 197214129 - 197221816	92.49	92.49	0
319	5p	262223	281457	19235	2q: 138714 - 123812	92.15	92.15	0
320	5p	263756	269778	6023	chr3: 198851711 - 198861497	93.82	93.82	0
321	5p	269995	281194	11200	chr8: 70399528 - 70407084	91.32	91.32	0
322	5p	270537	272685	2149	chr3: 73195843 - 73198706	94.51	94.51	0
323	5p	270974	288231	17258	chr5: 1653358 - 1668818	96.43	96.43	1

324	5p	271310	273942	2633	chr3: 197211300 - 197214109<br>chr3: 198848840 - 198851194<br>chr3: 197151399 - 197154125	94.26<br>94.66<br>94.13	94.35	0
325	5p	274733	278314	3582	chr3: 196868822 - 196872795<br>chr3: 198844298 - 198848270	93.39<br>93.82	93.6	0
326	5p	275691	278314	2624	chr3: 197206715 - 197208720	93.8	93.8	0
327	5q	1037	2646	1610	6q: 141080 - 139446	91.83	91.83	0
328	5q	1038	12824	11787	19p: 189683 - 199081<br>chr2: 114079703 - 114093488<br>8p: 112649 - 123045	95.90<br>95.72<br>94.91	95.51	0
329	5q	2247	41588	39342	17q: 43948 - 4319	98.67	98.67	0
330	5q	2247	13019	10773	22q: 16000 - 3342<br>10q: 17677 - 5039<br>21q: 17747 - 5548<br>19q: 13018 - 639	95.43<br>96.09<br>95.52<br>95.89	95.73	0
331	5q	5746	11967	6222	1q: 8975 - 1764	95.66	95.66	0
332	5q	5746	7808	2063	chr3: 75756447 - 75760281	93.52	93.52	0
333	5q	5836	137863	132028	6q: 133270 - 1	98.57	98.57	0
334	5q	7682	13021	5340	4p: 7719 - 14175	92.28	92.28	0
335	5q	10026	15432	5407	16q: 13032 - 1705	96.76	96.76	0
336	5q	10026	11694	1669	5p: 618 - 2291	94.7	94.7	1
337	5q	10390	13021	2632	4q: 5818 - 2781<br>18p: 88797 - 91117	92.10<br>93.44	92.77	0
338	5q	15084	130366	115283	1p: 1 - 120046	98.83	98.83	0
339	5q	42421	72329	29909	chrY: 24800028 - 24831337<br>chrY: 25807359 - 25838662	96.88<br>96.88	96.88	0
340	5q	42421	68260	25840	chr4: 119695468 - 119722680	97.48	97.48	0
341	5q	42421	45418	2998	chr1: 239479432 - 239482412	97.35	97.35	0
342	5q	42464	144676	102213	8p: 1 - 103892	98.77	98.77	0
343	5q	44054	45418	1365	chr1: 220383273 - 220384637	98.14	98.14	0
344	5q	46443	72329	25887	chr1: 220385665 - 220412991<br>chr1: 239483436 - 239510646	97.40<br>97.56	97.48	0
345	5q	62109	72329	10221	16q: 23961 - 13817	97.62	97.62	0
346	5q	64090	72329	8240	2q: 8258 - 107	97.67	97.67	0
347	5q	68264	72329	4066	chr4: 119944731 - 119948842	97.98	97.98	0
348	5q	80102	189359	109258	19p: 68278 - 176313	98.47	98.47	0
349	5q	80102	156343	76242	6p: 16681 - 87323	98.08	98.08	0
350	5q	80764	122536	41773	15q: 116787 - 75307	98.04	98.04	0
351	5q	81451	189359	107909	11p: 1 - 106130	98.43	98.43	0
352	5q	126263	156996	30734	20q: 46222 - 21028<br>chr1: 239518848 - 239549617<br>chr10: 38769816 - 38800933<br>chr4: 119904873 - 119936314	97.56<br>97.15<br>96.55<br>96.88	97.03	0
353	5q	126264	151484	25221	chrY: 24760333 - 24791629<br>chrY: 25847059 - 25878360	96.08<br>95.99	96.03	0
354	5q	126535	189359	62825	7p: 2359 - 66000<br>16q: 96795 - 32910	98.45<br>98.42	98.44	0
355	5q	126807	146656	19850	2q: 37420 - 17207	97.3	97.3	0
356	5q	126807	140872	14066	chr4: 165542477 - 165556462	95.78	95.78	0
357	5q	126807	130366	3560	chr1: 220421937 - 220425497	97.59	97.59	0
358	5q	130510	150281	19772	chr1: 220425641 - 220445916	97.24	97.24	0
359	5q	130510	131688	1179	1p: 120190 - 121369	98.33	98.33	0
360	5q	135265	158314	23050	1p: 124946 - 147924	98.87	98.87	0
361	5q	136460	138396	1937	chr1: 224457838 - 224459772	97.25	97.25	0
362	5q	137159	189359	52201	3q: 51879 - 1	98.61	98.61	0
363	5q	138750	151484	12735	chr1: 224467506 - 224480262	96.84	96.84	0
364	5q	142300	151187	8888	chr7: 55985248 - 55994150	94.68	94.68	0
365	5q	142303	144322	2020	chr7: 61805021 - 61806472<br>chr1: 193837523 - 193839718<br>chr7: 55420846 - 55423188	92.28<br>92.72<br>92.77	92.59	0
366	5q	142323	149040	6718	chr7: 45414266 - 45420987	95.29	95.29	0
367	5q	146288	157214	10927	chr7: 127667226 - 127677946	96.59	96.59	0

368	5q	146307	149040	2734	chr7: 51009103 - 51011866<br>chr7: 39393644 - 39396387<br>chr7: 62457999 - 62460749<br>chr7: 55363599 - 55366358<br>chr7: 56432157 - 56434907<br>chr1: 219029846 - 219032485	93.00<br>93.00<br>94.00<br>94.00<br>94.00<br>97.00	94.17	0
369	5q	147611	157214	9604	chr4: 120680507 - 120690578	95.28	95.28	0
370	5q	149130	157214	8085	chr7: 45402369 - 45414176	94.95	94.95	0
371	5q	149130	150883	1754	chr7: 56434997 - 56436149<br>chr1: 219032574 - 219033728<br>chr7: 62455575 - 62457909<br>chr7: 55366448 - 55368463<br>chr7: 39391514 - 39393554<br>chr7: 51011956 - 51014002	94.56<br>96.98<br>94.37<br>94.85<br>95.26<br>94.72	95.12	0
372	5q	151189	155937	4749	chr1: 224460233 - 224466670	94.41	94.41	0
373	5q	154639	162158	7520	1p: 151063 - 158591	98.78	98.78	0
374	5q	154639	158314	3676	1p: 147657 - 151331	98.91	98.91	0
375	5q	154642	155937	1296	chr7: 51020104 - 51021398	95.32	95.32	0
376	5q	154643	157207	2565	chr7: 55375429 - 55378281<br>chr7: 39386619 - 39389187<br>chr7: 56005902 - 56008476<br>chr1: 219039323 - 219041899<br>chr1: 220447184 - 220449711	96.40<br>95.18<br>96.06<br>95.95<br>97.75	96.27	0
377	5q	158046	189359	31314	chr1: 219039323 - 219078541<br>chr1: 239547046 - 239583712<br>chr4: 120688005 - 120724640	97.44<br>97.40<br>96.70	97.18	0
378	5q	158046	174974	16929	chr7: 56005902 - 56027313	93.1	93.1	0
379	5q	158093	160801	2709	chr7: 55375429 - 55383906<br>chr7: 56015725 - 56026447	93.85<br>90.67	92.26	0
380	5q	160422	189359	28938	chr10: 38735562 - 38764372<br>chr4: 119876270 - 119899413	97.16<br>97.58	97.37	0
381	5q	160425	186904	26480	chr1: 220455168 - 220481396	97.8	97.8	0
382	5q	160425	182182	21758	chr7: 39360178 - 39381110	92.94	92.94	0
383	5q	160425	177040	16616	chr7: 127645453 - 127661758	95.78	95.78	0
384	5q	160449	164697	4249	chr7: 45392682 - 45396890	96.05	96.05	0
385	5q	161794	167842	6049	chr11: 49997861 - 50003428<br>chr7: 62412186 - 62417768<br>chr7: 56475714 - 56481294<br>chr7: 51029490 - 51036010	90.62<br>91.30<br>91.30<br>93.07	91.57	0
386	5q	161924	164697	2774	chr7: 55385504 - 55388239	94.24	94.24	0
387	5q	164445	189359	24915	1p: 160844 - 185365	98.35	98.35	0
388	5q	164445	174106	9662	chr7: 65587537 - 65593232	91.45	91.45	0
389	5q	164445	167654	3210	chr7: 64516409 - 64519292	91.91	91.91	0
390	5q	168268	173807	5540	chr7: 64511343 - 64515807	90.86	90.86	0
391	5q	173730	189359	15630	9q: 16434 - 834<br>chr7: 45374387 - 45389874	98.07<br>96.33	97.2	0
392	5q	176293	177779	1487	chr7: 64507178 - 64508843	92.29	92.29	0
393	5q	176777	182238	5462	chr7: 56029594 - 56035175	92.96	92.96	0
394	5q	183119	184248	1130	chr7: 56036051 - 56037182	92.84	92.84	0
395	5q	184209	189359	5151	chr7: 127639839 - 127644996	95.62	95.62	0
396	5q	186770	188256	1487	chr7: 75222310 - 75223815<br>chr7: 65540948 - 65542445	93.75<br>93.81	93.78	0
397	5q	189665	204814	15150	chr9: 43466349 - 43481354	94.32	94.32	0
398	5q	189665	195466	5802	chr21: 13660344 - 13666335<br>chr18: 14857139 - 14863027	94.28<br>94.32	94.3	0
399	5q	191429	192460	1032	chr5: 177462676 - 177463723	90.73	90.73	1
400	5q	195065	204816	9752	chr21: 10119664 - 10128801	94.13	94.13	0
401	5q	239082	240163	1082	chr5: 108147435 - 108147689	91.33	91.33	1
402	5q	361545	363994	2450	chr13: 100054189 - 100087959	93.16	93.16	0
403	5q	473470	474634	1165	chr5: 180306440 - 180307608	98	98	1
404	5q	490760	495945	5186	chr15: 50626603 - 50636713	92.68	92.68	0
405	5q	491008	494245	3238	chr2: 31492818 - 31496144	91.05	91.05	0
406	5q	494980	496001	1022	chr2: 100583153 - 100584172	91	91	0
407	6p	1	58402	58402	15q: 116787 - 59048	98.13	98.13	0
408	6p	1	87328	87328	19p: 51596 - 143803	98.2	98.2	0

**409**	6p	16681	66233	49553	1p: 70481 - 120046	98.56	98.56	0
**410**	6p	16681	80537	63857	8p: 37604 - 103892	98.12	98.12	0
**411**	6p	17348	91429	74082	6q: 139658 - 72955	98.47	98.47	1
**412**	6p	17691	87325	69635	5q: 156343 - 80764<br>11p: 1 - 73516	98.19<br>98.37	98.28	0
**413**	6p	62132	91398	29267	20q: 50954 - 21027	98.1	98.1	0
**414**	6p	62134	86141	24008	chrY: 24761547 - 24791629<br>chrY: 25847059 - 25877147	95.68<br>95.68	95.68	0
**415**	6p	62241	87327	25087	chr1: 239518847 - 239548748<br>chr10: 38770686 - 38800933<br>7p: 2359 - 33495<br>chr4: 119905733 - 119936314<br>16q: 64320 - 32910	97.44<br>96.38<br>97.68<br>96.78<br>97.59	97.17	0
**416**	6p	62675	66233	3559	chr1: 220421937 - 220425497	97.56	97.56	0
**417**	6p	62675	77184	14510	chr4: 165542477 - 165556919	95.14	95.14	0
**418**	6p	62675	82522	19848	2q: 37420 - 17207	97.03	97.03	0
**419**	6p	66377	67555	1179	1p: 120190 - 121369	95.96	95.96	0
**420**	6p	66377	76979	10603	chr1: 220425641 - 220436743	96.65	96.65	0
**421**	6p	71131	76979	5849	1p: 124946 - 130821	97.35	97.35	0
**422**	6p	72297	74224	1928	chr1: 224457849 - 224459772	97.34	97.34	0
**423**	6p	72998	87328	14331	3q: 19226 - 1	98.04	98.04	0
**424**	6p	74610	86919	12310	chr1: 224460233 - 224480262	96.9	96.9	0
**425**	6p	78151	80082	1932	chr1: 193837523 - 193838976<br>chr7: 61805021 - 61807375	93.19<br>92.00	92.59	0
**426**	6p	78151	87325	9175	chr1: 220437912 - 220448841<br>chr7: 55985248 - 56007603<br>1p: 131997 - 145957	97.50<br>95.73<br>97.95	97.06	0
**427**	6p	78174	84906	6733	chr7: 45414266 - 45420987	95.66	95.66	0
**428**	6p	79475	83045	3571	chr7: 55422123 - 55425758	91.58	91.58	0
**429**	6p	82154	87323	5170	chr7: 127668095 - 127677946	96.56	96.56	0
**430**	6p	82173	84906	2734	chr7: 51009103 - 51011866<br>chr7: 39393644 - 39396387<br>chr7: 62457999 - 62460749<br>chr7: 55363599 - 55366358<br>chr7: 56432157 - 56434907<br>chr1: 219029845 - 219032485	93.00<br>93.24<br>94.00<br>94.00<br>95.00<br>97.00	94.37	0
**431**	6p	83477	87328	3852	chr4: 120680507 - 120689715	95.09	95.09	0
**432**	6p	84996	86400	1405	chr7: 62456760 - 62457909<br>chr7: 56434997 - 56436148<br>chr7: 51011956 - 51021398	94.14<br>94.29<br>94.51	94.31	0
**433**	6p	84996	87324	2329	chr7: 39387491 - 39393554<br>chr7: 55366448 - 55377459<br>chr7: 45403235 - 45414176<br>chr1: 219032574 - 219041033	95.63<br>95.17<br>95.47<br>96.80	95.77	0
**434**	6p	91477	93644	2168	21q: 19448 - 4338<br>10q: 20030 - 3829<br>1p: 281333 - 283465<br>16q: 104531 - 102637	93.37<br>91.83<br>92.33<br>93.28	92.7	0
**435**	6p	91611	105620	14010	1q: 28891 - 8975	93.62	93.62	0
**436**	6p	91699	94972	3274	chr2: 114079703 - 114096772<br>22q: 19698 - 2133<br>16q: 7769 - 4748<br>18p: 91144 - 94731<br>19q: 16715 - 13047<br>19p: 182231 - 185064<br>3q: 60982 - 57730<br>11p: 111977 - 115229<br>9q: 25525 - 22273<br>7p: 71845 - 75097<br>8p: 105117 - 107957	93.13<br>92.90<br>93.36<br>92.27<br>92.91<br>92.76<br>92.88<br>92.88<br>92.79<br>92.79<br>92.73	92.85	0
**437**	6p	91746	98867	7122	4q: 11634 - 5845<br>4p: 14202 - 22772<br>2p: 3251 - 10453	92.55<br>93.20<br>91.39	92.38	0
					5p: 2293 - 8075			

439	6p	114644	116863	2220	chr16: 32541387 - 32543099	92	92	0
440	6q	1	133275	133275	5q: 137868 - 5836	98.63	98.63	0
441	6q	1	33611	33611	17q: 43948 - 8545	98.2	98.2	0
442	6q	1	7193	7193	10q: 17677 - 8719<br>21q: 17747 - 8764<br>22q: 16000 - 7022<br>chr2: 114084500 - 114093488<br>19q: 13018 - 4044	94.99<br>94.73<br>94.55<br>94.53<br>95.02	94.76	0
443	6q	1	6375	6375	1q: 8975 - 1854<br>8p: 116423 - 123045	95.00<br>94.70	94.85	0
444	6q	1	1973	1973	chr3: 75756534 - 75760281	94.4	94.4	0
445	6q	1847	7194	5348	19p: 193598 - 199081<br>4p: 7719 - 14175	94.03<br>92.79	93.41	0
446	6q	4191	9611	5421	16q: 13032 - 1699	96.87	96.87	0
447	6q	4191	5868	1678	5p: 612 - 2291	94.7	94.7	0
448	6q	4559	7195	2637	4q: 5818 - 2771<br>18p: 88797 - 91117	92.20<br>93.29	92.75	0
449	6q	9260	128333	119074	1p: 1 - 122607	98.74	98.74	0
450	6q	34546	64494	29949	chrY: 25807359 - 25838662<br>chrY: 24800028 - 24831337	96.93<br>96.93	96.93	0
451	6q	34546	60422	25877	chr4: 119695468 - 119722680	97.52	97.52	0
452	6q	34546	37542	2997	chr1: 239479432 - 239482412	98.08	98.08	0
453	6q	34589	139657	105069	8p: 1 - 112857	98.55	98.55	0
454	6q	36178	37542	1365	chr1: 220383273 - 220384637	98.14	98.14	0
455	6q	38563	64494	25932	chr1: 220385665 - 220412991<br>chr1: 239483436 - 239510646	97.50<br>97.47	97.48	0
456	6q	54277	64494	10218	16q: 23961 - 13817	97.62	97.62	0
457	6q	56258	64494	8237	2q: 8258 - 107	97.67	97.67	0
458	6q	60426	64494	4069	chr4: 119944731 - 119948842	97.98	97.98	0
459	6q	72288	139658	67371	6p: 16681 - 91429	98.51	98.51	1
460	6q	72288	133275	60988	19p: 68278 - 124700	98.48	98.48	0
461	6q	72955	117939	44985	15q: 116787 - 75307	98.12	98.12	0
462	6q	73642	133275	59634	11p: 1 - 54766	98.61	98.61	0
463	6q	121665	139627	17963	20q: 50954 - 21029	97.27	97.27	0
464	6q	121883	133274	11392	chr1: 239518849 - 239530560<br>chr10: 38789362 - 38800932<br>chr4: 119924755 - 119936314<br>chrY: 25847059 - 25864666<br>chrY: 24774026 - 24791629<br>7p: 2360 - 13964<br>16q: 43956 - 32910<br>chr4: 165542477 - 165553415<br>chr1: 220421937 - 220433466<br>2q: 28270 - 17207	97.48<br>97.04<br>96.69<br>96.56<br>96.56<br>98.08<br>98.20<br>95.86<br>97.69<br>97.62	97.18	0
465	6q	130668	133267	2600	1p: 124946 - 127509	96.97	96.97	0
466	6q	131862	133275	1414	chr1: 224458366 - 224459772	97.14	97.14	0
467	6q	133279	137789	4511	8p: 130338 - 135407	97.54	97.54	0
468	6q	133279	134337	1059	1p: 241682 - 242735	98.36	98.36	0
469	6q	139446	146861	7416	10q: 14856 - 3829<br>22q: 13192 - 2133	92.36<br>91.57	91.97	0
470	6q	139446	145792	6347	5q: 7797 - 1037<br>17q: 12289 - 3110<br>21q: 12502 - 4338<br>19p: 189683 - 193721	92.54<br>94.37<br>92.50<br>92.12	92.88	0
471	6q	139446	141080	1635	chr2: 114079703 - 114081312	91.68	91.68	0
472	6q	140681	145794	5114	19q: 7793 - 639	91.94	91.94	0
473	6q	162238	164340	2103	chr12: 106789730 - 106791527	93.03	93.03	0
474	6q	162241	168430	6190	chr9: 113688734 - 113689483	92.62	92.62	0
475	7p	1	17376	17376	chr4: 165539585 - 165556842	95.92	95.92	0
476	7p	11	27604	27594	chrY: 24760333 - 24793975<br>chrY: 25844723 - 25878360	96.10<br>96.10	96.1	0
477	7p	91	1188	1098	chr1: 220419136 - 220420213	98.15	98.15	0
478	7p	91	22770	22680	2q: 37420 - 14408	97.49	97.49	0
479	7p	91	73743	73653	16q: 104535 - 30092	98.74	98.74	0

480	7p	223	34149	33927	20q: 46227 - 18783<br>chr4: 119904873 - 119938569<br>chr10: 38769816 - 38803195<br>chr1: 239517108 - 239549617	97.45<br>96.50<br>96.67<br>97.02	96.91	0
481	7p	2359	33495	31137	6p: 62133 - 87328	97.77	97.77	0
482	7p	2359	66000	63642	5q: 189359 - 126263	98.41	98.41	0
483	7p	2360	4799	2440	1p: 115941 - 118380	97.3	97.3	0
484	7p	2360	13959	11600	6q: 133270 - 121665	98.23	98.23	0
485	7p	2360	20796	18437	8p: 85490 - 103892	98.21	98.21	0
486	7p	2360	75076	72717	19p: 112806 - 185461	98.45	98.45	0
487	7p	2360	89945	87586	11p: 43183 - 128459	98.61	98.61	0
488	7p	2905	6467	3563	chr1: 220421937 - 220425497	98.09	98.09	0
489	7p	4882	6467	1586	1p: 118463 - 120046	99.15	99.15	0
490	7p	6611	9026	2416	1p: 120190 - 122607	98.33	98.33	0
491	7p	6611	26401	19791	chr1: 220425641 - 220445916	97.27	97.27	0
492	7p	11360	35468	24109	1p: 124946 - 147924	98.35	98.35	0
493	7p	12555	14503	1949	chr1: 224457827 - 224459772	97.82	97.82	0
494	7p	13255	102116	88862	3q: 87850 - 1	98.44	98.44	0
495	7p	14848	27604	12757	chr1: 224467506 - 224480262	96.38	96.38	0
496	7p	18424	27307	8884	chr7: 55985248 - 55994150	94.61	94.61	1
497	7p	18427	20413	1987	chr7: 61805021 - 61806480<br>chr1: 193837523 - 193839718<br>chr7: 55420846 - 55423090	92.11<br>92.86<br>92.63	92.53	1
498	7p	18447	25154	6708	chr7: 45414266 - 45420987	94.63	94.63	1
499	7p	22443	25154	2712	chr7: 56432166 - 56434907<br>chr7: 62457999 - 62460740<br>chr7: 51009141 - 51011866<br>chr7: 39393644 - 39396354<br>chr7: 55363641 - 55366358<br>chr1: 219029846 - 219032485	94.00<br>94.00<br>93.00<br>93.76<br>94.00<br>97.00	94.29	1
500	7p	22444	34368	11925	chr7: 127667226 - 127677904	96.27	96.27	1
501	7p	23725	34368	10644	chr4: 120680507 - 120690578	94.44	94.44	0
502	7p	25244	34368	9125	chr7: 45402369 - 45414176	94.38	94.38	1
503	7p	25245	27003	1759	chr7: 56434997 - 56436149<br>chr1: 219032574 - 219033728<br>chr7: 62455575 - 62457909<br>chr7: 51011956 - 51014002<br>chr7: 39391514 - 39393554<br>chr7: 55366459 - 55368463	94.28<br>96.70<br>94.13<br>94.60<br>95.13<br>94.73	94.93	1
504	7p	27309	33084	5776	chr1: 224460233 - 224466670	94.16	94.16	0
505	7p	31783	35468	3686	1p: 147657 - 151331	97.15	97.15	0
506	7p	31783	39251	7469	1p: 151063 - 158530	98.42	98.42	0
507	7p	31786	33084	1299	chr7: 51020104 - 51021398	95.16	95.16	1
508	7p	31787	34361	2575	chr7: 55375429 - 55378283<br>chr7: 56005902 - 56008476<br>chr7: 39386619 - 39389187<br>chr1: 219039323 - 219041899<br>chr1: 220447184 - 220449711	95.35<br>95.07<br>94.37<br>95.26<br>96.43	95.3	1
509	7p	35200	37796	2597	chr7: 55375429 - 55383896	93.93	93.93	1
510	7p	35200	45231	10032	chr7: 56005902 - 56021808	92.74	92.74	1
511	7p	35200	69447	34248	chr1: 219039323 - 219081989	97.25	97.25	0
512	7p	35200	71832	36633	chr4: 120688005 - 120730560<br>chr1: 239547046 - 239589627	96.34<br>97.45	96.9	0
513	7p	37576	71832	34257	chr10: 38729667 - 38764372<br>chr4: 119870679 - 119899413	97.00<br>97.37	97.19	0
514	7p	37579	53708	16130	chr7: 127645453 - 127661758	95.91	95.91	1
515	7p	37579	58830	21252	chr7: 39360178 - 39381110	92.86	92.86	1
516	7p	37579	63550	25972	chr1: 220455168 - 220481396	97.68	97.68	0
517	7p	37603	41850	4248	chr7: 45392682 - 45396890	95.99	95.99	1
518	7p	38948	41850	2903	chr7: 55385064 - 55388239	92.96	92.96	1
519	7p	38950	45231	6282	chr7: 51029490 - 51036010	92.76	92.76	1
520	7p	40374	44471	4098	chr11: 49999260 - 50003428<br>chr7: 56477111 - 56481294<br>chr7: 62412186 - 62416373	90.94<br>92.19<br>92.19	91.77	1

521	7p	41598	44473	2876	chr7: 64516409 - 64519292	91.46	91.46	1
522	7p	41598	71832	30235	1p: 160844 - 191186	98.24	98.24	0
523	7p	44629	50776	6148	chr7: 65587537 - 65590704<br>chr7: 64511343 - 64515807	91.48<br>91.43	91.46	1
524	7p	50174	102116	51943	9q: 51440 - 600	98.72	98.72	0
525	7p	50407	51792	1386	chr7: 56025929 - 56027313	93.19	93.19	1
526	7p	50414	71832	21419	chr7: 45368447 - 45390152	96.35	96.35	1
527	7p	52961	54425	1465	chr7: 64507178 - 64508843	91.9	91.9	1
528	7p	53445	58886	5442	chr7: 56029594 - 56035175	92.82	92.82	1
529	7p	59768	60894	1127	chr7: 56036051 - 56037182	92.33	92.33	1
530	7p	60855	69447	8593	chr7: 127636388 - 127644996	95.48	95.48	1
531	7p	63416	64900	1485	chr7: 75222310 - 75223815<br>chr7: 65540950 - 65542445	92.00<br>93.70	92.85	1
532	7p	65825	71832	6008	chr11: 50209890 - 50211062	90	90	0
533	7p	66399	67757	1359	chr7: 63774859 - 63776243<br>chr7: 64469009 - 64470133	90.56<br>90.16	90.36	1
534	7p	68043	71832	3790	chr1: 220481705 - 220485566	97.16	97.16	0
535	7p	71800	74951	3152	8p: 104622 - 108356<br>16q: 7774 - 5156<br>10q: 21274 - 18113<br>1q: 12159 - 9311<br>6p: 91944 - 95168<br>18p: 91554 - 95132<br>19q: 16701 - 13461<br>22q: 19684 - 16443<br>4p: 14618 - 17140<br>4q: 8783 - 6261<br>chr2: 114093933 - 114097169	97.24<br>95.38<br>93.71<br>93.28<br>92.94<br>94.44<br>96.73<br>96.98<br>94.05<br>94.27<br>96.79	95.07	0
536	7p	71846	73336	1491	1p: 281741 - 283465<br>21q: 19448 - 18188	96.14<br>95.69	95.91	0
537	7p	73396	75083	1688	2p: 4878 - 6570	92.18	92.18	0
538	7p	74366	101860	27495	16q: 131367 - 105158	98.15	98.15	0
539	7p	78903	88088	9186	17p: 727 - 18220	97.62	97.62	0
540	7p	130045	141501	11457	3q: 99280 - 87850	97.81	97.81	0
541	7p	130045	141501	11457	9q: 62988 - 51440	97.81	97.81	0
542	7q	100663	102918	2256	chr1: 148027673 - 148035069	90.78	90.78	0
543	8p	1	25799	25799	chr4: 119695468 - 119722637	97.49	97.49	0
544	8p	1	29862	29862	chr1: 239479475 - 239510646<br>chrY: 25807402 - 25838662<br>chrY: 24800028 - 24831294	97.48<br>96.80<br>96.79	97.02	0
545	8p	1	87928	87928	1p: 26996 - 118380	98.82	98.82	0
546	8p	1	103892	103892	5q: 144676 - 42464	98.75	98.75	0
547	8p	1	112857	112857	6q: 139657 - 34589	98.49	98.49	0
548	8p	1591	29862	28272	chr1: 220383273 - 220412991	97.47	97.47	0
549	8p	19652	29862	10211	16q: 23961 - 13817	97.45	97.45	0
550	8p	21631	29862	8232	2q: 8258 - 107	97.6	97.6	0
551	8p	25803	29862	4060	chr4: 119944731 - 119948842	97.74	97.74	0
552	8p	37604	103892	66289	19p: 68278 - 131513<br>6p: 16681 - 80537	98.33<br>98.00	98.16	0
553	8p	38266	81761	43496	15q: 116787 - 75307	98.02	98.02	0
554	8p	38953	103892	64940	11p: 1 - 61585	98.27	98.27	0
555	8p	85490	112827	27338	20q: 50954 - 21029	97.06	97.06	0
556	8p	85626	103892	18267	chr1: 239518849 - 239537377<br>7p: 2360 - 20796<br>chr4: 119917953 - 119936314<br>chr10: 38782531 - 38800932<br>chrY: 25847059 - 25871516<br>chrY: 24767177 - 24791629<br>2q: 35177 - 17207<br>16q: 50788 - 32910	96.96<br>98.07<br>96.82<br>96.80<br>96.28<br>96.27<br>97.05<br>97.70	96.99	0
557	8p	86034	89594	3561	chr1: 220421937 - 220425497	98.29	98.29	0
558	8p	86034	100468	14435	chr4: 165542477 - 165556842	95.67	95.67	0
559	8p	88011	89594	1584	1p: 118463 - 120046	97.94	97.94	0
560	8p	89738	92145	2408	1p: 120190 - 122607	97.61	97.61	0
561	8p	89738	103892	14155	chr1: 220425641 - 220440302	96.94	96.94	0

**562**	8p	94484	103892	9409	1p: 124946 - 134371	98.04	98.04	0
**563**	8p	95651	97588	1938	chr1: 224457839 - 224459772	97.34	97.34	0
**564**	8p	96353	103892	7540	3q: 7525 - 1	98.57	98.57	0
**565**	8p	97943	103892	5950	chr1: 224474330 - 224480262	96.92	96.92	0
**566**	8p	101522	103681	2160	chr7: 61805021 - 61806480<br>chr1: 193837523 - 193839718<br>chr7: 55985248 - 55987635<br>chr7: 55420846 - 55423188<br>chr7: 45418635 - 45420987	92.11<br>92.26<br>95.95<br>92.48<br>96.53	93.87	0
**567**	8p	104622	123046	18425	19p: 181737 - 198474	96.66	96.66	0
**568**	8p	104976	108216	3241	11p: 111483 - 115215<br>7p: 71351 - 75083<br>9q: 25511 - 21779<br>3q: 60968 - 57236<br>16q: 105875 - 102143<br>16q: 7774 - 5157<br>1q: 12159 - 9312<br>10q: 21274 - 18114<br>6p: 91945 - 94769<br>19q: 16701 - 13462<br>18p: 91555 - 95132<br>22q: 19684 - 16444<br>chr2: 114093933 - 114097169<br>4p: 14618 - 17140<br>4q: 8783 - 6261<br>2p: 3251 - 6171	97.26<br>97.29<br>97.29<br>97.26<br>97.40<br>95.03<br>93.21<br>93.41<br>92.74<br>96.97<br>94.27<br>96.76<br>96.87<br>92.86<br>93.06<br>91.79	95.22	0
**569**	8p	105117	106609	1493	1p: 281742 - 283465<br>21q: 19448 - 18188	96.19<br>95.42	95.81	0
**570**	8p	112649	123045	10397	5q: 12435 - 1040<br>17q: 16920 - 3113<br>21q: 17129 - 4341<br>chr2: 114079706 - 114092879<br>10q: 17073 - 3832<br>22q: 15407 - 2136	94.62<br>94.35<br>93.68<br>93.57<br>93.63<br>93.46	93.88	0
**571**	8p	113859	123046	9188	19q: 12422 - 639	93.73	93.73	0
**572**	8p	116378	122805	6428	1q: 8975 - 1764<br>6q: 6609 - 1	93.17<br>93.91	93.54	0
**573**	8p	120762	123146	2385	16q: 4133 - 1693<br>5p: 611 - 2409<br>4p: 11143 - 13573<br>4q: 5216 - 2781<br>18p: 88797 - 90510	93.21<br>95.15<br>91.10<br>91.47<br>93.94	92.97	0
**574**	8p	130338	135407	5070	6q: 137789 - 133279	97.71	97.71	0
**575**	8p	134360	139412	5053	1p: 237672 - 242735	98.07	98.07	0
**576**	8p	194471	231807	37337	1p: 245037 - 280290	97.26	97.26	0
**577**	8p	279211	281570	2360	1p: 231380 - 233742	97.7	97.7	0
**578**	8p	290588	307129	16542	2q: 91777 - 72438	96.56	96.56	0
**579**	8p	307013	339694	32682	2q: 71986 - 37438	96	96	0
**580**	8p	329082	353117	24036	1p: 205679 - 229635	96.36	96.36	0
**581**	8p	329082	353122	24041	chr1: 220511009 - 220534939	96.07	96.07	0
**582**	8q	79741	83765	4025	chr14: 74667926 - 74713049	93.22	93.22	0
**583**	8q	286728	288864	2137	chr3: 144028602 - 144029269<br>chr3: 175577873 - 175578523	93.25<br>91.93	92.59	0
**584**	8q	287142	288272	1131	chr21: 30557745 - 30558070	91.96	91.96	0
**585**	9p	1894	9953	8060	Xq: 10579 - 2524<br>16p: 1462 - 9529<br>Yq: 10579 - 2524	98.23<br>98.08<br>98.23	98.18	0
**586**	9p	1894	27840	25947	15q: 28282 - 2004	98.32	98.32	0
**587**	9p	1901	80815	78915	chr2: 113998296 - 114074366	98.07	98.07	0
**588**	9p	8506	27840	19335	19p: 1 - 19667	98.82	98.82	0
**589**	9p	23892	37630	13739	12p: 1705 - 15778	97.01	97.01	0
**590**	9p	41356	61444	20089	chr9: 67170612 - 67190611	97.67	97.67	1
**591**	9p	41356	63095	21740	chr9: 66932464 - 66954161	97.66	97.66	1
**592**	9p	41356	80815	39460	chr9: 68181420 - 68220146	97.34	97.34	1
**593**	9p	61591	63095	1505	chr9: 67190615 - 67192109	97.7	97.7	1

594	9p	63335	64473	1139	chr9: 67192374 - 67193524<br>chr9: 66954430 - 66955594	94.42<br>93.81	94.12	1
595	9p	64523	81099	16577	chr9: 67193574 - 67209106	97.45	97.45	1
596	9p	64523	88614	24092	chr9: 66955644 - 66978778	97.43	97.43	1
597	9p	80698	193743	113046	chr2: 113886167 - 113998262	97.86	97.86	0
598	9p	80699	190123	109425	chr9: 68061572 - 68181380	97.14	97.14	1
599	9p	82372	88610	6239	chr9: 67210389 - 67216665	97.79	97.79	1
600	9p	85947	92929	6983	chr11: 43103310 - 43113360	91.93	91.93	0
601	9p	89455	92929	3475	chr9: 66979634 - 66982999	97.72	97.72	1
602	9p	90396	103518	13123	chr9: 67218352 - 67241667	96.16	96.16	1
603	9p	106558	147516	40959	chr9: 67931971 - 67973226	97.34	97.34	1
604	9p	147662	185398	37737	chr9: 67973370 - 68011244	97.04	97.04	1
605	9p	191457	193743	2287	chr9: 66892011 - 66894295	97	97	1
606	9p	191457	193743	2287	chr9: 67131003 - 67133287	97.37	97.37	1
607	9q	600	51440	50841	7p: 50174 - 102116	98.72	98.72	0
608	9q	833	2218	1386	chr7: 56025929 - 56027313	92.79	92.79	0
609	9q	834	88449	87616	3q: 119439 - 36117	97.77	97.77	0
610	9q	834	39467	38634	11p: 90403 - 128459	98.32	98.32	0
611	9q	834	25504	24671	19p: 160708 - 185461	98.31	98.31	0
612	9q	834	24171	23338	16q: 104535 - 81201	98.96	98.96	0
613	9q	834	16434	15601	5q: 189359 - 173588	98.03	98.03	0
614	9q	839	22260	21422	1p: 169656 - 191186<br>chr10: 38729667 - 38751317<br>chr4: 119870679 - 119886584<br>chr4: 120708845 - 120730560<br>chr1: 239567905 - 239589627<br>chr7: 45368447 - 45390152	98.28<br>97.05<br>97.43<br>96.42<br>97.50<br>96.43	97.19	0
615	9q	840	19881	19042	chr1: 219062780 - 219081989	97.31	97.31	0
616	9q	840	13985	13146	chr1: 220467995 - 220481396	97.8	97.8	0
617	9q	840	4134	3295	chr7: 127645453 - 127648924	96.06	96.06	0
618	9q	870	9260	8391	chr7: 39360178 - 39369360	92.67	92.67	0
619	9q	3387	4855	1469	chr7: 64507178 - 64508843	92.18	92.18	0
620	9q	3871	9316	5446	chr7: 56029594 - 56035175	92.94	92.94	0
621	9q	5090	6480	1391	chr7: 65561411 - 65562799	90.72	90.72	0
622	9q	10199	11325	1127	chr7: 56036051 - 56037182	92.85	92.85	0
623	9q	11286	19881	8596	chr7: 127636388 - 127644996	95.54	95.54	0
624	9q	13851	15335	1485	chr7: 75222310 - 75223815<br>chr7: 65540950 - 65542445	92.31<br>94.03	93.17	0
625	9q	16259	22260	6002	chr11: 50209890 - 50211062	90	90	0
626	9q	16833	18191	1359	chr7: 63774859 - 63776243<br>chr7: 64469009 - 64470133	90.56<br>90.32	90.44	0
627	9q	18477	22260	3784	chr1: 220481705 - 220485566	97.22	97.22	0
628	9q	22230	25379	3150	8p: 104641 - 108356<br>16q: 7774 - 5156<br>10q: 21274 - 18113<br>1q: 12159 - 9311<br>6p: 91944 - 95168<br>18p: 91554 - 95132<br>22q: 19684 - 16443<br>19q: 16701 - 13461<br>chr2: 114093933 - 114097169<br>4q: 8783 - 6261<br>4p: 14618 - 17140	97.23<br>95.15<br>93.87<br>93.28<br>92.94<br>94.44<br>96.98<br>96.73<br>96.79<br>94.27<br>94.05	95.07	0
629	9q	22274	23764	1491	1p: 281741 - 283465<br>21q: 19448 - 18188	96.14<br>95.69	95.91	0
630	9q	23824	25511	1688	2p: 4878 - 6570	92.18	92.18	0
631	9q	24794	51184	26391	16q: 131367 - 105158	98.25	98.25	0
632	9q	29318	37610	8293	17p: 727 - 18220	97.63	97.63	0
633	9q	51440	62988	11549	7p: 130045 - 141501	97.95	97.95	0
634	9q	63045	124136	61092	10p: 10952 - 74445	94.32	94.32	0
635	9q	63045	108804	45760	18p: 15346 - 63444	95.07	95.07	0
636	9q	63059	88449	25391	chr12: 34168581 - 34211062	91.23	91.23	0
637	9q	64550	88437	23888	chrY: 18042510 - 18076998	91.08	91.08	0
638	9q	64550	70669	6120	chrY: 18957030 - 18963521	91.28	91.28	0

**639**	9q	65244	83002	17759	1p: 286972 - 301429	93.79	93.79	0
**640**	9q	69973	71163	1191	chrY: 10567257 - 10568441	92.43	92.43	0
**641**	9q	77401	83002	5602	4q: 126874 - 121031	93.56	93.56	0
**642**	9q	78086	80030	1945	chrY: 18940700 - 18942639	91.28	91.28	0
**643**	9q	81025	88447	7423	16q: 138855 - 131467<br>chr12: 36865089 - 36876160	95.54<br>91.04	93.29	0
**644**	9q	83210	87892	4683	4q: 121040 - 109513<br>chr1: 223993648 - 224001019	93.15<br>91.02	92.09	0
**645**	9q	86916	88448	1533	chrY: 10552604 - 10554424<br>chr3: 75834770 - 75835986	92.56<br>90.76	91.66	0
**646**	9q	108847	133085	24239	18p: 63487 - 87912	97.5	97.5	0
**647**	9q	113409	129597	16189	16p: 17959 - 37573	91.9	91.9	0
**648**	9q	113409	123870	10462	Yq: 29837 - 19014<br>Xq: 29837 - 19014	92.79<br>92.79	92.79	0
**649**	10p	1	13334	13334	chr1: 224017101 - 224033315	90.94	90.94	0
**650**	10p	1	39687	39687	chr12: 34147628 - 34211062	90.9	90.9	0
**651**	10p	1	59732	59732	18p: 4415 - 63117	97.6	97.6	0
**652**	10p	28	3077	3050	chr2: 132394730 - 132398023<br>chr22: 14844180 - 14847482<br>chr14: 18424456 - 18427756<br>1p: 284205 - 286519	94.87<br>94.25<br>94.25<br>93.81	94.3	0
**653**	10p	10584	18600	8017	chrY: 18957030 - 18965446	90.75	90.75	0
**654**	10p	10768	39371	28604	chrY: 18040585 - 18076372<br>3q: 119439 - 99337	90.58<br>95.08	92.83	0
**655**	10p	10952	74445	63494	9q: 124136 - 63045	94.26	94.26	0
**656**	10p	13173	19094	5922	chrY: 10567257 - 10573176	91.41	91.41	0
**657**	10p	13173	34283	21111	1p: 286972 - 301421	93.58	93.58	0
**658**	10p	29169	34592	5424	4q: 126874 - 121031<br>chrY: 18937298 - 18942639	93.29<br>90.00	91.65	0
**659**	10p	32563	39685	7123	16q: 138855 - 131398<br>chr12: 36865089 - 36875578	95.18<br>92.08	93.63	0
**660**	10p	33046	34901	1856	chr1: 223987482 - 223989310	91.19	91.19	0
**661**	10p	34674	39316	4643	4q: 121040 - 109513<br>chr1: 223993648 - 224001050<br>chrY: 10552604 - 10561454	92.52<br>92.00<br>91.79	92.1	0
**662**	10p	60126	74443	14318	18p: 63527 - 79293	93.08	93.08	0
**663**	10p	64762	74460	9699	16p: 17959 - 29051<br>Xq: 30139 - 19014	92.01<br>92.24	92.12	0
**664**	10p	64762	74476	9715	Yq: 30139 - 19014	92.24	92.24	0
**665**	10q	3829	21689	17861	chr2: 114079703 - 114097183	96.02	96.02	0
**666**	10q	3829	5438	1610	6q: 141080 - 139446	92.03	92.03	0
**667**	10q	3830	17532	13703	19p: 189683 - 199397<br>8p: 112649 - 123046	96.02<br>93.54	94.78	0
**668**	10q	5039	21689	16651	19q: 16715 - 639<br>22q: 19698 - 3342	96.10<br>95.79	95.94	0
**669**	10q	5039	19372	14334	21q: 19448 - 5548	96.59	96.59	0
**670**	10q	5039	17677	12639	17q: 17504 - 4319<br>5q: 13019 - 2247	94.93<br>95.77	95.35	0
**671**	10q	8629	21274	12646	1q: 12159 - 1764	93.97	93.97	0
**672**	10q	8629	12450	3822	chr3: 75756447 - 75760281	93.7	93.7	0
**673**	10q	8719	17677	8959	6q: 7193 - 1	94.94	94.94	0
**674**	10q	12304	21689	9386	4p: 7699 - 17154	91.42	91.42	0
**675**	10q	14669	21689	7021	5p: 617 - 2421	93.28	93.28	0
**676**	10q	14670	20034	5365	16q: 7077 - 1705	93.33	93.33	0
**677**	10q	14674	46814	32141	4q: 36107 - 2781	97.36	97.36	0
**678**	10q	15394	26302	10909	18p: 88797 - 102436	95.86	95.86	0
					2p: 3280 - 4793			

680	10q	18113	21273	3161	19p: 182231 - 185062<br>7p: 71845 - 74683<br>3q: 60572 - 57730<br>9q: 25111 - 22273<br>16q: 105475 - 102637<br>11p: 111977 - 114819<br>8p: 105117 - 107957	94.11<br>94.10<br>93.98<br>94.26<br>94.26<br>93.98<br>93.86	94.08	0
681	10q	19907	21274	1368	2p: 5103 - 6171	92.86	92.86	0
682	10q	26091	46139	20049	4q: 38723 - 18742	98.93	98.93	0
683	10q	26091	42829	16739	4q: 38723 - 22035	98.94	98.94	0
684	10q	26091	40278	14188	4q: 39488 - 25335	98.83	98.83	0
685	10q	26091	37163	11073	4q: 39682 - 28629	98.84	98.84	0
686	10q	26091	33868	7778	4q: 39686 - 31922	98.75	98.75	0
687	10q	26091	30558	4468	4q: 39686 - 35215	98.61	98.61	0
688	10q	26091	27259	1169	4q: 39686 - 38512	98.29	98.29	0
689	10q	29390	46814	17425	4q: 32814 - 15442	98.91	98.91	0
690	10q	32700	46814	14115	4q: 29521 - 15442	98.87	98.87	0
691	10q	33086	34151	1066	chr3: 75800797 - 75801859<br>4q: 83327 - 82274	91.00<br>98.00	94.5	0
692	10q	35999	46814	10816	4q: 26221 - 15442	98.94	98.94	0
693	10q	39308	46814	7507	4q: 22927 - 15442	98.85	98.85	0
694	10q	42618	46814	4197	4q: 19634 - 15442	98.66	98.66	0
695	10q	43004	44069	1066	chr3: 75800797 - 75801859<br>4q: 83327 - 82274	91.00<br>98.00	94.5	0
696	10q	46814	88677	41864	4q: 81906 - 39686	98.55	98.55	0
697	10q	48653	57770	9118	chrY: 18829808 - 18838126<br>chrY: 18167906 - 18176224	90.00<br>90.00	90	0
698	10q	50662	88610	37949	chr3: 75761698 - 75800337	95.44	95.44	0
699	10q	51576	56678	5103	chr12: 36756559 - 36761457	90.76	90.76	0
700	10q	74008	86451	12444	chr16: 34561514 - 34569480	90	90	0
701	10q	96865	98332	1468	chr5: 169326378 - 169329979	91.62	91.62	0
702	10q	469322	470901	1580	chr13: 83378198 - 83379391	91.44	91.44	0
703	10q	470021	473387	3367	chr8: 33697501 - 33698875	91.35	91.35	0
704	10q	470172	471273	1102	chrX: 26335756 - 26336472	92	92	0
705	11p	1	39454	39454	15q: 116787 - 75995	98.17	98.17	0
706	11p	1	47285	47285	1p: 71832 - 120046	98.62	98.62	0
707	11p	1	54766	54766	6q: 133275 - 73642	98.62	98.62	0
708	11p	1	61585	61585	8p: 38953 - 103892	98.36	98.36	0
709	11p	1	73516	73516	6p: 18035 - 87328	98.33	98.33	0
710	11p	1	90919	90919	5q: 174104 - 81451	98.6	98.6	0
711	11p	1	115208	115208	19p: 69632 - 185461	98.61	98.61	0
712	11p	43183	68406	25224	chrY: 24760333 - 24791629<br>chrY: 25847059 - 25878360	96.11<br>96.12	96.12	0
713	11p	43183	74176	30994	20q: 46227 - 21029<br>chr10: 38769816 - 38800932<br>chr4: 119904873 - 119936314<br>chr1: 239518849 - 239549617	97.66<br>96.56<br>96.67<br>97.15	97.01	0
714	11p	43183	125961	82779	7p: 2360 - 88088	98.7	98.7	0
715	11p	43725	47285	3561	chr1: 220421937 - 220425497	97.8	97.8	0
716	11p	43725	57778	14054	chr4: 165542477 - 165556462	95.7	95.7	0
717	11p	43725	63564	19840	2q: 37420 - 17207	97.38	97.38	0
718	11p	43725	90919	47195	16q: 81717 - 32910	98.28	98.28	0
719	11p	47429	48608	1180	1p: 120190 - 121369	98.33	98.33	0
720	11p	47429	67203	19775	chr1: 220425641 - 220445916	97.25	97.25	0
721	11p	52220	75496	23277	1p: 124981 - 147924	98.57	98.57	0
722	11p	53357	55279	1923	chr1: 224457849 - 224459772	96.91	96.91	0
723	11p	54057	90919	36863	3q: 36633 - 1	98.84	98.84	0
724	11p	55651	68406	12756	chr1: 224467506 - 224480247	96.85	96.85	0
725	11p	59210	68109	8900	chr7: 55985248 - 55994150	94.76	94.76	0
726	11p	59213	61532	2320	chr1: 193837523 - 193839718<br>chr7: 61805021 - 61807375<br>chr7: 55420846 - 55423188	92.86<br>91.53<br>93.06	92.48	0
727	11p	59233	65948	6716	chr7: 45414266 - 45420987	95	95	0
728	11p	63196	74396	11201	chr7: 127667226 - 127677946	96.5	96.5	0

729	11p	63215	65948	2734	chr7: 51009103 - 51011866<br>chr7: 39393644 - 39396387<br>chr7: 62457999 - 62460749<br>chr7: 55363599 - 55366358<br>chr7: 56432157 - 56434907<br>chr1: 219029846 - 219032485	93.00<br>93.00<br>94.00<br>94.00<br>94.00<br>97.00	94.17	0
730	11p	64519	74396	9878	chr4: 120680507 - 120690578	95.34	95.34	0
731	11p	66038	67805	1768	chr1: 219032574 - 219033728<br>chr7: 56434997 - 56436149<br>chr7: 62455575 - 62457909<br>chr7: 55366448 - 55368463<br>chr7: 39391514 - 39393554<br>chr7: 51011956 - 51014002	96.69<br>94.28<br>94.37<br>94.45<br>95.13<br>94.85	94.96	0
732	11p	66038	74396	8359	chr7: 45402369 - 45414176	94.75	94.75	0
733	11p	68111	73105	4995	chr1: 224460233 - 224466670	94.16	94.16	0
734	11p	71805	75496	3692	1p: 147657 - 151331	97.75	97.75	0
735	11p	71805	79340	7536	1p: 151063 - 158591	98.6	98.6	0
736	11p	71808	73105	1298	chr7: 51020104 - 51021398	95.17	95.17	0
737	11p	71809	74389	2581	chr7: 55375429 - 55378283<br>chr1: 219039323 - 219041899<br>chr7: 39386619 - 39389187<br>chr7: 56005902 - 56008476<br>chr1: 220447184 - 220449711	96.28<br>96.21<br>95.33<br>96.03<br>97.32	96.23	0
738	11p	75228	91791	16564	chr7: 56005902 - 56027313	92.81	92.81	0
739	11p	75228	111964	36737	chr4: 120688005 - 120730560	96.42	96.42	0
740	11p	75259	77944	2686	chr1: 239547046 - 239555313<br>chr1: 219039323 - 219047615<br>chr7: 56015725 - 56026447	98.00<br>97.50<br>90.25	95.25	0
741	11p	77607	103679	26073	chr1: 220455168 - 220481396	97.7	97.7	0
742	11p	77609	85222	7614	chr7: 51028105 - 51036010	92.24	92.24	0
743	11p	77631	81842	4212	chr7: 45392682 - 45396890	96.21	96.21	0
744	11p	77787	90919	13133	chr10: 38750807 - 38764372<br>chr4: 119886074 - 119899413<br>chr7: 39368881 - 39380748<br>chr7: 127648414 - 127661395	97.22<br>97.89<br>93.47<br>95.80	96.09	0
745	11p	77970	109571	31602	chr1: 219047720 - 219081989	97.14	97.14	0
746	11p	77970	111964	33995	chr1: 239555446 - 239589627	97.21	97.21	0
747	11p	78976	81842	2867	chr7: 55385064 - 55388239	93.7	93.7	0
748	11p	78976	84462	5487	chr11: 49997861 - 50003428<br>chr7: 62412186 - 62417768<br>chr7: 56475714 - 56481294	90.62<br>91.43<br>91.43	91.16	1
749	11p	81590	84464	2875	chr7: 64516409 - 64519292	91.91	91.91	0
750	11p	81590	90614	9025	chr7: 65587844 - 65593232	91.11	91.11	0
751	11p	81590	111964	30375	1p: 160844 - 191186	98.33	98.33	0
752	11p	85078	90622	5545	chr7: 64511343 - 64515807	91.43	91.43	0
753	11p	90763	111964	21202	chr7: 45368447 - 45390152<br>chr10: 38729667 - 38750786<br>chr4: 119870679 - 119886048	96.20<br>96.94<br>97.35	96.83	0
754	11p	90941	93849	2909	chr7: 127645453 - 127648385	96.44	96.44	0
755	11p	90941	98964	8024	chr7: 39360178 - 39368857	92.71	92.71	0
756	11p	90941	106130	15190	5q: 189359 - 174125	98.11	98.11	0
757	11p	90941	125961	35021	9q: 37610 - 1370<br>3q: 73851 - 36653<br>16q: 117597 - 81737	98.29<br>98.54<br>98.30	98.38	0
758	11p	93102	94582	1481	chr7: 64507178 - 64508843	92.35	92.35	0
759	11p	93586	99020	5435	chr7: 56029594 - 56035175	92.59	92.59	0
760	11p	99886	101019	1134	chr7: 56036051 - 56037182	92.73	92.73	0
761	11p	100980	109571	8592	chr7: 127636388 - 127644996	95.35	95.35	0
762	11p	103545	105029	1485	chr7: 75222310 - 75223815<br>chr7: 65540950 - 65542445	92.68<br>94.03	93.36	0
763	11p	105959	111964	6006	chr11: 50209890 - 50211058	90	90	1
764	11p	106526	107887	1362	chr7: 63774859 - 63776250<br>chr7: 64469009 - 64470133	90.56<br>90.16	90.36	0
765	11p	108173	111964	3792	chr1: 220481705 - 220485566	97.26	97.26	0

766	11p	111928	115175	3248	8p: 104622 - 108356<br>10q: 21274 - 18113<br>6p: 91944 - 95168<br>22q: 19684 - 16443<br>18p: 91554 - 95132<br>1q: 12559 - 9311<br>19q: 16701 - 13461<br>4q: 8783 - 6261<br>4p: 14618 - 17140<br>chr2: 114093933 - 114097169	97.39<br>93.85<br>92.86<br>97.31<br>94.40<br>92.80<br>97.01<br>94.16<br>94.01<br>96.95	95.07	0
767	11p	111977	113607	1631	1p: 281741 - 283465<br>16q: 7077 - 5156<br>21q: 19448 - 18188	96.57<br>95.38<br>96.13	96.03	0
768	11p	113532	115215	1684	2p: 4878 - 6570	92.1	92.1	0
769	11p	119534	136823	17290	17p: 727 - 29146	98.45	98.45	0
770	11p	259549	260712	1164	chr7: 55790049 - 55790646<br>chr6: 29824853 - 29825369<br>chr12: 31790478 - 31791125	92.39<br>90.46<br>93.66	92.17	0
771	11p	259554	260747	1194	chr4: 66787231 - 66787846	93.71	93.71	0
772	11q	85593	89518	3926	chr18: 14893727 - 14897656	94.11	94.11	0
773	11q	89697	138789	49093	chr18: 14925562 - 14971183	94.46	94.46	0
774	12p	1705	5645	3941	19p: 15718 - 19667<br>15q: 28282 - 24333	96.76<br>96.76	96.76	0
775	12p	1705	15597	13893	chr2: 114039589 - 114052071<br>9p: 23892 - 37630	97.04<br>97.02	97.03	0
776	12p	27892	42389	14498	20q: 18388 - 3777	97.74	97.74	0
777	12q	455241	456964	1724	20q: 444018 - 442754	90	90	0
778	13q	1374	15153	13780	2p: 2614 - 23214	91.57	91.57	0
779	13q	1375	10045	8671	4p: 7699 - 26903	91.55	91.55	0
780	13q	1401	4251	2851	1q: 13812 - 4693	92.14	92.14	0
781	13q	1734	5935	4202	6p: 94216 - 98867	91.25	91.25	0
782	13q	2102	7373	5272	4q: 13063 - 8663	92.33	92.33	0
783	13q	2108	11739	9632	5p: 2293 - 14897	91.38	91.38	0
784	13q	38599	62471	23873	chr17: 16579782 - 16581890<br>chr17: 20209730 - 20211830	98.47<br>98.11	98.29	0
785	13q	38603	57887	19285	chr9: 99835472 - 99837034	92.41	92.41	0
786	14q	1555	5024	3470	chr16: 33518166 - 33520701	90.22	90.22	0
787	14q	5215	6605	1391	chr14: 105712752 - 105714137	90	90	1
788	14q	17998	19246	1249	chr1: 57461707 - 57462952	98.1	98.1	0
789	14q	68172	73035	4864	chr16: 33526959 - 33531826	94.72	94.72	0
790	14q	89584	90661	1078	chr14: 105816615 - 105817683	90.14	90.14	1
791	14q	89938	106193	16256	chr16: 33560842 - 33561564	91.65	91.65	0
792	14q	102384	104211	1828	chr14: 105798183 - 105800773	90	90	1
793	14q	104829	106216	1388	chr14: 105785334 - 105786719	93.18	93.18	1
794	14q	150416	152240	1825	chr14: 105705643 - 105707206<br>chr21: 9882604 - 9884598	92.70<br>91.58	92.14	1
795	14q	151099	152955	1857	chr15: 18429899 - 18431719	90.73	90.73	0
796	14q	157413	158664	1252	chr16: 33504106 - 33505367	91	91	0
797	14q	171502	173031	1530	chr16: 31958693 - 31960767<br>chr16: 33563258 - 33565002	91.64<br>91.00	91.32	0
798	14q	213296	217065	3770	chr16: 31865447 - 31869175<br>chr16: 32806991 - 32810721<br>chr16: 33661812 - 33665517	91.48<br>91.38<br>91.25	91.37	0
799	14q	213296	214704	1409	chr14: 105698396 - 105699947	91.58	91.58	1
800	14q	214297	217071	2775	chr16: 33518087 - 33520687	90.26	90.26	0
801	14q	221649	223489	1841	chr15: 18438331 - 18440159<br>chr14: 105712888 - 105714706	90.00<br>90.00	90	1
802	14q	239773	242810	3038	chr16: 33504106 - 33506584	90.52	90.52	0
803	14q	290757	292740	1984	chr16: 33496390 - 33498689	91.49	91.49	0
804	14q	295187	296429	1243	chr14: 105588909 - 105590457<br>chr14: 105762490 - 105763545<br>chr16: 32920104 - 32921368<br>chr16: 33545878 - 33547142<br>chr16: 31977088 - 31978106	91.47<br>92.34<br>90.95<br>90.47<br>90.35	91.12	1

805	14q	317376	319579	2204	chr16: 31965067 - 31967475<br>chr16: 33556533 - 33558953<br>chr14: 105652797 - 105655235<br>chr16: 32908522 - 32910710	90.58<br>90.11<br>92.91<br>91.00	91.15	1
806	14q	322259	324671	2413	chr15: 18428171 - 18430168	91.54	91.54	0
807	14q	324223	325491	1269	chr14: 105607796 - 105609059	90	90	1
808	14q	360562	361821	1260	chr2: 95060054 - 95061370	91	91	0
809	14q	360571	370049	9479	chr15: 18453235 - 18455979	92.43	92.43	0
810	14q	364239	366620	2382	chr14: 105620163 - 105622561	90.09	90.09	1
811	14q	443538	444630	1093	chr14: 105692521 - 105693618	93	93	1
812	14q	443764	455721	11958	chr15: 18438652 - 18453387	90.38	90.38	0
813	14q	483496	484585	1090	chr15: 19974033 - 19975119	91.73	91.73	0
814	14q	483496	484585	1090	chr14: 105548902 - 105549981	92.1	92.1	1
815	15q	2004	10069	8066	Xq: 10579 - 2524<br>16p: 1462 - 9529<br>Yq: 10579 - 2524	97.76<br>97.79<br>97.76	97.77	0
816	15q	2007	28282	26276	9p: 1894 - 27840<br>chr2: 114048111 - 114074366	98.39<br>97.93	98.16	0
817	15q	8621	116787	108167	19p: 1 - 109077	98.5	98.5	0
818	15q	24333	28282	3950	12p: 1705 - 5645	96.89	96.89	0
819	15q	59048	116787	57740	6p: 1 - 58402	98.06	98.06	0
820	15q	75329	116787	41459	1p: 70845 - 112213<br>8p: 37990 - 81761<br>6q: 117939 - 72955<br>5q: 122536 - 80764<br>11p: 1 - 39454	97.91<br>98.12<br>98.08<br>98.15<br>98.11	98.07	0
821	15q	187268	199153	11886	chr15: 98137309 - 98148859	96.46	96.46	1
822	15q	187385	194606	7222	chr13: 91922051 - 91930591	92.11	92.11	0
823	15q	194717	202307	7591	chr7: 30120820 - 30129014	91.18	91.18	0
824	15q	194956	196153	1198	chr15: 76796036 - 76797273	91.83	91.83	1
825	15q	196769	198171	1403	chr15: 28868410 - 28870653<br>chr15: 80506706 - 80508296<br>chr15: 80893231 - 80894821<br>chr15: 82862061 - 82863651<br>chr15: 82730871 - 82732083<br>chr15: 76798290 - 76805545<br>chr5: 174276897 - 174277917<br>chr15: 73852798 - 73853994<br>chr15: 88633970 - 88635174<br>chr15: 76007224 - 76008425	91.92<br>92.16<br>92.16<br>91.61<br>91.44<br>91.56<br>90.25<br>90.59<br>91.20<br>90.30	91.32	1
826	15q	196920	205593	8674	chrY: 25934288 - 25940928<br>chrY: 24697760 - 24704400	91.16<br>91.21	91.19	0
827	15q	196938	215927	18990	chr10: 30682692 - 30701934	90.91	90.91	0
828	15q	197828	200670	2843	chrY: 24651121 - 24653928<br>chrY: 25984763 - 25987570	91.89<br>91.89	91.89	0
829	15q	199369	204239	4871	chr15: 28871217 - 28876264	91.13	91.13	1
830	15q	199586	200748	1163	chr15: 20807150 - 20808309<br>chr15: 28162918 - 28164076<br>chr15: 28215014 - 28216172<br>chr15: 28631942 - 28633098<br>chr15: 28683991 - 28685149<br>chr15: 30673416 - 30674572	90.00<br>90.00<br>90.00<br>90.00<br>90.00<br>90.00<br>90.00	90	1

**831**	15q	201248	204230	2983	chr15: 19029385 - 19032731<br>chr15: 20988210 - 20991556<br>chr15: 21153007 - 21156349<br>chr15: 26299076 - 26302420<br>chr15: 20262047 - 20265393<br>chr15: 26572143 - 26575487<br>chr15: 30675435 - 30678238<br>chr15: 28686013 - 28688829<br>chr15: 32463988 - 32466749<br>chr15: 32610209 - 32612970<br>chr15: 28165012 - 28167753<br>chr15: 28634031 - 28636764<br>chr15: 28488465 - 28491200<br>chr15: 30477478 - 30480220<br>chr15: 30529833 - 30532570	90.89<br>90.85<br>90.78<br>90.85<br>90.90<br>90.90<br>90.59<br>90.59<br>91.07<br>91.07<br>91.01<br>90.59<br>90.59<br>91.03<br>90.59	90.82	1
**832**	15q	201420	203000	1581	chr15: 20809174 - 20810748<br>chr15: 26752549 - 26754121	92.55<br>92.55	92.55	1
**833**	15q	201501	205611	4111	chr15: 28217110 - 28221244	90.9	90.9	1
**834**	15q	202975	204549	1575	chr16: 51944569 - 51946166	90.59	90.59	0
**835**	15q	205285	210741	5457	chrY: 24655974 - 24660095<br>chrY: 25978596 - 25982717	92.46<br>92.46	92.46	0
**836**	15q	205482	207296	1815	chr15: 21157200 - 21159481<br>chr15: 26303271 - 26305558<br>chr15: 28168615 - 28170758<br>chr15: 28637626 - 28639759<br>chr15: 28689681 - 28691820<br>chr15: 28877114 - 28879256<br>chr15: 30679100 - 30681234<br>chr15: 20992795 - 20994669<br>chr15: 88636451 - 88637777<br>chr15: 26569006 - 26570361<br>chr15: 28485472 - 28486644<br>chr15: 30474484 - 30475654<br>chr15: 30526837 - 30528009	90.46<br>90.46<br>91.14<br>90.42<br>91.24<br>90.96<br>91.03<br>92.07<br>90.34<br>90.72<br>90.69<br>91.39<br>91.69	90.97	1
**837**	15q	205543	208622	3080	chr15: 19033582 - 19037186<br>chr15: 20257572 - 20260266	91.18<br>90.93	91.06	1
**838**	15q	207904	242877	34974	chr15: 98149130 - 98172164	96.82	96.82	1
**839**	15q	209356	212765	3410	chr15: 30683396 - 30686806	90.91	90.91	1
**840**	15q	209439	211568	2130	chr15: 32458883 - 32460979<br>chr15: 32605102 - 32607200<br>chr15: 19038003 - 19040161<br>chr15: 20996828 - 20998986<br>chr15: 26307729 - 26309887<br>chr15: 20254597 - 20256755<br>chr15: 26564677 - 26566835<br>chr15: 20816922 - 20819714<br>chr15: 21161647 - 21164438<br>chr15: 28172919 - 28175710<br>chr15: 28224987 - 28227778<br>chr15: 26743427 - 26746216<br>chr15: 28480522 - 28483310<br>chr15: 30469559 - 30472350<br>chr15: 30521723 - 30524674<br>chr15: 28641921 - 28644874<br>chr15: 28693981 - 28696937<br>chr15: 88640040 - 88641226<br>chr15: 70746041 - 70747120<br>chr15: 73349895 - 73350972<br>chr15: 72148922 - 72149999	91.04<br>91.30<br>91.95<br>91.95<br>91.58<br>91.95<br>91.07<br>91.55<br>91.56<br>91.46<br>91.52<br>91.56<br>91.12<br>91.52<br>91.07<br>91.07<br>91.39<br>90.33<br>91.00<br>91.00	91.33	1

842	15q	212903	214599	1697	chr15: 32602604 - 32605056<br>chr15: 28176532 - 28178043<br>chr15: 28645530 - 28647040<br>chr15: 28478195 - 28479706<br>chr15: 30519551 - 30521065<br>chr15: 30466904 - 30468742<br>chr15: 28228917 - 28230435	90.31<br>90.00<br>90.00<br>90.00<br>90.00<br>90.87<br>90.84	90.29	1
843	15q	213931	215516	1586	chr22: 41507667 - 41509055	91.87	91.87	0
844	15q	214553	229500	14948	chr9: 128086775 - 128092943	90	90	0
845	15q	229301	233364	4064	chr15: 32595687 - 32600446	92.4	92.4	1
846	15q	233166	234260	1095	chr15: 82630934 - 82632099<br>chr15: 80799801 - 80800970<br>chr15: 82768162 - 82769333<br>chr15: 82845450 - 82846616<br>chr15: 32594623 - 32595648	90.27<br>90.27<br>90.27<br>90.27<br>91.48	90.51	1
847	15q	233655	239471	5817	chrY: 25947578 - 25952655<br>chrY: 24686029 - 24691110	91.43<br>91.45	91.44	0
848	15q	237263	238999	1737	chr15: 32590595 - 32592832<br>chr15: 82634169 - 82636141<br>chr15: 80451324 - 80462817<br>chr15: 80837868 - 80849351<br>chr15: 82671190 - 82683825<br>chr15: 83560288 - 83572931<br>chr15: 82841389 - 82843394<br>chr15: 82708569 - 82710578<br>chr15: 80526976 - 80528244<br>chr15: 80913507 - 80914775<br>chr15: 82911466 - 82912770<br>chr15: 80795775 - 80797035<br>chr15: 82764130 - 82765390	91.29<br>91.79<br>91.73<br>91.64<br>91.40<br>91.40<br>91.24<br>91.51<br>92.18<br>92.18<br>92.12<br>92.04<br>92.16	91.74	1
849	15q	237702	248990	11289	chrY: 25909036 - 25921556<br>chrY: 24717131 - 24729651	92.02<br>91.96	91.99	0
850	15q	239350	242355	3006	chr15: 82710941 - 82714269<br>chr15: 82907741 - 82911078<br>chr15: 82636529 - 82640276<br>chr15: 80791676 - 80795389<br>chr15: 82837278 - 82841026	92.97<br>92.30<br>91.46<br>91.62<br>91.46	91.96	1
851	15q	241976	244363	2388	chrY: 25955573 - 25958256<br>chrY: 24680429 - 24683110	93.67<br>93.67	93.67	0
852	15q	243592	247141	3550	chr15: 82715442 - 82719227	91.23	91.23	1
853	15q	247466	248990	1525	chr15: 80467769 - 80471182<br>chr15: 80854303 - 80857717<br>chr15: 82899233 - 82900478	90.59<br>90.28<br>90.43	90.43	1
854	15q	473217	475124	1908	chr9: 11773481 - 11775112	92.61	92.61	0
855	16p	1462	28780	27319	Xq: 29837 - 2524<br>Yq: 29837 - 2524	96.97<br>96.97	96.97	0
856	16p	1464	9529	8066	15q: 10069 - 2004<br>9p: 1894 - 9953<br>chr2: 114066302 - 114074366	97.79<br>98.08<br>98.13	98	0
857	16p	8082	9529	1448	19p: 1 - 1448	98	98	0
858	16p	17959	29051	11093	10p: 64762 - 74445	92.38	92.38	0
859	16p	17959	37573	19615	9q: 129597 - 113409<br>18p: 68156 - 84342	91.98<br>92.31	92.15	0
860	16p	48123	52702	4580	chr3: 14589148 - 14591139	91.94	91.94	0
861	16q	1705	13032	11328	17q: 19918 - 14511<br>5q: 15432 - 10026<br>6q: 9611 - 4197	96.51<br>96.27<br>96.72	96.5	0
862	16q	1705	5031	3327	19p: 196020 - 199397	93.59	93.59	0
863	16q	1705	3756	2052	5p: 618 - 2291	94.00	93.82	0
					4q: 7430 - 2781			

865	16q	1827	7774	5948	chr2: 114090474 - 114096529<br>19q: 16059 - 10016<br>22q: 19042 - 13006<br>1q: 11916 - 7032<br>4p: 11143 - 16488<br>18p: 88797 - 94488	94.03<br>94.22<br>93.97<br>93.07<br>92.61<br>93.45	93.56	0
866	16q	5038	7399	2362	1p: 281205 - 283465<br>6p: 91533 - 94526<br>19p: 182231 - 184131<br>3q: 59632 - 57730<br>11p: 111977 - 113879<br>9q: 24868 - 22273<br>7p: 71845 - 74440<br>8p: 105117 - 107714	95.08<br>92.95<br>95.40<br>95.01<br>95.01<br>95.10<br>95.32<br>94.95	94.85	0
867	16q	5897	7770	1874	2p: 4068 - 5924	91.53	91.53	0
868	16q	13817	57598	43782	chrY: 24760333 - 24810244<br>chrY: 25828452 - 25878360	96.04<br>96.05	96.05	0
869	16q	13817	30416	16600	chr1: 239500425 - 239517107	96.79	96.79	0
870	16q	13817	23961	10145	5q: 72329 - 62109<br>8p: 19652 - 29862<br>1p: 52507 - 62722<br>6q: 64494 - 54277	97.74<br>97.57<br>97.70<br>97.74	97.69	0
871	16q	13817	19869	6053	chr4: 119695468 - 119701610	97.38	97.38	0
872	16q	13817	14928	1112	chr1: 220402740 - 220403853	97.36	97.36	0
873	16q	15778	52766	36989	2q: 37420 - 79	97.64	97.64	0
874	16q	15806	36468	20663	chr1: 220404716 - 220425495	97.88	97.88	0
875	16q	19873	56395	36523	chr4: 119912346 - 119948842	96.99	96.99	0
876	16q	26485	57598	31114	chr10: 38775755 - 38806807	96.58	96.58	0
877	16q	27493	47361	19869	chr4: 165537042 - 165556842	95.66	95.66	0
878	16q	30092	64320	34229	20q: 46227 - 18783	97.43	97.43	0
879	16q	30356	57598	27243	7p: 91 - 27604<br>chr1: 239517108 - 239544133	98.38<br>96.91	97.64	0
880	16q	32910	64320	31411	6p: 62675 - 87328	97.58	97.58	0
881	16q	32910	57598	24689	19p: 113350 - 138324<br>5q: 151484 - 126807<br>11p: 43725 - 68406	98.19<br>98.57<br>98.18	98.31	0
882	16q	32910	50788	17879	8p: 86034 - 103892	97.66	97.66	0
883	16q	32910	43951	11042	6q: 133270 - 122211	98.12	98.12	0
884	16q	32910	36468	3559	1p: 116486 - 120044	97.92	97.92	0
885	16q	36614	56395	19782	chr1: 220425641 - 220445916	96.91	96.91	0
886	16q	36614	37793	1180	1p: 120190 - 121369	98.33	98.33	0
887	16q	41363	57598	16236	1p: 124946 - 141190	98.43	98.43	0
888	16q	42548	44495	1948	chr1: 224457827 - 224459772	96.91	96.91	0
889	16q	43247	57598	14352	3q: 14339 - 1	98.77	98.77	0
890	16q	44840	57598	12759	chr1: 224467506 - 224480262	96.67	96.67	0
891	16q	48412	50258	1847	chr7: 61805021 - 61806472<br>chr1: 193837523 - 193839718	91.98<br>92.15	92.06	0
892	16q	48423	57415	8993	chr7: 55985248 - 55994150<br>chr7: 45409991 - 45420987	94.69<br>94.60	94.64	0
893	16q	48424	53289	4866	chr7: 55420849 - 55425758	91.35	91.35	0
894	16q	52440	57598	5159	chr7: 127672739 - 127677904	96.4	96.4	0
895	16q	52457	56847	4391	chr7: 62456759 - 62460740<br>chr7: 56432166 - 56436149<br>chr7: 39391514 - 39396354<br>chr7: 55363641 - 55368463<br>chr1: 219029846 - 219033728<br>chr7: 51009264 - 51014002	94.27<br>94.10<br>93.54<br>94.12<br>96.93<br>94.19	94.52	0
896	16q	53721	56391	2671	chr4: 120680507 - 120683166	94.69	94.69	0
897	16q	62611	131365	68755	3q: 87591 - 17517<br>7p: 31783 - 101860	98.27<br>98.54	98.41	0
898	16q	62611	119454	56844	11p: 71805 - 128459	98.42	98.42	0
899	16q	62611	105868	43258	19p: 142094 - 185461	98.5	98.5	0
900	16q	62611	96795	34185	5q: 189359 - 154639	98.22	98.22	0
901	16q	62611	70142	7532	1p: 151063 - 158591	98.44	98.44	0

**902**	16q	62611	66298	3688	1p: 144248 - 147924<br>1p: 147657 - 151331	98.57<br>98.05	98.31	0
**903**	16q	62612	63909	1298	chr7: 45403637 - 45404932<br>chr1: 224460233 - 224461546<br>chr7: 51020104 - 51021398	96.38<br>95.07<br>95.75	95.73	0
**904**	16q	62613	65222	2610	chr4: 119904851 - 119907443<br>chr4: 120688005 - 120690600<br>chr10: 38769794 - 38772390<br>chr7: 39386597 - 39389187<br>chr7: 127667204 - 127669796<br>chr7: 55375429 - 55378337<br>chr7: 56005902 - 56008498<br>chr1: 219039323 - 219041921<br>chr1: 239547046 - 239549639<br>chr1: 220447184 - 220449733	96.33<br>96.08<br>96.70<br>95.95<br>97.89<br>96.62<br>96.56<br>96.63<br>97.34<br>98.06	96.82	0
**905**	16q	66125	68934	2810	chr7: 56015725 - 56026447	90.67	90.67	0
**906**	16q	68404	100242	31839	chr1: 219047352 - 219081989	97.26	97.26	0
**907**	16q	68407	102624	34218	chr10: 38729667 - 38764372<br>chr4: 119870679 - 119899413<br>chr4: 120696041 - 120730560<br>chr1: 239555083 - 239589627	96.86<br>97.28<br>96.34<br>97.48	96.99	0
**908**	16q	68409	94345	25937	chr1: 220455168 - 220481396	97.57	97.57	0
**909**	16q	68409	89625	21217	chr7: 39360178 - 39381110	92.88	92.88	0
**910**	16q	68409	70142	1734	chr7: 127660030 - 127661758	94.82	94.82	0
**911**	16q	68411	82585	14175	chr7: 56013907 - 56027313	92.43	92.43	0
**912**	16q	68433	72644	4212	chr7: 45392682 - 45396890	95.86	95.86	0
**913**	16q	69778	75519	5742	chr7: 62412186 - 62417768<br>chr7: 56475714 - 56481294<br>chr7: 51029490 - 51036010	90.73<br>90.73<br>92.87	91.44	0
**914**	16q	69778	72644	2867	chr7: 55385064 - 55388239	93.03	93.03	0
**915**	16q	72392	102624	30233	1p: 160844 - 191186	98.33	98.33	0
**916**	16q	72392	84501	12110	chr7: 127645453 - 127657799	96.24	96.24	0
**917**	16q	72392	81719	9328	chr7: 65587537 - 65593232	91.29	91.29	0
**918**	16q	72392	75264	2873	chr11: 50000552 - 50003428<br>chr7: 64516409 - 64519292	90.00<br>91.46	90.73	0
**919**	16q	75881	81420	5540	chr7: 64511343 - 64515807	90.86	90.86	0
**920**	16q	81201	131367	50167	9q: 51184 - 834	98.62	98.62	0
**921**	16q	81485	102624	21140	chr7: 45368447 - 45389874	96.36	96.36	0
**922**	16q	83754	85220	1467	chr7: 64507178 - 64508843	92.35	92.35	0
**923**	16q	84238	89681	5444	chr7: 56029594 - 56035175	92.72	92.72	0
**924**	16q	85455	86845	1391	chr7: 65561411 - 65562799	90.72	90.72	0
**925**	16q	90566	91685	1120	chr7: 56036051 - 56037182	92.33	92.33	0
**926**	16q	91646	100242	8597	chr7: 127636388 - 127644996	95.54	95.54	0
**927**	16q	94211	95696	1486	chr7: 75222310 - 75223815<br>chr7: 65540950 - 65542445	92.31<br>94.03	93.17	0
**928**	16q	96620	102624	6005	chr11: 50209890 - 50211062	90	90	0
**929**	16q	97194	98396	1203	chr7: 63775170 - 63776243<br>chr7: 64469009 - 64470133	90.25<br>90.32	90.28	0
**930**	16q	98838	102624	3787	chr1: 220481705 - 220485566	97.22	97.22	0
**931**	16q	102588	105715	3128	8p: 104622 - 108356<br>10q: 21274 - 18113<br>18p: 91554 - 94731<br>1q: 12159 - 9311<br>6p: 91944 - 94769<br>19q: 16701 - 13461<br>22q: 19684 - 16443<br>chr2: 114093933 - 114097169<br>4q: 8783 - 6261<br>4p: 14618 - 17140	97.34<br>93.87<br>94.70<br>93.14<br>93.07<br>96.79<br>97.11<br>97.04<br>94.27<br>94.27	95.16	0
					21q: 19448 - 18188			

**935**	16q	131390	145989	14600	9q: 108513 - 80928<br>10p: 32239 - 59811<br>18p: 36632 - 63394	95.40<br>94.97<br>95.73	95.37	0
**936**	16q	131398	133440	2043	4q: 123167 - 121031<br>1p: 286972 - 289118	93.70<br>92.74	93.22	0
**937**	16q	131430	138853	7424	3q: 119439 - 113998<br>chr12: 36865089 - 36876210<br>chr12: 34199947 - 34211062	94.43<br>90.88<br>91.90	92.4	0
**938**	16q	133608	138299	4692	4q: 121040 - 109513<br>chr1: 223993648 - 224001048	92.66<br>90.56	91.61	0
**939**	16q	137312	138848	1537	chr3: 75834770 - 75836658<br>chrY: 10552604 - 10554424<br>chrY: 18929039 - 18930242<br>chrY: 18075794 - 18076997	90.47<br>91.38<br>90.00<br>90.00	90.46	0
**940**	16q	149733	171180	21448	chr5: 23540912 - 23563027	93.82	93.82	0
**941**	16q	260295	278932	18638	chr17: 20447480 - 20450998	94.73	94.73	0
**942**	16q	292277	295725	3449	chr6: 90066806 - 90068310	90.79	90.79	0
**943**	16q	292549	293561	1013	chr19: 6446216 - 6447222<br>chr6: 3097672 - 3098689	92.00<br>91.00	91.5	0
**944**	16q	292551	293568	1018	chr6: 3168560 - 3169577	92	92	0
**945**	17p	727	18220	17494	7p: 78903 - 88088<br>3q: 73851 - 65850<br>9q: 37610 - 29318<br>16q: 117597 - 109676	97.52<br>97.23<br>97.52<br>97.52	97.45	0
**946**	17p	727	29146	28420	11p: 119534 - 136823	98.46	98.46	0
**947**	17q	3110	36125	33016	5q: 32068 - 1037	98.75	98.75	0
**948**	17q	3110	17406	14297	chr2: 114079703 - 114093488<br>22q: 16000 - 2133<br>10q: 17677 - 3829<br>21q: 17747 - 4338<br>19p: 189683 - 199081<br>8p: 112649 - 123045	95.87<br>95.90<br>95.43<br>96.10<br>95.88<br>94.76	95.66	0
**949**	17q	4319	17504	13186	19q: 13018 - 639	95.82	95.82	0
**950**	17q	8455	16452	7998	1q: 8975 - 1764	95.61	95.61	0
**951**	17q	8455	12292	3838	chr3: 75756447 - 75760281	93.52	93.52	0
**952**	17q	8545	43948	35404	6q: 33611 - 1	98.25	98.25	0
**953**	17q	12166	17506	5341	4p: 7719 - 14175	92.28	92.28	0
**954**	17q	14511	19918	5408	16q: 13032 - 1705	97.01	97.01	0
**955**	17q	14511	16179	1669	5p: 618 - 2291	94.7	94.7	0
**956**	17q	14875	17506	2632	4q: 5818 - 2781<br>18p: 88797 - 91117	92.10<br>93.44	92.77	0
**957**	17q	19570	36125	16556	1p: 1 - 15016	97.97	97.97	0
**958**	17q	36022	43948	7927	1p: 15044 - 25292<br>5q: 41588 - 32148	98.00<br>98.56	98.28	0
**959**	17q	125910	128514	2605	chr8: 86742308 - 86747680<br>chr8: 86860045 - 86865693<br>chr8: 86872236 - 86877884<br>chr8: 86884426 - 86889798<br>chr8: 86896340 - 86901988	98.41<br>98.20<br>97.90<br>97.90<br>98.41	98.16	0
**960**	17q	125910	126941	1032	chr8: 86754222 - 86758297	98.29	98.29	0
**961**	17q	126802	128514	1713	chr8: 86851789 - 86853502	96.23	96.23	0
**962**	18p	1159	2971	1813	chr2: 132391011 - 132392834<br>chr22: 14840436 - 14842245<br>chr14: 18429691 - 18431501	92.18<br>91.20<br>91.20	91.53	0
**963**	18p	1669	6849	5181	1p: 283791 - 286519	93.24	93.24	0
**964**	18p	3342	15324	11983	chr1: 224019203 - 224035343	91.13	91.13	0
**965**	18p	3342	44090	40749	chr12: 34145401 - 34211062	91.07	91.07	0
**966**	18p	3866	7707	3842	chr2: 132394175 - 132398023<br>chr22: 14843625 - 14847482<br>chr14: 18424456 - 18428301	94.20<br>93.77<br>93.94	93.97	0
**967**	18p	4415	79293	74879	10p: 1 - 74443	96.24	96.24	0
**968**	18p	14978	22991	8014	chrY: 18957030 - 18965446	90.99	90.99	0
**969**	18p	14978	35863	20886	chrY: 18040585 - 18065337	90.91	90.91	0
**970**	18p	15346	44090	28745	3q: 119439 - 99337	95.12	95.12	0
**971**	18p	15346	66163	50818	9q: 111417 - 63045	95.48	95.48	0

**972**	18p	17567	38676	21110	1p: 286972 - 301421	93.47	93.47	0
**973**	18p	22287	23484	1198	chrY: 10567257 - 10568441	91.33	91.33	0
**974**	18p	33215	38676	5462	4q: 126874 - 121031	93.48	93.48	0
**975**	18p	33905	35863	1959	chrY: 18940699 - 18942639	90	90	0
**976**	18p	36956	44089	7134	16q: 138858 - 131398<br>chr12: 36865089 - 36875578	95.83<br>90.74	93.28	0
**977**	18p	38560	43990	5431	4q: 121040 - 109513	92.98	92.98	0
**978**	18p	42541	44089	1549	chrY: 10552604 - 10554424<br>chr3: 75834770 - 75835986	90.44<br>90.72	90.58	0
**979**	18p	62317	63700	1384	Xq: 16188 - 15056<br>Yq: 16188 - 15056	90.00<br>90.00	90	0
**980**	18p	68156	79029	10874	Xq: 29838 - 19014<br>Yq: 29838 - 19014	92.75<br>92.75	92.75	0
**981**	18p	68156	84342	16187	16p: 17959 - 37573	92.34	92.34	0
**982**	18p	68156	87912	19757	9q: 133085 - 113409	97.59	97.59	0
**983**	18p	88797	91058	2262	8p: 121350 - 123046<br>17q: 17506 - 15235<br>6q: 7195 - 4927<br>5q: 13021 - 10750<br>19p: 196746 - 199397	93.94<br>93.44<br>93.28<br>93.44<br>93.32	93.48	0
**984**	18p	88797	92815	4019	21q: 19448 - 15450	93.54	93.54	0
**985**	18p	88797	95052	6256	16q: 7774 - 2435<br>chr2: 114091199 - 114097183<br>19q: 16715 - 10741<br>4p: 11867 - 17154<br>1q: 12573 - 7757<br>22q: 19698 - 13731<br>5p: 1348 - 2421	93.74<br>93.93<br>94.02<br>92.96<br>92.37<br>93.61<br>91.99	93.23	0
**986**	18p	88797	102436	13640	10q: 26302 - 15394<br>4q: 15653 - 3505	95.92<br>93.65	94.78	0
**987**	18p	91285	93377	2093	1p: 281205 - 283465<br>16q: 104535 - 102637	94.44<br>94.68	94.56	0
**988**	18p	91495	94967	3473	6p: 91533 - 94769<br>19p: 182231 - 185064<br>3q: 60982 - 57730<br>9q: 25525 - 22273<br>11p: 111977 - 115229<br>7p: 71845 - 75097<br>8p: 105117 - 107957	92.31<br>94.43<br>94.38<br>94.34<br>94.24<br>94.34<br>94.66	94.1	0
**989**	18p	93350	94731	1382	2p: 5103 - 6171	92.46	92.46	0
**990**	18p	102640	118325	15686	chr18: 16777494 - 16793498	97.27	97.27	1
**991**	19p	1	1448	1448	16p: 8082 - 9529<br>Xq: 10579 - 9133<br>Yq: 10579 - 9133	98.00<br>98.35<br>98.35	98.23	0
**992**	19p	1	19667	19667	9p: 8506 - 27840<br>chr2: 114048111 - 114067751	98.81<br>98.03	98.42	0
**993**	19p	1	109077	109077	15q: 116787 - 8621	98.47	98.47	0
**994**	19p	15718	19667	3950	12p: 1705 - 5645	96.98	96.98	0
**995**	19p	51596	143803	92208	6p: 1 - 87328	98.18	98.18	0
**996**	19p	68278	116908	48631	1p: 70481 - 120046	98.63	98.63	0
**997**	19p	68278	131513	63236	8p: 37604 - 103892	98.38	98.38	0
**998**	19p	68945	124695	55751	6q: 133270 - 72955	98.45	98.45	0
**999**	19p	68945	176313	107369	5q: 189359 - 80764	98.53	98.53	0
**1000**	19p	69632	185488	115857	11p: 1 - 115235	98.63	98.63	0
**1001**	19p	112806	138324	25519	chrY: 25847059 - 25878360<br>chrY: 24760333 - 24791629	95.75<br>95.76	95.75	0
**1002**	19p	112806	144474	31669	20q: 46227 - 21029<br>chr1: 239518849 - 239549639<br>chr10: 38769794 - 38800932<br>chr4: 119904851 - 119936314	97.53<br>97.05<br>96.67<br>96.83	97.02	0
					7p: 2360 - 75103<br>16q: 105895 - 32910	98.42		

1007	19p	117052	118231	1180	1p: 120190 - 121369	98.03	98.03	0
1008	19p	117052	137121	20070	chr1: 220425641 - 220445916	97.07	97.07	0
1009	19p	121798	145776	23979	1p: 124946 - 147924	98.85	98.85	0
1010	19p	123290	125228	1939	chr1: 224457838 - 224459772	97.34	97.34	0
1011	19p	123991	185488	61498	3q: 60988 - 1	98.72	98.72	0
1012	19p	125582	138324	12743	chr1: 224467506 - 224480262	96.86	96.86	0
1013	19p	129136	138027	8892	chr7: 55985248 - 55994150	94.6	94.6	0
1014	19p	129139	131162	2024	chr7: 61805021 - 61806480<br>chr1: 193837523 - 193839718<br>chr7: 55420846 - 55423188	92.37<br>93.09<br>92.44	92.63	0
1015	19p	129159	135874	6716	chr7: 45414266 - 45420987	94.82	94.82	0
1016	19p	133122	144698	11577	chr7: 127667204 - 127677946	96.6	96.6	0
1017	19p	133141	135874	2734	chr7: 51009103 - 51011866<br>chr7: 55363599 - 55366358<br>chr7: 56432157 - 56434907<br>chr7: 39393644 - 39396387<br>chr7: 62457999 - 62460749<br>chr1: 219029846 - 219032485	93.00<br>94.00<br>94.00<br>93.00<br>94.00<br>97.00	94.17	0
1018	19p	134445	144698	10254	chr4: 120680507 - 120690600	94.83	94.83	0
1019	19p	135964	137723	1760	chr1: 219032574 - 219033728<br>chr7: 56434997 - 56436149<br>chr7: 55366448 - 55368463<br>chr7: 62455575 - 62457909<br>chr7: 51011956 - 51014002<br>chr7: 39391514 - 39393554	96.86<br>94.15<br>94.48<br>93.77<br>94.76<br>95.02	94.84	0
1020	19p	135964	143392	7429	chr7: 45403637 - 45414176	95.23	95.23	0
1021	19p	138029	143392	5364	chr1: 224460233 - 224466670	94.42	94.42	0
1022	19p	142094	145776	3683	1p: 147657 - 151331	98.39	98.39	0
1023	19p	142094	149620	7527	1p: 151063 - 158591	98.58	98.58	0
1024	19p	142097	143392	1296	chr7: 51020104 - 51021398	95.53	95.53	0
1025	19p	142098	144698	2601	chr1: 219039323 - 219041921<br>chr7: 55375429 - 55378337<br>chr7: 56005902 - 56008498<br>chr7: 39386597 - 39389187<br>chr1: 220447184 - 220449733	96.72<br>96.61<br>96.65<br>96.16<br>98.20	96.87	0
1026	19p	145508	162092	16585	chr7: 56005902 - 56027313	92.91	92.91	0
1027	19p	145508	179758	34251	chr1: 219039323 - 219081989	97.15	97.15	0
1028	19p	145508	182218	36711	chr1: 239547046 - 239589627<br>chr4: 120688005 - 120730560	97.34<br>96.40	96.87	0
1029	19p	145555	148263	2709	chr7: 55375429 - 55383906<br>chr7: 56015725 - 56026447	94.40<br>90.67	92.53	0
1030	19p	147884	182218	34335	chr10: 38729667 - 38764372<br>chr4: 119870679 - 119899413	96.98<br>97.20	97.09	0
1031	19p	147887	149620	1734	chr7: 127660030 - 127661758	94.57	94.57	0
1032	19p	147887	169131	21245	chr7: 39360178 - 39381110	92.89	92.89	0
1033	19p	147887	173860	25974	chr1: 220455168 - 220481396	97.73	97.73	0
1034	19p	147911	152151	4241	chr7: 45392682 - 45396890	95.86	95.86	0
1035	19p	149256	152151	2896	chr7: 55385064 - 55388239	92.87	92.87	0
1036	19p	149256	155026	5771	chr7: 56475714 - 56481294<br>chr7: 62412186 - 62417768<br>chr7: 51029490 - 51036010	90.50<br>90.50<br>92.79	91.26	0
1037	19p	151899	154772	2874	chr11: 50000552 - 50003428<br>chr7: 64516409 - 64519292	90.00<br>91.46	90.73	0
1038	19p	151899	161226	9328	chr7: 65587537 - 65593232	91.29	91.29	0
1039	19p	151899	164008	12110	chr7: 127645453 - 127657799	95.93	95.93	0
1040	19p	151899	182218	30320	1p: 160844 - 191186	98.41	98.41	0
1041	19p	155388	160927	5540	chr7: 64511343 - 64515807	91.43	91.43	0
1042	19p	160708	185488	24781	9q: 25531 - 834	98.24	98.24	0
1043	19p	160992	182218	21227	chr7: 45368447 - 45389874	96.35	96.35	0
1044	19p	163261	164727	1467	chr7: 64507178 - 64508843	92.01	92.01	0
1045	19p	163745	169187	5443	chr7: 56029594 - 56035175	92.74	92.74	0
1046	19p	164962	166351	1390	chr7: 65561411 - 65562799	90.72	90.72	0
1047	19p	170070	171196	1127	chr7: 56036051 - 56037182	92.98	92.98	0
1048	19p	171157	179758	8602	chr7: 127636388 - 127644996	95.2	95.2	0

**1049**	19p	173726	175216	1491	chr7: 75222310 - 75223826<br>chr7: 65540948 - 65542445	92.69<br>94.40	93.55	0
**1050**	19p	176709	178070	1362	chr7: 63774859 - 63776250<br>chr7: 64469009 - 64470133	90.56<br>90.16	90.36	0
**1051**	19p	178356	182218	3863	chr1: 220481705 - 220485566	96.99	96.99	0
**1052**	19p	181756	198474	16719	8p: 104641 - 123046	96.74	96.74	0
**1053**	19p	182231	183859	1629	1p: 281741 - 283465<br>16q: 7077 - 5156<br>21q: 19448 - 18188	96.51<br>95.40<br>96.00	95.97	0
**1054**	19p	182254	185274	3021	10q: 21268 - 18113<br>6p: 91944 - 94765<br>1q: 12155 - 9311<br>18p: 91554 - 94727<br>19q: 16721 - 13461<br>22q: 19704 - 16443<br>4p: 14618 - 17154<br>4q: 8797 - 6261<br>chr2: 114093933 - 114097189<br>2p: 3251 - 6167	93.66<br>93.04<br>93.19<br>94.67<br>96.95<br>96.88<br>93.85<br>93.96<br>97.03<br>91.52	94.47	1
**1055**	19p	189683	193721	4039	6q: 145794 - 139446	92.13	92.13	0
**1056**	19p	189683	199296	9614	5q: 13019 - 1037<br>17q: 17504 - 3110<br>10q: 17976 - 3829<br>21q: 18046 - 4338<br>6p: 91217 - 91808<br>22q: 16306 - 2133<br>chr2: 114079703 - 114093794	95.55<br>95.45<br>96.26<br>96.04<br>95.43<br>96.26<br>96.18	95.88	0
**1057**	19p	190896	199385	8490	19q: 13324 - 639	96.04	96.04	1
**1058**	19p	193592	199283	5692	4p: 7699 - 14477<br>6q: 7193 - 1847<br>1q: 9172 - 4691	91.97<br>94.05<br>94.70	93.57	0
**1059**	19p	196019	197721	1703	5p: 617 - 2291	94.58	94.58	0
**1060**	19p	196022	199383	3362	16q: 5015 - 1705<br>4q: 6120 - 2781	93.60<br>92.19	92.89	0
**1061**	19p	196746	199380	2635	18p: 88797 - 91414	93.17	93.17	0
**1062**	19q	639	16745	16107	10q: 21689 - 5039<br>22q: 19746 - 3342<br>chr2: 114080913 - 114097231	96.03<br>98.00<br>98.32	97.45	0
**1063**	19q	639	14724	14086	21q: 19448 - 5548	97.17	97.17	0
**1064**	19q	639	12948	12310	8p: 113859 - 123046<br>5q: 13019 - 2247<br>17q: 17504 - 4319<br>19p: 190896 - 199397	93.81<br>95.29<br>95.21<br>96.04	95.09	1
**1065**	19q	3954	16715	12762	1q: 12573 - 1764	94.48	94.48	0
**1066**	19q	3954	7796	3843	chr3: 75756447 - 75760281	93.52	93.52	0
**1067**	19q	4044	13018	8975	6q: 7193 - 1	94.64	94.64	0
**1068**	19q	7670	16713	9044	13q: 2231 - 1395<br>4p: 7719 - 17154	91.92<br>92.68	92.3	0
**1069**	19q	7670	10865	3196	6q: 147506 - 145671	90.65	90.65	0
**1070**	19q	10198	16551	6354	5p: 617 - 2423<br>16q: 7774 - 1705<br>4q: 8797 - 2781<br>18p: 88797 - 95146	93.63<br>94.07<br>92.78<br>94.01	93.62	0
**1071**	19q	13190	15273	2084	1p: 281205 - 283465<br>16q: 104535 - 102637	95.55<br>97.05	96.3	0
**1072**	19q	13438	16749	3312	6p: 91533 - 95228<br>19p: 182231 - 185488<br>3q: 61030 - 57730<br>11p: 111977 - 115277<br>7p: 71845 - 75145<br>9q: 25573 - 22273	92.75<br>96.96<br>96.93<br>96.79<br>96.66<br>96.66	95.65	1
					chr2: 110574910 - 110576267			

1075	19q	208689	210500	1812	chr8: 81374441 - 81374984	92.4	92.4	0
1076	19q	208781	215337	6557	chrX: 6199833 - 6200483<br>chr6: 116684955 - 116685502	93.51<br>95.79	94.65	0
1077	19q	209984	215339	5356	chr21: 33775923 - 33776376	91.21	91.21	0
1078	20p	404459	408223	3765	chr11: 11323729 - 11324230	98.37	98.37	0
1079	20p	409322	429307	19986	chr11: 11324227 - 11325163	99.16	99.16	0
1080	20q	3777	18388	14612	12p: 27892 - 42389	97.97	97.97	0
1081	20q	18783	45040	26258	chrY: 24761547 - 24793892<br>chrY: 25844800 - 25877147	96.02<br>95.99	96	0
1082	20q	18783	41420	22638	2q: 37420 - 14408	97.56	97.56	0
1083	20q	18783	19856	1074	chr1: 220419136 - 220420213	98	98	0
1084	20q	18785	36084	17300	chr4: 165539678 - 165556918	95.44	95.44	0
1085	20q	18888	46226	27339	chr4: 119905733 - 119938569<br>16q: 64320 - 30092<br>chr10: 38770686 - 38803195<br>7p: 91 - 33495<br>chr1: 239517108 - 239548748	97.03<br>97.60<br>96.69<br>97.49<br>97.48	97.26	0
1086	20q	21028	50897	29870	6p: 62132 - 91344<br>6q: 139561 - 121665<br>8p: 85490 - 112773	98.23<br>97.49<br>97.28	97.67	0
1087	20q	21028	46225	25198	5q: 156343 - 126263<br>19p: 112806 - 143803<br>11p: 43183 - 73516	97.71<br>97.70<br>97.75	97.72	0
1088	20q	21307	25147	3841	1p: 115941 - 120046<br>chr1: 220421937 - 220425497	97.78<br>97.94	97.86	0
1089	20q	25288	35880	10593	chr1: 220425641 - 220436743	97.29	97.29	0
1090	20q	25288	27704	2417	1p: 120190 - 122607	98.77	98.77	0
1091	20q	30035	35880	5846	1p: 124946 - 130821	96.54	96.54	0
1092	20q	31196	33132	1937	chr1: 224457838 - 224459772	97.34	97.34	0
1093	20q	31895	46227	14333	3q: 19226 - 1	98	98	0
1094	20q	33511	45818	12308	chr1: 224460233 - 224480262	96.9	96.9	0
1095	20q	37052	38981	1930	chr1: 193837523 - 193838976<br>chr7: 61805021 - 61807375	93.19<br>91.70	92.44	0
1096	20q	37057	46223	9167	chr1: 220437912 - 220448841<br>chr7: 55985248 - 56007603<br>1p: 131997 - 145957<br>chr7: 45403235 - 45420987	97.49<br>95.09<br>97.95<br>95.12	96.41	0
1097	20q	38376	41943	3568	chr7: 55422123 - 55425758	91.58	91.58	0
1098	20q	41068	45827	4760	chr7: 51009103 - 51021398<br>chr7: 62456760 - 62460749<br>chr7: 56432157 - 56436148<br>chr7: 39387491 - 39396387<br>chr7: 127668095 - 127677946<br>chr7: 55363599 - 55377459<br>chr1: 219029845 - 219041033	93.53<br>94.16<br>93.99<br>93.67<br>96.62<br>94.25<br>96.93	94.74	0
1099	20q	42375	46227	3853	chr4: 120680507 - 120689715	94.64	94.64	0
1100	20q	398145	405831	7687	chr8: 23391299 - 23395680	92.14	92.14	0
1101	20q	442754	444018	1265	12q: 456964 - 455241	90	90	0
1102	21q	2734	19448	16715	22q: 17706 - 532	97.22	97.22	0
1103	21q	4338	19448	15111	chr2: 114079703 - 114095193<br>10q: 19372 - 3829	96.91<br>96.59	96.75	0
1104	21q	4338	17671	13334	5q: 13019 - 1037<br>17q: 17504 - 3110<br>19p: 189683 - 199397<br>8p: 112649 - 123046	95.05<br>95.52<br>95.57<br>93.76	94.97	0
1105	21q	4338	5796	1459	6q: 140926 - 139446	91.85	91.85	0
1106	21q	5548	19448	13901	19q: 14724 - 639	97.27	97.27	0
1107	21q	8673	19448	10776	1q: 10576 - 1764	94.96	94.96	0
1108	21q	8673	12505	3833	chr3: 75756447 - 75760281	93.52	93.52	0
1109	21q	8764	17747	8984	6q: 7193 - 1	94.37	94.37	0
1110	21q	12379	18851	6473	4p: 7719 - 15281	91.64	91.64	0
1111	21q	12379	15574	3196	6q: 147506 - 145671	90.65	90.65	0
1112	21q	14724	21380	6657	5p: 617 - 17258	92.39	92.39	0

**1113**	21q	14968	19249	4282	16q: 6418 - 1705<br>4q: 6924 - 2781<br>18p: 88797 - 92815	93.54<br>91.68<br>93.35	92.86	0
**1114**	21q	18082	19448	1367	1p: 281205 - 283004<br>6p: 91533 - 93211<br>7p: 71847 - 73106<br>19p: 182233 - 183494<br>8p: 105118 - 106379<br>16q: 103898 - 102639<br>9q: 23534 - 22275<br>11p: 111979 - 113241<br>3q: 58994 - 57732	95.19<br>93.18<br>95.69<br>95.69<br>95.70<br>95.69<br>95.69<br>96.13<br>96.13	95.45	0
**1115**	21q	18444	20521	2078	2p: 3280 - 17320	92.63	92.63	0
**1116**	21q	20229	21380	1152	chr3: 197230489 - 197231637	92	92	0
**1117**	21q	23660	26137	2478	2p: 89270 - 91971	93.21	93.21	0
**1118**	22q	532	17706	17175	21q: 19448 - 2734	97.1	97.1	0
**1119**	22q	2133	65623	63491	chr2: 114079703 - 114144877	97.94	97.94	0
**1120**	22q	2133	19680	17548	10q: 21671 - 3829	95.86	95.86	0
**1121**	22q	2133	15931	13799	17q: 17504 - 3110<br>5q: 13019 - 1037<br>19p: 189683 - 199397<br>8p: 112649 - 123046	95.33<br>94.98<br>96.25<br>93.54	95.03	0
**1122**	22q	2133	3743	1611	6q: 141080 - 139446	91.39	91.39	0
**1123**	22q	3342	19684	16343	19q: 16701 - 639	98.05	98.05	0
**1124**	22q	6932	19684	12753	1q: 12559 - 1764	94.15	94.15	0
**1125**	22q	6932	10786	3855	chr3: 75756447 - 75760281	93.52	93.52	0
**1126**	22q	7022	16000	8979	6q: 7193 - 1	94.18	94.18	0
**1127**	22q	10660	19682	9023	13q: 2219 - 1395<br>4p: 7719 - 17140	92.13<br>92.45	92.29	0
**1128**	22q	10660	13855	3196	6q: 147506 - 145671	90.65	90.65	0
**1129**	22q	13188	19523	6336	5p: 617 - 2407<br>16q: 7774 - 1705<br>4q: 8783 - 2781<br>18p: 88797 - 95132	93.37<br>93.97<br>92.15<br>93.63	93.28	0
**1130**	22q	16172	18256	2085	1p: 281205 - 283465<br>16q: 104535 - 102637	95.22<br>97.24	96.23	0
**1131**	22q	16423	19683	3261	6p: 91533 - 95168<br>19p: 182231 - 185461<br>7p: 71845 - 75083<br>11p: 111977 - 115215<br>3q: 60968 - 57730<br>9q: 25511 - 22273<br>8p: 105117 - 108356<br>2p: 3280 - 6570	92.49<br>97.00<br>96.93<br>97.02<br>97.02<br>96.93<br>96.76<br>92.00	95.77	0
**1132**	22q	338214	359132	20919	chr8: 56523161 - 56528801	91.25	91.25	0
**1133**	22q	345476	359332	13857	chr1: 11872093 - 11875082	91.1	91.1	0
**1134**	22q	363588	375954	12367	chr21: 14300639 - 14310578	91.52	91.52	0
**1135**	22q	363588	375954	12367	chr18: 14131836 - 14141611	90.72	90.72	0
**1136**	Xp_Yp	114316	123873	9558	chr15: 20004096 - 20015923	92.15	92.15	0
**1137**	Xq	2524	29837	27314	16p: 1462 - 28780	96.97	96.97	0
**1138**	Xq	2526	10579	8054	9p: 1894 - 9953<br>15q: 10069 - 2004<br>chr2: 114066302 - 114074366	98.23<br>97.73<br>97.86	97.94	0
**1139**	Xq	9133	10579	1447	19p: 1 - 1448	98.34	98.34	0
**1140**	Xq	19014	29938	10925	9q: 123870 - 113409<br>18p: 68156 - 79029<br>10p: 64762 - 74476	92.89<br>92.76<br>92.26	92.64	0
**1141**	Xq	419175	420323	1149	chrX: 154128108 - 154129278	94.66	94.66	1
**1142**	Yq	393125	395835	2711	chr16: 33448552 - 33451269	94.89	94.89	0
					chr2: 96049906 - 96052677	93.51		
					chr1: 145641470 - 145642988			

**1146**	Yq	337518	351120	13603	chr10: 127580506 - 127594372	93.8	93.8	0
**1147**	Yq	331072	356227	25156	chr10: 127573354 - 127599628	94.54	94.54	0
**1148**	Yq	19014	29938	10925	9q: 123870 - 113409<br>18p: 68156 - 79029<br>10p: 64762 - 74476	92.89<br>92.76<br>92.26	92.64	0
					chr2: 114066302 - 114074366			
