# Supplementary material for: Human subtelomeric duplicon structure and organization
Source: Genome Biol. 2007 Jul 30;8(7):R151. doi: 10.1186/gb-2007-8-7-r151 (PMC2323237; doi:10.1186/gb-2007-8-7-r151)
Supplement: Additional data file 4 — This table shows the numbers of duplicon modules defined per subtelomere. The complete list of these modules is included in Additional data file 3. The 'subtelomeric' column shows the total number of modules for each subtelomere region (since each module is defined by a set of subtelomeric coordinates). The 'non-subtelomeric' column lists the subset of these modules with homology to duplicated regions that lie outside the subtelomeres. A comparison of these non-subtelomeric duplicons to the subtelomeric copies is included in Figure 3 and in Additional data file 5. The 'intra-chromosomal' column indicates the subset of modules with homology to a different region on the same chromosome. [file gb-2007-8-7-r151-S4.pdf]

Additional Data File 4. Modules defined by similarity to human subtelomeric DNA.

	Sub telomeric	Non-subtelomeric	Intra-chromosomal
**1p**	97	68	27
**1q**	15	4	2
**2p**	14	3	1
**2q**	31	23	
**3p**	5	5	5
**3q**	84	68	2
**4p**	17	5	1
**4q**	39	12	1
**5p**	24	15	4
**5q**	80	55	4
**6p**	33	18	1
**6q**	35	13	1
**7p**	67	44	27
**7q**	1	1	
**8p**	39	14	
**8q**	3	3	
**9p**	22	18	15
**9q**	42	24	
**10p**	16	10	
**10q**	40	12	
**11p**	67	46	2
**11q**	2	2	
**12p**	3	1	
**12q**	1		
**13q**	8	2	
**14q**	29	29	13
**15q**	40	35	20
**16p**	6	2	
**16q**	84	54	
**17p**	2		
**17q**	15	5	
**18p**	29	12	1
**19p**	71	45	2
**19q**	16	7	2
**20p**	2	2	
**20q**	22	14	
**21q**	16	3	
**22q**	18	6	
**Xp_Yp**	1	1	
**Xq**	5	2	1
**Yq**	10	7	1
**Total**	**1151**	**690**	**133**
