# Supplementary material for: Human subtelomeric duplicon structure and organization
Source: Genome Biol. 2007 Jul 30;8(7):R151. doi: 10.1186/gb-2007-8-7-r151 (PMC2323237; doi:10.1186/gb-2007-8-7-r151)
Supplement: Additional data file 48 — This table shows blocks of modules that occur exclusively in subtelomere regions. The first column gives an identifier for each block. The next three columns (query sequence) give the subtelomeric location that defines the block (which will consist of one or more adjacent modules). For completeness, in some cases aligned sequences have been included in these blocks even though they fell below thresholds for module definition. The percent identity of the chained alignments between the sequences is indicated (excluding masked sequence). Named genes/gene families that have transcripts matching part or all of the respective duplicon blocks are listed in the last column. Block 7 is the D4Z4 tandem repeat on the 4q and 10q subtelomeres, for which no percent identity is calculated because of the very large number and diverse percent identities of the BLAST alignments among tandem D4Z4 repeats. [file gb-2007-8-7-r151-S48.pdf]

**Subtel-only blocks**

| block | query sequence | | | len | aligned sequences | %ID | Named Transcripts |
|---|---|---|---|---|---|---|---|
| **1** | 1p | 1 | 25292 | 25292 | 17q: 19570 - 43429<br>5q: 15084 - 41588<br>6q: 9260 - 33611<br>16q: 12682 - 13816 | 97.61<br>98.10<br>98.42<br>97.15 | sim to protein phosphatase 1 inhibitor subunit 2 |
| **2** | 15q | 29000 | 116788 | 87789 | 19p: 20386 - 109078<br>6p: 1 - 58403<br>11p: 1 - 39455<br>6q: 66845 - 117940<br>8p: 32210 - 81762<br>1p: 67656 - 112213<br>5q: 77269 - 122537 | 98.33<br>97.97<br>98.00<br>97.86<br>97.84<br>97.92<br>97.86 | OR4F3, OR4F4, OR4F5, OR4F29, OR4F21, OR4F16, OR4F17, C6orf88 |
| **3** | 1p | 245037 | 280290 | 35254 | 8p: 194471 - 231807 | 97 | |
| **3a** | 1p | 237884 | 242735 | 4852 | 8p: 134360 - 139200 | 98 | Znf596 |
| **3b** | 1p | 231380 | 233742 | 2363 | 8p: 279211 - 281570 | 97 | FAM87B |
| **4** | 1q | 12455 | 15423 | 2969 | 6p: 95196 - 98868<br>5p: 2433 - 5242<br>4q: 8814 - 11635<br>13q: 2258 - 5129<br>4p: 17171 - 22773<br>2p: 6592 - 10454 | 93.21<br>92.86<br>90.73<br>91.40<br>91.14<br>91.24 | sim to RPL23AP7 (multiple transcripts) |
| **5** | 2p | 6601 | 23214 | 16614 | 13q: 2258 - 15153<br>21q: 19455 - 21054<br>5p: 2449 - 16995<br>4p: 17171 - 27400<br>6p: 95211 - 98868<br>1q: 12594 - 15423<br>4q: 8814 - 14548 | 91.23<br>92.00<br>91.02<br>90.49<br>91.07<br>91.22<br>90.94 | |
| **6** | 3q | 60958 | 99281 | 38324 | 9q: 25703 - 62989<br>7p: 75073 - 141501<br>16q: 105940 - 131364<br>11p: 115754 - 129425<br>17p: 1 - 21439 | 97.89<br>97.71<br>97.15<br>97.35<br>97.69 | sim to RPL23AP7 (multiple transcripts) |
| **6'** | 11p | 125961 | 136823 | 10863 | 17p: 21439 - 29146 | 97.98 | SCGB1C1 |
| **7** | 4q | 15654 | 43535 | 27882 | 10q: 14673 - 88676 | * | DUX4 |
| **8** | 4q | 111450 | 125571 | 14122 | 1p: 286892 - 291767<br>10p: 30149 - 56397<br>18p: 34540 - 42205<br>9q: 73305 - 75051<br>3q: 111837 - 116459<br>16q: 131398 - 136973 | 91.33<br>94.60<br>93.00<br>93.47<br>92.00<br>93.47 | TUBB4q |
| **9** | 5p | 4451 | 16125 | 11675 | 4q: 8933 - 14656<br>4p: 19173 - 24814<br>13q: 5070 - 11775<br>2p: 9585 - 16953 | 92<br>92<br>92.42<br>91.26 | |
| **10** | 2q | 42832 | 91779 | 48948 | 8p: 290588 - 328066 | 97.14 | FBOXO25 |
| **11** | 9q | 88452 | 124137 | 35686 | 18p: 30838 - 69538<br>10p: 39691 - 74445<br>Xq+Yq: 18812 - 30108<br>16p: 17757 - 26950<br>16q: 138869 - 143475<br>4q: 100856 - 109294 | 95.68<br>95.37<br>92.18<br>91.88<br>94.63<br>91.26 | IL9R |
| **12** | 12p | 27892 | 42389 | 14498 | 20q: 3777 - 18388 | 97.89 | IQSEC3 |
