# Supplementary material for: Human subtelomeric duplicon structure and organization
Source: Genome Biol. 2007 Jul 30;8(7):R151. doi: 10.1186/gb-2007-8-7-r151 (PMC2323237; doi:10.1186/gb-2007-8-7-r151)
Supplement: Additional data file 49 — This table shows blocks of modules that are adjacent to the ends of finished telomeres (see Materials and methods). The columns describe the same categories of information as indicated in Additional data file 48. A limited set of non-subtelomeric copies of subterminal duplicons exist (Additional data file 49). Their genomic locations suggest sites of ancestral telomere-associated chromosome rearrangements, including a well-documented telomere fusion at 2q13-q14 [37] that contains representatives of subterminal duplicon families A, B, C, and D (Additional data file 49). The non-subtelomeric site of a duplicon from family D at 3p12.3 is the tip of an extended duplication region; the DNA on the centromeric flank of this site contains 4q and 10q subtelomere homology, including beta satellite repeat structure resembling part of the D4Z4 repeat. Subterminal family F contains several non-subtelomeric sites of duplicons; those on chromosomes 22q, 14q, and 12p are very close to the respective centromeres (Additional data file 49), indicating potential ancestral inversion of a chromosome arm followed by duplication of pericentromeric sequences as a mechanism for the genesis of the non-subterminal copies of this subterminal sequence family. The sequence similarity between subterminal duplicon copies within a family is mainly in the 90-96% range for subterminal blocks A, B, and D (Table 2; see Additional data file 49 for the rare exceptions.). As with the subtel-only blocks, some of these duplicons correspond to only part of the subterminal block sequence. There is also some overlap in sequences occupied by subterminal duplicon blocks A, B, and D; this is reflected in their occupancy of parts of the same transcript families RPL23A7 and FAM41C (Table 2). The cross-family homologies between subterminal blocks A, B, and D are also in the 90-96% identity range but the positions of the duplicons within the blocks vary and are located at different distances from the (TTAGGG)n tract; [file gb-2007-8-7-r151-S49.pdf]

Additional Data File 49. Subterminal Duplicons subterm | subtel | non-subtel

Subterminal block	Tel	Size (kb)	Aligned sequences	%ID	Named Transcripts
A	2p	7	1q: 12558 - 9568	91.74	Sim to RPL23AP7, FAM41C
			4p: 15418 - 17140	91.78	
			4q: 8782 - 7060	92.15	
			16q: 7769 - 5896	92.46	
			21q: 19447 - 18443	92.35	
			10q: 21273 - 18370	92.11	
			22q: 19683 - 16700	92	
			19q: 16700 - 13690	91.94	
			9q: 25510 - 22502	91.98	
			6p: 92174 - 95168	91.76	
			11p: 112207 - 115215	92.11	
			7p: 72075 - 75083	91.98	
			3q: 60967 - 57959	92.11	
			19p: 182461 - 185461	92.05	
			18p: 91812 - 94731	92.22	
			8p: 105346 - 107957	92.65	
			16q: 104534 - 102866	91.9	
			1p: 281971 - 283465	91.24	
			chr2: 114095565 - 114098261	91.8	
B	4p	17	2p: 3558 - 6584	91.88	Sim to RPL23AP7, FAM41C
			13q: 2230 - 1374	93.1	
			10q: 21688 - 12303	90.67	
			21q: 18850 - 12378	91.12	
			6q: 7194 - 1846	91.98	
			17q: 17505 - 12165	91.5	
			5q: 13020 - 7681	91.48	
			1q: 12572 - 4690	92.57	
			16q: 7773 - 1698	91.88	
			4q: 8796 - 2760	98.39	
			19q: 16730 - 7669	92.11	
			22q: 19713 - 10659	91.95	
			5p: 622 - 2423	91.95	
			9q: 25540 - 22274	93.66	
			8p: 120622 - 123052	91.01	
			19p: 193578 - 199396	91.27	
			6q: 147519 - 145650	90.57	
			18p: 88797 - 95146	92.65	
			6p: 91533 - 95199	92.93	
			1p: 281205 - 283465	91.78	
			16q: 104534 - 102638	92.94	
			3q: 60997 - 57731	92.61	
			7p: 71847 - 75113	93.66	
			8p: 105118 - 108386	91.85	
			11p: 111979 - 115245	93.07	
			19p: 182233 - 185487	92.9	
			chr2: 114089220 - 114097873	91.9	

**C**	9p	10	15q: 10068 - 2003	98.28	Sim to MGC13005, Sim to DDX11, CXYorf1-related
			19p: 1 - 1448	99	
			16p: 1462 - 9529	98.28	
			Xq: 10578 - 2523	98.29	
			Yq: 10578 - 2523	98.29	
			chr2: 114067394 - 114075458	98.27	
**D**	10q	22	19q: 16716 - 638	95.84	Sim to RPL23AP7, FAM41C
			4q: 8796 - 2780	91.19	
			4p: 7699 - 17154	91.39	
			2p: 5115 - 6171	90.7	
			6q: 7192 - 0	94.95	
			17q: 17503 - 3109	95.1	
			5q: 13018 - 1036	95.99	
			1q: 12158 - 1763	93.51	
			21q: 19447 - 4337	96.65	
			16q: 7773 - 1704	93.24	
			22q: 19699 - 2132	95.85	
			5p: 617 - 2423	93.27	
			9q: 25526 - 22272	93.82	
			6q: 141079 - 139445	91.68	
			19p: 189683 - 199397	96.09	
			18p: 88797 - 95146	96.03	
			16q: 104534 - 102636	94.46	
			19p: 182231 - 185062	94.09	
			7p: 71845 - 75099	93.56	
			11p: 111977 - 115231	93.44	
			3q: 60983 - 57729	93.44	
			8p: 105117 - 107957	93.97	
			1p: 281205 - 283465	94.29	
			6p: 91533 - 94769	91.95	
			8p: 112649 - 123052	93.71	
			chr2: 114080795 - 114098277	95.8	
			chr3: 75756446 - 75760280	93.69	
**E**	17p	21	11p: 119534 - 128459	96.12	
			16q: 117979 - 111342	97.16	
			7p: 81837 - 90234	97.16	
			9q: 37992 - 31358	97.16	
			3q: 75707 - 67230	95.97	
**F**	18p	15	10p: 1 - 3292	99	
			1p: 283791 - 286519	93.58	
			chr1: 224029771 - 224035342	91.4	
			chr2: 132392103 - 132393925	94.07	
			chr2: 132395267 - 132399115	94.27	
			chr12: 34153760 - 34159188	94.02	
			chr14: 18424456 - 18428301	91.19	
			chr14: 18429691 - 18431501	92	
			chr22: 14843625 - 14847482	91.19	
			chr22: 14840436 - 14842245	92.24	
