# Supplementary material for: Human subtelomeric duplicon structure and organization
Source: Genome Biol. 2007 Jul 30;8(7):R151. doi: 10.1186/gb-2007-8-7-r151 (PMC2323237; doi:10.1186/gb-2007-8-7-r151)
Supplement: Additional data file 50 — Comparison of subtel-only and subterminal duplicon blocks defined in this work with the subtelomeric homology blocks reported in Linardopoulou et al. [12] [file gb-2007-8-7-r151-S50.pdf]

subtel-only block	Trask Block	Coverage of Trask block
1	Block19<br>Block28	46%<br>85%
2	Block5<br>Block3	92%<br>97%
3	Block35	91%
3a	Block34<br>Block38	84%<br>20%
3b	Block36	59%
4	Block32<br>Block39	46%<br>14%
5	Block32<br>Block39	46%<br>38%
6	Block11<br>Block12<br>Block13	95%<br>98%<br>100%
6a	Block29	72%
7	--	--
8	Block15<br>Block14	20%<br>13%
9	Block39<br>Block32	39%<br>25%
10	--	--
11	Block16<br>Block17	72%<br>93%
12	Block27	56%
**subterm block**		
A	Block25	84%
B	Block24<br>Block25	45%<br>92%
C	Block1	96%
D	Block23<br>Block24<br>Block25	36%<br>100%<br>91%
E	Block11	76%
F	Block30	96%
