# Supplementary material for: Human subtelomeric duplicon structure and organization
Source: Genome Biol. 2007 Jul 30;8(7):R151. doi: 10.1186/gb-2007-8-7-r151 (PMC2323237; doi:10.1186/gb-2007-8-7-r151)
Supplement: Additional data file 51 — Candidate transcripts were identified by blasting the representative subtelomere-only query sequences (Additional data file 48) against the NCBI RefSeq mrna database (downloaded 24 July 2006) [52]. Human mRNAs with 90% or greater homology were run through Spidey [53] against the set of subtelomere-only duplicon block representatives. This table has been filtered to those hits above 95% identity according to the Spidey predictions. The first and second columns indicate the subtelomere-only block and RefSeq accession that align to each other. The third is the description line from the RefSeq database. The fourth and fifth columns are the percent identity and percent coverage of the aligned mRNA as reported by Spidey. [file gb-2007-8-7-r151-S51.pdf]

subtel-only block	refseq accession	description	Percent ID	coverage
1	gi|88942280|ref|XM_496331.2|	PREDICTED: Homo sapiens hypothetical LOC440551 (LOC440551), mRNA, 1710 bp	100	99
1	gi|88954198|ref|XM_932777.1|	PREDICTED: Homo sapiens similar to F-box only protein 25 isoform 2, transcript variant 3 (LOC653224), mRNA, 992 bp	96.9	32
2	gi|88986529|ref|XM_929243.1|	PREDICTED: Homo sapiens similar to Olfactory receptor 4F3 (LOC653728), mRNA, 1732 bp	99.1	100
2	gi|88942152|ref|XM_927013.1|	PREDICTED: Homo sapiens similar to Olfactory receptor 4F3 (LOC653345), mRNA, 1732 bp	99.1	100
2	gi|57528065|ref|NM_001004195.2|	Homo sapiens olfactory receptor, family 4, subfamily F, member 4 (OR4F4), mRNA, 918 bp	100	100
2	gi|52421344|ref|NM_001005221.1|	Homo sapiens olfactory receptor, family 4, subfamily F, member 29 (OR4F29), mRNA, 939 bp	99.4	100
2	gi|53933273|ref|NM_001005504.1|	Homo sapiens olfactory receptor, family 4, subfamily F, member 21 (OR4F21), mRNA, 939 bp	99.4	100
2	gi|52421348|ref|NM_001005224.1|	Homo sapiens olfactory receptor, family 4, subfamily F, member 3 (OR4F3), mRNA, 939 bp	99.3	100
2	gi|52627208|ref|NM_001005277.1|	Homo sapiens olfactory receptor, family 4, subfamily F, member 16 (OR4F16), mRNA, 939 bp	99.3	100
2	gi|52546738|ref|NM_001005240.1|	Homo sapiens olfactory receptor, family 4, subfamily F, member 17 (OR4F17), mRNA, 918 bp	99.7	100
2	gi|53828739|ref|NM_001005484.1|	Homo sapiens olfactory receptor, family 4, subfamily F, member 5 (OR4F5), mRNA, 918 bp	99.3	100
2	gi|88990420|ref|XM_496791.2|	PREDICTED: Homo sapiens hypothetical LOC441123, transcript variant 1 (LOC441123), mRNA, 972 bp	99.3	27
2	gi|88990418|ref|XM_930599.1|	PREDICTED: Homo sapiens hypothetical LOC441123, transcript variant 2 (LOC441123), mRNA, 930 bp	99.3	28
2	gi|89027702|ref|XM_927320.1|	PREDICTED: Homo sapiens similar to microtubule associated serine/threonine kinase 2 (LOC644091), mRNA, 690 bp	95.6	83
3	gi|88944470|ref|XM_936770.1|	PREDICTED: Homo sapiens hypothetical protein LOC647710 (LOC647710), mRNA, 958 bp	99.8	100
3	gi|88942300|ref|XM_927114.1|	PREDICTED: Homo sapiens hypothetical protein LOC643837 (LOC643837), mRNA, 1051 bp	100	100
3	gi|89028471|ref|XM_936324.1|	PREDICTED: Homo sapiens hypothetical protein LOC642169 (LOC642169), mRNA, 958 bp	97.9	100

**3**	gi|89027710|ref|XM_927357.1|	PREDICTED: Homo sapiens hypothetical protein LOC644147 (LOC644147), mRNA, 1105 bp	97.6	100
**3b**	gi|88942298|ref|XM_927098.1|	PREDICTED: Homo sapiens family with sequence similarity 87, member B (FAM87B), mRNA, 1935 bp	100	94
**4**	gi|88952946|ref|XM_937539.1|	PREDICTED: Homo sapiens hypothetical protein LOC648490 (LOC648490), mRNA, 375 bp	98.4	65
**4**	gi|88943945|ref|XM_925877.1|	PREDICTED: Homo sapiens similar to similar to RPL23AP7 protein (LOC653081), mRNA, 279 bp	97.4	54
**6**	gi|89030138|ref|XM_927024.1|	PREDICTED: Homo sapiens similar to similar to RPL23AP7 protein (LOC653346), mRNA, 916 bp	100	45
**6**	gi|89026748|ref|XM_935631.1|	PREDICTED: Homo sapiens similar to RPL23AP7 protein (MGC70863), mRNA, 913 bp	99.5	45
**6**	gi|89040554|ref|XM_926151.1|	PREDICTED: Homo sapiens similar to similar to RPL23AP7 protein (LOC653138), mRNA, 913 bp	99	45
**6a**	gi|21717813|ref|NM_145651.1|	Homo sapiens secretoglobin, family 1C, member 1 (SCGB1C1), mRNA, 431 bp	99.5	100
**6a**	gi|89042044|ref|XM_927639.1|	PREDICTED: Homo sapiens similar to secretoglobin, family 1C, member 1 (LOC653486), mRNA, 424 bp	98.1	100
**8**	gi|55770867|ref|NM_020040.3|	Homo sapiens tubulin, beta polypeptide 4, member Q (TUBB4Q), mRNA, 1305 bp	99.9	100
**8**	gi|88981265|ref|XM_945046.1|	PREDICTED: Homo sapiens similar to Tubulin beta-4q chain, transcript variant 3 (LOC649679), mRNA, 2102 bp	98	100
**8**	gi|88981262|ref|XM_945045.1|	PREDICTED: Homo sapiens similar to Tubulin beta-4q chain, transcript variant 2 (LOC649679), mRNA, 1348 bp	97.4	100
**8**	gi|88981268|ref|XM_941232.1|	PREDICTED: Homo sapiens similar to Tubulin beta-4q chain, transcript variant 1 (LOC649679), mRNA, 1713 bp	97.6	100
**8**	gi|89030119|ref|XM_932731.1|	PREDICTED: Homo sapiens similar to tubulin, beta 8, transcript variant 4 (LOC643224), mRNA, 1572 bp	95.2	100
**8**	gi|89030123|ref|XM_932740.1|	PREDICTED: Homo sapiens similar to tubulin, beta 8, transcript variant 6 (LOC643224), mRNA, 2199 bp	95.3	100
**8**	gi|89030121|ref|XM_932733.1|	PREDICTED: Homo sapiens similar to tubulin, beta 8, transcript variant 5 (LOC643224), mRNA, 1422 bp	95.1	100
**8**	gi|89030115|ref|XM_928232.1|	PREDICTED: Homo sapiens similar to tubulin, beta 8, transcript variant 1 (LOC643224), mRNA, 2225 bp	95.1	100
**8**	gi|89030113|ref|XM_932717.1|	PREDICTED: Homo sapiens similar to tubulin, beta 8, transcript variant 2 (LOC643224), mRNA, 1786 bp	95.5	100
**8**	gi|42558278|ref|NM_177987.1|	Homo sapiens tubulin, beta 8 (TUBB8), mRNA, 1335 bp	95.1	100

**8**	gi|89030117|ref|XM_932725.1|	PREDICTED: Homo sapiens similar to tubulin, beta 8, transcript variant 3 (LOC643224), mRNA, 1342 bp	96	100
**10**	gi|88954194|ref|XM_932773.1|	PREDICTED: Homo sapiens similar to F-box only protein 25 isoform 2, transcript variant 2 (LOC653224), mRNA, 545 bp	100	47
**12**	gi|89057930|ref|XM_375928.3|	PREDICTED: Homo sapiens similar to IQ motif and Sec7 domain 3 (LOC400855), mRNA, 2723 bp	99.2	100
**12**	gi|89077212|ref|XM_942369.1|	PREDICTED: Homo sapiens IQ motif and Sec7 domain 3 (IQSEC3), mRNA, 558 bp	99.6	100
