# Supplementary material for: Human subtelomeric duplicon structure and organization
Source: Genome Biol. 2007 Jul 30;8(7):R151. doi: 10.1186/gb-2007-8-7-r151 (PMC2323237; doi:10.1186/gb-2007-8-7-r151)
Supplement: Additional data file 52 — Candidate transcripts were identified by blasting the representative subterminal query sequences (Additional data file 49) against the NCBI RefSeq mrna database (downloaded 24 July 2006) [52]. Human mRNAs with 90% or greater homology were run through Spidey [53] against the set of subterminal duplicon block representatives. The first and second columns indicate the subterminal block and RefSeq accession that align to each other. The third is the description line from the RefSeq database. The fourth and fifth columns are the percent identity and percent coverage of the aligned mRNA as reported by Spidey. [file gb-2007-8-7-r151-S52.pdf]

Subterm Block	Accession	Description	Percent ID	Coverage
**A**	gi|89050223|ref|XM_928041.1|	PREDICTED: Homo sapiens hypothetical protein LOC644964 (LOC644964), mRNA, 531 bp	92.1	100
**A**	gi|88944468|ref|XM_936761.1|	PREDICTED: Homo sapiens family with sequence similarity 41, member C (FAM41C), mRNA, 660 bp	91.6	75
**A**	gi|88942302|ref|XM_496333.2|	PREDICTED: Homo sapiens family with sequence similarity 41, member C (FAM41C), mRNA, 660 bp	91.6	75
**A**	gi|89033725|ref|XM_925804.1|	PREDICTED: Homo sapiens similar to similar to RPL23AP7 protein (LOC642257), mRNA, 529 bp	91.9	100
**A**	gi|89027706|ref|XM_927336.1|	PREDICTED: Homo sapiens hypothetical protein LOC644113 (LOC644113), mRNA, 531 bp	91.1	100
**A**	gi|89026748|ref|XM_935631.1|	PREDICTED: Homo sapiens similar to RPL23AP7 protein (MGC70863), mRNA, 913 bp	91.2	54
**A**	gi|89040554|ref|XM_926151.1|	PREDICTED: Homo sapiens similar to similar to RPL23AP7 protein (LOC653138), mRNA, 913 bp	91.2	54
**A**	gi|89030138|ref|XM_927024.1|	PREDICTED: Homo sapiens similar to similar to RPL23AP7 protein (LOC653346), mRNA, 916 bp	91.2	54
**A**	gi|89024636|ref|XM_928275.1|	PREDICTED: Homo sapiens similar to similar to RPL23AP7 protein (LOC645236), mRNA, 526 bp	91.3	100
**A**	gi|89027700|ref|XM_927280.1|	PREDICTED: Homo sapiens similar to similar to RPL23AP7 protein (LOC644033), mRNA, 531 bp	91	100
**A**	gi|89066164|ref|XM_939197.1|	PREDICTED: Homo sapiens similar to similar to RPL23AP7 protein (LOC650103), mRNA, 531 bp	90.8	100
**A**	gi|89040556|ref|XM_926164.1|	PREDICTED: Homo sapiens hypothetical protein LOC642720 (LOC642720), mRNA, 531 bp	90.6	100
**A**	gi|88952946|ref|XM_937539.1|	PREDICTED: Homo sapiens hypothetical protein LOC648490 (LOC648490), mRNA, 375 bp	94.3	37
**A**	gi|88943945|ref|XM_925877.1|	PREDICTED: Homo sapiens similar to similar to RPL23AP7 protein (LOC653081), mRNA, 279 bp	94.3	50
**B**	gi|89027700|ref|XM_927280.1|	PREDICTED: Homo sapiens similar to similar to RPL23AP7 protein (LOC644033), mRNA, 531 bp	94.6	55
**B**	gi|89066164|ref|XM_939197.1|	PREDICTED: Homo sapiens similar to similar to RPL23AP7 protein (LOC650103), mRNA, 531 bp	94.2	55
**B**	gi|89050223|ref|XM_928041.1|	PREDICTED: Homo sapiens hypothetical protein LOC644964 (LOC644964), mRNA, 531 bp	93.1	59
**B**	gi|89040556|ref|XM_926164.1|	PREDICTED: Homo sapiens hypothetical protein LOC642720 (LOC642720), mRNA, 531 bp	93.9	55
**B**	gi|89033725|ref|XM_925804.1|	PREDICTED: Homo sapiens similar to similar to RPL23AP7 protein (LOC642257), mRNA, 529 bp	93.4	60

**B**	gi|88944468|ref|XM_936761.1|	PREDICTED: Homo sapiens family with sequence similarity 41, member C (FAM41C), mRNA, 660 bp	94	48
**B**	gi|88942302|ref|XM_496333.2|	PREDICTED: Homo sapiens family with sequence similarity 41, member C (FAM41C), mRNA, 660 bp	94	48
**B**	gi|89027706|ref|XM_927336.1|	PREDICTED: Homo sapiens hypothetical protein LOC644113 (LOC644113), mRNA, 531 bp	92.1	59
**B**	gi|89026748|ref|XM_935631.1|	PREDICTED: Homo sapiens similar to RPL23AP7 protein (MGC70863), mRNA, 913 bp	92.4	34
**B**	gi|89040554|ref|XM_926151.1|	PREDICTED: Homo sapiens similar to similar to RPL23AP7 protein (LOC653138), mRNA, 913 bp	92.4	34
**B**	gi|89030138|ref|XM_927024.1|	PREDICTED: Homo sapiens similar to similar to RPL23AP7 protein (LOC653346), mRNA, 916 bp	92.4	34
**B**	gi|89024636|ref|XM_928275.1|	PREDICTED: Homo sapiens similar to similar to RPL23AP7 protein (LOC645236), mRNA, 526 bp	92.4	59
**C**	gi|89029107|ref|XM_931012.1|	PREDICTED: Homo sapiens CXYorf1-related protein, transcript variant 43 (FLJ00038), mRNA, 10198 bp	100	48
**C**	gi|89029081|ref|XM_930913.1|	PREDICTED: Homo sapiens CXYorf1-related protein, transcript variant 31 (FLJ00038), mRNA, 10957 bp	100	52
**C**	gi|89029067|ref|XM_930858.1|	PREDICTED: Homo sapiens CXYorf1-related protein, transcript variant 24 (FLJ00038), mRNA, 10467 bp	100	54
**C**	gi|89029065|ref|XM_930847.1|	PREDICTED: Homo sapiens CXYorf1-related protein, transcript variant 23 (FLJ00038), mRNA, 10553 bp	100	54
**C**	gi|89029063|ref|XM_930839.1|	PREDICTED: Homo sapiens CXYorf1-related protein, transcript variant 22 (FLJ00038), mRNA, 10338 bp	100	49
**C**	gi|89029053|ref|XM_930782.1|	PREDICTED: Homo sapiens CXYorf1-related protein, transcript variant 17 (FLJ00038), mRNA, 10147 bp	100	48
**C**	gi|89029051|ref|XM_930773.1|	PREDICTED: Homo sapiens CXYorf1-related protein, transcript variant 16 (FLJ00038), mRNA, 10238 bp	100	48
**C**	gi|89029049|ref|XM_930763.1|	PREDICTED: Homo sapiens CXYorf1-related protein, transcript variant 15 (FLJ00038), mRNA, 9383 bp	100	60
**C**	gi|89029077|ref|XM_930901.1|	PREDICTED: Homo sapiens CXYorf1-related protein, transcript variant 29 (FLJ00038), mRNA, 11093 bp	100	52
**C**	gi|89029155|ref|XM_376814.3|	PREDICTED: Homo sapiens hypothetical protein MGC13005 (MGC13005), mRNA, 3992 bp	100	100
**C**	gi|89060451|ref|XM_933164.1|	PREDICTED: Homo sapiens similar to CXYorf1-related protein, transcript variant 27 (LOC653440), mRNA, 4919 bp	98.3	100
**C**	gi|89060449|ref|XM_933161.1|	PREDICTED: Homo sapiens similar to CXYorf1-related protein, transcript variant 26 (LOC653440), mRNA, 4260 bp	98.3	100

**C**	gi|89060437|ref|XM_933136.1|	PREDICTED: Homo sapiens similar to CXYorf1-related protein, transcript variant 20 (LOC653440), mRNA, 5678 bp	98.4	100
**C**	gi|89060419|ref|XM_933090.1|	PREDICTED: Homo sapiens similar to CXYorf1-related protein, transcript variant 11 (LOC653440), mRNA, 5059 bp	98.3	100
**C**	gi|89060415|ref|XM_933073.1|	PREDICTED: Homo sapiens similar to CXYorf1-related protein, transcript variant 9 (LOC653440), mRNA, 5159 bp	98.3	100
**C**	gi|89060413|ref|XM_933069.1|	PREDICTED: Homo sapiens similar to CXYorf1-related protein, transcript variant 8 (LOC653440), mRNA, 4868 bp	98.3	100
**C**	gi|89060409|ref|XM_933062.1|	PREDICTED: Homo sapiens similar to CXYorf1-related protein, transcript variant 6 (LOC653440), mRNA, 4959 bp	98.3	100
**C**	gi|89060401|ref|XM_933053.1|	PREDICTED: Homo sapiens similar to CXYorf1-related protein, transcript variant 2 (LOC653440), mRNA, 3435 bp	98.6	100
**C**	gi|88942112|ref|XM_926939.1|	PREDICTED: Homo sapiens similar to DEAD/H (Asp-Glu-Ala-Asp/His) box polypeptide 11 isoform 1 (LOC643635), mRNA, 4032 bp	98.5	100
**C**	gi|89038711|ref|XM_928159.1|	PREDICTED: Homo sapiens similar to DEAD/H (Asp-Glu-Ala-Asp/His) box polypeptide 11 isoform 1 (LOC645128), mRNA, 4001 bp	98.4	100
**C**	gi|89029109|ref|XM_931018.1|	PREDICTED: Homo sapiens CXYorf1-related protein, transcript variant 44 (FLJ00038), mRNA, 9365 bp	100	44
**C**	gi|89029087|ref|XM_930941.1|	PREDICTED: Homo sapiens CXYorf1-related protein, transcript variant 34 (FLJ00038), mRNA, 10776 bp	100	51
**C**	gi|89060453|ref|XM_933171.1|	PREDICTED: Homo sapiens similar to CXYorf1-related protein, transcript variant 28 (LOC653440), mRNA, 4083 bp	98.2	100
**C**	gi|89060439|ref|XM_933140.1|	PREDICTED: Homo sapiens similar to CXYorf1-related protein, transcript variant 21 (LOC653440), mRNA, 5501 bp	98.3	100
**C**	gi|89029099|ref|XM_930979.1|	PREDICTED: Homo sapiens CXYorf1-related protein, transcript variant 39 (FLJ00038), mRNA, 10159 bp	100	48
**C**	gi|89029097|ref|XM_930971.1|	PREDICTED: Homo sapiens CXYorf1-related protein, transcript variant 38 (FLJ00038), mRNA, 10299 bp	100	49
**C**	gi|89029093|ref|XM_930957.1|	PREDICTED: Homo sapiens CXYorf1-related protein, transcript variant 36 (FLJ00038), mRNA, 10398 bp	100	49
**C**	gi|89029079|ref|XM_930907.1|	PREDICTED: Homo sapiens CXYorf1-related protein, transcript variant 30 (FLJ00038), mRNA, 10023 bp	100	47
**C**	gi|89029075|ref|XM_930896.1|	PREDICTED: Homo sapiens CXYorf1-related protein, transcript variant 28 (FLJ00038), mRNA, 10405 bp	100	49

**C**	gi|89029071|ref|XM_930877.1|	PREDICTED: Homo sapiens CXYorf1-related protein, transcript variant 26 (FLJ00038), mRNA, 10036 bp	100	47
**C**	gi|89029039|ref|XM_930711.1|	PREDICTED: Homo sapiens CXYorf1-related protein, transcript variant 10 (FLJ00038), mRNA, 4038 bp	100	96
**C**	gi|89029037|ref|XM_930700.1|	PREDICTED: Homo sapiens CXYorf1-related protein, transcript variant 9 (FLJ00038), mRNA, 3533 bp	100	95
**C**	gi|89029031|ref|XM_930659.1|	PREDICTED: Homo sapiens CXYorf1-related protein, transcript variant 6 (FLJ00038), mRNA, 3360 bp	100	95
**C**	gi|89029157|ref|XM_928555.1|	PREDICTED: Homo sapiens similar to hypothetical protein MGC13005 (LOC645536), mRNA, 2888 bp	100	100
**C**	gi|89029085|ref|XM_930933.1|	PREDICTED: Homo sapiens CXYorf1-related protein, transcript variant 33 (FLJ00038), mRNA, 9643 bp	100	45
**C**	gi|89029111|ref|XM_931024.1|	PREDICTED: Homo sapiens CXYorf1-related protein, transcript variant 45 (FLJ00038), mRNA, 9126 bp	100	42
**C**	gi|89029101|ref|XM_930989.1|	PREDICTED: Homo sapiens CXYorf1-related protein, transcript variant 40 (FLJ00038), mRNA, 9646 bp	100	45
**C**	gi|89060445|ref|XM_933153.1|	PREDICTED: Homo sapiens similar to CXYorf1-related protein, transcript variant 24 (LOC653440), mRNA, 4347 bp	98.5	100
**C**	gi|89060435|ref|XM_933132.1|	PREDICTED: Homo sapiens similar to CXYorf1-related protein, transcript variant 19 (LOC653440), mRNA, 4487 bp	98.4	100
**C**	gi|89060433|ref|XM_933128.1|	PREDICTED: Homo sapiens similar to CXYorf1-related protein, transcript variant 18 (LOC653440), mRNA, 4743 bp	98.4	100
**C**	gi|89060431|ref|XM_933121.1|	PREDICTED: Homo sapiens similar to CXYorf1-related protein, transcript variant 17 (LOC653440), mRNA, 5126 bp	98.2	100
**C**	gi|89060429|ref|XM_933115.1|	PREDICTED: Homo sapiens similar to CXYorf1-related protein, transcript variant 16 (LOC653440), mRNA, 4753 bp	98.5	100
**C**	gi|89060421|ref|XM_933094.1|	PREDICTED: Homo sapiens similar to CXYorf1-related protein, transcript variant 12 (LOC653440), mRNA, 3102 bp	98.2	99
**C**	gi|88942114|ref|XM_926948.1|	PREDICTED: Homo sapiens similar to hypothetical protein MGC13005 (LOC643643), mRNA, 2928 bp	98.5	100
**C**	gi|89038709|ref|XM_928155.1|	PREDICTED: Homo sapiens similar to hypothetical protein MGC13005 (LOC645124), mRNA, 2897 bp	98.2	100
**C**	gi|89060459|ref|XM_933185.1|	PREDICTED: Homo sapiens similar to CXYorf1-related protein, transcript variant 31 (LOC653440), mRNA, 3844 bp	98.2	100

**C**	gi|89060455|ref|XM_933177.1|	PREDICTED: Homo sapiens similar to CXYorf1-related protein, transcript variant 29 (LOC653440), mRNA, 3708 bp	98.1	100
**C**	gi|89029069|ref|XM_930868.1|	PREDICTED: Homo sapiens CXYorf1-related protein, transcript variant 25 (FLJ00038), mRNA, 8888 bp	100	41
**C**	gi|89029057|ref|XM_930803.1|	PREDICTED: Homo sapiens CXYorf1-related protein, transcript variant 19 (FLJ00038), mRNA, 9123 bp	100	42
**C**	gi|89029095|ref|XM_930965.1|	PREDICTED: Homo sapiens CXYorf1-related protein, transcript variant 37 (FLJ00038), mRNA, 9863 bp	100	46
**C**	gi|89029027|ref|XM_930631.1|	PREDICTED: Homo sapiens CXYorf1-related protein, transcript variant 4 (FLJ00038), mRNA, 2289 bp	100	99
**C**	gi|89029105|ref|XM_931007.1|	PREDICTED: Homo sapiens CXYorf1-related protein, transcript variant 42 (FLJ00038), mRNA, 9264 bp	100	43
**C**	gi|89029073|ref|XM_930885.1|	PREDICTED: Homo sapiens CXYorf1-related protein, transcript variant 27 (FLJ00038), mRNA, 9440 bp	100	44
**C**	gi|89029033|ref|XM_930674.1|	PREDICTED: Homo sapiens CXYorf1-related protein, transcript variant 7 (FLJ00038), mRNA, 2049 bp	100	92
**C**	gi|89029113|ref|XM_931031.1|	PREDICTED: Homo sapiens CXYorf1-related protein, transcript variant 46 (FLJ00038), mRNA, 8215 bp	100	36
**C**	gi|89029103|ref|XM_930998.1|	PREDICTED: Homo sapiens CXYorf1-related protein, transcript variant 41 (FLJ00038), mRNA, 8608 bp	100	39
**C**	gi|89029091|ref|XM_926088.1|	PREDICTED: Homo sapiens CXYorf1-related protein, transcript variant 1 (FLJ00038), mRNA, 2473 bp	100	83
**C**	gi|89029089|ref|XM_930947.1|	PREDICTED: Homo sapiens CXYorf1-related protein, transcript variant 35 (FLJ00038), mRNA, 9507 bp	100	44
**C**	gi|89029083|ref|XM_930924.1|	PREDICTED: Homo sapiens CXYorf1-related protein, transcript variant 32 (FLJ00038), mRNA, 8595 bp	100	39
**C**	gi|89029061|ref|XM_930826.1|	PREDICTED: Homo sapiens CXYorf1-related protein, transcript variant 21 (FLJ00038), mRNA, 8983 bp	100	41
**C**	gi|89029059|ref|XM_930816.1|	PREDICTED: Homo sapiens CXYorf1-related protein, transcript variant 20 (FLJ00038), mRNA, 2411 bp	100	94
**C**	gi|89029055|ref|XM_930792.1|	PREDICTED: Homo sapiens CXYorf1-related protein, transcript variant 18 (FLJ00038), mRNA, 7388 bp	100	29
**C**	gi|89029047|ref|XM_930756.1|	PREDICTED: Homo sapiens CXYorf1-related protein, transcript variant 14 (FLJ00038), mRNA, 8494 bp	100	38
**C**	gi|89029045|ref|XM_930746.1|	PREDICTED: Homo sapiens CXYorf1-related protein, transcript variant 13 (FLJ00038), mRNA, 8437 bp	100	37
**C**	gi|89029043|ref|XM_930734.1|	PREDICTED: Homo sapiens CXYorf1-related protein, transcript variant 12 (FLJ00038), mRNA, 2442 bp	100	93

**C**	gi|89029041|ref|XM_930724.1|	PREDICTED: Homo sapiens CXYorf1-related protein, transcript variant 11 (FLJ00038), mRNA, 1931 bp	100	92
**C**	gi|89029035|ref|XM_930687.1|	PREDICTED: Homo sapiens CXYorf1-related protein, transcript variant 8 (FLJ00038), mRNA, 2442 bp	100	93
**C**	gi|89029025|ref|XM_930619.1|	PREDICTED: Homo sapiens CXYorf1-related protein, transcript variant 3 (FLJ00038), mRNA, 8259 bp	100	36
**C**	gi|89060411|ref|XM_933065.1|	PREDICTED: Homo sapiens similar to CXYorf1-related protein, transcript variant 7 (LOC653440), mRNA, 2282 bp	97.9	99
**C**	gi|89060425|ref|XM_933104.1|	PREDICTED: Homo sapiens similar to CXYorf1-related protein, transcript variant 14 (LOC653440), mRNA, 3828 bp	98.4	100
**C**	gi|89060417|ref|XM_933080.1|	PREDICTED: Homo sapiens similar to CXYorf1-related protein, transcript variant 10 (LOC653440), mRNA, 3628 bp	98.4	100
**C**	gi|89029029|ref|XM_930643.1|	PREDICTED: Homo sapiens CXYorf1-related protein, transcript variant 5 (FLJ00038), mRNA, 7909 bp	100	33
**C**	gi|89060461|ref|XM_377073.2|	PREDICTED: Homo sapiens similar to CXYorf1-related protein, transcript variant 1 (LOC653440), mRNA, 2929 bp	98.4	100
**C**	gi|89060457|ref|XM_933181.1|	PREDICTED: Homo sapiens similar to CXYorf1-related protein, transcript variant 30 (LOC653440), mRNA, 3325 bp	98.3	100
**C**	gi|89060447|ref|XM_933157.1|	PREDICTED: Homo sapiens similar to CXYorf1-related protein, transcript variant 25 (LOC653440), mRNA, 3696 bp	98.4	100
**C**	gi|89060441|ref|XM_933144.1|	PREDICTED: Homo sapiens similar to CXYorf1-related protein, transcript variant 22 (LOC653440), mRNA, 3208 bp	98.5	100
**C**	gi|89060427|ref|XM_933107.1|	PREDICTED: Homo sapiens similar to CXYorf1-related protein, transcript variant 15 (LOC653440), mRNA, 3688 bp	98.5	100
**C**	gi|89060423|ref|XM_933099.1|	PREDICTED: Homo sapiens similar to CXYorf1-related protein, transcript variant 13 (LOC653440), mRNA, 2776 bp	98.4	100
**C**	gi|89060407|ref|XM_933060.1|	PREDICTED: Homo sapiens similar to CXYorf1-related protein, transcript variant 5 (LOC653440), mRNA, 3836 bp	98.3	100
**C**	gi|89060405|ref|XM_933059.1|	PREDICTED: Homo sapiens similar to CXYorf1-related protein, transcript variant 4 (LOC653440), mRNA, 3151 bp	98.4	100
**C**	gi|89060403|ref|XM_933055.1|	PREDICTED: Homo sapiens similar to CXYorf1-related protein, transcript variant 3 (LOC653440), mRNA, 2104 bp	99	100
**C**	gi|89060443|ref|XM_933149.1|	PREDICTED: Homo sapiens similar to CXYorf1-related protein, transcript variant 23 (LOC653440), mRNA, 1523 bp	97.5	99

**C**	gi|40353752|ref|NM_199163.2|	Homo sapiens CXYorf1-related protein (FLJ25222), mRNA, 2579 bp	98.3	61
**C**	gi|39573729|ref|NM_198943.1|	Homo sapiens CXYorf1-related protein (MGC52000), mRNA, 1748 bp	97.1	90
**C**	gi|89029023|ref|XM_930604.1|	PREDICTED: Homo sapiens CXYorf1-related protein, transcript variant 2 (FLJ00038), mRNA, 1585 bp	100	73
**C**	gi|39573706|ref|NM_182905.1|	Homo sapiens CXYorf1-related protein (LOC349338), mRNA, 795 bp	98.7	100
**D**	gi|89056981|ref|XM_929847.1|	PREDICTED: Homo sapiens similar to 60S ribosomal protein L23a (LOC653789), mRNA, 885 bp	99	100
**D**	gi|45238850|ref|NM_203477.1|	Homo sapiens similar to RPL23AP7 protein (MGC70863), transcript variant 1, mRNA, 1389 bp	96.6	70
**D**	gi|45238852|ref|NM_203302.2|	Homo sapiens similar to RPL23AP7 protein (MGC70863), transcript variant 2, mRNA, 1249 bp	98.7	68
**D**	gi|89042049|ref|XM_371204.4|	PREDICTED: Homo sapiens similar to 60S ribosomal protein L23a (LOC388574), mRNA, 4062 bp	95.9	73
**D**	gi|25141250|ref|NR_000029.1|	Homo sapiens ribosomal protein L23a pseudogene 7 (RPL23AP7) on chromosome 2, 969 bp	98.3	80
**D**	gi|88942148|ref|XM_926984.1|	PREDICTED: Homo sapiens similar to 60S ribosomal protein L23a (LOC653341), mRNA, 823 bp	98.5	100
**D**	gi|89027708|ref|XM_927344.1|	PREDICTED: Homo sapiens hypothetical protein LOC644128 (LOC644128), mRNA, 4440 bp	94.1	65
**D**	gi|89037197|ref|XM_939563.1|	PREDICTED: Homo sapiens similar to 60S ribosomal protein L23a (LOC650472), mRNA, 805 bp	95.4	100
**D**	gi|88969474|ref|XM_936392.1|	PREDICTED: Homo sapiens similar to RPL23AP7 protein (MGC70863), mRNA, 704 bp	95.3	100
**D**	gi|78190460|ref|NM_000984.5|	Homo sapiens ribosomal protein L23a (RPL23A), mRNA, 979 bp	91	52
**D**	gi|88971265|ref|XM_941396.1|	PREDICTED: Homo sapiens similar to 60S ribosomal protein L23a (LOC652071), mRNA, 582 bp	90.8	88
**D**	gi|88965928|ref|XM_930113.1|	PREDICTED: Homo sapiens similar to 60S ribosomal protein L23a (LOC647099), mRNA, 582 bp	90.8	88
**D**	gi|89057256|ref|XM_937620.1|	PREDICTED: Homo sapiens similar to 60S ribosomal protein L23a (LOC401904), mRNA, 960 bp	90.9	50
**D**	gi|89052442|ref|XM_377521.3|	PREDICTED: Homo sapiens similar to 60S ribosomal protein L23a (LOC401904), mRNA, 960 bp	90.9	50
**D**	gi|89033725|ref|XM_925804.1|	PREDICTED: Homo sapiens similar to similar to RPL23AP7 protein (LOC642257), mRNA, 529 bp	95.1	100
**D**	gi|89050223|ref|XM_928041.1|	PREDICTED: Homo sapiens hypothetical protein LOC644964 (LOC644964), mRNA, 531 bp	94.9	100
**D**	gi|88944468|ref|XM_936761.1|	PREDICTED: Homo sapiens family with sequence similarity 41, member C (FAM41C), mRNA, 660 bp	95.2	76

**D**	gi|88942302|ref|XM_496333.2|	PREDICTED: Homo sapiens family with sequence similarity 41, member C (FAM41C), mRNA, 660 bp	95.2	76
**D**	gi|89030138|ref|XM_927024.1|	PREDICTED: Homo sapiens similar to similar to RPL23AP7 protein (LOC653346), mRNA, 916 bp	95.2	55
**D**	gi|89027706|ref|XM_927336.1|	PREDICTED: Homo sapiens hypothetical protein LOC644113 (LOC644113), mRNA, 531 bp	94.4	100
**D**	gi|89027700|ref|XM_927280.1|	PREDICTED: Homo sapiens similar to similar to RPL23AP7 protein (LOC644033), mRNA, 531 bp	94.4	100
**D**	gi|89026748|ref|XM_935631.1|	PREDICTED: Homo sapiens similar to RPL23AP7 protein (MGC70863), mRNA, 913 bp	95.2	54
**D**	gi|89040554|ref|XM_926151.1|	PREDICTED: Homo sapiens similar to similar to RPL23AP7 protein (LOC653138), mRNA, 913 bp	95.2	54
**D**	gi|89024636|ref|XM_928275.1|	PREDICTED: Homo sapiens similar to similar to RPL23AP7 protein (LOC645236), mRNA, 526 bp	95.1	100
**D**	gi|89066164|ref|XM_939197.1|	PREDICTED: Homo sapiens similar to similar to RPL23AP7 protein (LOC650103), mRNA, 531 bp	94.2	100
**D**	gi|89040556|ref|XM_926164.1|	PREDICTED: Homo sapiens hypothetical protein LOC642720 (LOC642720), mRNA, 531 bp	94	100
**F**	gi|88957849|ref|XM_943168.1|	PREDICTED: Homo sapiens hypothetical protein LOC647989 (LOC647989), mRNA, 726 bp	93.2	96
**F**	gi|47551355|ref|NM_214461.1|	Homo sapiens MGC50273 protein (MGC50273), mRNA, 1429 bp	93.3	40
